# Associations Between Dietary Factors, Metabolic Factors, Sleep Disorders, Physical Activity, and the Risk of Multiple Sclerosis: A Univariable and Multivariable Mendelian Randomization Study

**DOI:** 10.1002/fsn3.70905

**Published:** 2025-09-11

**Authors:** Hongwei Liu, Minghui Wu, Jie Yang, Minheng Zhang, Xuan Chen, Haixia Fan

**Affiliations:** ^1^ Department of Neurology Taiyuan City Central Hospital, the Ninth Clinical Medical College of Shanxi Medical University Taiyuan Shanxi Province China; ^2^ Department of Gerontology The First People's Hospital of Jinzhong, Yuci Shanxi Province China; ^3^ Department of Sleep Center First Hospital of Shanxi Medical University Taiyuan Shanxi Province China

**Keywords:** dietary factors, mendelian randomization, metabolic factors, multiple sclerosis, physical activity, sleep disorders

## Abstract

Observational studies propose associations between sleep disorders, dietary factors, metabolic factors, physical activity, and multiple sclerosis (MS). However, the causal nature of these relationships remains unclear. This study aims to determine whether these factors causally influence MS risk through Mendelian randomization (MR) analysis. We utilized two‐sample MR to investigate the causal relationships between 29 distinct traits related to sleep disorders, dietary factors, metabolic factors, physical activity, and their impact on MS. The inverse‐variance weighted (IVW) method served as the primary analytical tool to estimate causality. Univariate Mendelian randomization analyses (UVMR) demonstrated that higher genetically predicted chronotype (OR = 0.571; *p* = 0.044), pork intake (OR = 6.764; *p* = 0.005), fish/liver oil dietary supplements (OR = 0.119, *p* = 0.002), body mass index (BMI, OR = 1.347; *p* = 0.004), type 2 diabetes (T2DM, OR = 1.122; *p* = 0.032), hip circumference (OR = 1.267; *p* = 0.023), short sleep duration (OR = 0.170; *p* = 0.013), and moderate to vigorous physical activity (MVPA, OR = 0.282; *p* < 0.001) were significantly associated with the risk of MS. In the multivariate Mendelian randomization (MVMR) analyses, the significant causal associations between pork intake, BMI, T2DM, and MVPA with MS were robust even after adjusting for potential confounding variables. However, after adjusting for these confounders, the initially observed causal links between the remaining four exposure factors and MS were no longer statistically significant. This study suggests that certain factors may have a causal relationship with MS risk. Specifically, increased participation in moderate to vigorous physical activity may reduce the risk of MS, whereas higher BMI, greater pork intake, and a history of T2DM may increase the risk of MS.

## Graphical abstract

Mendelian randomization analysis identified causal links between dietary factors, sleep disorders, physical activity, and metabolic factors and multiple sclerosis risk. Higher pork intake, body mass index, and type 2 diabetes increased multiple sclerosis risk, whereas greater moderate‐to‐vigorous physical activity reduced risk. These findings underscore lifestyle and metabolic modifications as potential strategies for MS prevention.

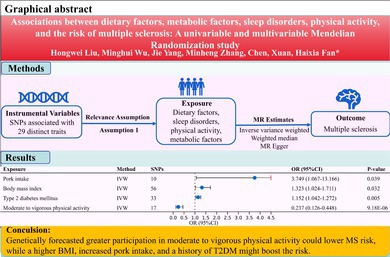

## 1 Introduction

Pathological demyelination caused by inflammation in multiple sclerosis (MS) results in axonal degeneration and neuron loss in the central nervous system (CNS) (Stampanoni Bassi et al. 2022). In 2021, the Global Burden of Disease Study (Khan and Hashim 2025) found that approximately 1.89 million individuals across the globe were living with MS, with a prevalence of 23.9 per 100,000 population. In the past three decades, this burden has been on the rise, especially in affluent countries, emphasizing the urgent necessity to identify modifiable risk factors and the underlying mechanisms (Khan and Hashim 2025). The disease typically presents as a relapsing–remitting condition, manifesting with sudden or gradual onset of neurological symptoms such as impaired visual acuity, sensory or motor deficits, ataxia, and bladder dysfunction. These symptoms often lessen in severity or resolve completely in the early stages of the disease (Dobson and Giovannoni 2019). The origins of MS are believed to involve both genetic and environmental components, though the precise etiopathology is not known. Polymorphisms in the genetic regions responsible for the vitamin D receptor, interleukin‐10 (IL‐10), tumor necrosis factor‐alpha (TNF‐α), and interferon‐gamma are significant genetic contributors (Pourostadi et al. 2021; Asgharzadeh, Najafi‐Ghalehlou, et al. 2021; Asgharzadeh, Sanajoo, et al. 2021). Genetic contributors alone cannot fully account for the heightened risk associated with this disease; environmental factors are also thought to significantly influence the escalating likelihood of developing MS (Rodríguez Murúa et al. 2022). However, genetic predisposition alone does not fully explain the increased risk of MS, suggesting a significant role of environmental influences. Environmental factors, in contrast to genetic ones, are modifiable and, therefore, critical in preventing and managing MS progression. These factors include sunlight exposure, tobacco use, adolescent obesity, night shifts, Epstein–Barr virus infection, and nutritional intake (Olsson et al. 2017). Notably, some environmental factors can have dual effects, either beneficial or detrimental, depending on specific circumstances. Nutrition is one such factor that impacts MS risk positively or negatively. For instance, a low‐calorie diet rich in polyunsaturated fatty acids is associated with reduced MS risks, whereas adolescent obesity and plasma vitamin D deficiency are linked to increased risks (Langley et al. 2020). Research similarly points out that inadequate sleep and poor sleep quality are associated with a higher chance of developing MS (Åkerstedt et al. 2023; Johansson et al. 2024). Moreover, adolescents who participate in higher levels of vigorous physical activity tend to have a decreased risk of developing MS across various European regions (Wesnes et al. 2018). The findings from a population‐based cohort study demonstrated an increased risk of MS in patients of both genders with Type 2 Diabetes Mellitus (T2DM), with a marked increase in risk for women below the age of 50 (Hou et al. 2017).

Mendelian randomization (MR) has recently become a valuable technique for identifying causal relationships between adjustable risk factors and complex diseases, including multiple sclerosis MS (Belbasis et al. 2020). An expanding collection of MR‐based findings has furthered our insight into MS etiology by revealing potential causal connections with obesity, physical inactivity, and various dietary influences. Jacobs et al. reported that increased BMI and reduced vitamin D levels correlate with a higher risk of MS (Jacobs et al. 2020), and Harroud et al. delved into the unique roles of leptin and adiponectin in these mechanisms (Harroud et al. 2021). The potential protective role of physical activity is gaining attention (Li et al. 2022), whereas smoking's influence is now regarded as less significant than earlier believed (Mitchell et al. 2020). More recently, dietary components have been highlighted: pork intake emerged as a new risk factor, and fish oil supplementation was found to have protective associations (Ye et al. 2025). Fazia et al. and Vandebergh et al. have analyzed these findings, pointing out MR's utility in merging observational research with causal inference (Vandebergh, Becelaere, et al. 2022; Fazia et al. 2023).

Nonetheless, there are still crucial gaps. The majority of past MR studies have analyzed individual exposures in isolation, often failing to consider the complex nature of MS pathogenesis and the likelihood of shared genetic frameworks among interconnected traits. Furthermore, few researchers have employed multivariable Mendelian randomization (MVMR) frameworks capable of jointly modeling multiple correlated exposures such as smoking, air pollution, or clustered dietary and lifestyle factors to adequately account for confounding and horizontal pleiotropy. In addition, there is a lack of standardization in selecting instrumental variables across studies, with some using genome‐wide significance thresholds of *p* < 5 × 10^−8^, whereas others choose more lenient criteria such as *p* < 1 × 10^−5^ or *p* < 5 × 10^−6^. These inconsistencies may cause variability in instrument strength and heighten the risk of false‐positive findings, which could undermine the comparability and credibility of causal estimates across different studies. Most importantly, the repercussions of sleep issues, dietary factors, metabolic dysfunction, and inactivity are likely to build up over extended time spans. By acting in synergy and interacting with each other, these factors may collectively contribute to the initiation and progression of MS, thus compounding the risk of the disease. Such complexity stresses the requirement for causal inference systems that take into account temporality as well as the multifactorial and interlinked nature of risk factors that can be modified.

The aforementioned limitations pose challenges to conducting randomized controlled trials, and observational studies may not yield adequate evidence of causality. Conversely, MR studies can offer an alternative approach to ascertain causality through the utilization of genome‐wide association study (GWAS) data (Richmond and Smith 2022). MR utilizes instrumental variable analysis to evaluate causal hypotheses with observational data. In this analytical model, genetic variants, with a focus on single nucleotide polymorphisms (SNPs), function as instrumental variables for risk evaluation (Lawlor et al. 2008). The underlying principle of MR reflects Mendel's law of independent assortment, where genetic alleles independently segregate during the formation of gametes, similar to the random assignment in controlled experiments. This approach strives to establish groups with similar characteristics, effectively reducing the impact of confounding factors (Davey Smith and Hemani 2014). This investigation used univariable and multivariable MR approaches to strengthen causal attribution and handle correlated exposures. The genetic instruments adhered to the rigorous genome‐wide significance threshold (*p* < 5 × 10^−8^), thus minimizing the chances of weak instrument bias and false‐positive associations. By integrating a wide range of modifiable exposures and adjusting for confounding within a strong analytical framework, this study reveals new insights into the causal architecture of MS. Consequently, to deal with these challenges, this study executes one of the most comprehensive MR analyses to date, systematically reviewing 29 traits across four domains: dietary factors, metabolic factors, sleep disorders, and physical activity. These traits include snoring, chronotype, sleep duration, short sleep duration, long sleep duration, sleep apnea syndrome, sleeplessness/insomnia, tea intake, beef intake, pork intake, coffee intake, poultry intake, cheese intake, processed meat intake, dried fruit intake, salad/raw vegetable intake, average weekly red wine intake, fish/liver oil dietary supplements, BMI, waist‐to‐hip ratio, hip circumference, waist circumference, essential hypertension, high‐density lipoprotein (HDL) cholesterol levels, low‐density lipoprotein (LDL) cholesterol levels, T2DM, vigorous physical activity, strenuous sports or other exercises, and moderate to vigorous physical activity (MVPA). This approach aims to yield insights for the prevention of MS.

## 2 Materials and Methods

### 2.1 Study Design

**FIGURE 1:**
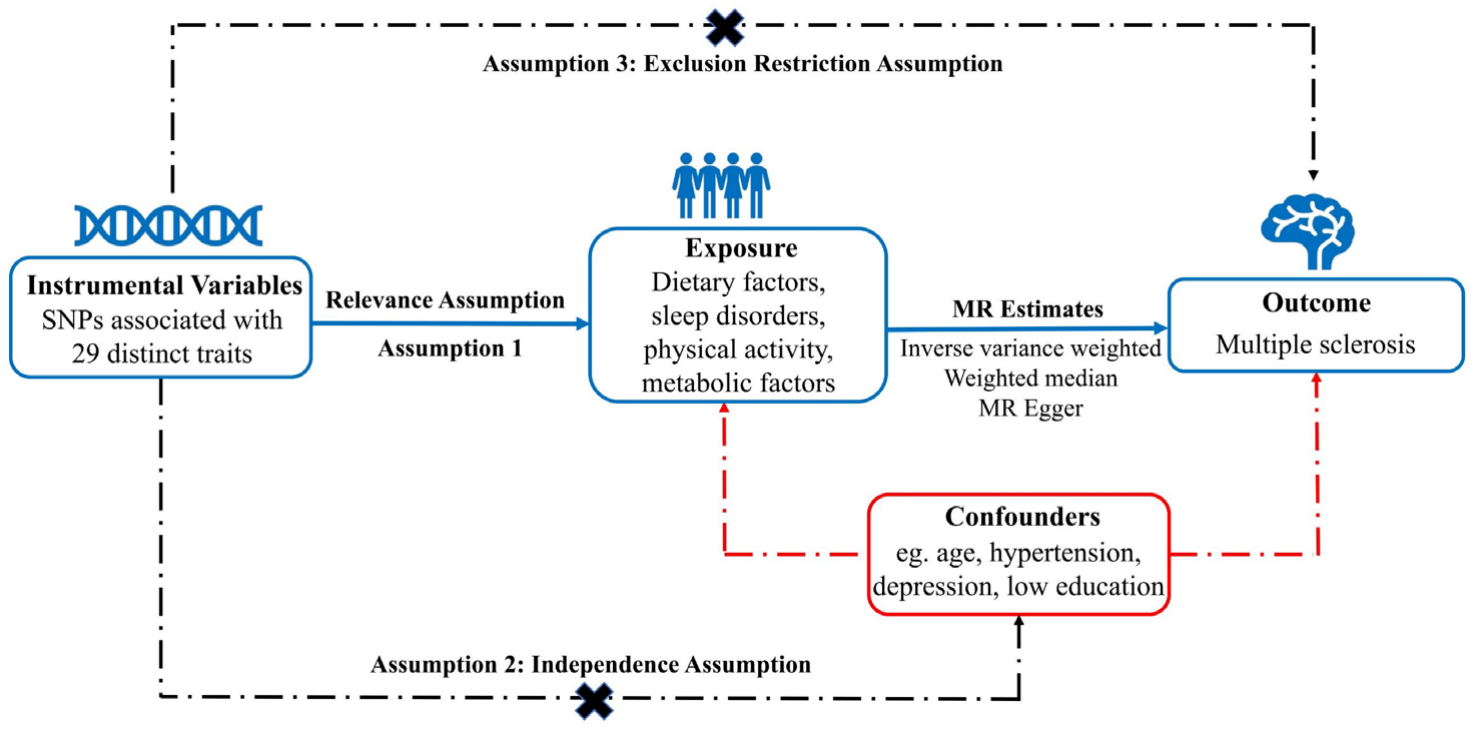
The process of the present Mendelian randomization (MR) analyses is depicted in a flow chart. Assumption 1: The instrumental variables (IVs) selected for this study should exhibit significant associations with sleep disorders, dietary factors, metabolic factors, and physical activity. Assumption 2: The IVs chosen for this study should not show any significant associations with other potential confounding factors. Assumption 3: The IVs employed in this study should not possess any independent causal pathways affecting the outcome (multiple sclerosis) except through sleep disorders, dietary factors, metabolic factors, and physical activity. IV, instrumental variable; MR, Mendelian randomization; SNPs, single nucleotide polymorphisms.

The causal connection between MS risk and 29 unique traits was examined using a two‐sample Mendelian Randomization (2S‐MR) technique. This analysis was grounded in three fundamental assumptions about the genetic variants: (1) they are strongly associated with the exposure to 29 distinct traits; (2) they are not associated with any confounders; and (3) they affect the outcome of MS exclusively through these exposures (Burgess et al. 2015). Figure 1 depicts the 2S‐MR analysis process. Table S1 online provides the Strengthening the Reporting of Observational Studies in Epidemiology Statement (STROBE) checklist for our observational studies.

### 2.2 Data Sources for Exposures

The Integrative Epidemiology Unit's OpenGWAS platform, which compiles more than 126 billion genetic associations from 14,582 datasets, was used to obtain GWAS summary statistics for exposures (Hemani et al. 2018). Exposure information was sourced from the UK Biobank, FinnGen Release, MRC Integrative Epidemiology Unit (MRC‐IEU), and the Genetic Investigation of ANthropometric Traits (GIANT) consortium (Canela‐Xandri et al. 2018). The data sources, consortia, study populations, and sample sizes associated with research on dietary factors, metabolic factors, sleep disorders, and physical activity are detailed in Table 1.

**TABLE 1:** Details of the GWAS included in the two‐sample Mendelian randomization study.

Exposure/Confounder/Outcome	Year	GWAS ID	Consortium	Sample size	Number of SNPs	Population
Dietary factors						
Tea intake	2018	ukb‐b‐6066	MRC‐IEU	447,485	9,851,867	European
Beef intake	2018	ukb‐b‐2862	MRC‐IEU	461,053	9,851,867	European
Pork intake	2018	ukb‐b‐5640	MRC‐IEU	460,162	9,851,867	European
Coffee intake	2018	ukb‐b‐5237	MRC‐IEU	428,860	9,851,867	European
Poultry intake	2018	ukb‐b‐8006	MRC‐IEU	461,900	9,851,867	European
Cheese intake	2018	ukb‐b‐1489	MRC‐IEU	451,486	9,851,867	European
Dried fruit intake	2018	ukb‐b‐16576	MRC‐IEU	421,764	9,851,867	European
Processed meat intake	2018	ukb‐b‐6324	MRC‐IEU	461,981	9,851,867	European
Salad/raw vegetable intake	2018	ukb‐b‐1996	MRC‐IEU	435,435	9,851,867	European
Average weekly red wine intake	2018	ukb‐b‐5239	MRC‐IEU	327,026	9,851,867	European
Fish/liver oil dietary supplements	2018	ukb‐b‐11,075	MRC‐IEU	461,384	9,851,867	European
Metabolic factor						
Body mass index	2015	ieu‐a‐835	GIANT	322,154	2,554,668	European
Waist‐to‐hip ratio	2015	ieu‐a‐73	GIANT	212,244	2,560,782	European
Hip circumference	2015	ieu‐a‐49	GIANT	213,038	2,559,739	European
Waist circumference	2015	ieu‐a‐67	GIANT	231,353	2,546,074	European
Essential hypertension	2018	finn‐b‐I9_HYPTENSESS	FinnGen	205,694	16,380,443	European
HDL cholesterol levels	2022	ebi‐a‐GCST90092822	UK Biobank	115,082	11,590,399	European
LDL cholesterol levels	2022	ebi‐a‐GCST90092883	UK Biobank	115,082	11,590,399	European
Type 2 diabetes mellitus		finn‐b‐E4_DM2_STRICT	FinnGen	212,351	16,380,434	European
Sleep trait						
Snoring	2018	ukb‐b‐17400	MRC‐IEU	430,438	9,851,867	European
Chronotype	2016	ieu‐a‐1087	UK Biobank	128,266	17,032,431	European
Sleep duration	2018	ukb‐b‐4424	MRC‐IEU	460,099	9,851,867	European
Short sleep duration	2016	ebi‐a‐GCST006686	UK Biobank	110,188	16,561,726	European
Long sleep duration	2016	ebi‐a‐GCST006685	UK Biobank	91,306	16,563,303	European
Sleep apnea syndrome	2021	ebi‐a‐GCST90018916	UK Biobank	476,853	24,183,940	European
Sleeplessness/insomnia	2018	ukb‐b‐3957	MRC‐IEU	462,341	9,851,867	European
Physical activity						
Vigorous physical activity	2018	ebi‐a‐GCST006098	UK Biobank	377,234	11,808,007	European
Strenuous sports or other exercises	2018	ebi‐a‐GCST006100	UK Biobank	350,492	11,807,536	European
Moderate to vigorous physical activity	2018	ebi‐a‐GCST006097	UK Biobank	377,234	11,808,007	European
Confounder						
Past tobacco smoking	2017	ukb‐a‐17	UK Biobank	310,749	10,894,596	European
Particulate matter air pollution (PM2.5)	2018	ukb‐b‐10817	MRC‐IEU	423,796	9,851,867	European
Outcome						
Multiple sclerosis	2019	ieu‐b‐18	IMSGC	115,803	6,304,359	European

Abbreviations: GIANT, Genetic Investigation of Anthropometric Traits; GWAS, Genome‐Wide Association Study; HDL, High‐density lipoprotein; IMSGC, International Multiple Sclerosis Genetics Consortium; LDL, Low‐density lipoprotein; MRC‐IEU, Medical Research Council Integrated Epidemiology Unit; SNPs, Single nucleotide polymorphisms.

Four consortia aggregated data from participants of European ancestry on 29 distinct traits, encompassing a wide range of characteristics: snoring (*n* = 430,438), chronotype (*n* = 128,266), various sleep durations including general (*n* = 460,099), short (*n* = 110,188), and long (*n* = 91,306), sleep apnea syndrome (*n* = 476,853), sleeplessness/insomnia (*n* = 462,341), dietary intakes such as tea (*n* = 447,485), beef (*n* = 461,053), pork (*n* = 460,162), coffee (*n* = 428,860), poultry (*n* = 461,900), cheese (*n* = 451,486), processed meats (*n* = 461,981), dried fruits (*n* = 421,764), salads/raw vegetables (*n* = 460,162), and average weekly red wine (*n* = 327,026), along with fish/liver oil dietary supplements (*n* = 461,384). Additionally, measurements included BMI (*n* = 322,154), waist‐to‐hip ratio (*n* = 212,244), hip circumference (*n* = 213,038), waist circumference (*n* = 231,353), essential hypertension (*n* = 205,694), HDL and LDL cholesterol levels (*n* = 115,082 each), T2DM (*n* = 212,351), and physical activities like vigorous exercise (*n* = 377,234), strenuous sports or other exercises (*n* = 350,492), and MVPA (*n* = 377,234).

### 2.3 Data Source for Outcome

The International Multiple Sclerosis Genetics Consortium GWAS supplied outcome data, including 47,429 MS cases and 68,374 controls of European ancestry, with these summary statistics being publicly accessible via OpenGWAS.

### 2.4 Data Sources for Confounders

We identified two common factors as potential confounders in the relationship between exposure and outcome. Consistent findings from randomized controlled trials, systematic reviews, and epidemiological research suggest that smokers have an increased risk of developing MS and enduring its harmful symptoms and complications (Arneth 2020; Nielsen et al. 2024). Although the role of air pollution in the pathogenesis of MS is not fully understood, evidence from the included studies suggests that exposure to polluted air may activate several mechanisms contributing to the development of MS, as well as to episodes of disease relapse or neurological disability (Hedström et al. 2023; Noorimotlagh et al. 2021). Data on past tobacco smoking were obtained from a meta‐analysis conducted by the UK Biobank, involving 310,749 individuals. Summarized data from the MRC‐IEU, available to the public, involved 423,796 European individuals in relation to genetic associations with matter air pollution 2.5 (PM2.5). The data sources are described in Table 1.

### 2.5 Selection of Instrumental Variables

Rigorous criteria were employed to identify relevant SNPs as genetic instruments. Genome‐wide SNPs associated with 29 distinct traits were identified, each meeting a significance threshold of *p* < 5 × 10^−8^. For conditions such as sleep apnea syndrome and fish/liver oil dietary supplements, which were linked to fewer than four selected SNPs, the significance threshold was adjusted to *p* < 5 × 10^−7^ to ensure a sufficient recruitment of instrumental variables (IVs). Each SNP required independent association with the microbiota, adhering to a clumping distance of 10,000 kilobases and a linkage disequilibrium threshold of *r* ^2^ less than 0.001 (Zha et al. 2021). The overall *F*‐statistic (*F* = beta^2^/se^2^) was computed to verify that each SNP possessed adequate statistical power, setting a minimum *F*‐statistic of 10 for robust analysis (Rosa et al. 2019). Table S2 details summary data for SNPs linked to 29 distinct traits and their correlation with MS.

### 2.6 MR Analysis and Sensitivity Analysis

To begin with, the 2S‐MR analysis employed the Inverse‐Variance Weighted (IVW) method to study the causal associations between 29 distinct traits and the risk of MS (Burgess et al. 2017). The study also conducted MR‐Egger (MRE), Simple Mode (SMod), Weighted Median (WMed), Weighted Mode (WMod), and MR‐Pleiotropy RESidual Sum and Outlier (MR‐PRESSO) analyses. The WMed approach produces significant causal estimates provided that at least 50% of the instrumental weight is derived from validated SNPs (Bowden et al. 2016). The MRE technique identifies and adjusts for potential pleiotropy in the estimates (Bowden et al. 2015). The MR‐PRESSO serves to pinpoint and exclude outliers before recalculating the causal estimates (Verbanck et al. 2018). Using Cochrane's *Q* to evaluate heterogeneity, a *p*‐value of less than 0.05 suggested horizontal pleiotropy (Greco et al. 2015). Directional pleiotropy was assessed using the intercept *p*‐value from the MRE analysis (Bowden et al. 2015). The analysis using the leave‐one‐out method demonstrated the findings' sensitivity and reliability. Statistical analyses utilized R version 4.3.3 along with the 2S‐MR package.

### 2.7 MVMR Analysis

To strengthen the robustness of our findings from the univariable MR analysis, we conducted an MVMR analysis focusing on eight exposures, namely, chronotype, pork intake, fish/liver oil dietary supplements, BMI, T2DM, hip circumference, short sleep duration, and MVPA, which showed significant causal associations with MS in the primary analysis and were supported by prior biological evidence. To mitigate potential confounding, past tobacco smoking and PM2.5, both established or suspected MS risk factors, were included as covariates. In the MVMR framework, causal effect estimates were derived using the IVW estimator, and statistical significance was defined as *p* < 0.05.

## 3 Results

### 3.1 Identification of IVs for MR Analysis

Table 1 provides the source of the GWAS data. Details of the SNPs for 29 various traits and their *F*‐statistics can be found in Table S2. Specifically, the following numbers of SNPs were identified: 43 for snoring, 12 for chronotype, 71 for sleep duration, 15 for short sleep duration, 23 for long sleep duration, 38 for sleep apnea syndrome, 42 for sleeplessness/insomnia, 40 for tea intake, 8 for beef intake, 14 for pork intake, 40 for coffee intake, 8 for poultry intake, 65 for cheese intake, 23 for processed meat intake, 43 for dried fruit intake, 22 for salad/raw vegetable intake, 19 for average weekly red wine intake, 40 for fish/liver oil dietary supplements, 69 for BMI, 29 for waist‐to‐hip ratio, 52 for hip circumference, 22 for waist circumference, 40 for essential hypertension, 91 for HDL cholesterol levels, 48 for LDL cholesterol levels, 34 for T2DM, 7 for vigorous physical activity, 14 for strenuous sports or other exercises, and 19 for MVPA. All 1064 SNPs demonstrated *F*‐statistics exceeding the threshold of 20, indicating robust predictions of the sleep disorders, dietary factors, metabolic factors, and physical activity in the MR analysis.

### 3.2 Causal Influence of Sleep Disorders, Dietary Factors, Metabolic Factors, and Physical Activity on MS

**FIGURE 2:**
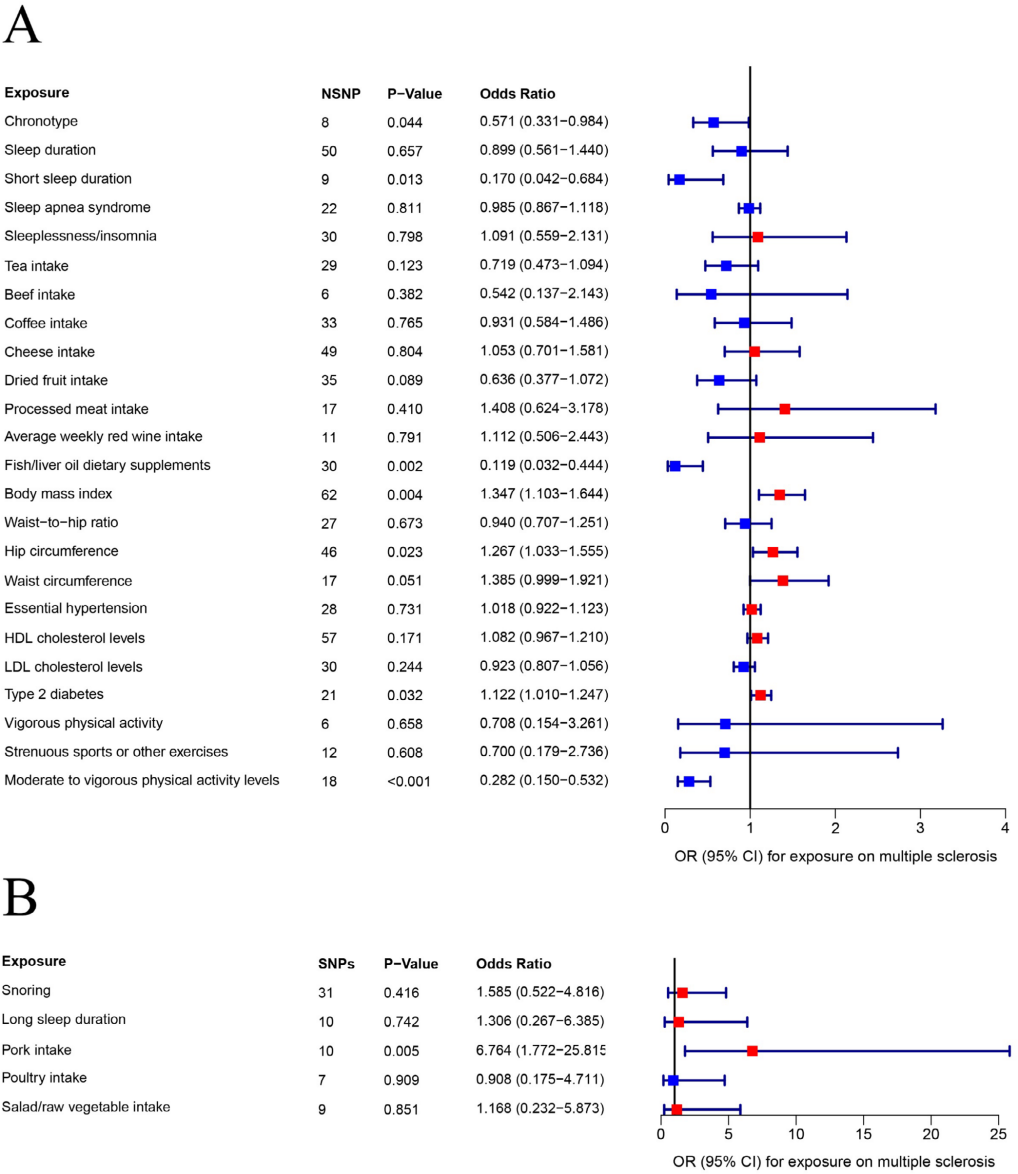
A, B Forest plots illustrate the estimated effects of exposure on multiple sclerosis in MR analysis. Estimates were obtained using the inverse‐variance weighted method. CI, confidence interval; MR, Mendelian randomization; SNPs, single nucleotide polymorphisms.

**FIGURE 3:**
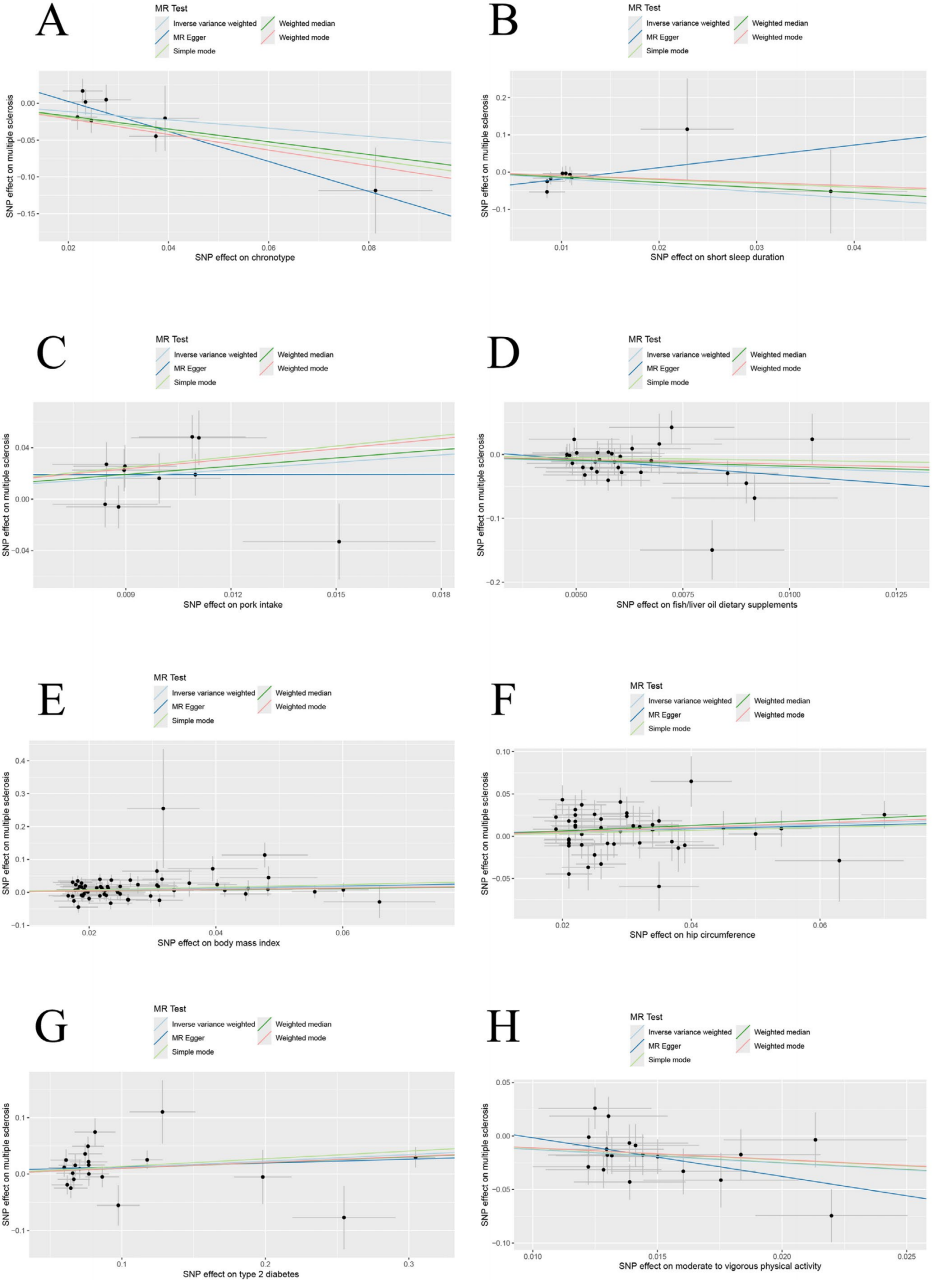
Scatter plots illustrate the causality analysis of exposure to multiple sclerosis. Chronotype (A), short sleep duration (B), pork intake (C), fish/liver oil dietary supplements (D), body mass index (E), hip circumference (F), type 2 diabetes (G), moderate to vigorous physical activity (H) as exposure, and multiple sclerosis as outcome. MR, Mendelian randomization; SNP, single nucleotide polymorphism.

A 2S‐MR analysis was performed to explore the causal associations among 29 traits linked to sleep disorders, dietary habits, metabolic factors, physical activity, and the risk of MS. The estimated effects of exposure on multiple sclerosis are illustrated by forest plots in MR analysis (Figure 2A,B). Estimates were obtained using the IVW method. The IVW method indicated that certain factors significantly increased the risk of MS: pork intake (OR = 6.764; 95% CI, 1.772–25.815; *p* = 0.005), BMI (OR = 1.347; 95% CI, 1.103–1.644; *p* = 0.004), T2DM (OR = 1.122; 95% CI, 1.010–1.247; *p* = 0.032), and hip circumference (OR = 1.267; 95% CI, 1.033–1.555; *p* = 0.023). Conversely, chronotype (OR = 0.571; 95% CI, 0.331–0.984; *p* = 0.044), short sleep duration (OR = 0.170; 95% CI, 0.042–0.684; *p* = 0.013), fish/liver oil dietary supplements (OR = 0.119; 95% CI, 0.032–0.444; *p* = 0.002), and MVPA (OR = 0.282; 95% CI, 0.150–0.532; *p* < 0.001) were associated with a significant decrease in MS risk. Scatter plots illustrate the causality analysis of chronotype, pork intake, fish/liver oil dietary supplements, BMI, T2DM, hip circumference, short sleep duration, and MVPA on multiple sclerosis in MR analysis (Figure 3). Figure S1, displaying scatter plots from five different methods, shows no significant causal links between migraine and the remaining factors: snoring, sleep duration, long sleep duration, sleep apnea syndrome, sleeplessness/insomnia, tea intake, beef intake, coffee intake, poultry intake, cheese intake, processed meat intake, dried fruit intake, salad/raw vegetable intake, average weekly red wine intake, waist‐to‐hip ratio, hip circumference, waist circumference, essential hypertension, HDL cholesterol levels, LDL cholesterol levels, T2DM, vigorous physical activity, strenuous sports or other exercises, and MVPA. The MRE, SMod, WMed, and WMod analyses produced causal effect estimates that matched the IVW method in terms of magnitude and direction (Table S3).

### 3.3 Heterogeneity, Pleiotropy, and Sensitivity Analysis

We assessed heterogeneity using Cochran's *Q* statistics through both the IVW and MRE methods, as detailed in Table 2. Notable heterogeneity was detected in the analysis of cereal intake (*p* = 0.021, *Q* = 69.832), LDL cholesterol levels (*p* = 0.010, *Q* = 57.662), T2DM (*p* = 0.020, *Q* = 35.081), and processed meat intake (*p* = 0.025, *Q* = 28.794). In reaction, a multiplicative random‐effects model was utilized within the IVW framework for MR analysis. According to the MR‐PRESSO and MRE intercept analyses, there was no significant directional horizontal pleiotropy (Table 2). The reliability of our two‐sample MR findings was affirmed by sensitivity analyses, including Cochran's *Q* statistics, MRE intercept, MR‐PRESSO global test, and leave‐one‐out analysis. No individual instrumental variable was found to significantly affect the results, as further demonstrated by the leave‐one‐out analysis (Figure S2).

**TABLE 2:** The heterogeneity and pleiotropy analysis in the two‐sample Mendelian randomization study.

Exposure	Outcome	Method	Heterogeneity		Horizontal pleiotropy		MR PRESSO	
			Q	Q_p‐value	MRE intercept	*p*	Outliers	*p*
Tea intake	Multiple sclerosis	MRE	34.032	0.165	−0.015	0.147	0	0.127
		IVW	36.839	0.122				
Beef intake	Multiple sclerosis	MRE	4.683	0.321	0.081	0.254	0	0.164
		IVW	6.752	0.240				
Pork intake	Multiple sclerosis	MRE	12.375	0.135	0.019	0.703	0	0.080
		IVW	12.616	0.181				
Coffee intake	Multiple sclerosis	MRE	35.172	0.277	−0.011	0.177	0	0.063
		IVW	37.339	0.237				
Poultry intake	Multiple sclerosis	MRE	10.380	0.065	0.205	0.501	0	0.084
		IVW	11.470	0.075				
Cheese intake	Multiple sclerosis	MRE	67.281	0.028	0.020	0.188	0	0.051
		IVW	69.832	0.021				
Processed meat intake	Multiple sclerosis	MRE	28.117	0.021	−0.023	0.557	0	0.063
		IVW	28.794	0.025				
Dried fruit intake	Multiple sclerosis	MRE	32.456	0.494	0.010	0.513	1	0.604
		IVW	32.893	0.522				
Salad/raw vegetable intake	Multiple sclerosis	MRE	9.673	0.208	0.071	0.214	3	0.089
		IVW	12.259	0.140				
Average weekly red wine intake	Multiple sclerosis	MRE	9.691	0.376	0.022	0.269	0	0.432
		IVW	11.182	0.344				
Fish/liver oil dietary supplements	Multiple sclerosis	MRE	31.700	0.287	0.018	0.411	0	0.116
		IVW	32.487	0.299				
Body mass index	Multiple sclerosis	MRE	80.878	0.038	−0.001	0.884	0	0.061
		IVW	80.907	0.045				
Waist‐to‐hip ratio	Multiple sclerosis	MRE	25.100	0.457	−0.030	0.068	0	0.353
		IVW	28.751	0.323				
Hip circumference	Multiple sclerosis	MRE	59.775	0.057	0.002	0.794	3	0.084
		IVW	59.869	0.068				
Waist circumference	Multiple sclerosis	MRE	17.473	0.291	0.015	0.298	3	0.414
		IVW	18.827	0.278				
Essential hypertension	Multiple sclerosis	MRE	24.952	0.522	0.023	0.166	0	0.440
		IVW	26.981	0.465				
HDL cholesterol levels	Multiple sclerosis	MRE	71.825	0.063	0.003	0.586	4	0.142
		IVW	72.217	0.071				
LDL cholesterol levels	Multiple sclerosis	MRE	53.674	0.020	0.010	0.160	6	0.053
		IVW	57.662	0.010				
Type 2 diabetes mellitus	Multiple sclerosis	MRE	34.531	0.016	0.006	0.589	2	0.063
		IVW	35.081	0.020				
Snoring	Multiple sclerosis	MRE	37.617	0.131	−0.033	0.212	3	0.054
		IVW	39.727	0.110				
Chronotype	Multiple sclerosis	MRE	4.843	0.564	0.043	0.116	0	0.155
		IVW	8.214	0.314				
Sleep duration	Multiple sclerosis	MRE	56.198	0.195	0.010	0.353	0	0.288
		IVW	57.227	0.196				
Short sleep duration	Multiple sclerosis	MRE	6.308	0.504	−0.049	0.178	0	0.295
		IVW	8.553	0.381				
Long sleep duration	Multiple sclerosis	MRE	1.959	0.982	0.005	0.806	0	0.992
		IVW	2.024	0.991				
Sleep apnea syndrome	Multiple sclerosis	MRE	27.812	0.114	−0.018	0.390	0	0.317
		IVW	28.884	0.117				
Sleeplessness/insomnia	Multiple sclerosis	MRE	34.705	0.179	0.010	0.426	0	0.057
		IVW	35.512	0.188				
Vigorous physical activity	Multiple sclerosis	MRE	3.649	0.456	−0.010	0.880	0	0.646
		IVW	3.675	0.597				
Strenuous sports or other exercises	Multiple sclerosis	MRE	10.715	0.380	0.018	0.584	0	0.535
		IVW	11.058	0.438				
Moderate to vigorous physical activity	Multiple sclerosis	MRE	17.207	0.372	0.034	0.234	0	0.383
		IVW	18.849	0.337				

Abbreviations: HDL, High‐density lipoprotein; IVW, Inverse Variance Weighted; LDL, Low‐density lipoprotein; MR, Mendelian randomization; MRE, MR Egger; Q, Cochran's *Q* test.

### 3.4 MVMR Analysis

**FIGURE 4:**
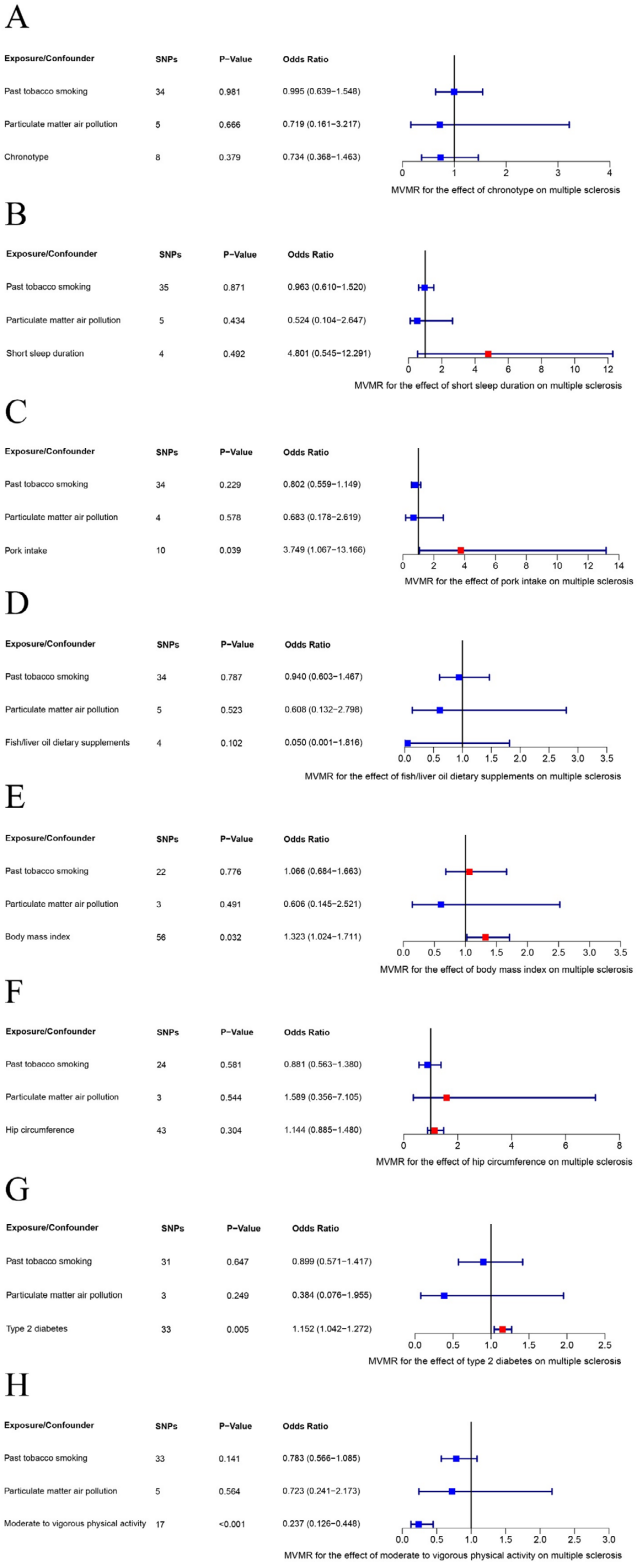
Forest plots illustrate the estimated effects of chronotype (A), short sleep duration (B), pork intake (C), fish/liver oil dietary supplements (D), body mass index (E), hip circumference (F), type 2 diabetes (G), moderate to vigorous physical activity (H) on multiple sclerosis in MVWR after adjusting for potential confounders. Estimates were obtained using the inverse‐variance weighted method.CI, confidence interval; MVWR, multivariable Mendelian randomization; SNPs, single nucleotide polymorphisms.

To further explore the causal effects of chronotype, pork intake, fish/liver oil dietary supplements, BMI, T2DM, hip circumference, short sleep duration, and MVPA on MS, we conducted a MVMR analysis, adjusting for past tobacco smoking and PM2.5. The results from the MVMR analysis suggested that the connections between pork intake, BMI, T2DM, and MVPA with MS risk stayed statistically significant, with effect estimates largely matching in magnitude and direction with the univariable MR outcome. In contrast, following these adjustments, the initially observed causal associations between fish/liver oil dietary supplements, hip circumference, short sleep duration, and chronotype with MS did not remain statistically significant. Forest plots represent the estimated effects of chronotype, short sleep duration, pork intake, fish/liver oil dietary supplements, BMI, hip circumference, T2DM, and moderate to vigorous physical activity on multiple sclerosis in MVWR following adjustment (Figure 4 and Table S4).

## 4 Discussion

The precise cause of MS remains elusive; however, epidemiological and association studies suggest that a complex interplay between environmental and lifestyle factors, such as smoking, body mass, nutrition, sunlight exposure, and vitamin D levels, and genetic susceptibilities influences the pathological processes (Bjornevik et al. 2022). These processes may be activated by viral infections, notably the Epstein–Barr virus (Lanz et al. 2022; Bjornevik et al. 2022). It is now acknowledged that environmental factors interact with genetic predispositions to influence susceptibility to MS as well as the disease's progression. Crucially, several of these environmental factors are modifiable (Alfredsson and Olsson 2019). Identifying the causes and risk factors of MS and substantiating the effectiveness of certain interventions are both crucial and feasible. This research could lead to advanced preventive strategies for patients, potentially mitigating the onset and progression. Research has demonstrated associations between sleep disorders, dietary factors, metabolic factors, and physical activity with the onset and progression of MS in some studies. However, the causal relationships among these factors have not yet been established. Should these causal links be confirmed, interventions targeting daily life and mental health could potentially serve as effective strategies to reduce both the incidence and severity of MS.

In this study, 29 traits encompassing dietary factors, metabolic factors, sleep disorders, and physical activity were investigated to explore their potential causal associations with MS. These traits influence our bodies in subtle and gradual ways, making it challenging to establish causality through traditional randomized controlled trials (RCTs). Consequently, MR analysis may offer a viable alternative approach. In our initial findings, variables such as chronotype, short sleep duration, pork intake, fish/liver oil dietary supplements, BMI, T2DM, hip circumference, and MVPA exhibited potential causal links with MS. To mitigate statistical bias arising from multiple testing, MVMR analysis was performed, incorporating adjustments for potential confounding variables. Subsequently, significant and causal relationships with MS were identified for several factors: pork intake, BMI, T2DM, and MVPA.

The relationship between dietary patterns and MS has been a prominent focus within epidemiological studies. The relationship between red meat intake and MS has been the subject of considerable debate. The immune system operates at its best when supported by a balanced diet, and indications exist that dietary factors contribute to the onset of inflammatory diseases (Christ et al. 2019). Asgharzadeh V. et al. found that consuming fruits regularly until the age of 15 may help reduce the risk of MS in later stages of life. In contrast, frequent consumption of high‐fat dairy, fast foods, sausages, soybean products, kielbasa, pickles, sweets, meat, sauces, and fizzy drinks before the age of 15 may affect the health and risks of MS adversely (Asgharzadeh et al. 2023). Red meat is a nutrient‐dense source of protein fundamental for everyone, which includes beef, pork, or lamb. A study involving 102 adult MS patients found that reduced consumption of red meat may help attenuate MS symptoms (Ertaş Öztürk et al. 2023). Moreover, a separate case–control investigation into environmental risk factors for CNS demyelination in Australia similarly found that supplementing a Mediterranean diet with unprocessed red meat might offer advantages to individuals at elevated risk of MS (Black et al. 2019). However, recent research provided no substantial evidence to support the hypothesis that high fiber intake and low meat consumption significantly affect the risk of developing MS (Rubin et al. 2020). Prior research using Mendelian randomization has found a strong positive correlation between genetically anticipated pork intake and the risk of multiple sclerosis, but these analyses did not adjust for possible lifestyle‐related confounding (Nan 2024; Ye et al. 2025). Complementary to the genetic evidence, longitudinal cohort studies have regularly demonstrated that meat consumption is related to greater disability progression in those with MS. During a 2.5‐year follow‐up of the HOLISM cohort, initial meat consumption was correlated with an 86% higher chance of disability worsening (Taylor et al. 2018). A subsequent 7.5‐year analysis of the same cohort confirmed this association, reporting a twofold increased risk of disability progression and greater cumulative disability among meat consumers, although part of this effect was explained by overall diet quality (Simpson‐Yap et al. 2023). By employing MVMR and adjusting for relevant confounders in our study, we not only validate these observational findings but also reinforce the evidence for a potential causal association between pork consumption and MS risk. Several factors are involved in the pathophysiological connections between red meat and MS. Western‐style diets, characterized by high red meat consumption and other factors like excessive salt, animal fats, sugary drinks, fried foods, low fiber intake, and a sedentary lifestyle, are known to elevate inflammation levels. These diets enhance the metabolism of human cells, directing them toward biosynthetic pathways, including those that produce pro‐inflammatory molecules (Riccio and Rossano 2015). They also contribute to an unhealthy gut microbiota, altered intestinal immune responses, and chronic low‐grade inflammation (Riccio and Rossano 2015). Norartocarpetin, identified as a flavonoid, effectively inhibits 2‐Amino‐1‐methyl‐6‐phenylimidazo[4,5‐b]pyridine formation by trapping phenylacetaldehyde and forming adducts in roast beef patties (Zheng et al. 2016). Moreover, the effects of clenbuterol and salbutamol on the metabolism of pork meat illustrate the complex metabolic processes related to red meat (Lu et al. 2017). By potentially altering the body's metabolic and immune responses, these processes may have implications for the pathophysiology of MS, which is involved in its development and progression.

BMI has been demonstrated to be associated with the risk of MS in numerous population studies worldwide. Obesity is recognized as a risk factor for a wide range of metabolic, inflammatory, and autoimmune diseases, influencing their incidence, severity, and patient outcomes (Gremese et al. 2014). A systematic review and wide‐angled MR study to examine that both childhood and adulthood BMI were positively associated with MS in univariate UVMR (Yuan et al. 2021). Furthermore, another study identified interleukin‐6 signaling as a major mediator in the association between BMI and the risk of MS, as demonstrated through MR analyses (Vandebergh, Degryse, et al. 2022). In our study, significant causal relationships between BMI and MS were established through MVMR analyses that adjusted for confounding factors such as past tobacco smoking and PM2.5. The evidence aligns with mechanistic findings that a higher BMI can modify ceramide signaling, induce changes in DNA methylation, and thus influence the clinical development of MS (Castro et al. 2019). Moreover, research involving longitudinal neuroimaging has revealed an association between BMI and progressive changes in brain volume in patients with MS (Mowry et al. 2018). Taken together, these complementary genetic, mechanistic, and longitudinal observations strengthen the case for a robust causal relationship between BMI and MS. Inflammation and oxidative stress might be a mechanism for BMI‐related neurodegeneration in MS. Increased production of reactive oxygen species (ROS) can be a consequence of the chronic low‐grade inflammation associated with obesity. Neurons and myelin can be damaged by ROS, which contributes to neurodegenerative processes (Oliveira et al. 2017). In research involving MS patients, higher levels of oxidative stress markers were noted, potentially worsened by inflammation linked to obesity (Oliveira et al. 2017). In addition, adipokines such as leptin and resistin, which are released by adipose tissue and might be affected by BMI, have the ability to traverse the blood–brain barrier and impact neuronal functions. Leptin has been observed to have both beneficial and detrimental effects on neurons, contingent on the context, and its imbalance in obesity may be linked to neurodegenerative processes in MS (Daryabor et al. 2022). Moreover, in patients with relapsing–remitting MS, obesity has been associated with an amplified central inflammatory response, as evidenced by elevated cerebrospinal fluid concentrations of the pro‐inflammatory cytokine interleukin‐6 (IL‐6) and diminished levels of the anti‐inflammatory cytokine interleukin‐13 (IL‐13) (Stampanoni Bassi et al. 2020).

T2DM has traditionally been characterized as a metabolic disorder, marked by chronic hyperglycemia, insulin resistance, and a progressively diminishing insulin secretion from the pancreas. T2DM has also been identified as a risk factor for MS: over 9 years of follow‐up, a comprehensive study conducted within a large Chinese cohort, comprising 614,623 patients with T2DM and 614,021 controls, revealed a moderate yet significant correlation between T2DM and the incidence of newly diagnosed MS. Notably, this correlation was maintained across varying sociodemographic profiles and irrespective of specific MS‐related comorbidities (Hou et al. 2017). Numerous studies have suggested that MS may increase the risk of developing T2DM; however, investigations into the risk factors associated with MS have rarely considered T2DM (Cho et al. 2024; Brnabic et al. 2024) T2DM may exert its impact by affecting other risk factors, such as dietary habits and obesity (Morales‐Suarez‐Varela et al. 2021). Our analyses revealed a significant causal link between T2DM and MS, filling an important gap in the literature regarding the metabolic factors influencing MS risk. This genetic evidence complements longitudinal clinical findings showing that T2DM is associated with accelerated disability progression and greater overall disability burden in MS patients, as well as mechanistic data implicating impaired glycemic control, chronic inflammation, and oxidative stress in MS pathogenesis (Peng et al. 2025; Conway et al. 2017). These converging lines of evidence collectively highlight the potential significance of effective glycemic control in mitigating MS risk and informing preventive and therapeutic strategies. Both T2DM and MS are significantly influenced by autoimmune mechanisms, although the exact targets and processes differ. Even though T2DM is not often considered a conventional autoimmune disease like MS, there is evidence of autoimmune‐related processes. One study, for example, explored antibodies to post‐translationally modified insulin in T2DM. In T2DM patients, reactive oxidants connected to islet inflammation cause oxidative post‐translational modification of insulin, resulting in neoepitopes that elicit an immune response (Strollo et al. 2015). The association between T2DM, insulin resistance, and autoimmune‐related diseases (ARDs) was explored through a Mendelian randomization study (Strollo et al. 2015). The findings also revealed that immune cells are linked to T2DM and serve as a marker for insulin resistance, implying that dysregulation of autoimmune‐related immune cells could be involved in the relationship between T2DM and ARDs like MS (Peng et al. 2025).

Our study included three types of physical activities, which were analyzed to determine their causal associations with MS. Significant evidence emerged from this analysis, indicating a notable link between exercise and MS. Furthermore, population studies have consistently demonstrated that physical activity acts as a protective factor against MS (Motl 2020). A meta‐analysis of 45 randomized clinical trials indicated that aerobic and mind–body exercises significantly improve both the physical and mental health‐related quality of life in individuals with MS (Reina‐Gutiérrez et al. 2022). Physical activity can play a variety of beneficial roles in the body, particularly in enhancing muscle strength and reducing fatigue. Recently, new research has suggested that physical activity may restrict the infiltration of peripheral immune cells into the CNS parenchyma, curb the hyperactivation of these cells, and facilitate a shift in the balance of immune cells from a pro‐inflammatory to an anti‐inflammatory state (Zong et al. 2023). Evidence from our study indicates that engaging more in MVPA may offer a significant defense against the onset of MS. In individuals with relapsing–remitting MS, longitudinal studies using accelerometers have found that higher MVPA levels are related to better functional outcomes, improved sleep quality, and a decrease in common MS symptoms such as fatigue and depression (Macdonald et al. 2023). Moreover, patterns of MVPA over time suggest that tailored behavioral interventions, particularly those targeting individuals with lower baseline activity and higher disability, may sustain or enhance physical activity levels and thereby amplify potential neuroprotective and systemic benefits (Klaren et al. 2017). The integration of genetic, epidemiological, and behavioral findings points to MVPA as a significant lifestyle factor that can be adjusted to aid in MS prevention and management. In the context of MS, neuroinflammation is a prominent feature, and physical activity has been shown to have immune‐modulating effects that may help in reducing this inflammation. In the realm of MS, exercise has been shown to engage innate signaling mechanisms, particularly toll‐like receptors (TLRs), which direct the innate immune response (Barry et al. 2016). Moreover, exercise has been demonstrated to improve chronic neuroinflammation and its associated conditions by shifting cytokine profiles towards an anti‐inflammatory pattern (Barry et al. 2016). The effects of exercise on neuroinflammation in the hypothalamus and hippocampus were studied, showing that exercise reduced fat mass and leptin levels in the blood for both adolescents and adults (Soch et al. 2016).

In this study, stringent SNP filtration steps were implemented to minimize bias due to horizontal pleiotropy and to adhere to the three core assumptions of MR. However, there are several strengths and limitations of this study. The main advantage lies in employing the 2S‐MR approach, utilizing genetic variation as IVs to establish causality, thereby reducing biases stemming from reverse causality and confounding variables. By conducting sensitivity and pleiotropic analyses and utilizing data from diverse European populations, we ensured the accuracy of our MR analysis. However, acknowledging limitations is crucial. First, even though most IVs were strongly associated with exposure, some had F‐statistics below 100, which could potentially impact the accuracy of the results. Second, we were unable to distinguish between different types of dietary intake or evaluate the impact of particular dietary combinations. Third, the lack of sex‐stratified GWAS data prevented sex‐specific analyses. Fourth, the MR instruments in this study were based on dietary categories or specific food items (e.g., pork intake and total fruit intake) rather than single nutrients such as heme iron or eicosapentaenoic acid/docosahexaenoic acid. This reflects the limited availability of robust nutrient‐specific GWAS instruments, which restricts our ability to attribute observed associations to individual nutrients.

## 5 Conclusion

In conclusion, this study established potential causal relationships between dietary factors, metabolic factors, sleep disorders, physical activity, and MS. Specifically, increased engagement in MVPA was associated with a reduced risk of MS, whereas higher BMI, greater pork intake, and a history of T2DM were linked to an increased risk. No significant associations were found with the other remaining factors. These findings lay the groundwork for developing targeted nutritional interventions for MS patients.

## Author Contributions

**Hongwei Liu:** data curation (equal), formal analysis (equal), funding acquisition (equal), investigation (equal), methodology (equal), project administration (equal), resources (equal), software (equal), supervision (equal), validation (equal), visualization (equal), writing – original draft (equal), writing – review and editing (equal). **Minghui Wu:** conceptualization (equal), data curation (equal), formal analysis (equal), funding acquisition (equal), investigation (equal), methodology (equal), project administration (equal), resources (equal), software (equal), supervision (equal), validation (equal), visualization (equal), writing – original draft (equal), writing – review and editing (equal). **Jie Yang:** conceptualization (equal), data curation (equal), formal analysis (equal), funding acquisition (equal), investigation (equal), methodology (equal), project administration (equal), resources (equal), software (equal), supervision (equal), validation (equal), visualization (equal), writing – original draft (equal), writing – review and editing (equal). **Minheng Zhang:** data curation (equal), funding acquisition (equal), methodology (equal), software (equal), validation (equal), writing – original draft (equal). **Xuan Chen:** conceptualization (equal), formal analysis (equal), investigation (equal), project administration (equal), software (equal), validation (equal), writing – original draft (equal). **Haixia Fan:** conceptualization (equal), data curation (equal), formal analysis (equal), funding acquisition (equal), investigation (equal), methodology (equal), project administration (equal), resources (equal), software (equal), supervision (equal), validation (equal), visualization (equal), writing – original draft (equal), writing – review and editing (equal).

## Consent

The authors have nothing to report.

## Conflicts of Interest

The authors declare no conflicts of interest.

## Supporting information


**Figures S1–S2:** fsn370905‐sup‐0001‐FiguresS1‐S2.docx.

**Tables S1–S4:** fsn370905‐sup‐0002‐TablesS1‐S4.docx.

## Data Availability

Details of the original contributions from this study can be found in the article and its Supporting Information. For more inquiries, please get in touch with the corresponding author.
