# Supplementary material for: Associations Between Dietary Factors, Metabolic Factors, Sleep Disorders, Physical Activity, and the Risk of Multiple Sclerosis: A Univariable and Multivariable Mendelian Randomization Study
Source: Food Sci Nutr. 2025 Sep 11;13(9):e70905. doi: 10.1002/fsn3.70905 (PMC12424063; doi:10.1002/fsn3.70905)
Supplement: Supplementary file 1 — Figures S1–S2: fsn370905‐sup‐0001‐FiguresS1‐S2.docx. [file FSN3-13-e70905-s001.docx]

**Associations between** **dietary factors, metabolic factors, sleep disorders, physical activity, and the risk of multiple sclerosis: A univariable and multivariable Mendelian Randomization study**

**Supplementary Figure 1** Scatter plots illustrate causality analysis of exposure on multiple sclerosis.

Snoring, sleep duration, long sleep duration, sleep apnea syndrome, sleeplessness/insomnia, tea intake, beef intake, coffee intake, poultry intake, cheese intake, processed meat intake, dried fruit intake, salad/raw vegetable intake, average weekly red wine intake, waist-to-hip ratio, waist circumference, essential hypertension, HDL cholesterol levels, LDL cholesterol levels, vigorous physical activity and strenuous sports or other exercises as exposure and multiple sclerosis as outcome. [Page 2-12]

**Abbreviation:** SNP, single nucleotide polymorphism; MR, Mendelian randomization; low-density lipoprotein, LDL; HDL, high-density lipoprotein.

**Supplementary Figure 2** Leave-one-out analysis illustrates causality analysis of exposure on multiple

sclerosis. Snoring, chronotype, sleep duration, short sleep duration, long sleep duration, sleep apnea syndrome, sleeplessness/insomnia, tea intake, beef intake, pork intake, coffee intake, poultry intake, cheese intake, processed meat intake, dried fruit intake, salad/raw vegetable intake, average weekly red wine intake, fish/liver oil dietary supplements, body mass index, waist-to-hip ratio, hip circumference, waist circumference, essential hypertension, HDL cholesterol levels, LDL cholesterol levels, type 2 diabetes, vigorous physical activity, strenuous sports or other exercises and moderate to vigorous physical activity, as exposure and multiple sclerosis as outcome. [Page 13-27]

**Abbreviation:** SNP, single nucleotide polymorphism; MR, Mendelian randomization; low-density lipoprotein, LDL; HDL, high-density lipoprotein.


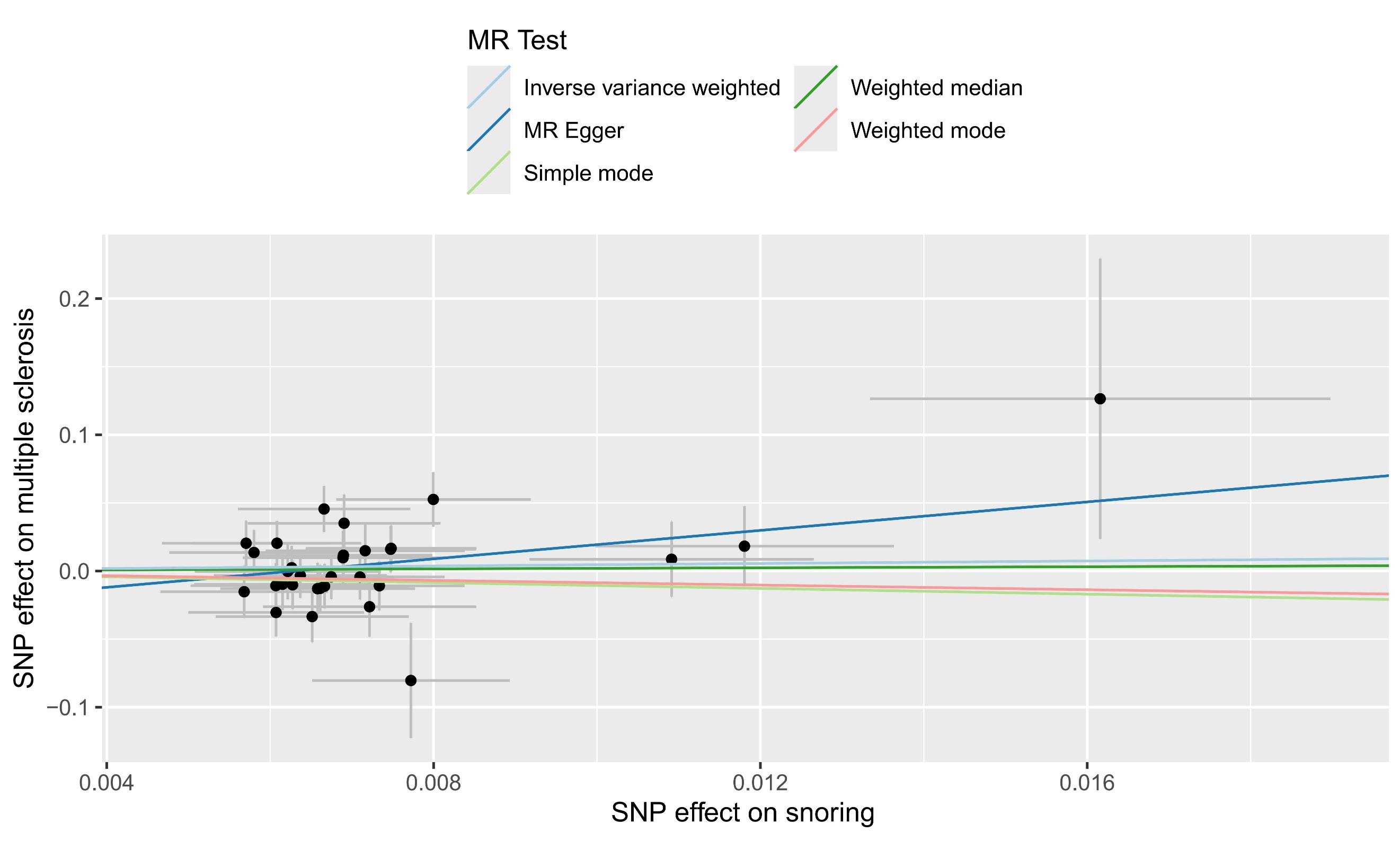


**Supplementary Figure 1A** Scatter plots illustrate causality analysis of snoring on multiple sclerosis


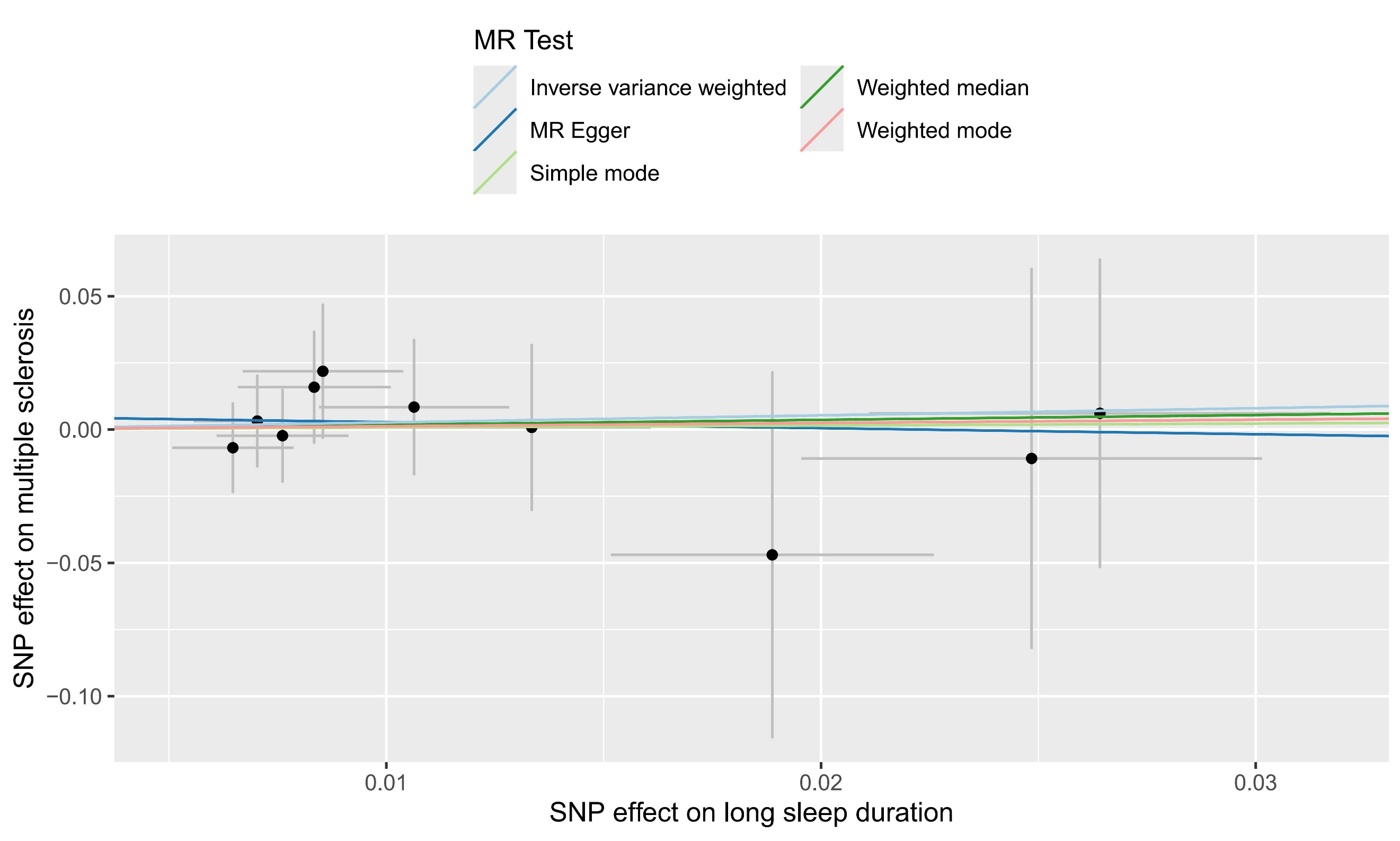


**Supplementary Figure 1B** Scatter plots illustrate causality analysis of long sleep duration on multiple sclerosis


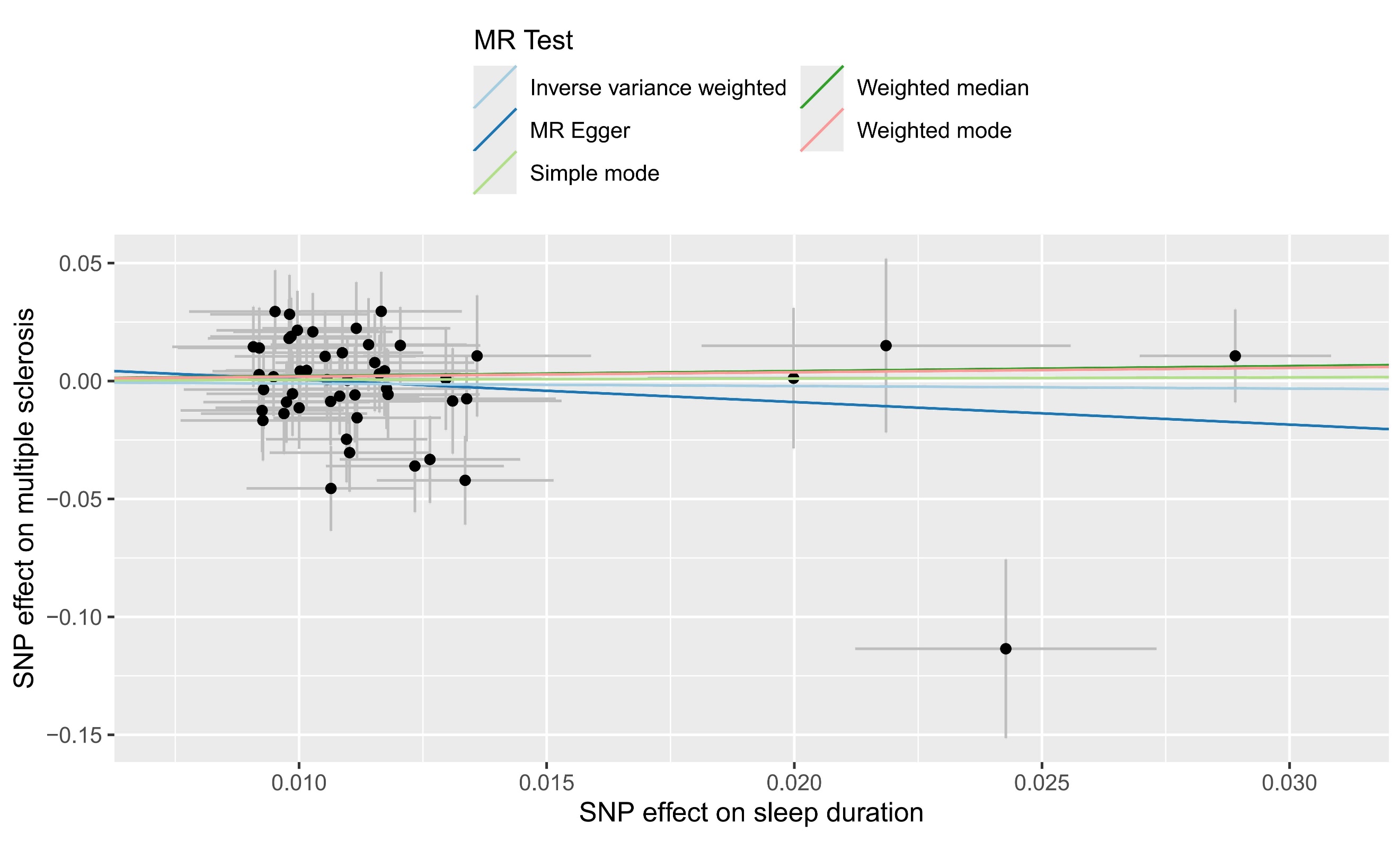


**Supplementary Figure 1C** Scatter plots illustrate causality analysis of sleep duration on multiple sclerosis


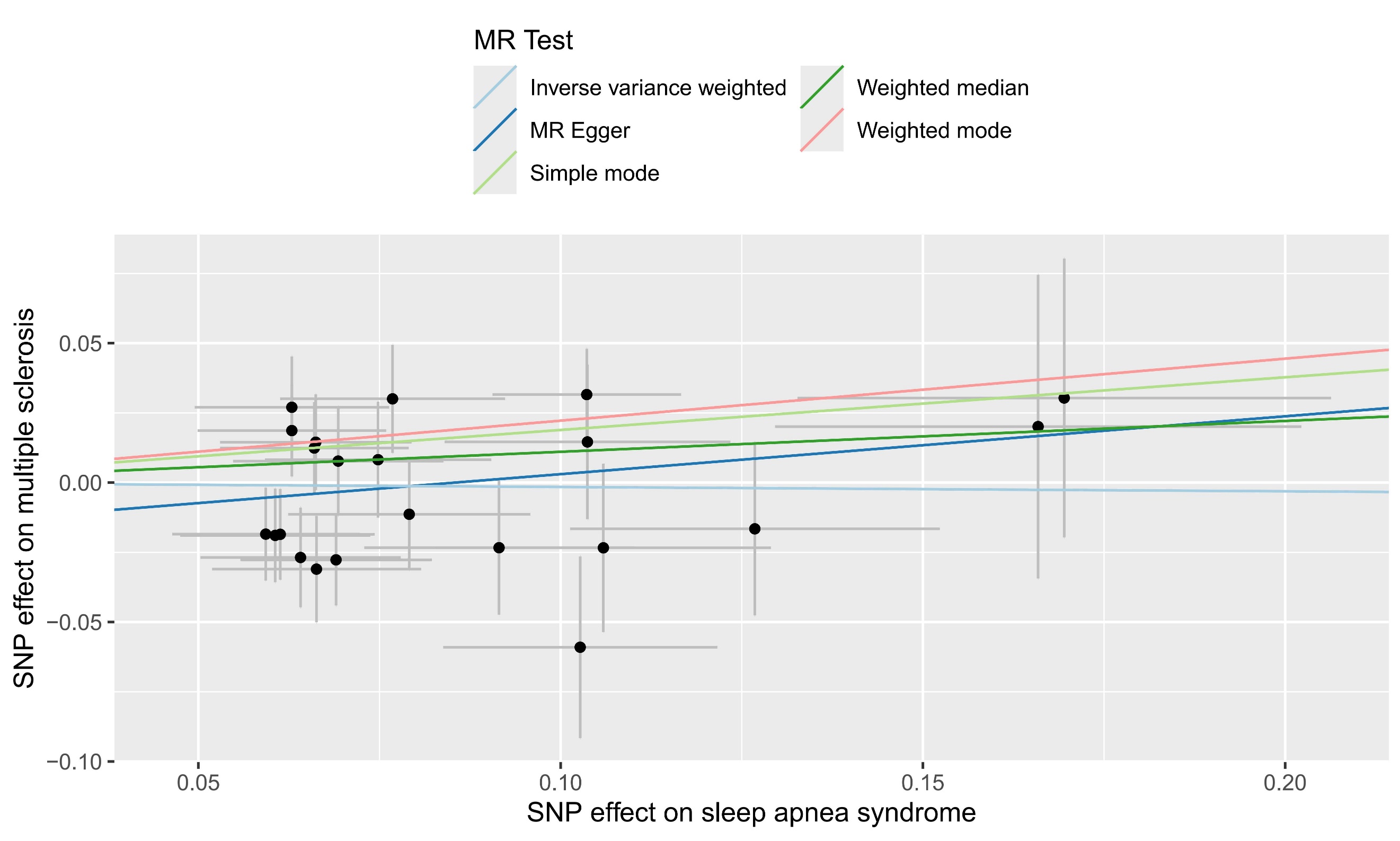


**Supplementary Figure 1D** Scatter plots illustrate causality analysis of sleep apnea syndrome on multiple sclerosis


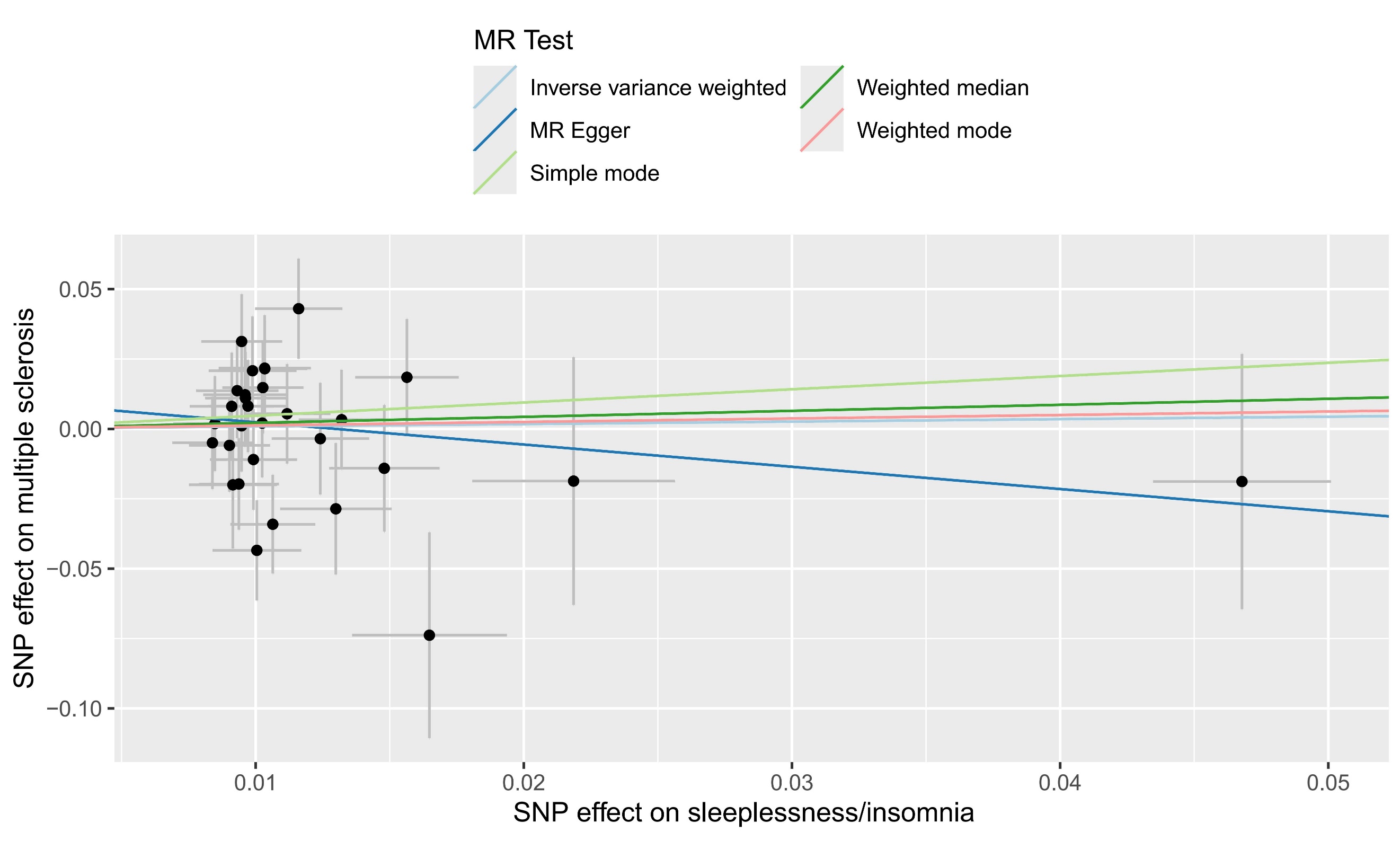


**Supplementary Figure 1E** Scatter plots illustrate causality analysis of sleeplessness/insomnia on multiple sclerosis


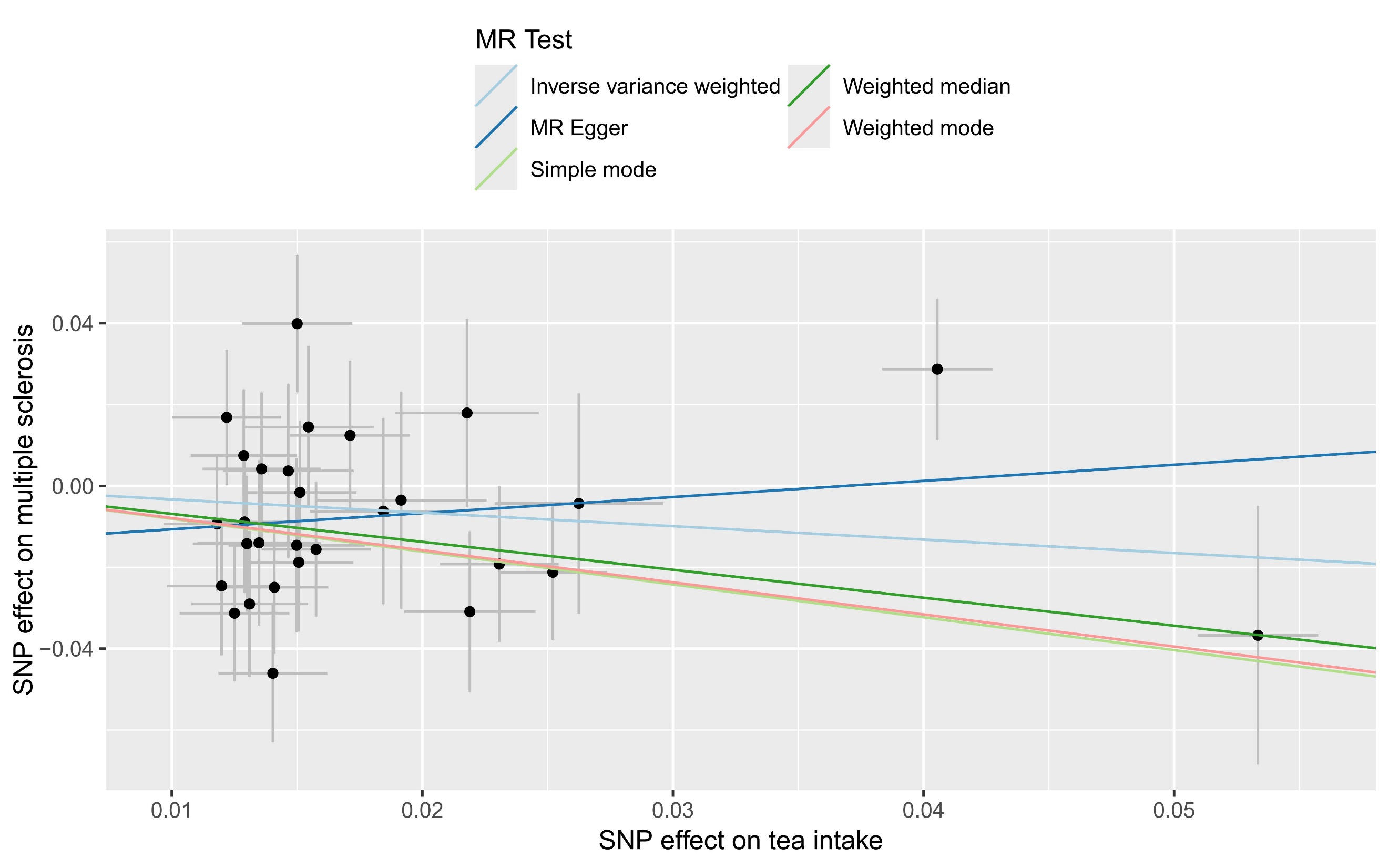


**Supplementary Figure 1F** Scatter plots illustrate causality analysis of tea intake on multiple sclerosis


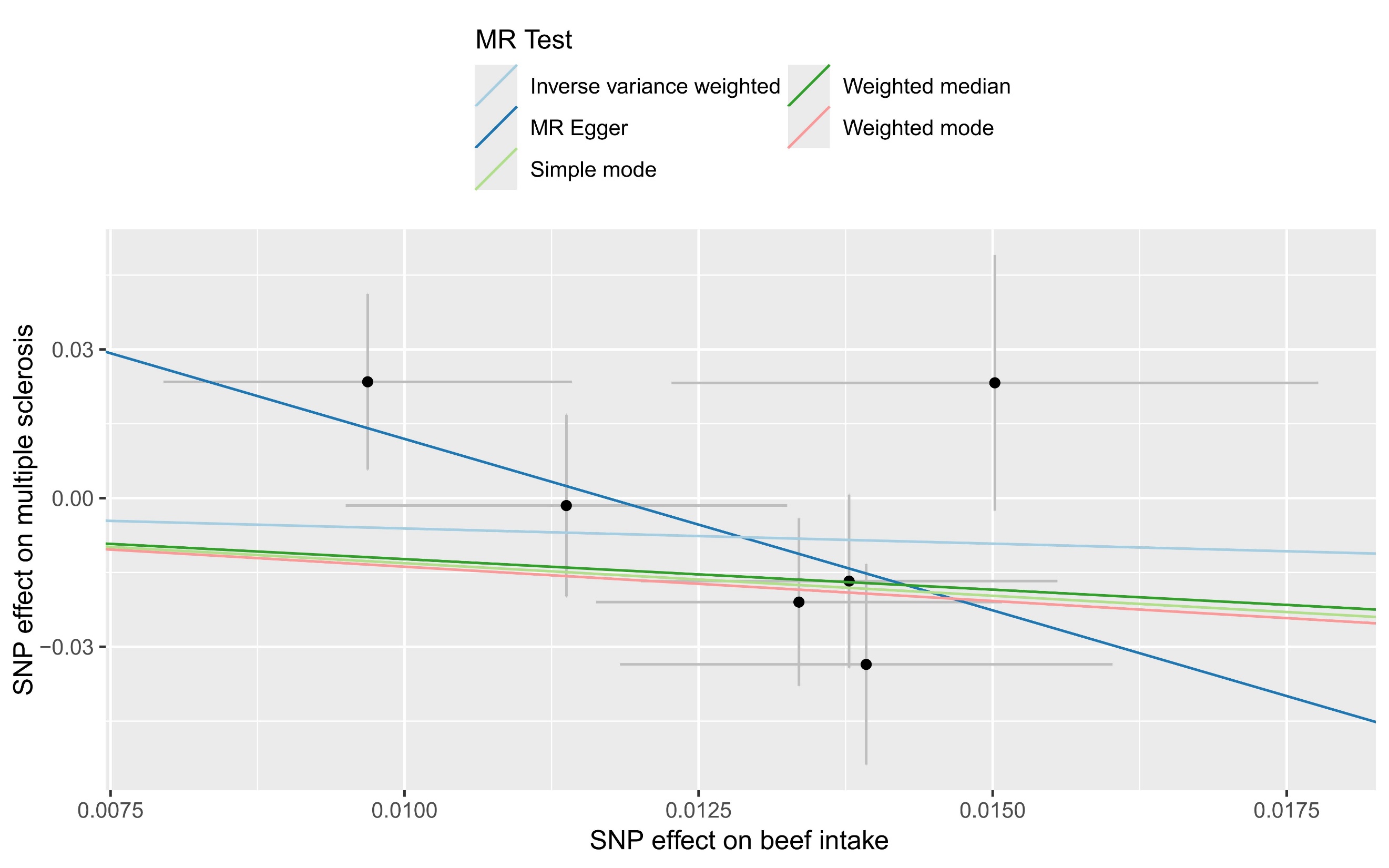
**Supplementary Figure 1G** Scatter plots illustrate causality analysis of beef intake on multiple sclerosis


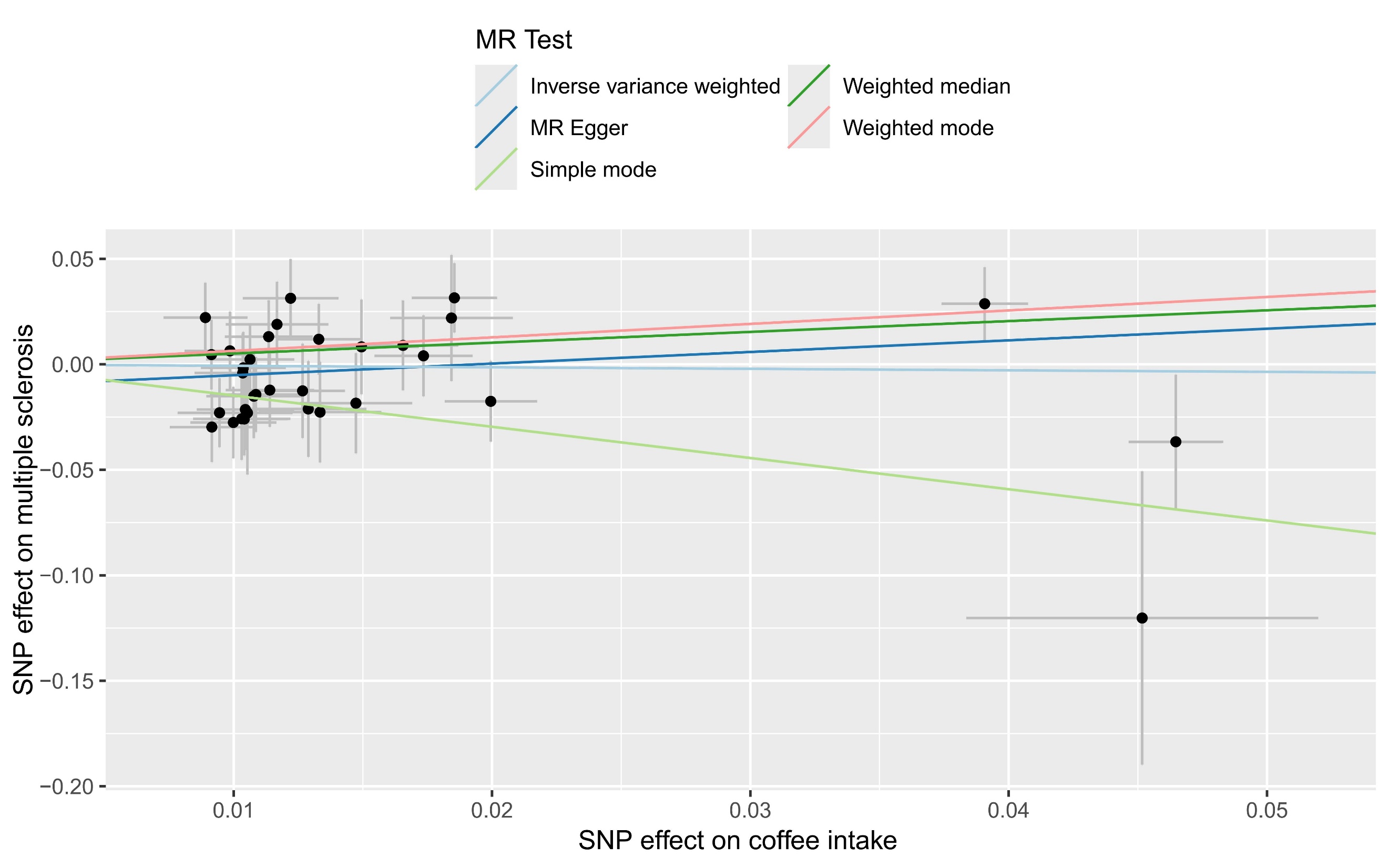


**Supplementary Figure 1H** Scatter plots illustrate causality analysis of coffee intake on multiple sclerosis


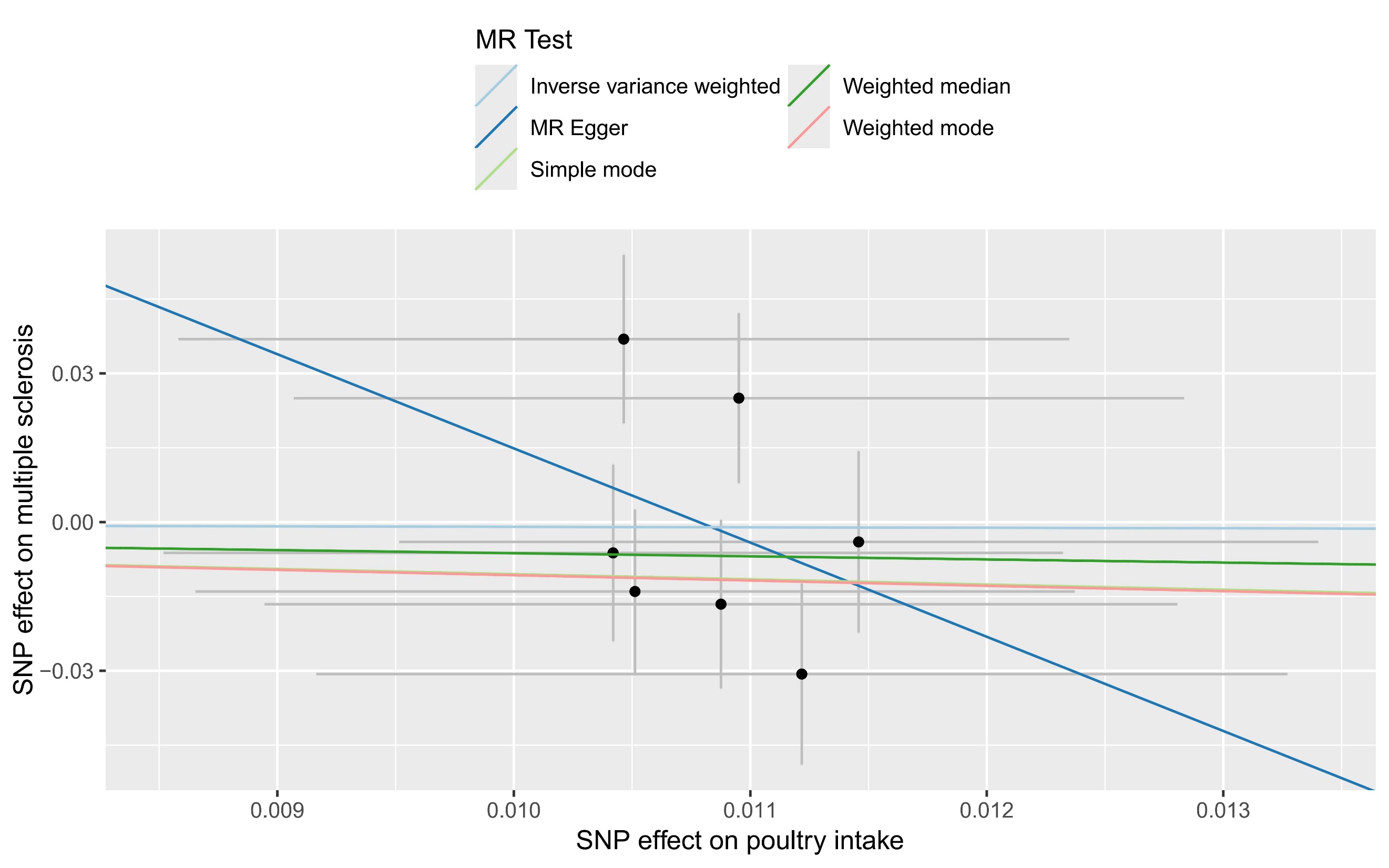


**Supplementary Figure 1I** Scatter plots illustrate causality analysis of poultry intake on multiple sclerosis


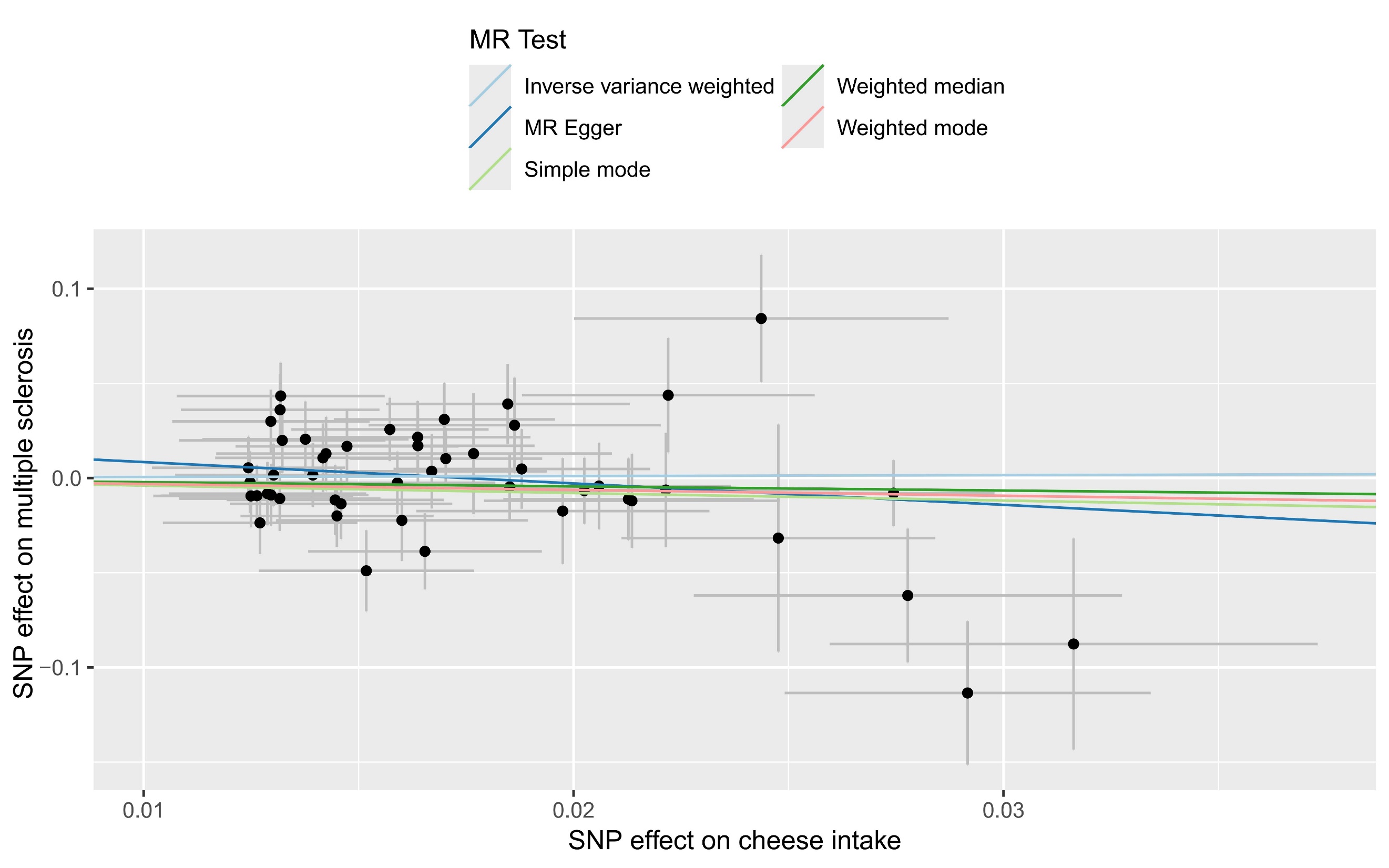


**Supplementary Figure 1J** Scatter plots illustrate causality analysis of cheese intake on multiple sclerosis


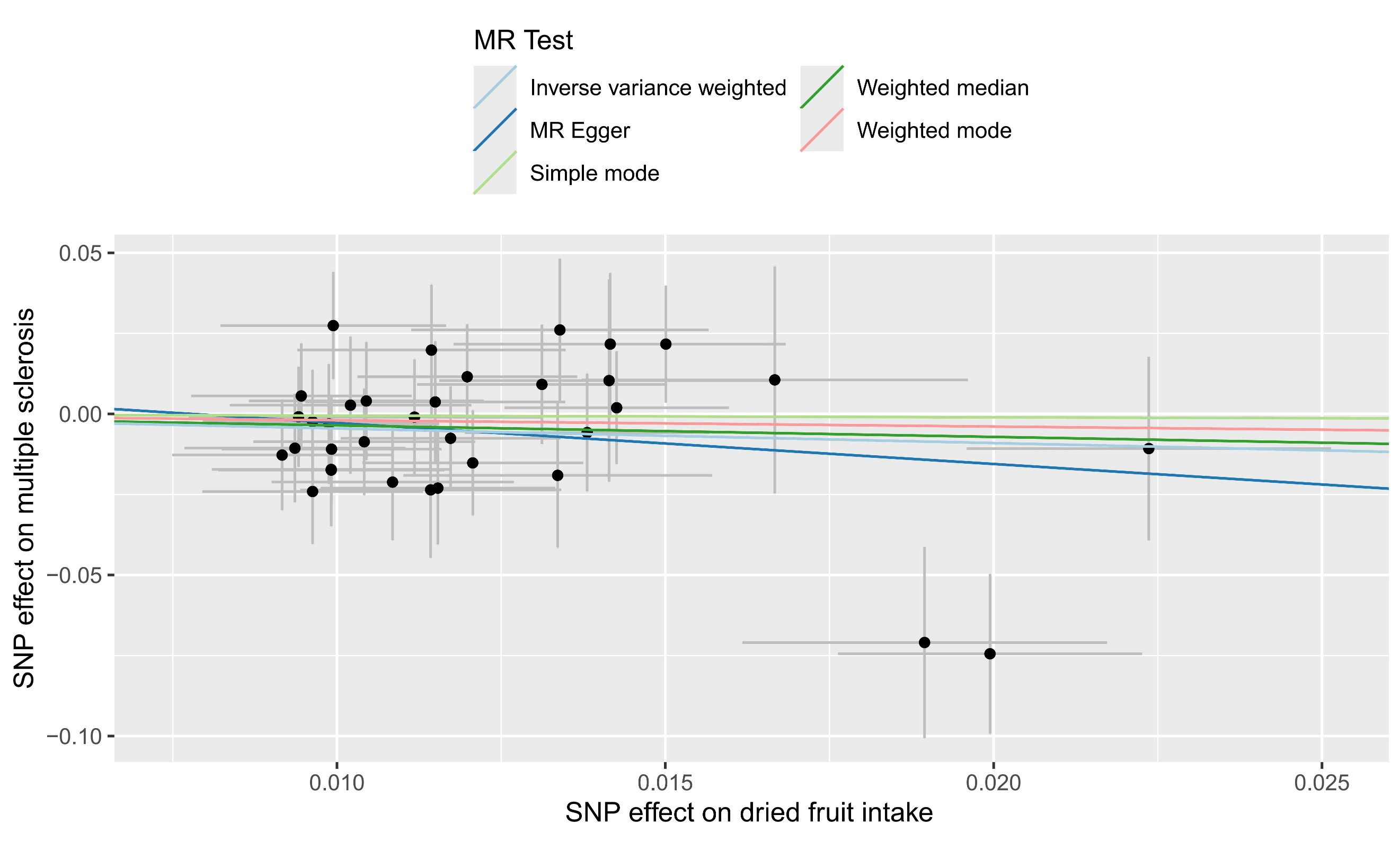


**Supplementary Figure 1K** Scatter plots illustrate causality analysis of dried fruit intake on multiple sclerosis


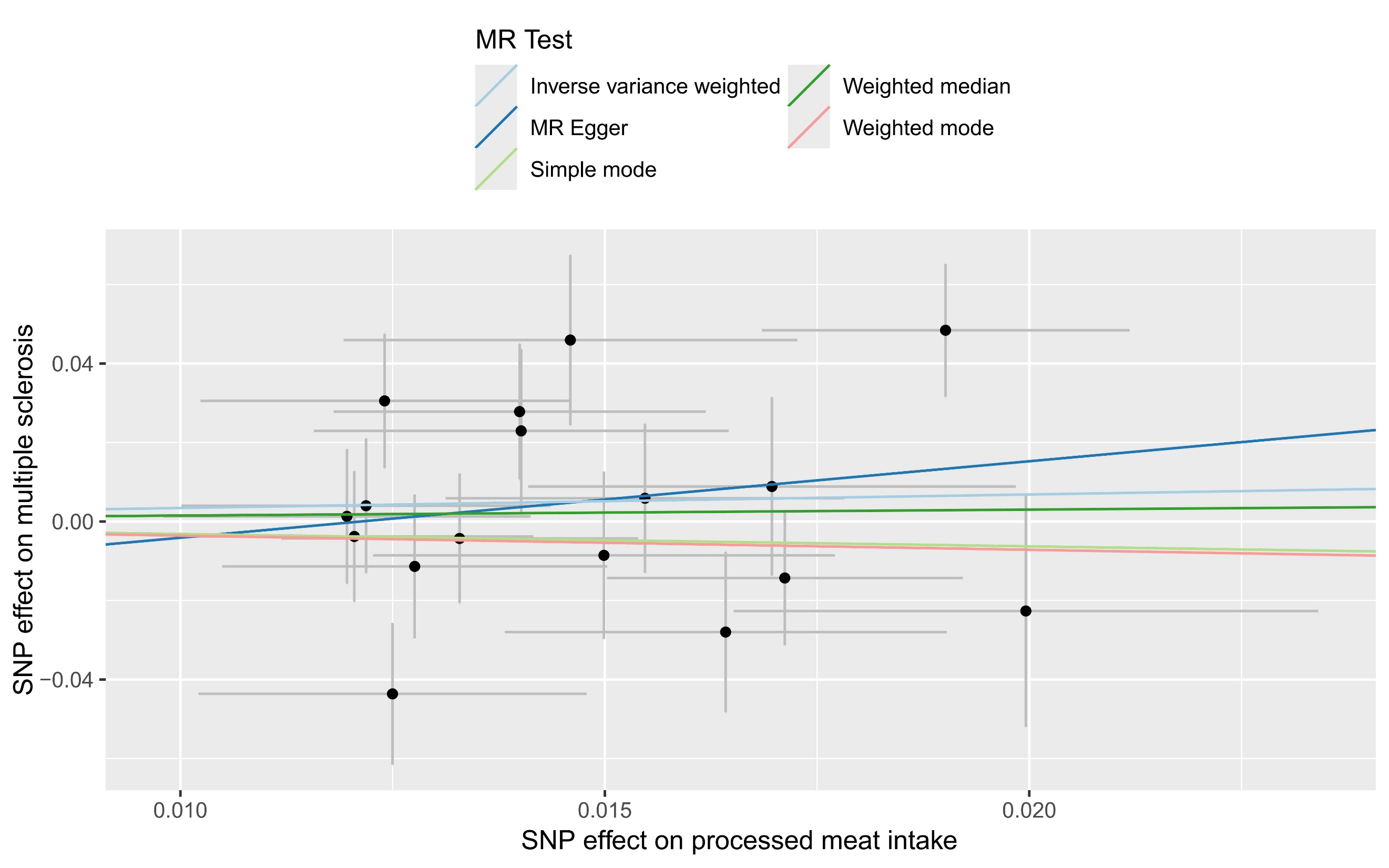


**Supplementary Figure 1L** Scatter plots illustrate causality analysis of processed meat intake on multiple sclerosis


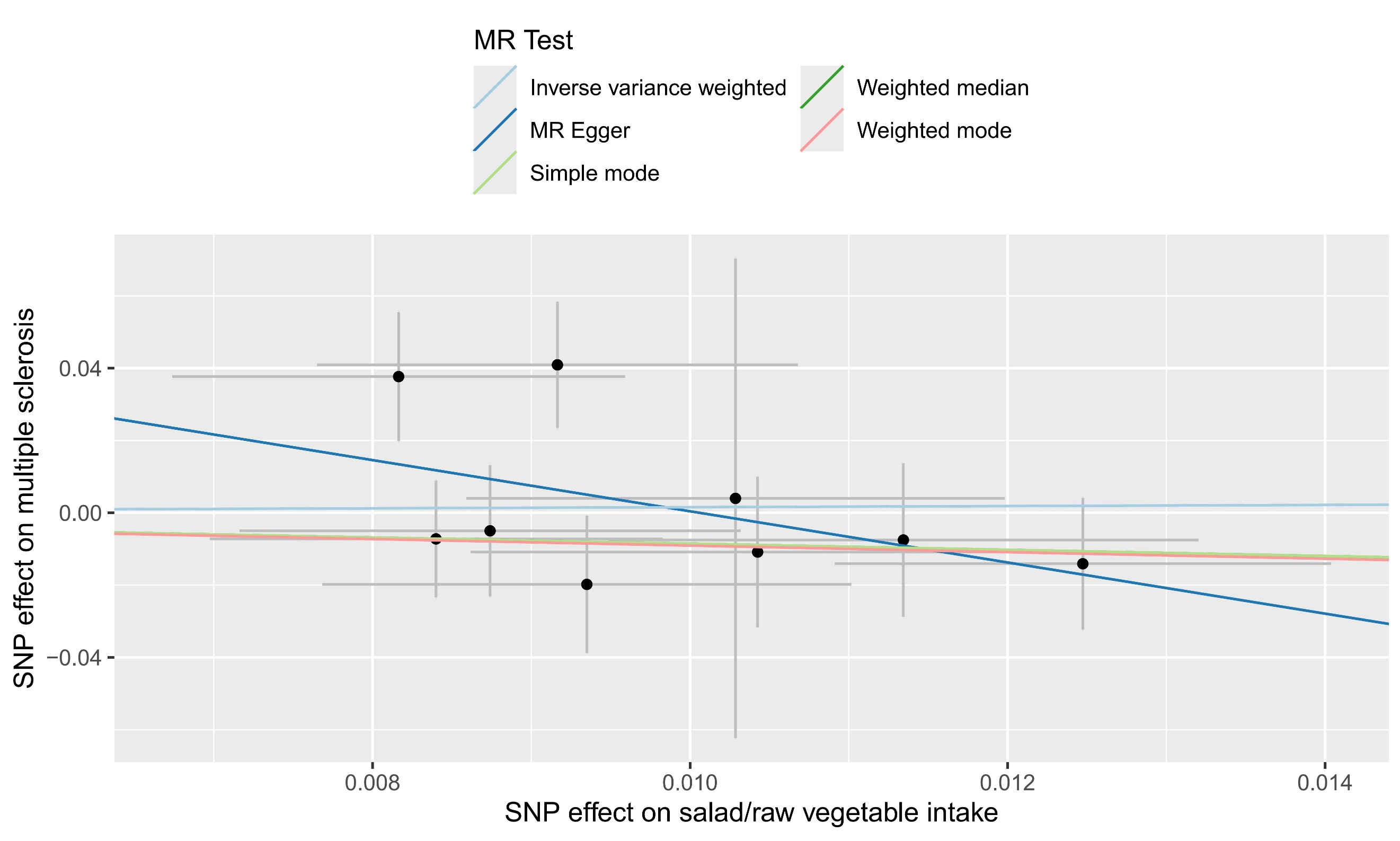


**Supplementary Figure 1M** Scatter plots illustrate causality analysis of salad/raw vegetable intake on multiple sclerosis


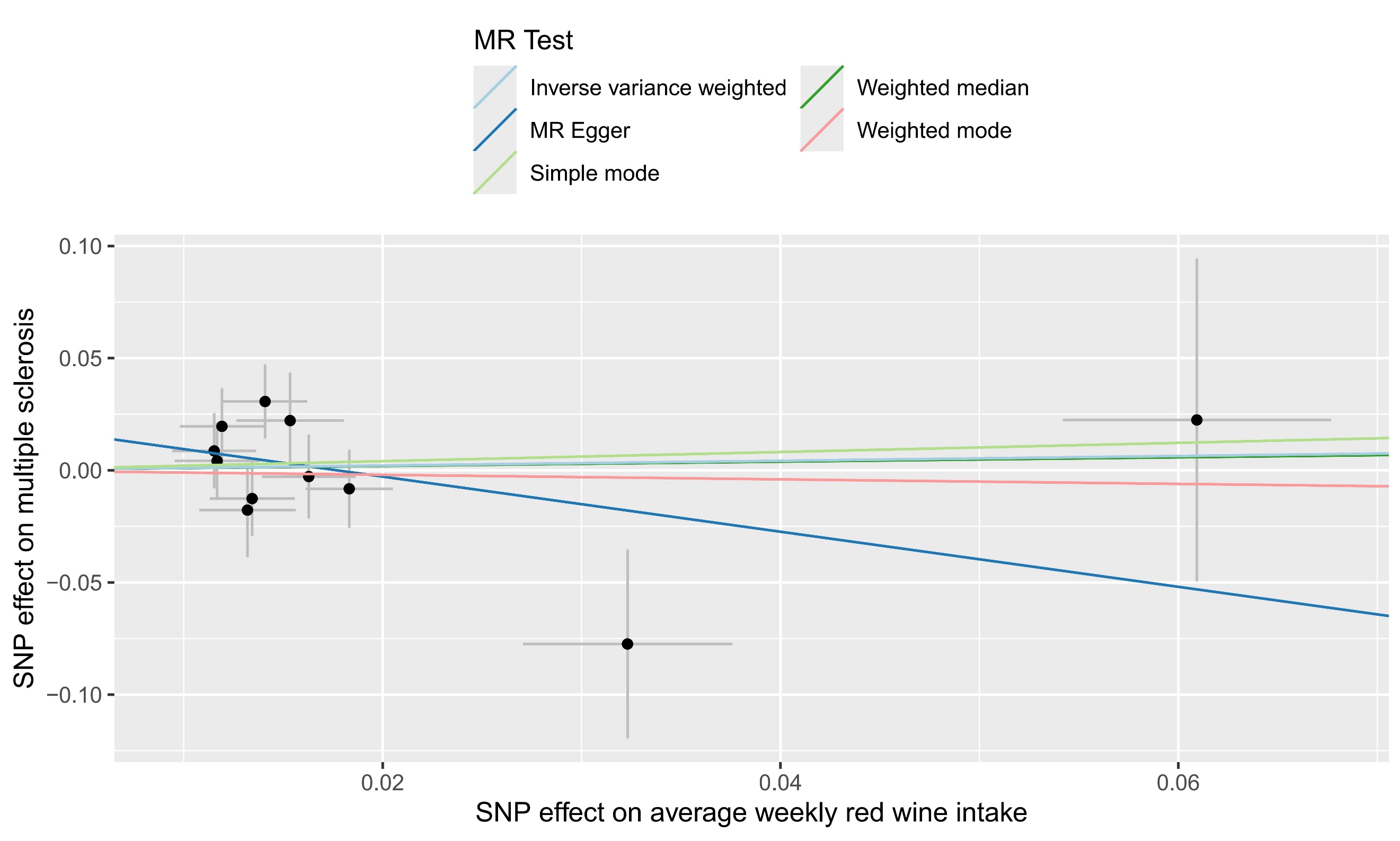


**Supplementary Figure 1N** Scatter plots illustrate causality analysis of average weekly red wine intake on multiple sclerosis


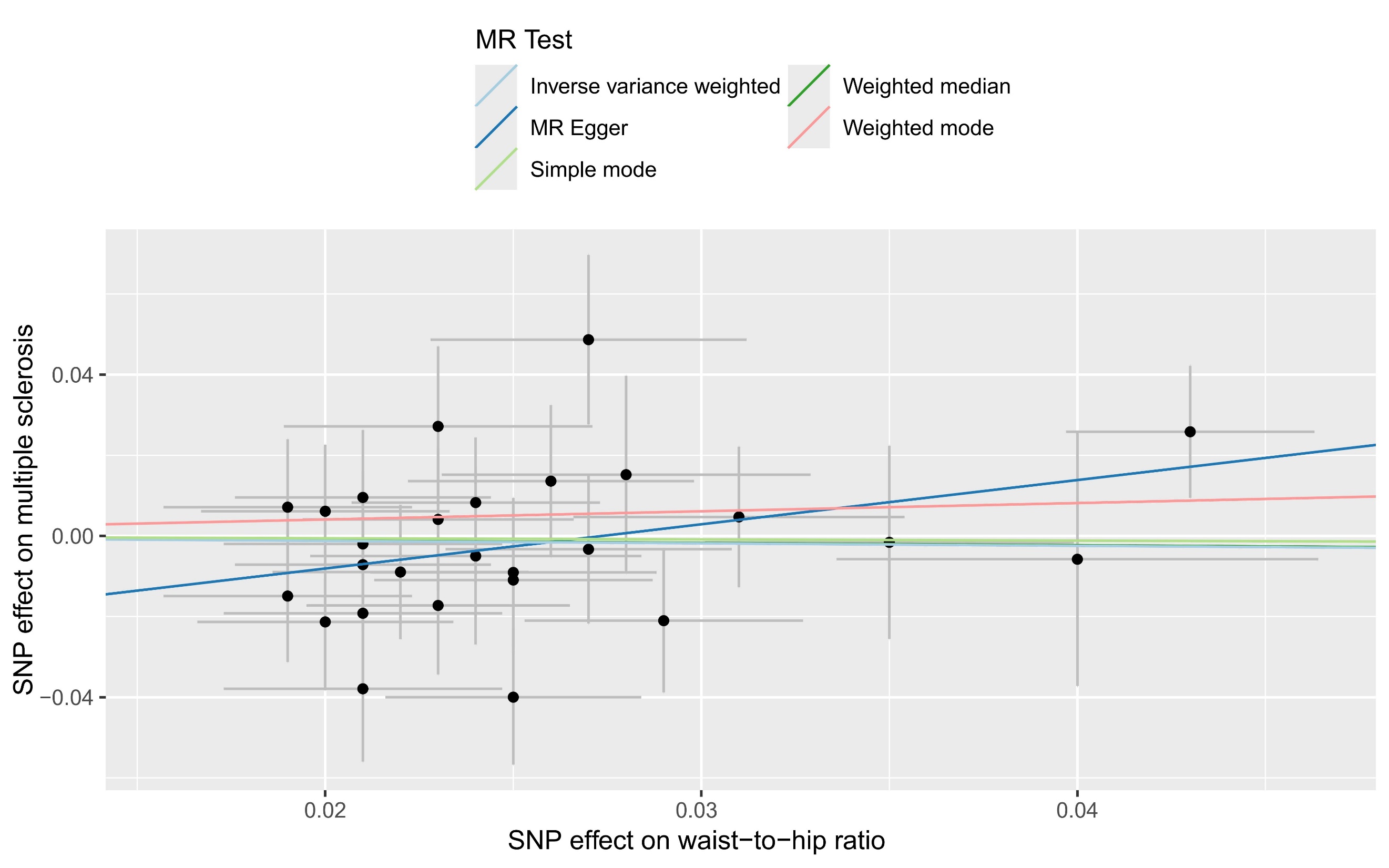


**Supplementary Figure 1O** Scatter plots illustrate causality analysis of waist-to-hip ratio on multiple sclerosis


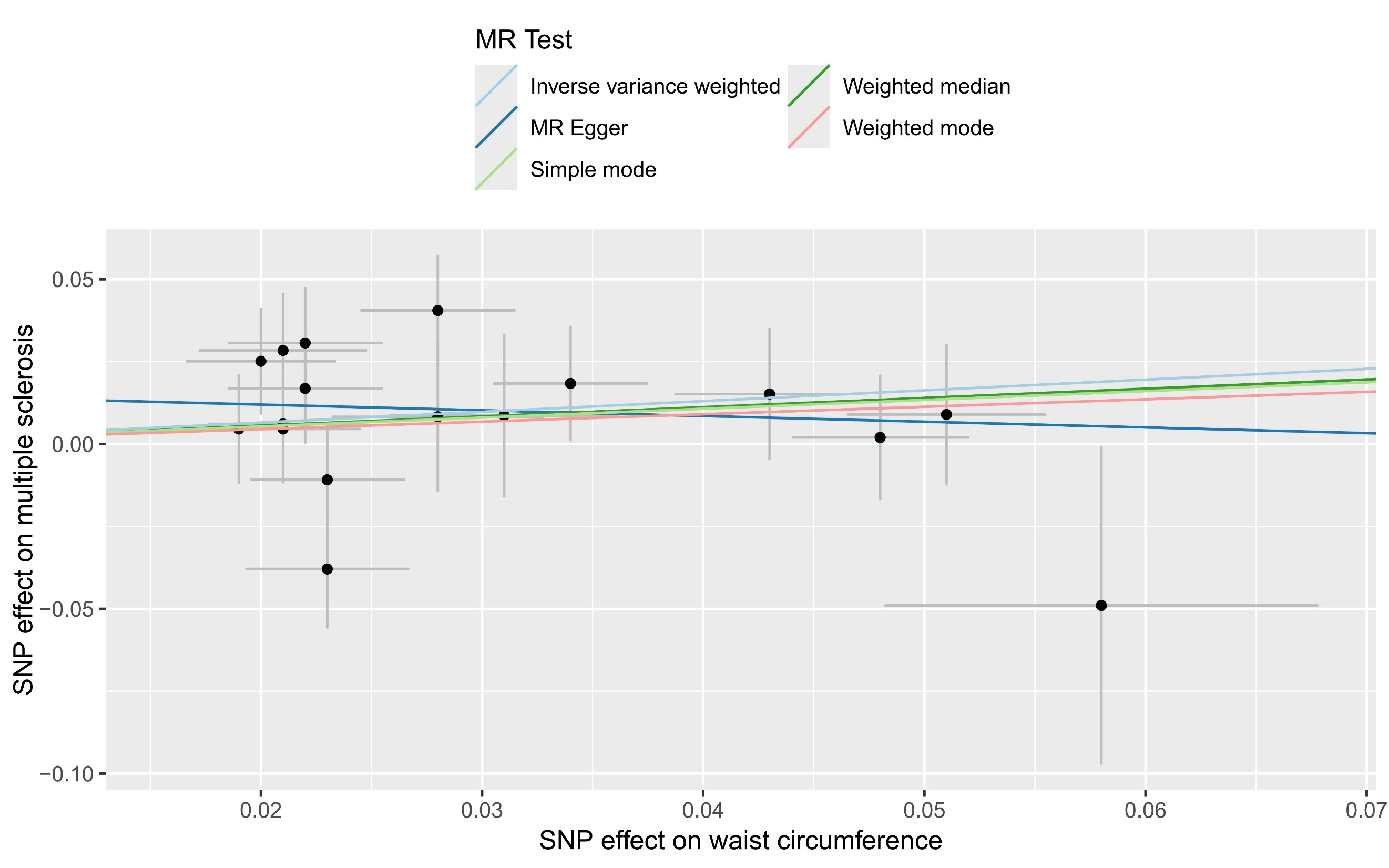


**Supplementary Figure 1P** Scatter plots illustrate causality analysis of waist circumference on multiple sclerosis


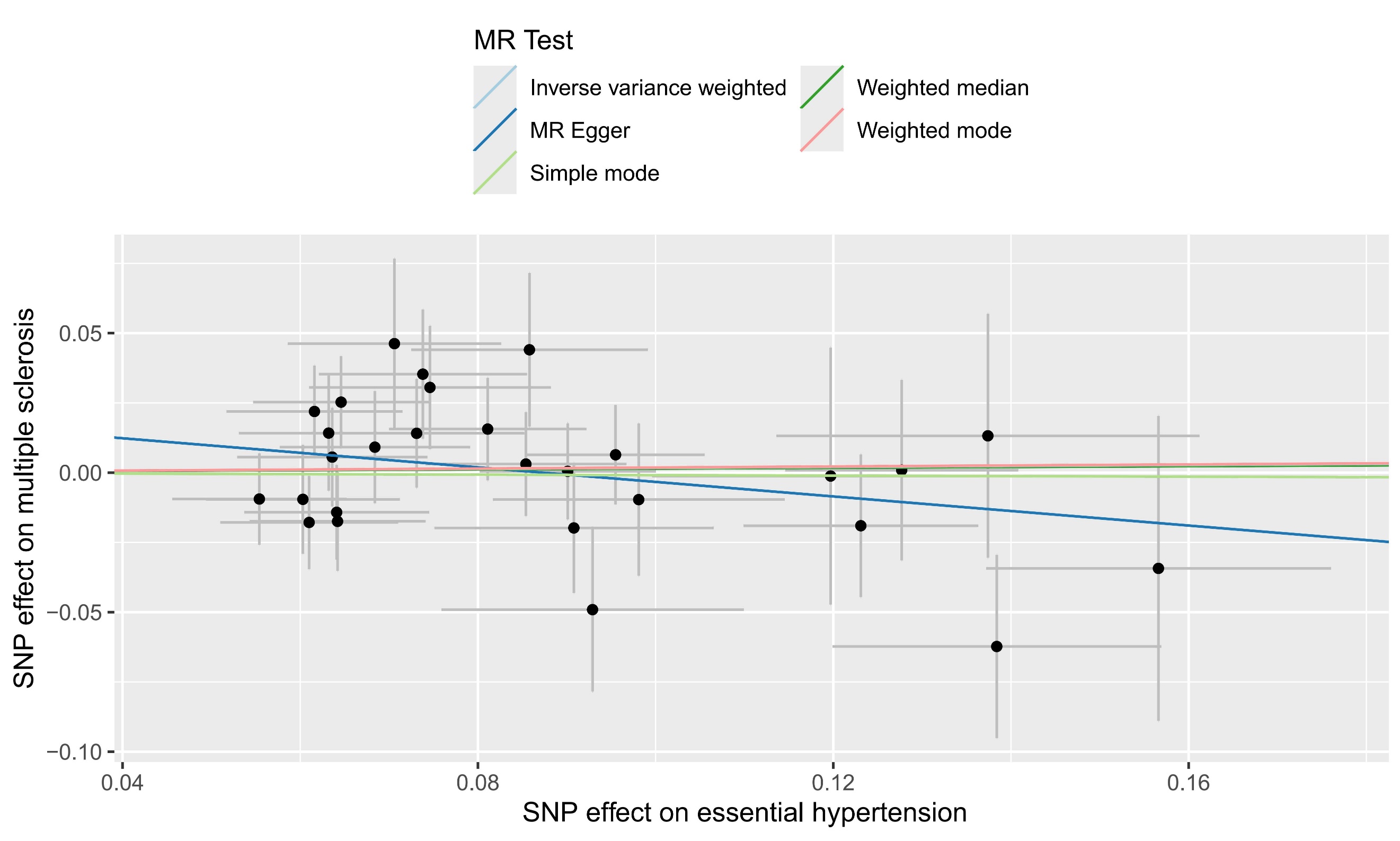


**Supplementary Figure 1Q** Scatter plots illustrate causality analysis of essential hypertension on multiple sclerosis


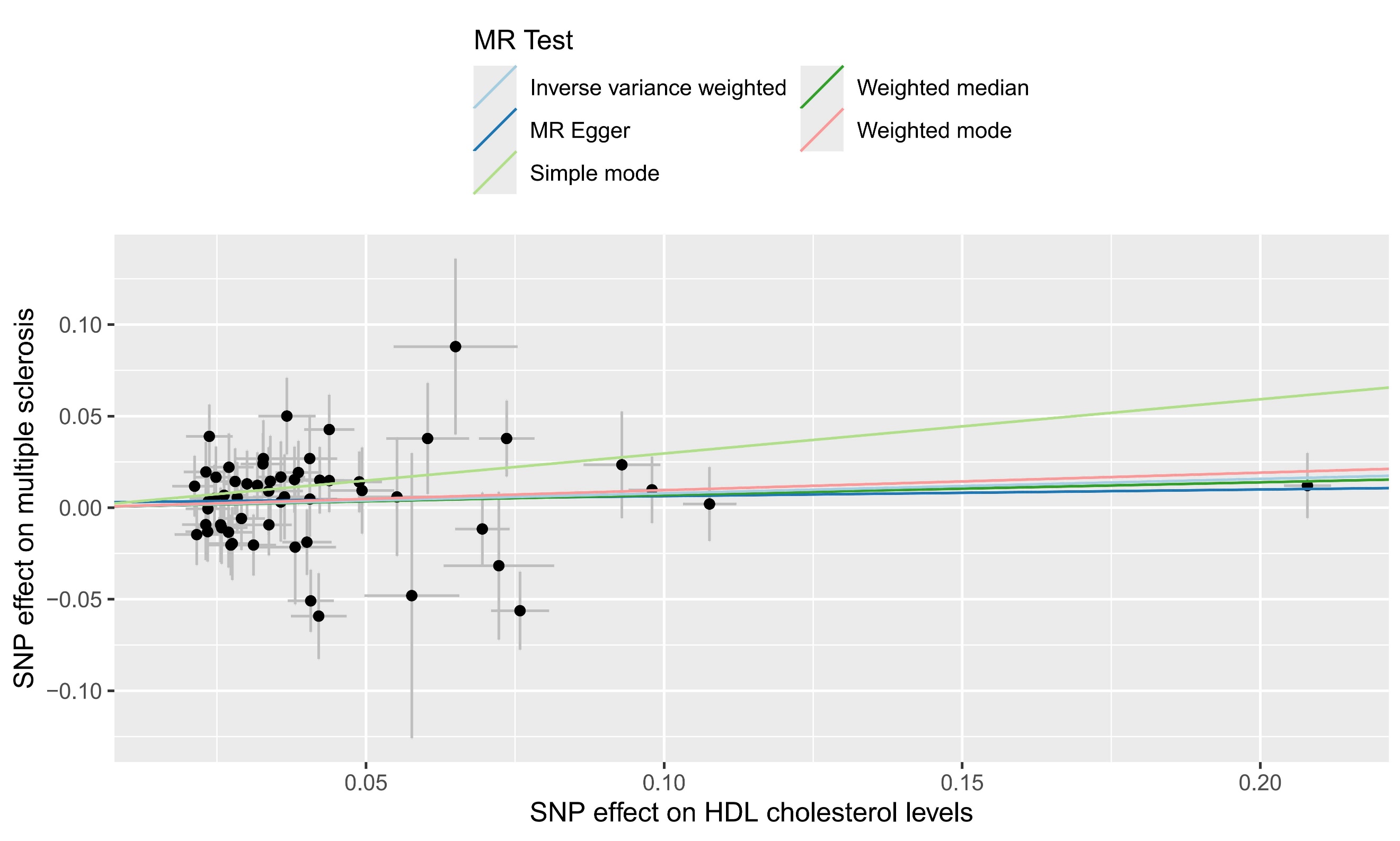


**Supplementary Figure 1R** Scatter plots illustrate causality analysis of HDL cholesterol levels on multiple sclerosis


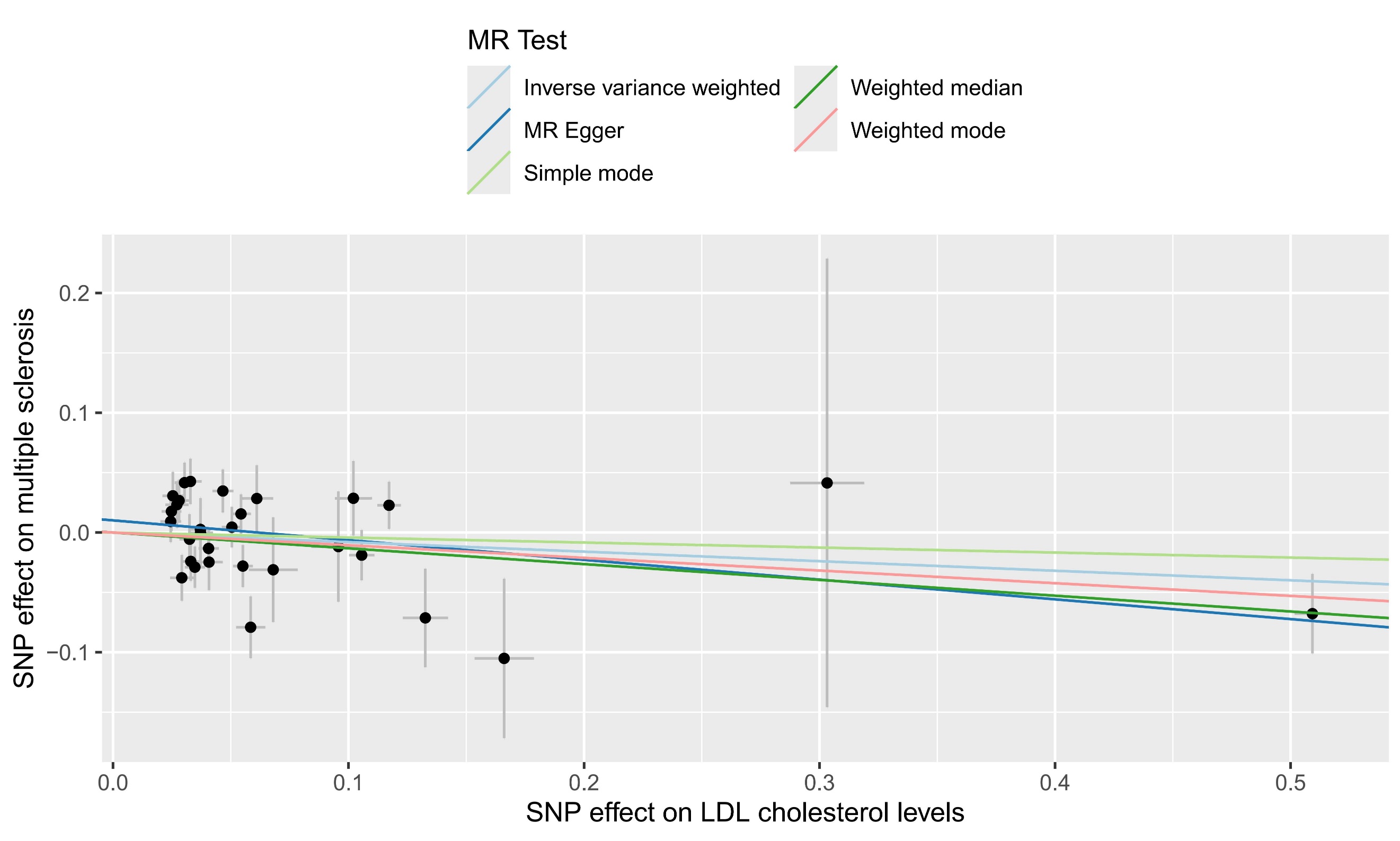


**Supplementary Figure 1S** Scatter plots illustrate causality analysis of LDL cholesterol levels on multiple sclerosis


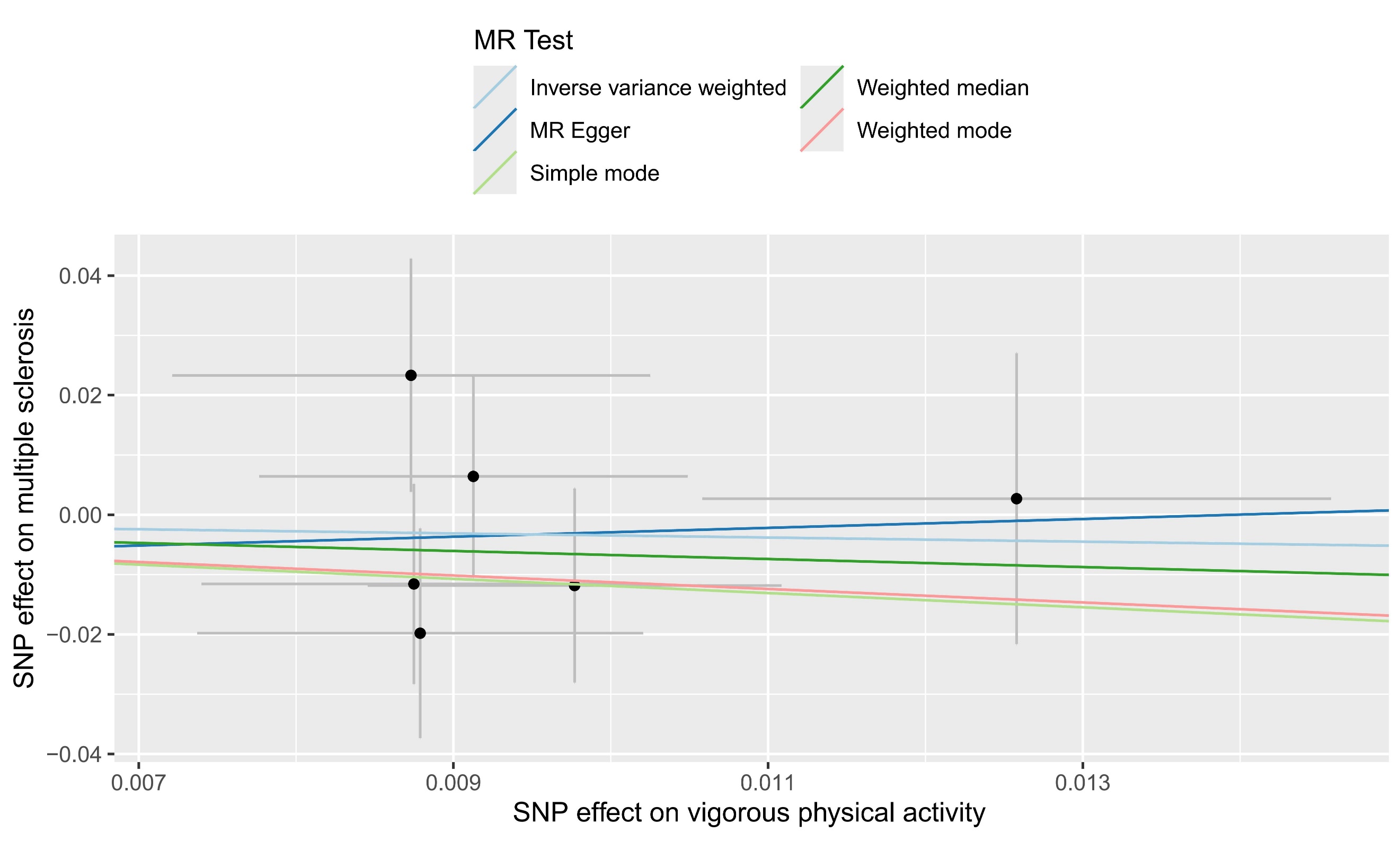


**Supplementary Figure 1T** Scatter plots illustrate causality analysis of vigorous physical activity on multiple sclerosis


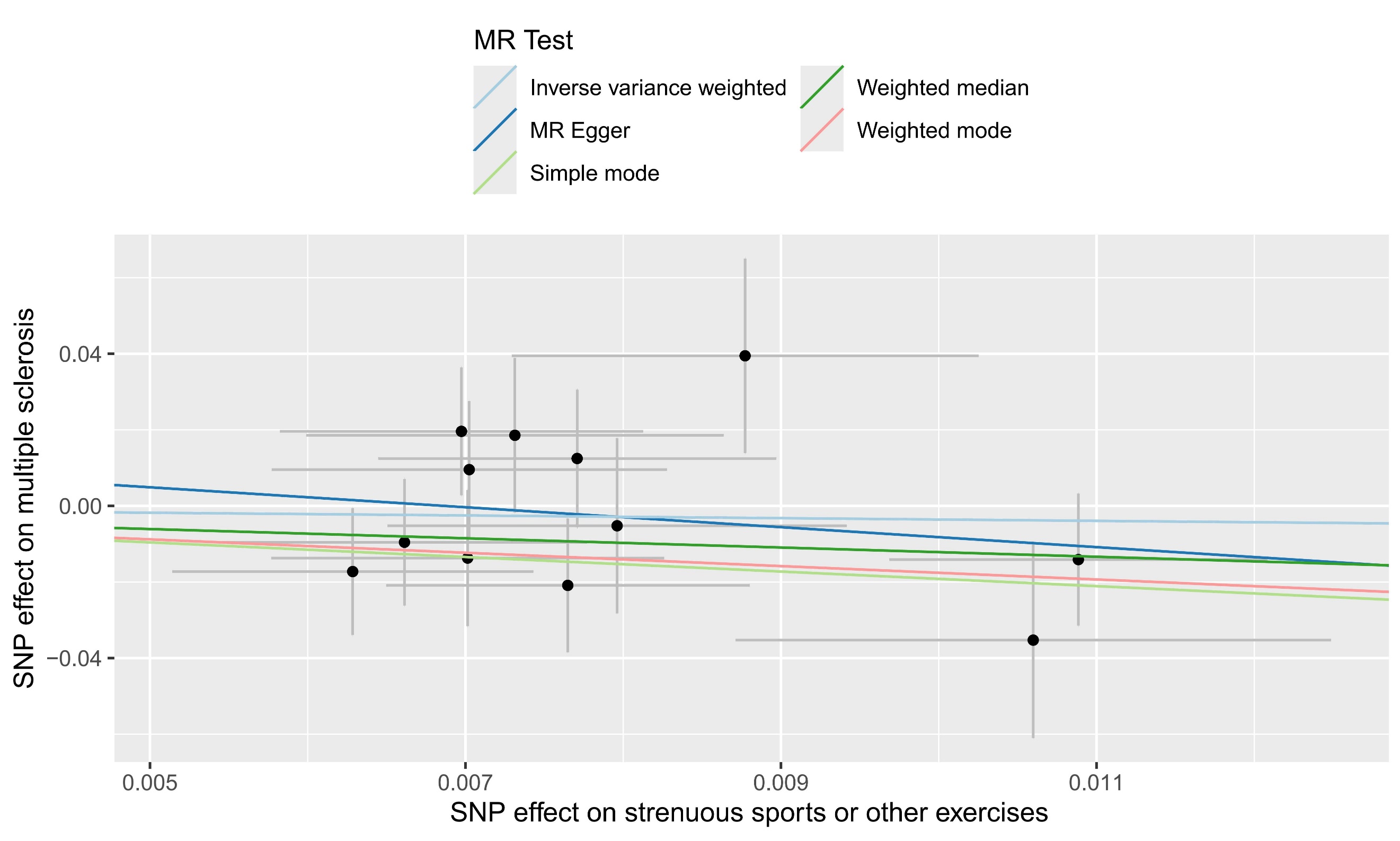


**Supplementary Figure 1U** Scatter plots illustrate causality analysis of strenuous sports or other exercises on multiple sclerosis


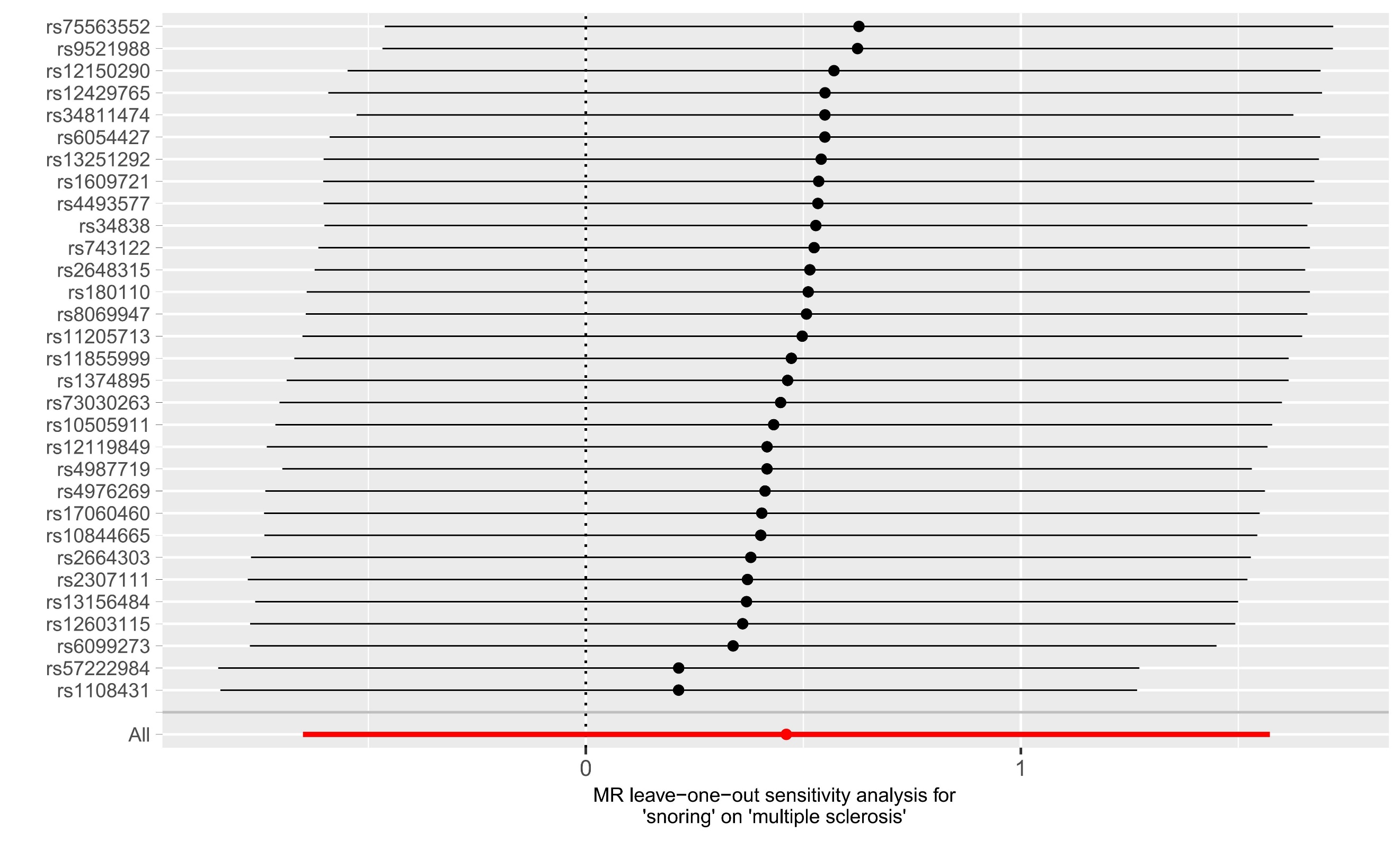


**Supplementary Figure 1A** Leave-one-out analysis illustrates causality analysis of snoring on multiple sclerosis


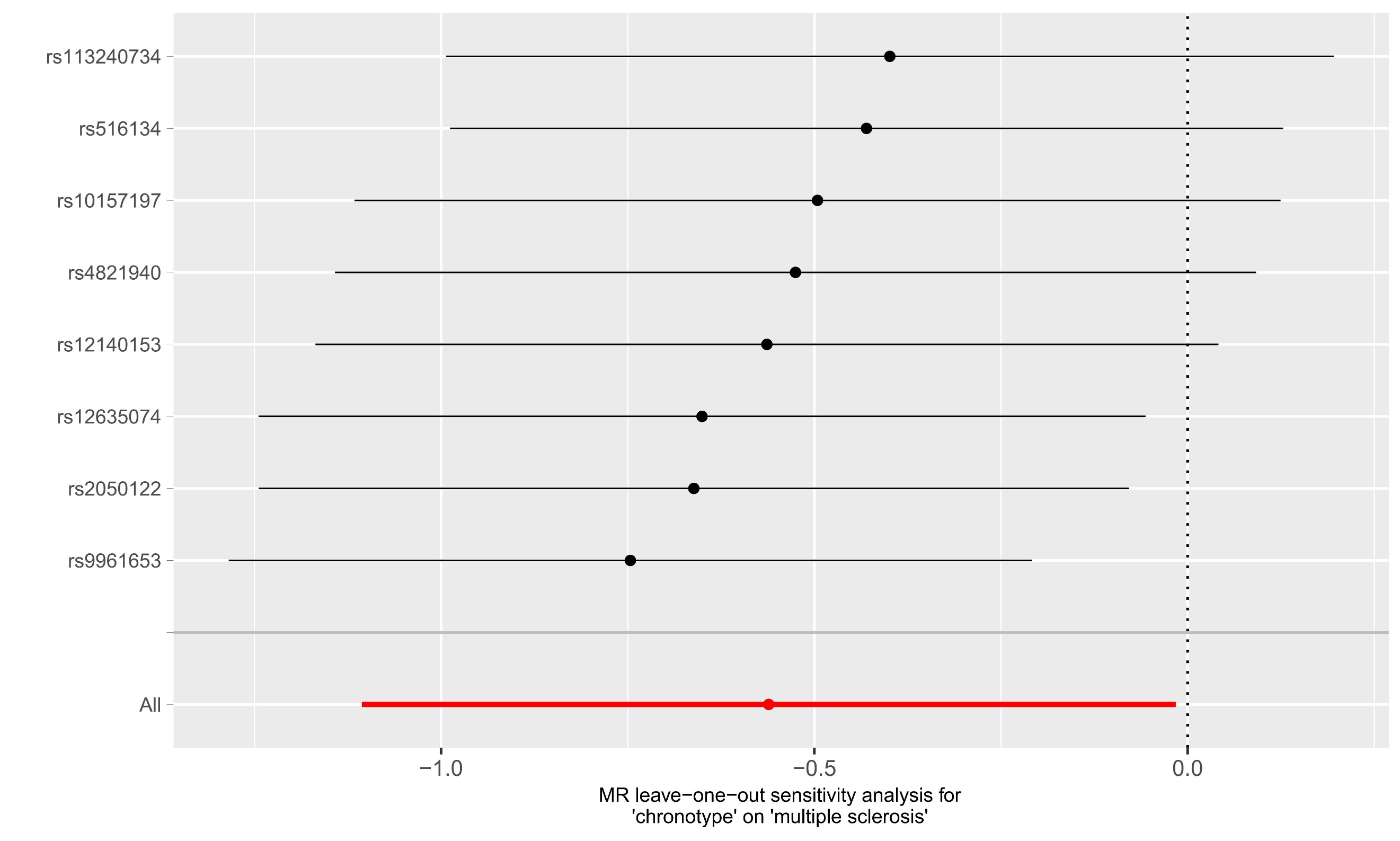


**Supplementary Figure 1B** Leave-one-out analysis illustrates causality analysis of chronotype on multiple sclerosis


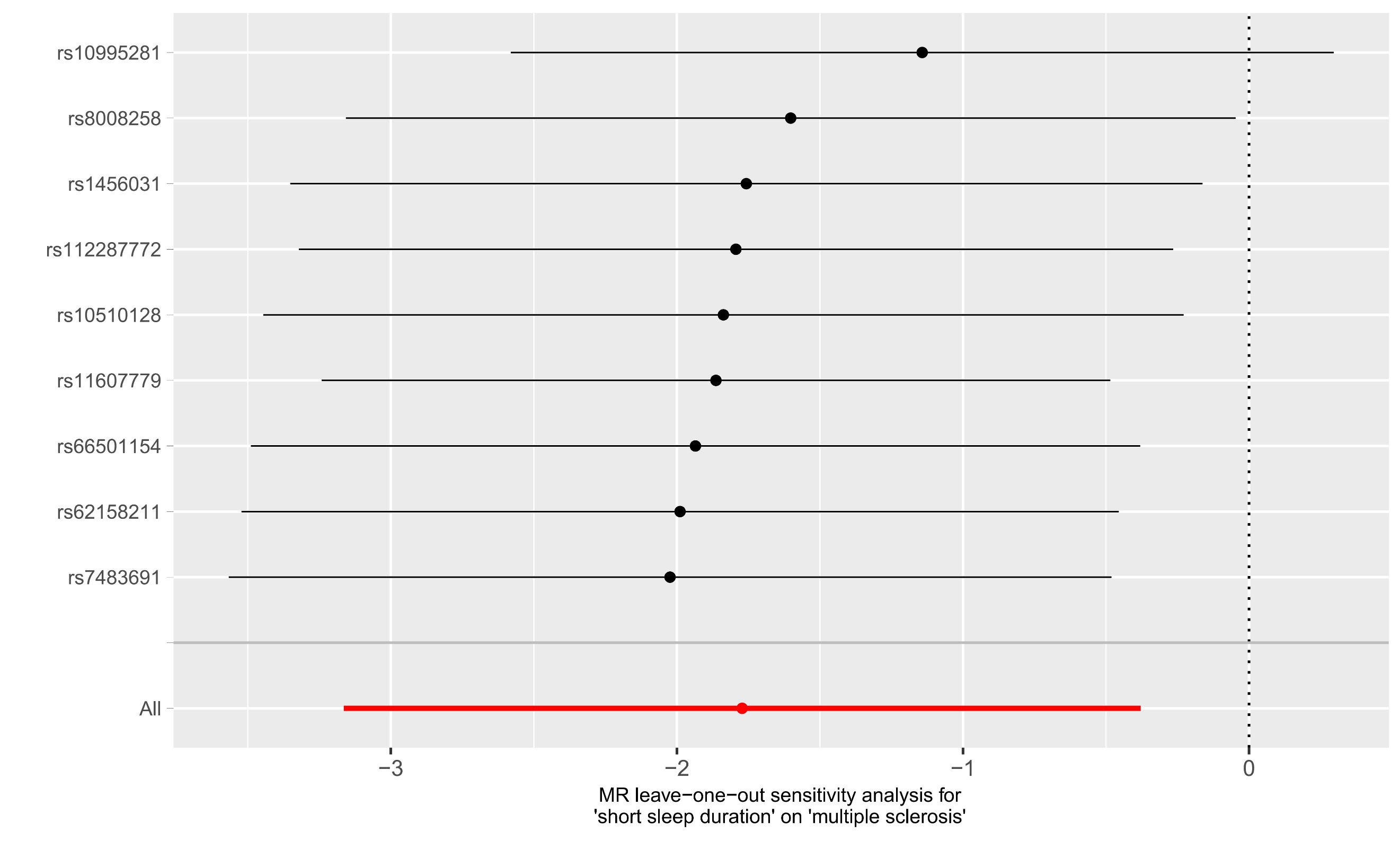


**Supplementary Figure 1C** Leave-one-out analysis illustrates causality analysis of short sleep duration on multiple sclerosis


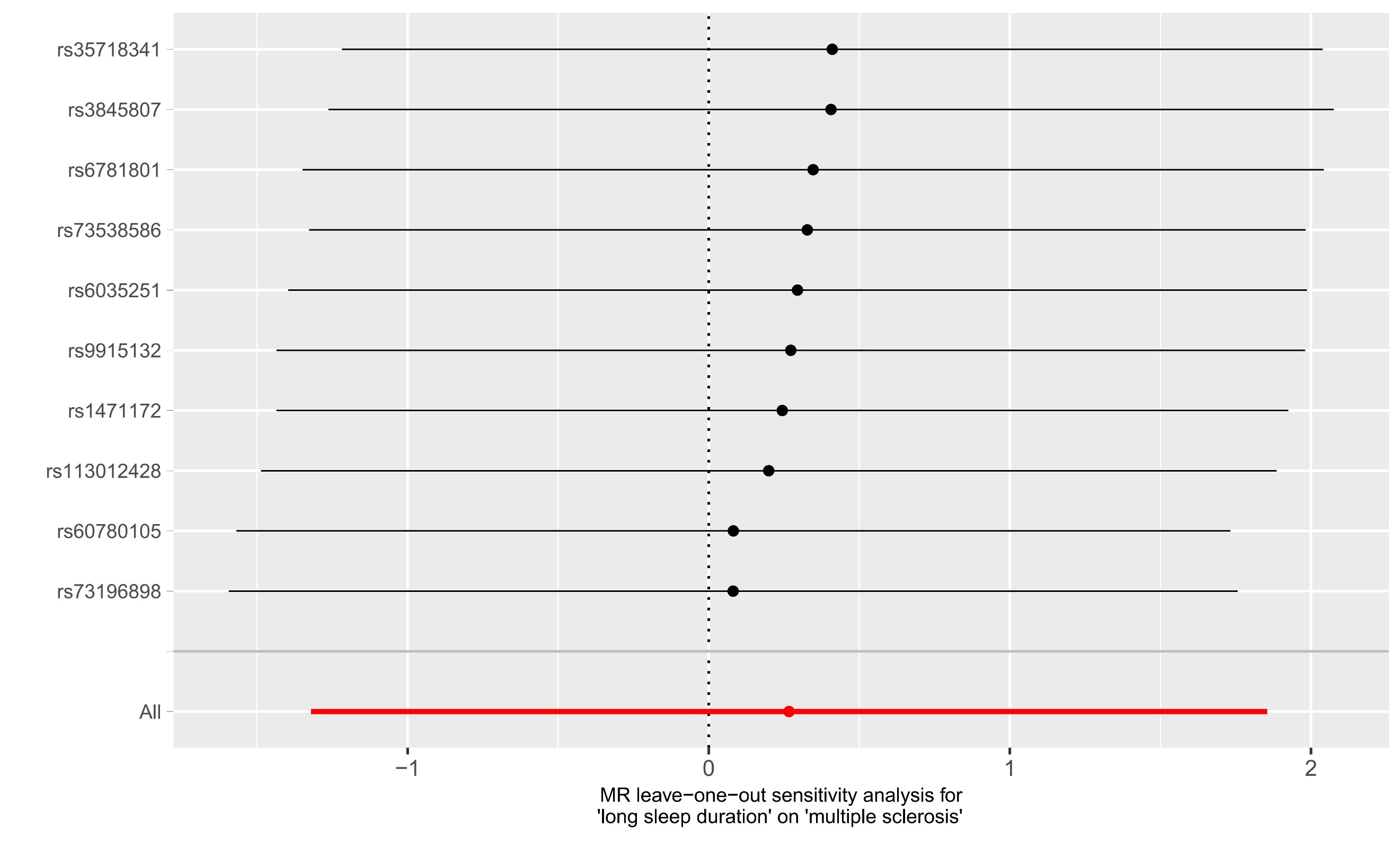


**Supplementary Figure 1D** Leave-one-out analysis illustrates causality analysis of long sleep duration on multiple sclerosis


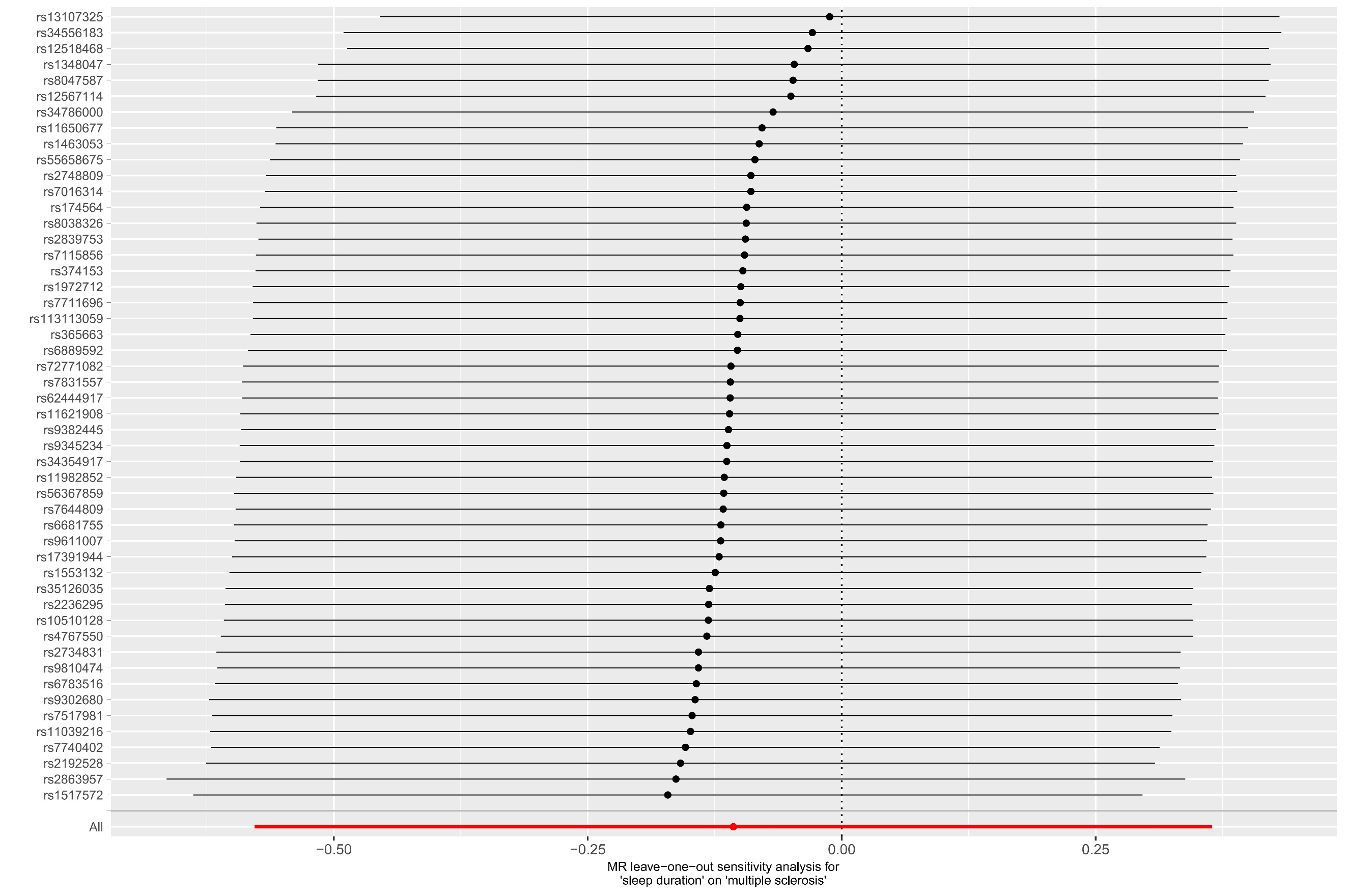


**Supplementary Figure 1E** Leave-one-out analysis illustrates causality analysis of sleep duration on multiple sclerosis


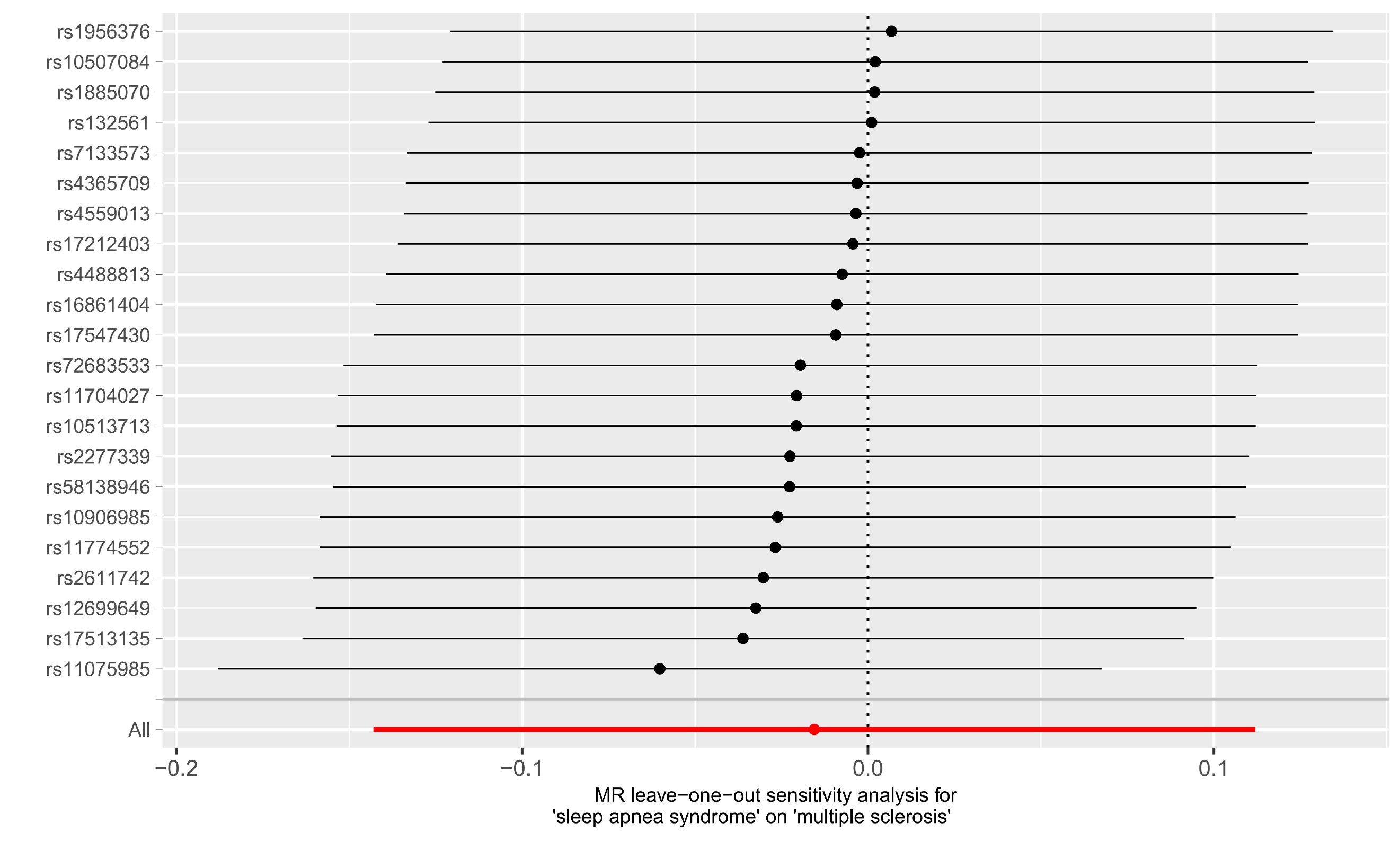


**Supplementary Figure 1F** Leave-one-out analysis illustrates causality analysis of sleep apnea syndrome on multiple sclerosis


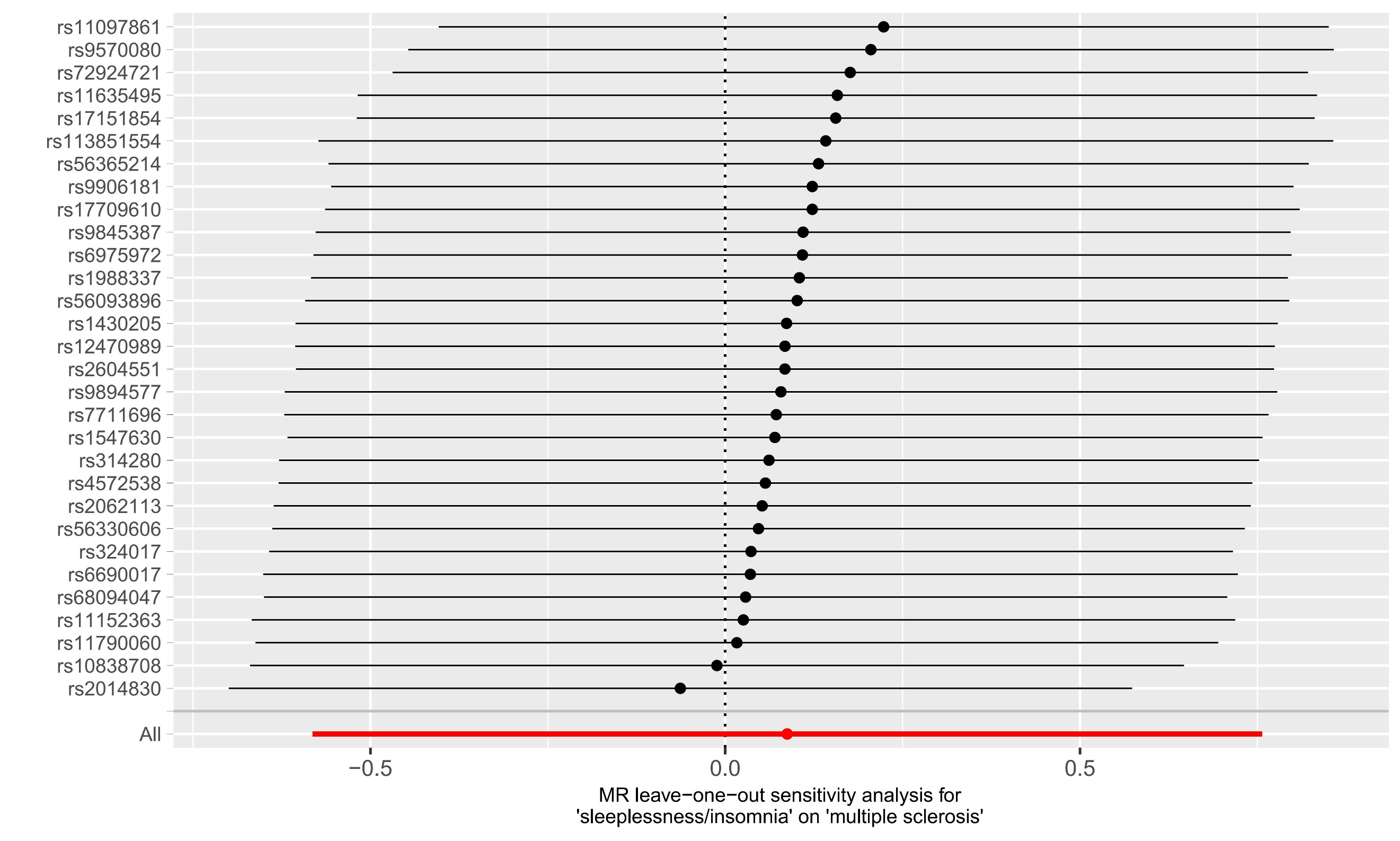


**Supplementary Figure 1G** Leave-one-out analysis illustrates causality analysis of sleeplessness/insomnia on multiple sclerosis


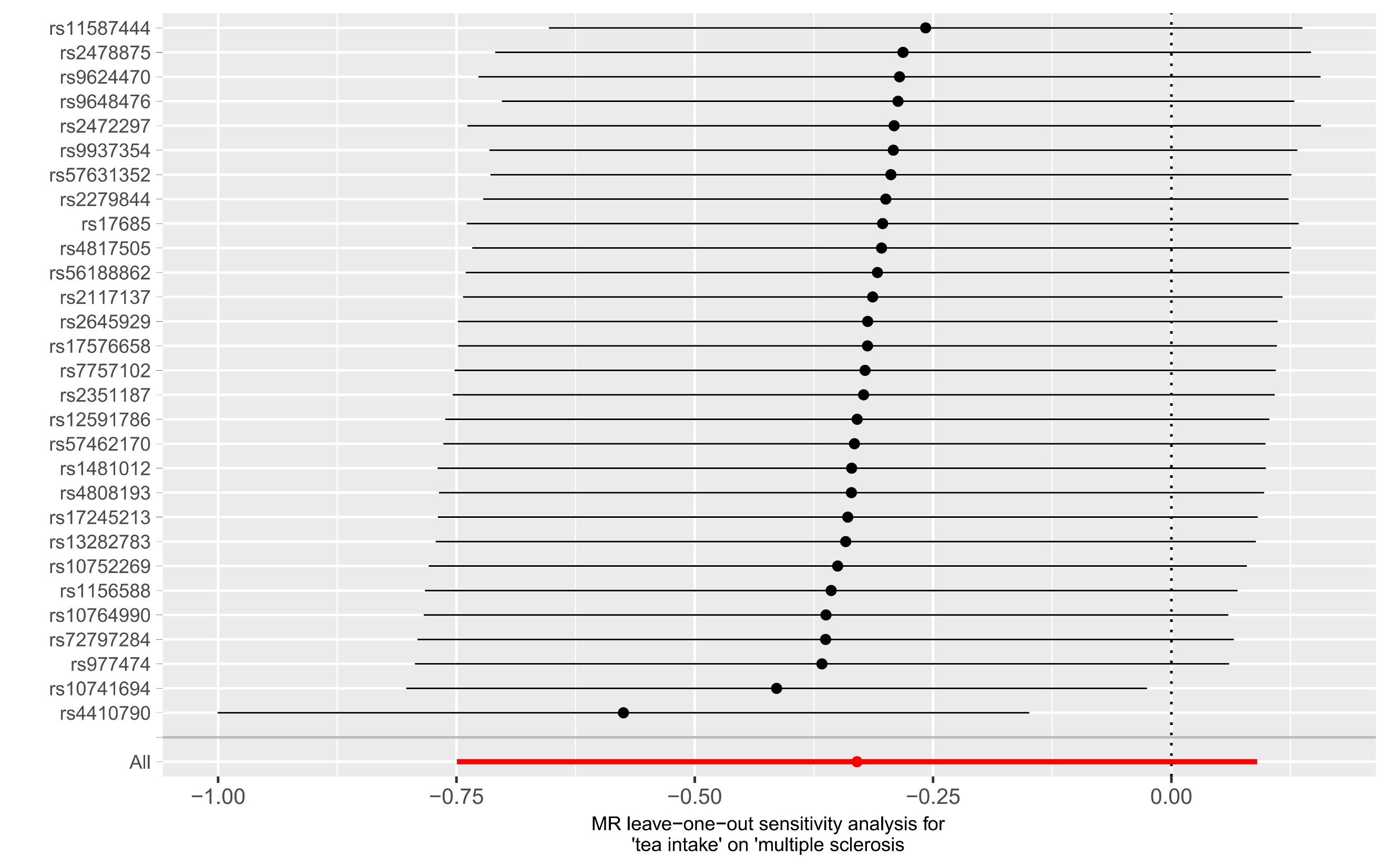


**Supplementary Figure 1H** Leave-one-out analysis illustrates causality analysis of tea intake on multiple sclerosis


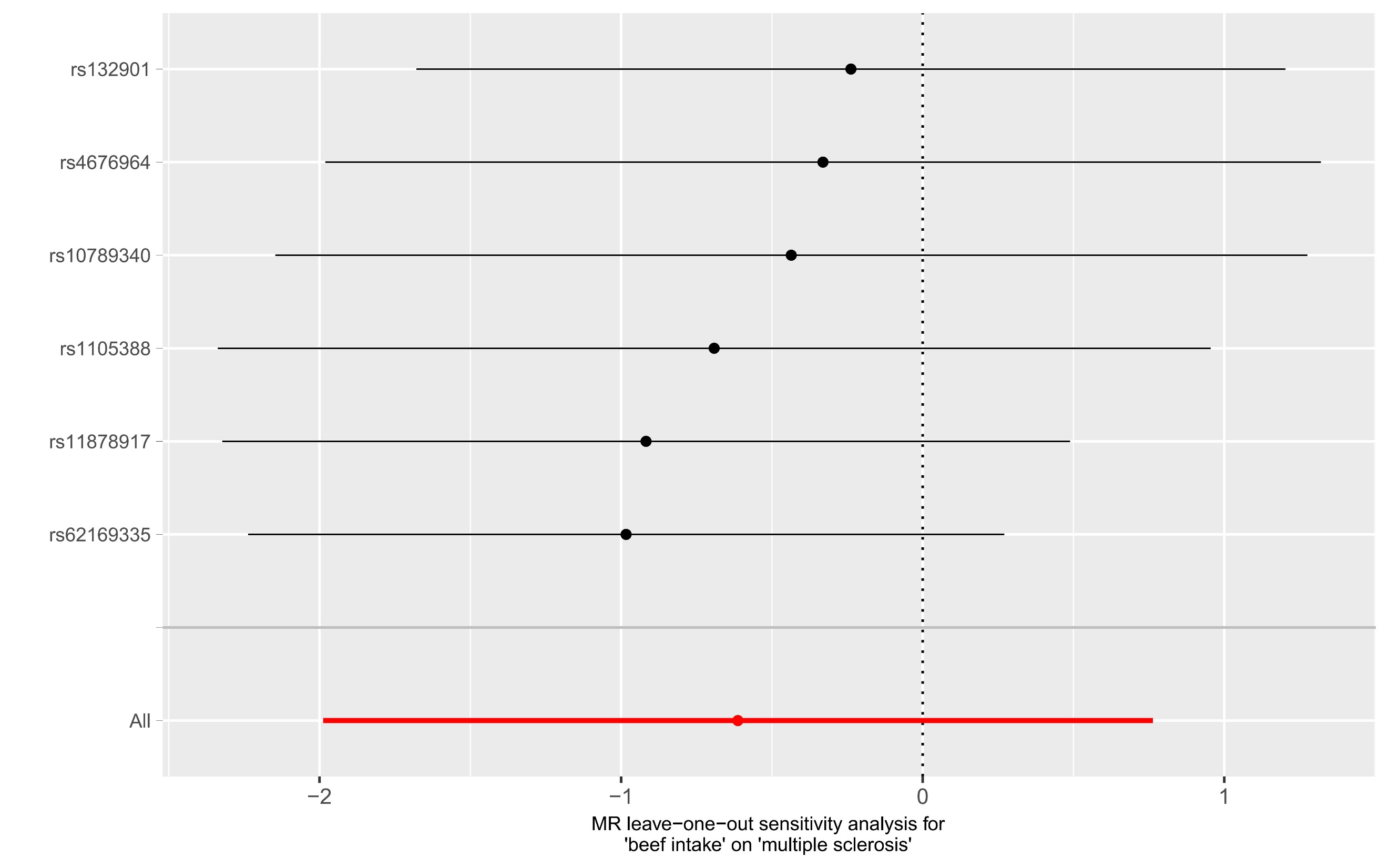


**Supplementary Figure 1I** Leave-one-out analysis illustrates causality analysis of beef intake on multiple sclerosis


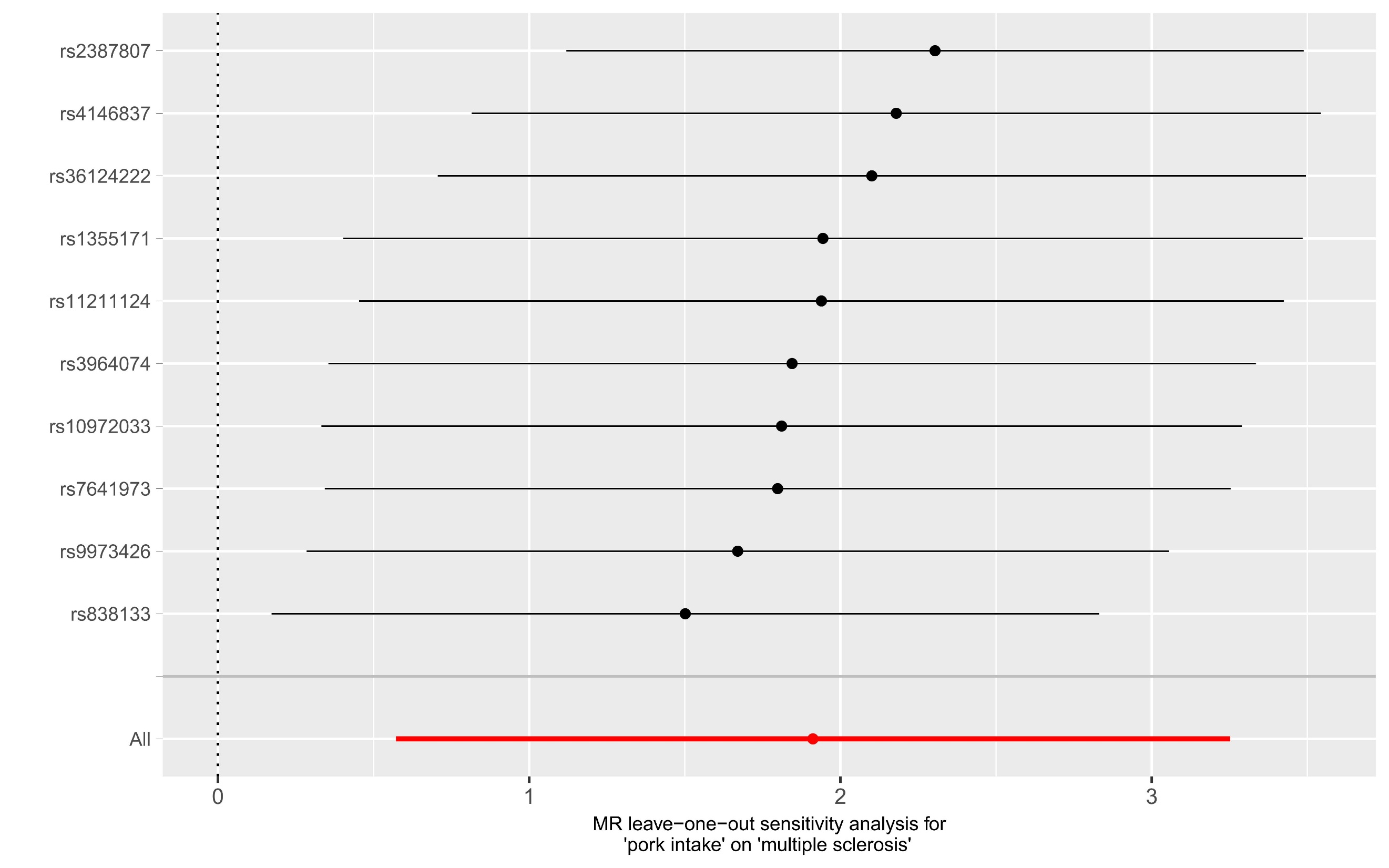


**Supplementary Figure 1J** Leave-one-out analysis illustrates causality analysis of pork intake on multiple sclerosis


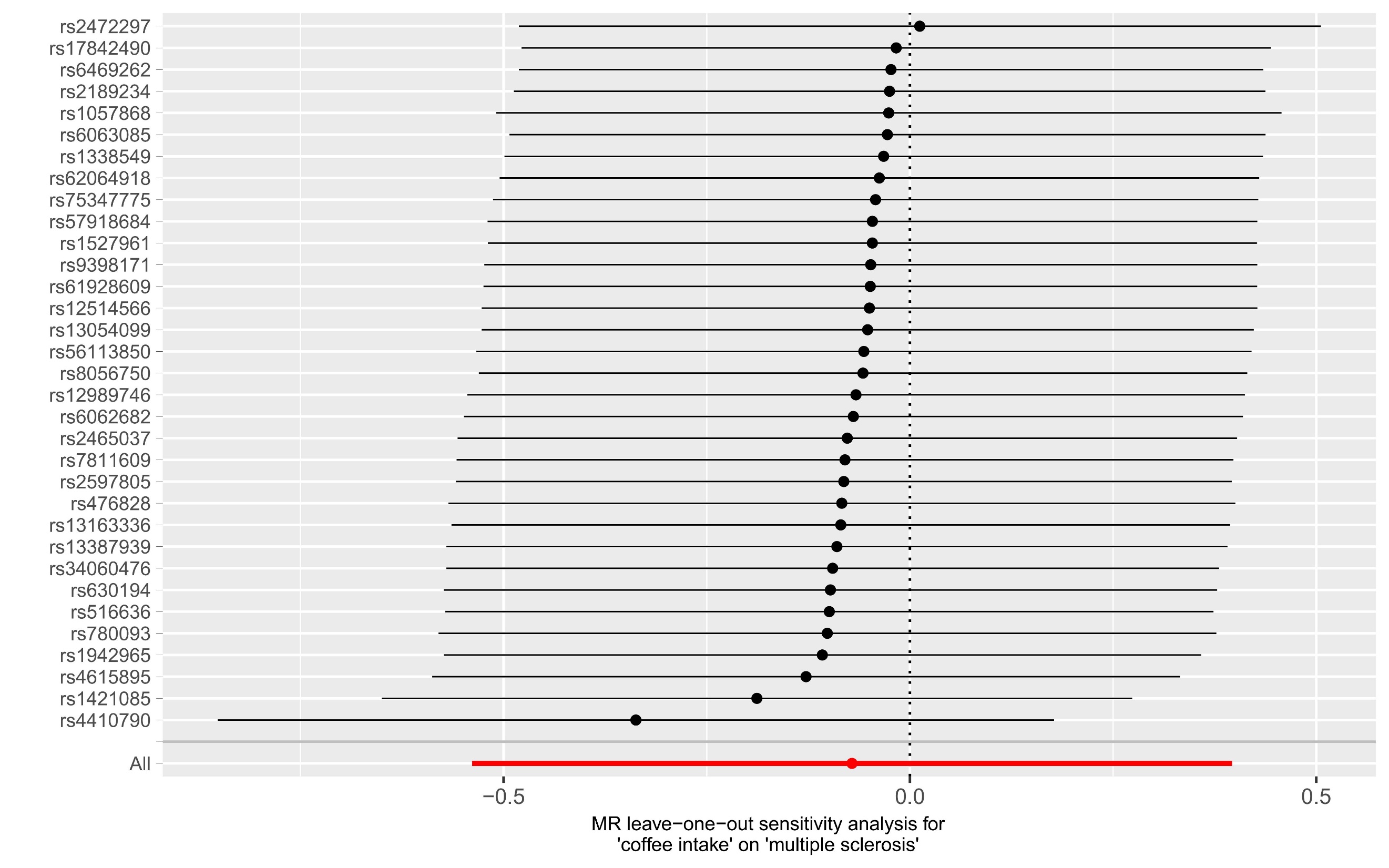


**Supplementary Figure 1K** Leave-one-out analysis illustrates causality analysis of coffee intake on multiple sclerosis


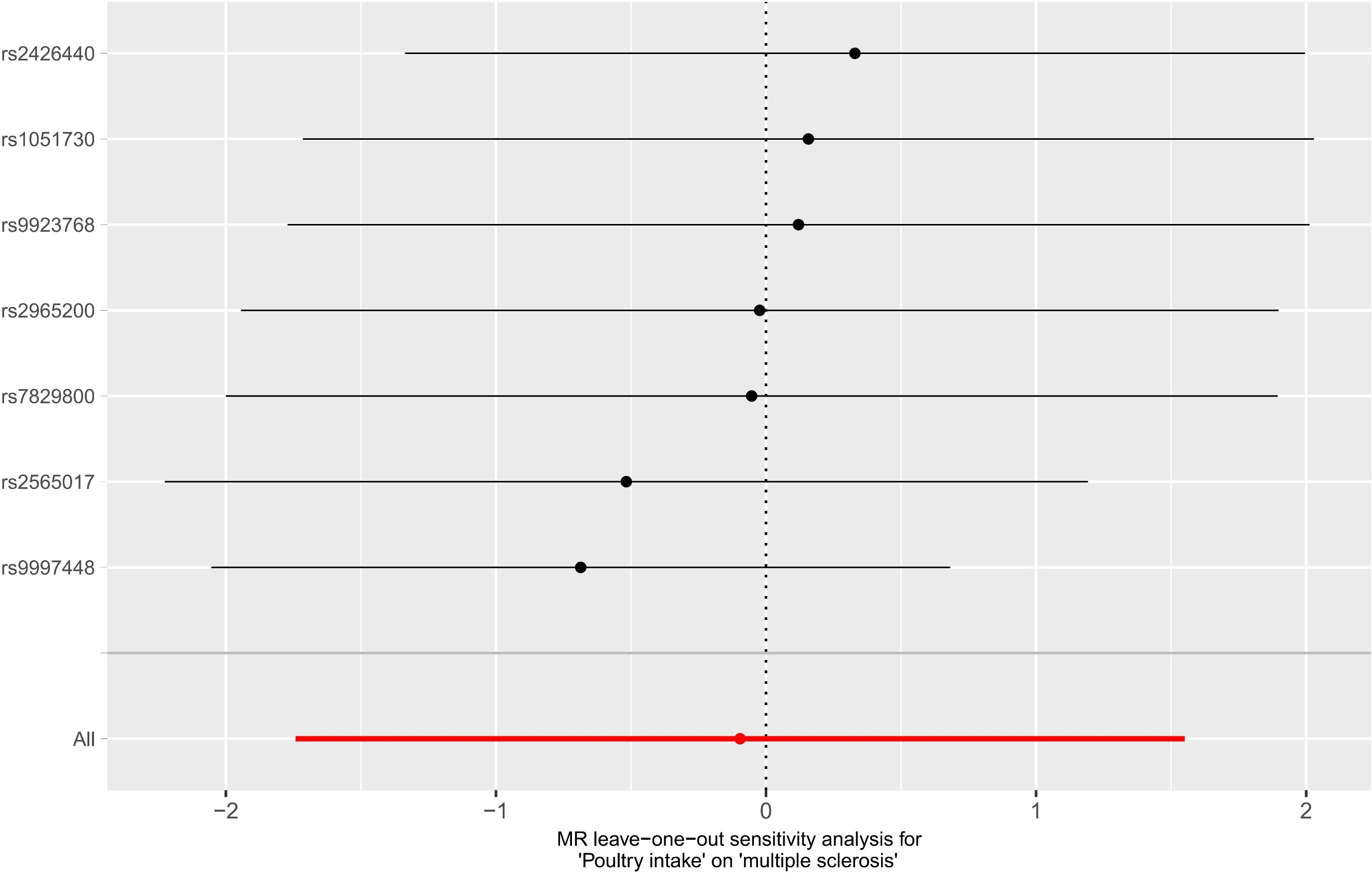


**Supplementary Figure 1L** Leave-one-out analysis illustrates causality analysis of poultry intake on multiple sclerosis


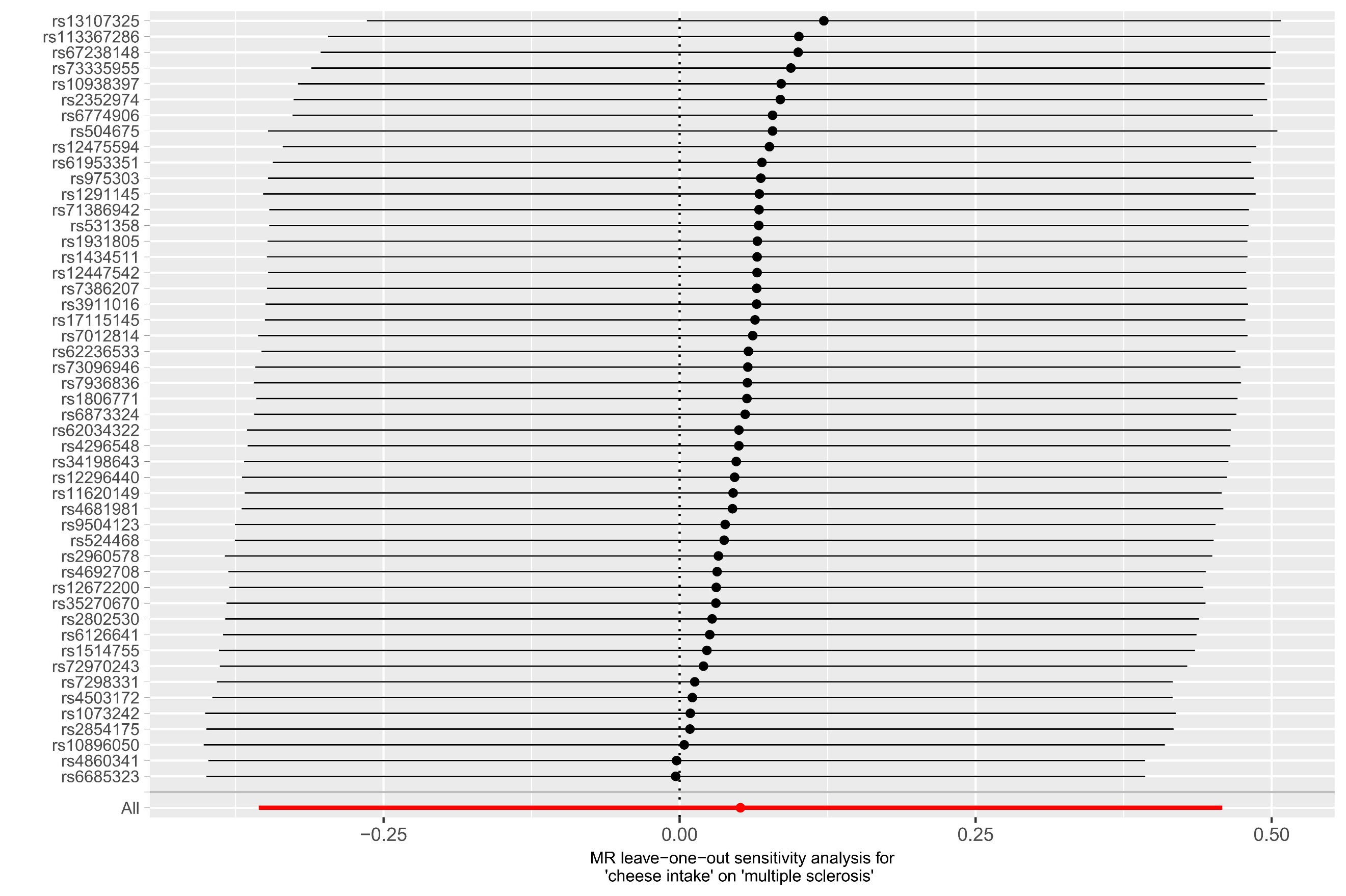


**Supplementary Figure 1M** Leave-one-out analysis illustrates causality analysis of cheese intake on multiple sclerosis


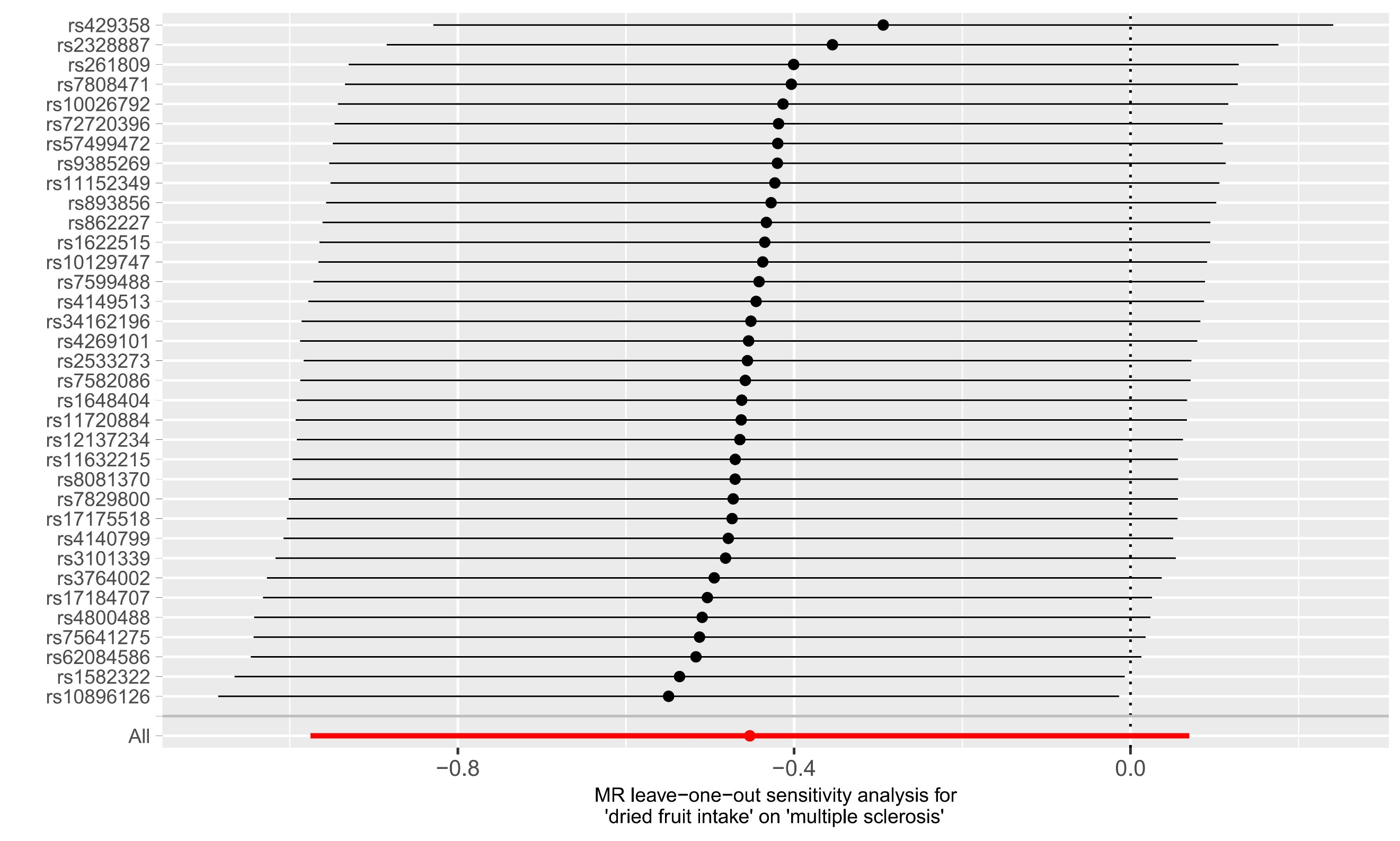


**Supplementary Figure 1N** Leave-one-out analysis illustrates causality analysis of dried fruit intake on multiple sclerosis


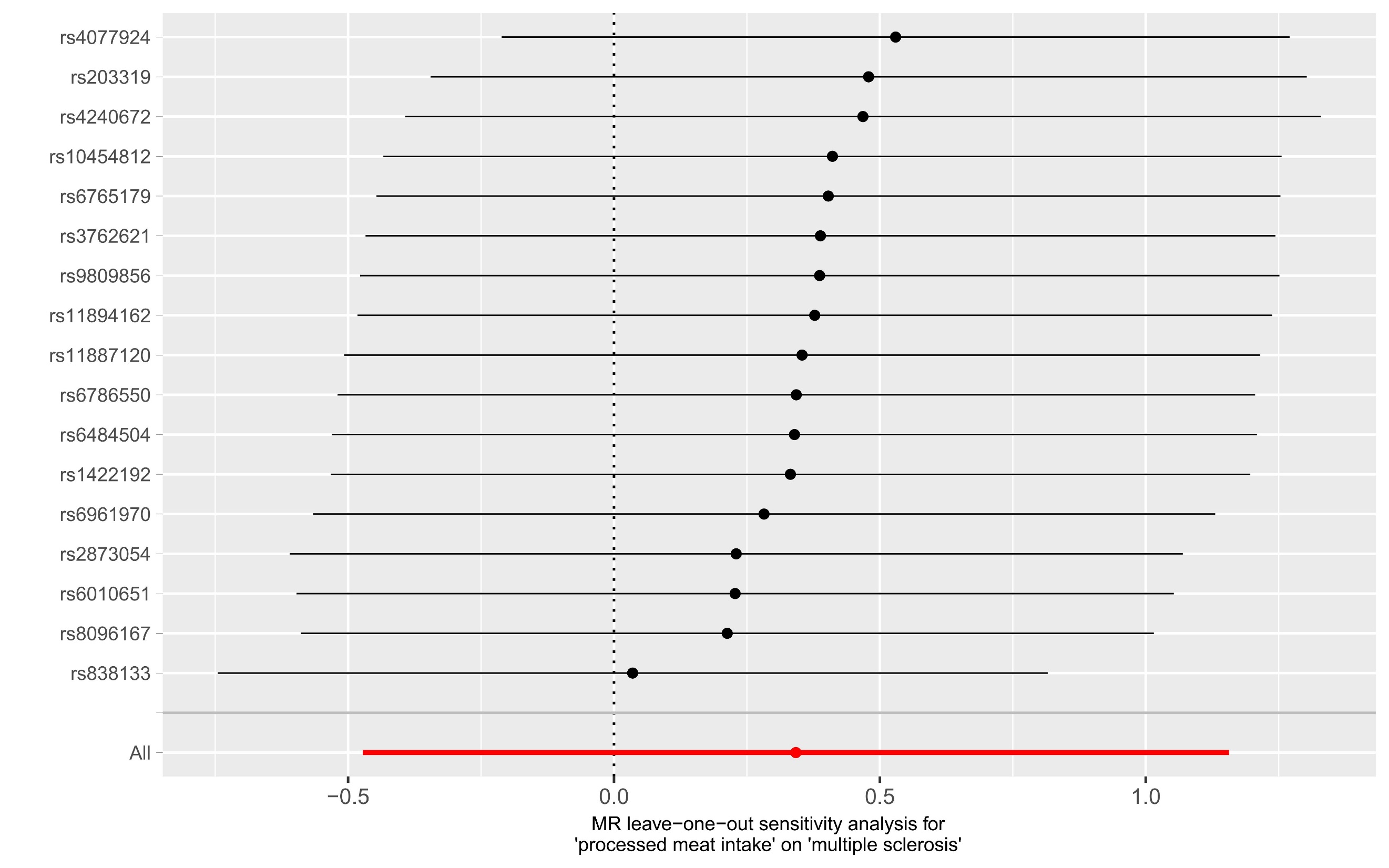


**Supplementary Figure 1O** Leave-one-out analysis illustrates causality analysis of processed meat intake on multiple sclerosis


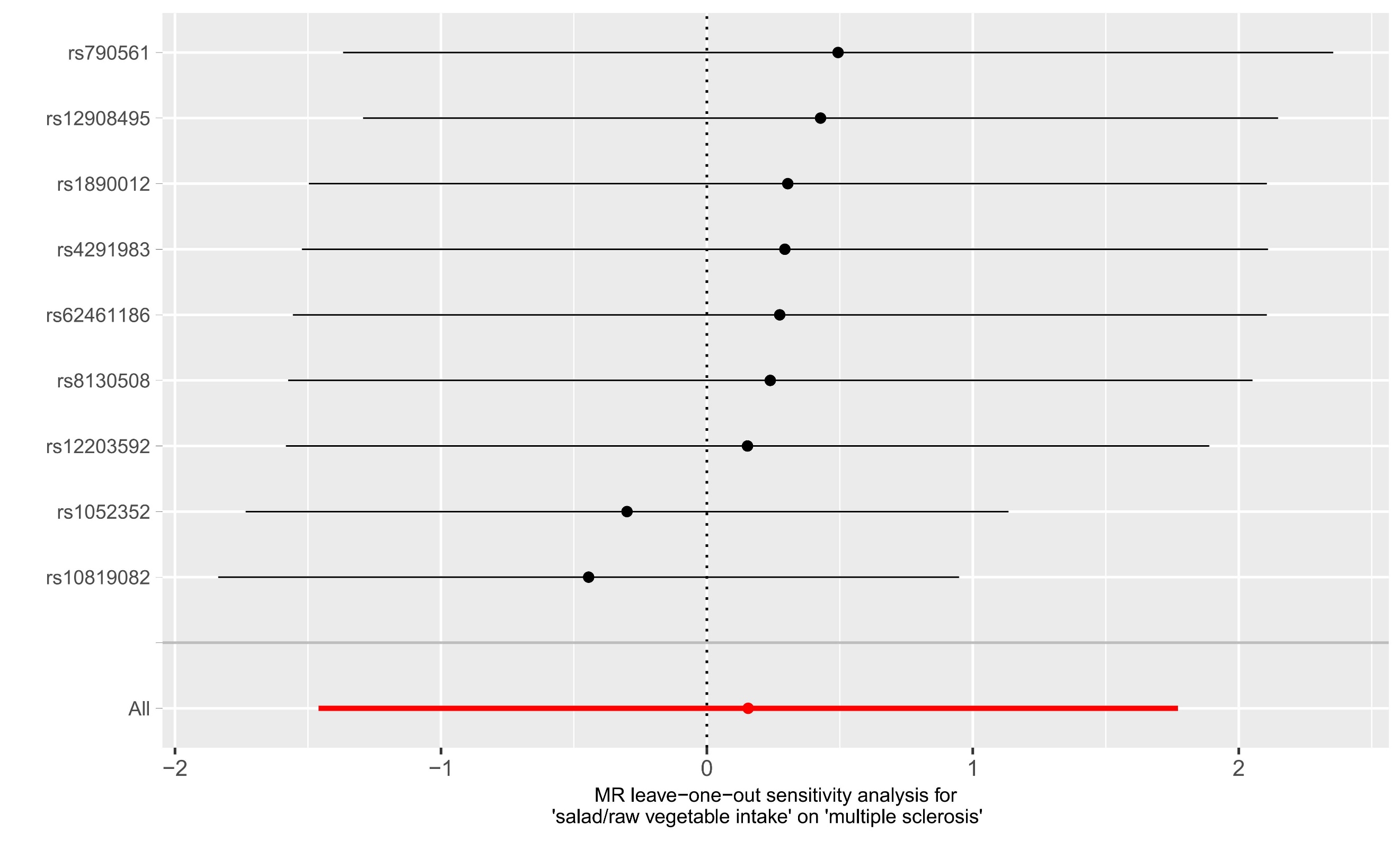


**Supplementary Figure 1P** Leave-one-out analysis illustrates causality analysis of salad/raw vegetable intake on multiple sclerosis


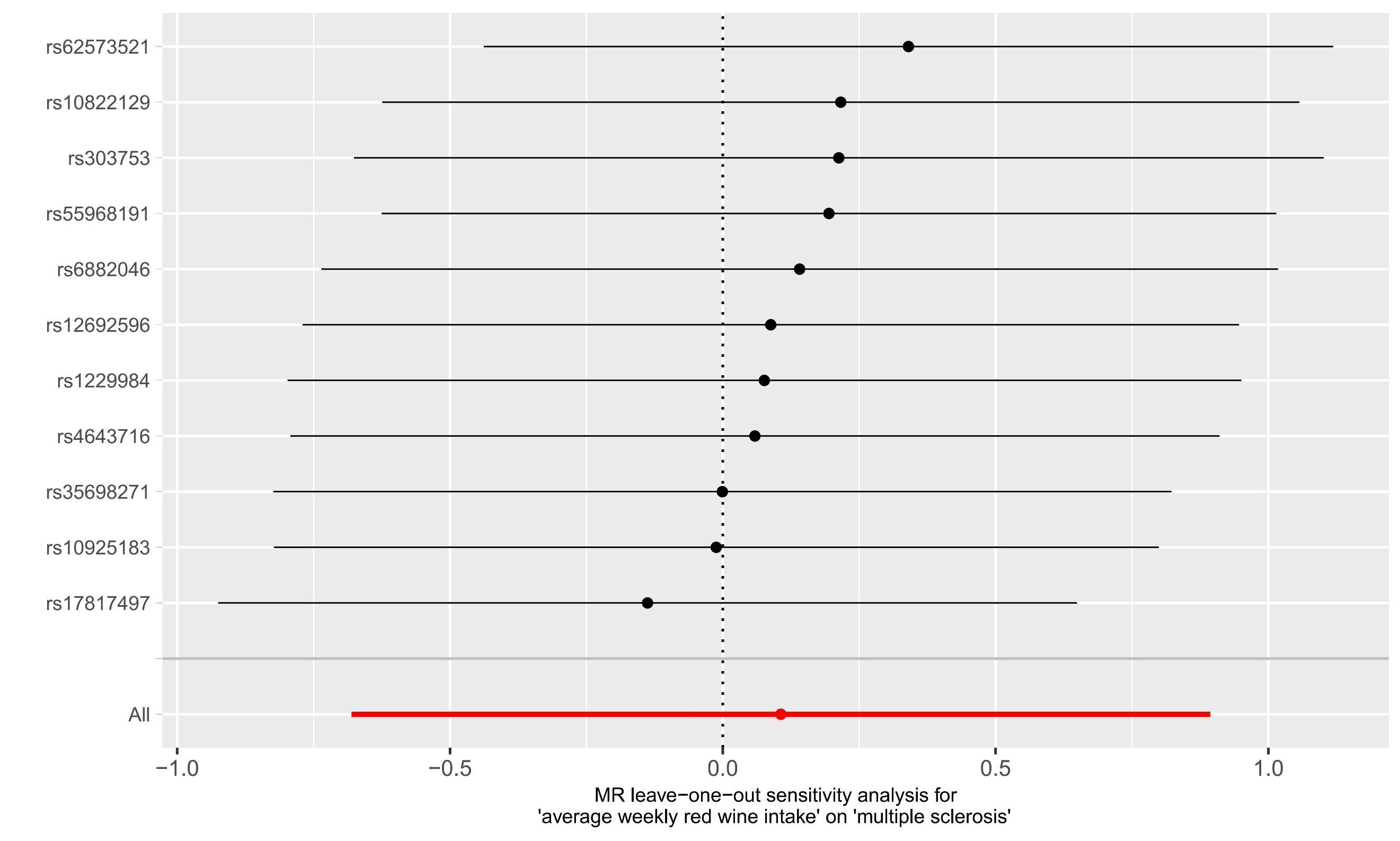


**Supplementary Figure 1Q** Leave-one-out analysis illustrates causality analysis of average weekly red wine intake on multiple sclerosis


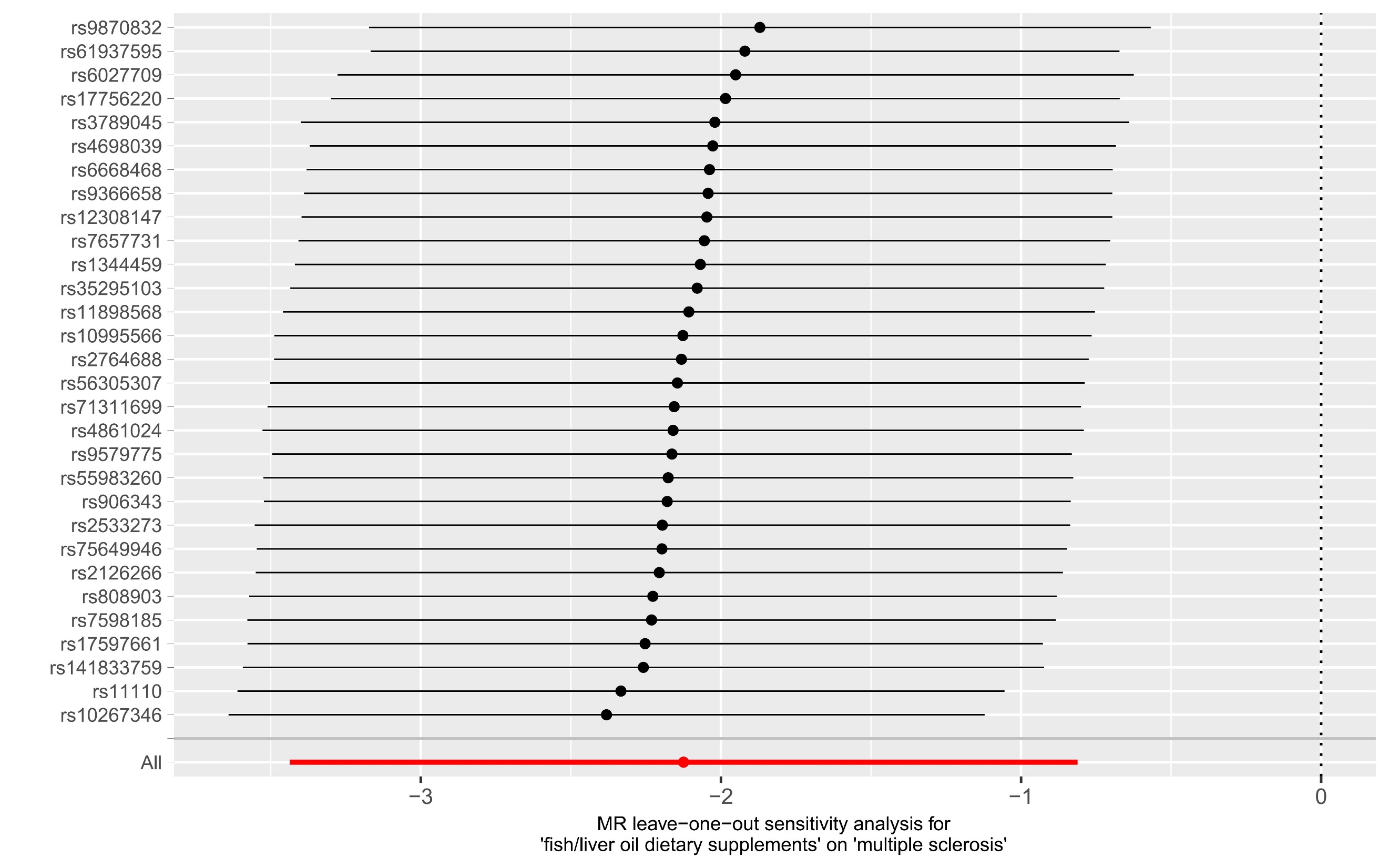


**Supplementary Figure 1R** Leave-one-out analysis illustrates causality analysis of fish/liver oil dietary supplements on multiple sclerosis


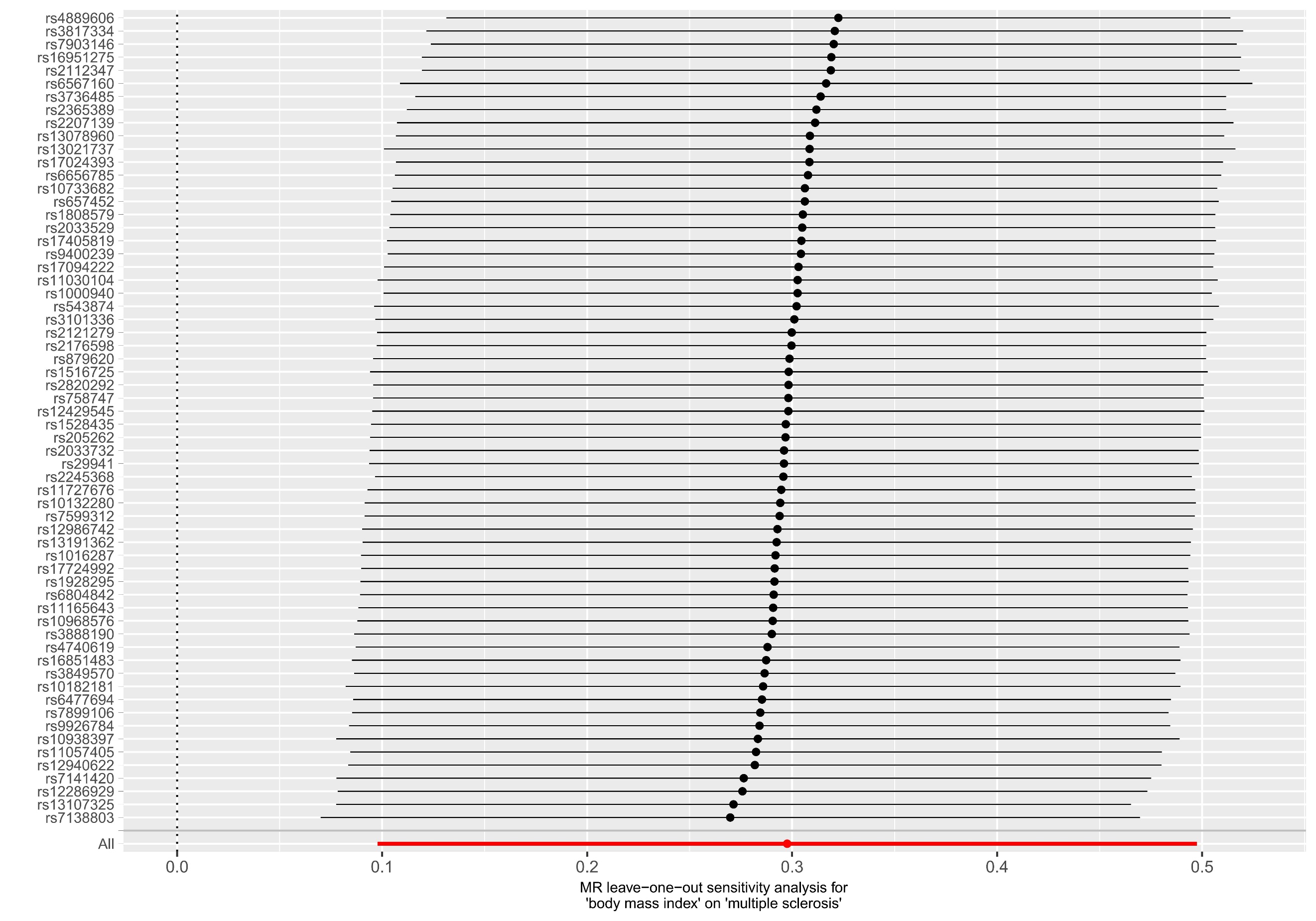


**Supplementary Figure 1S** Leave-one-out analysis illustrates causality analysis of body mass index on multiple sclerosis


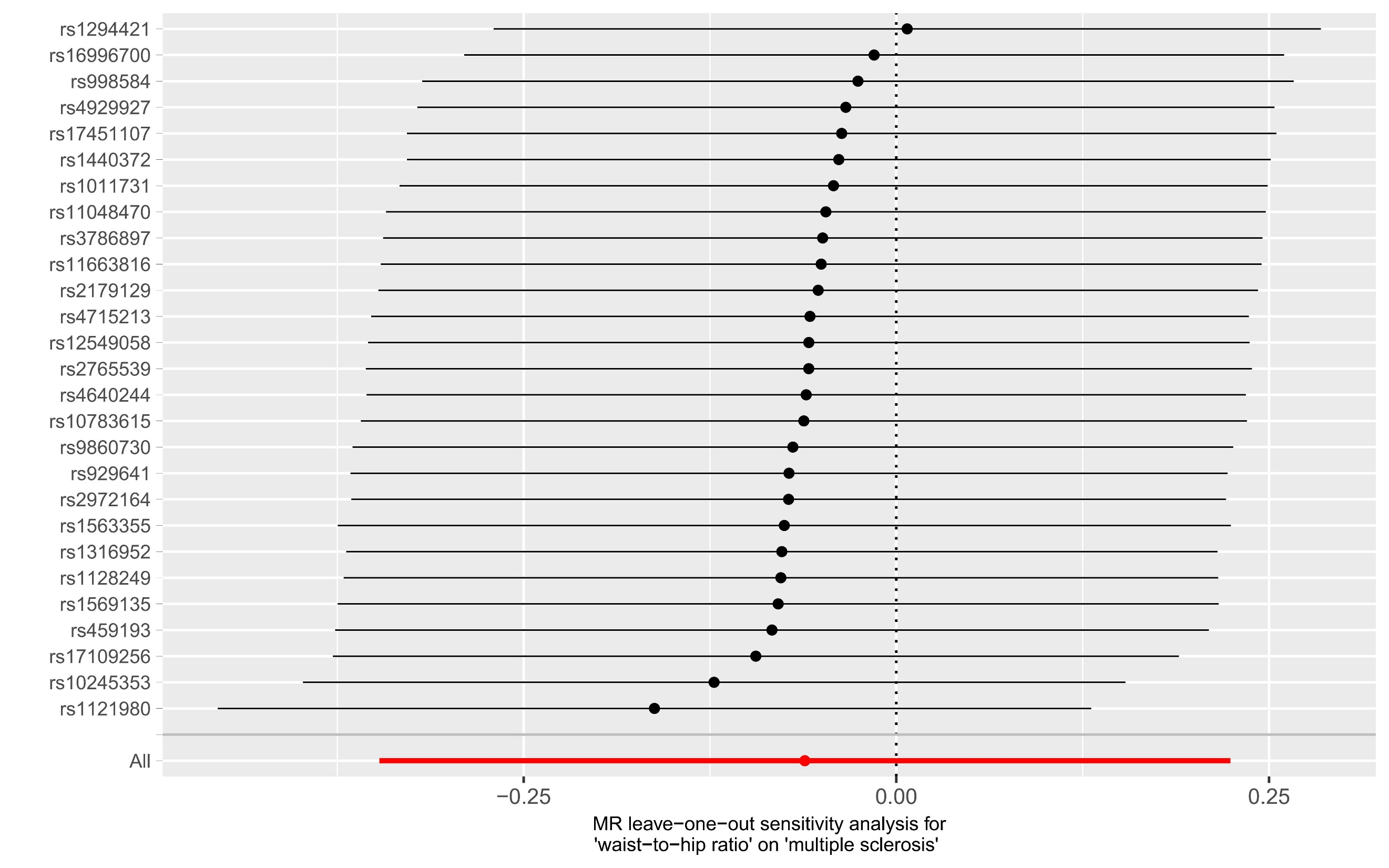


**Supplementary Figure 1T** Leave-one-out analysis illustrates causality analysis of waist-to-hip ratio on multiple sclerosis


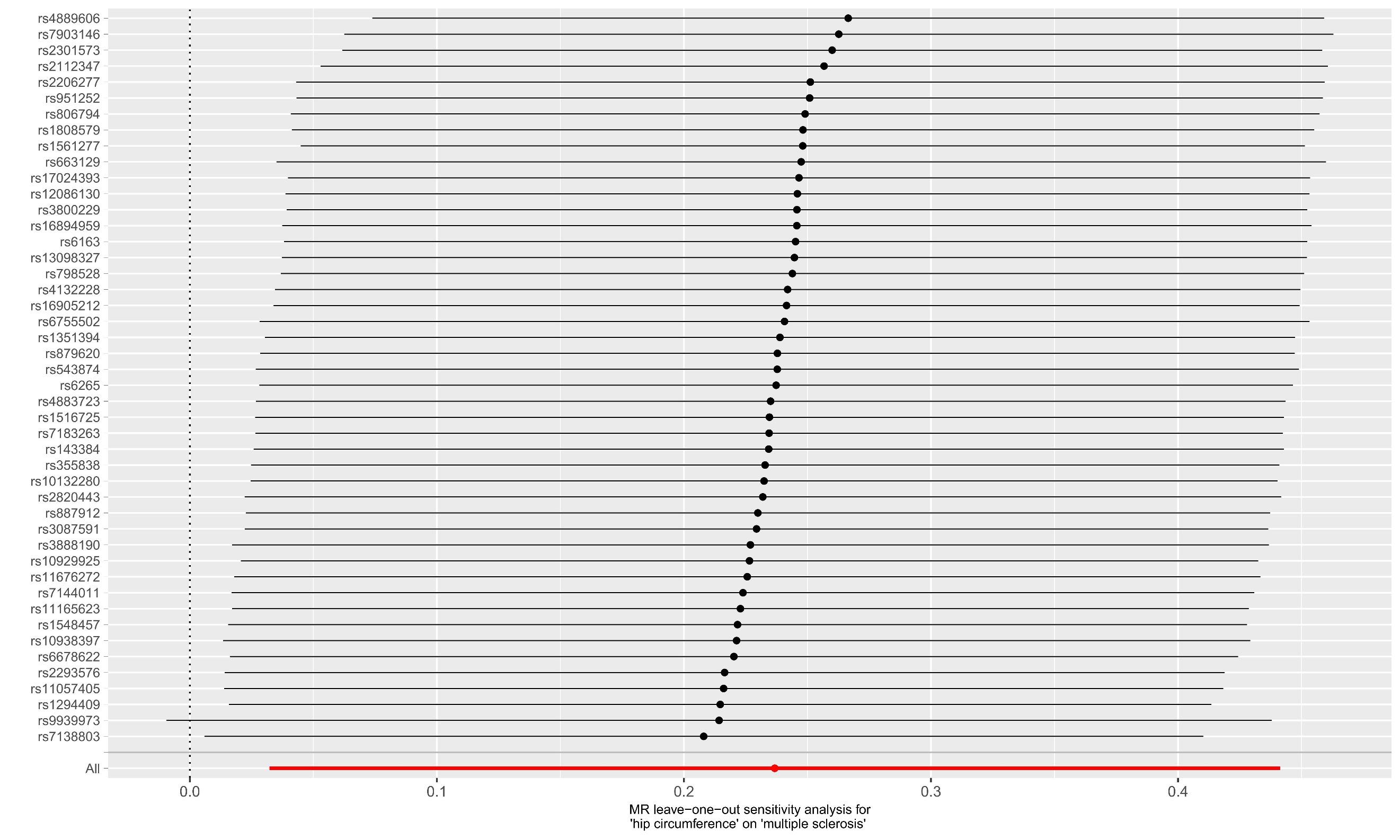


**Supplementary Figure 1U** Leave-one-out analysis illustrates causality analysis of hip circumference on multiple sclerosis


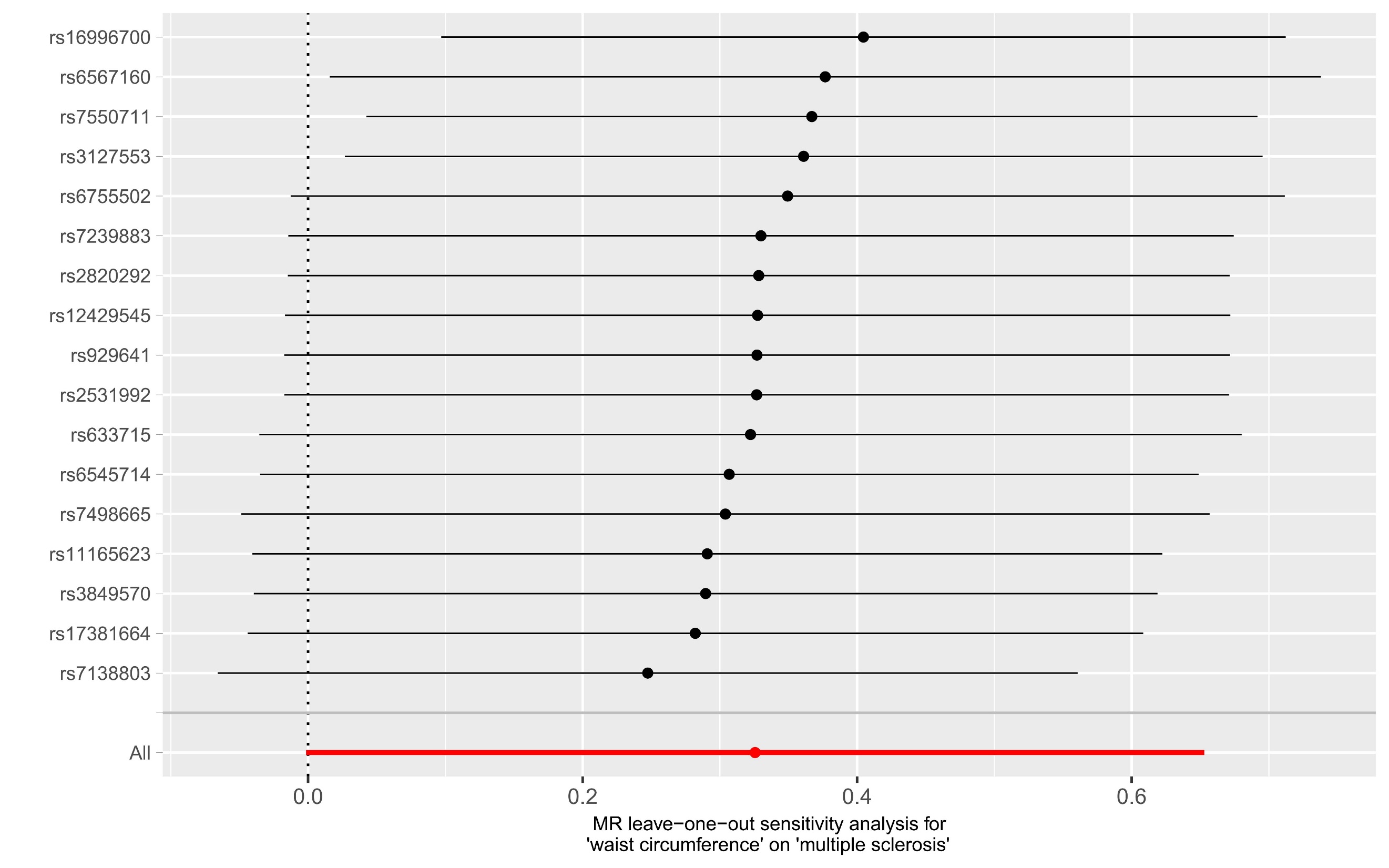


**Supplementary Figure 1V** Leave-one-out analysis illustrates causality analysis of waist circumference on multiple sclerosis


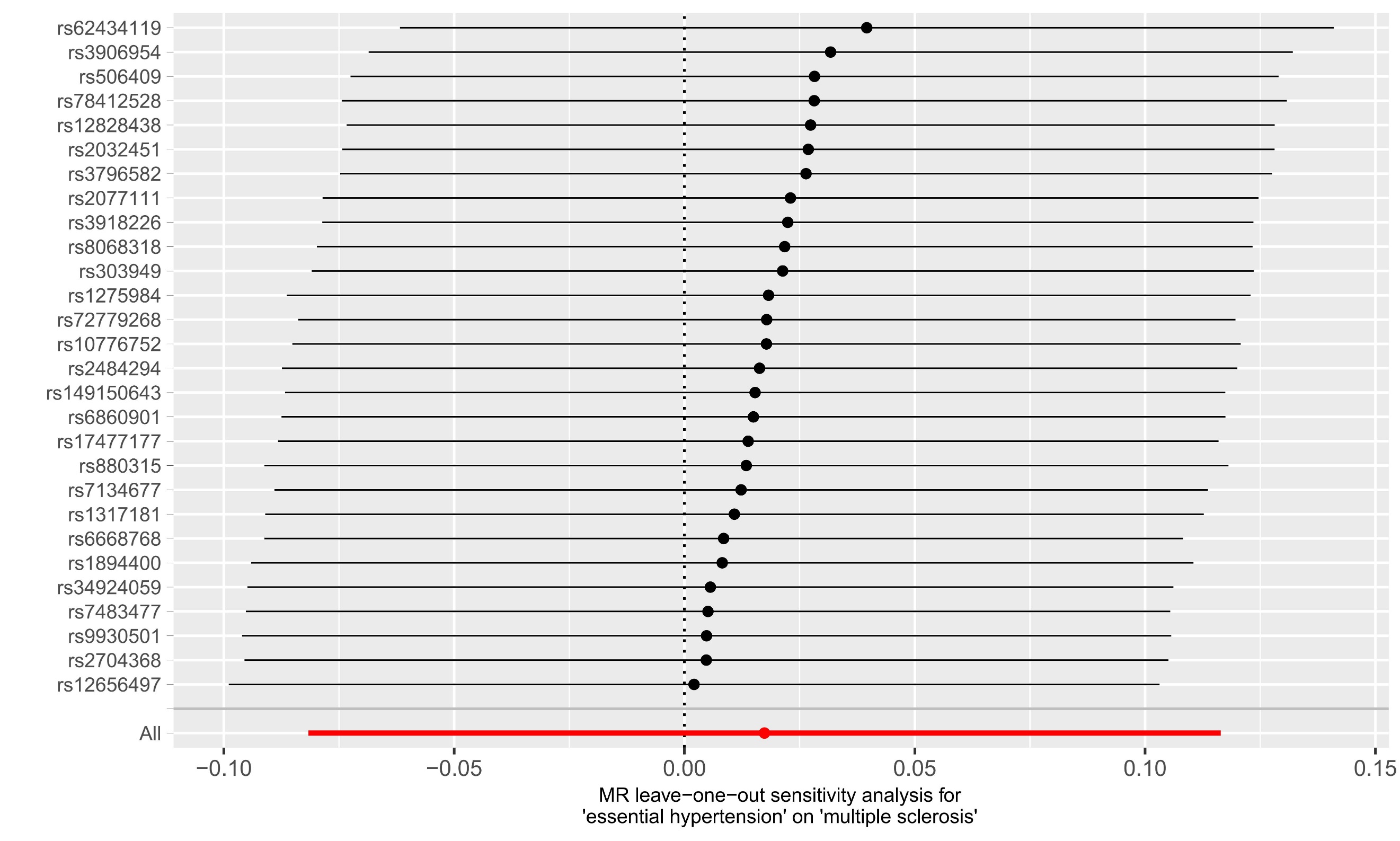


**Supplementary Figure 1W** Leave-one-out analysis illustrates causality analysis of essential hypertension on multiple sclerosis


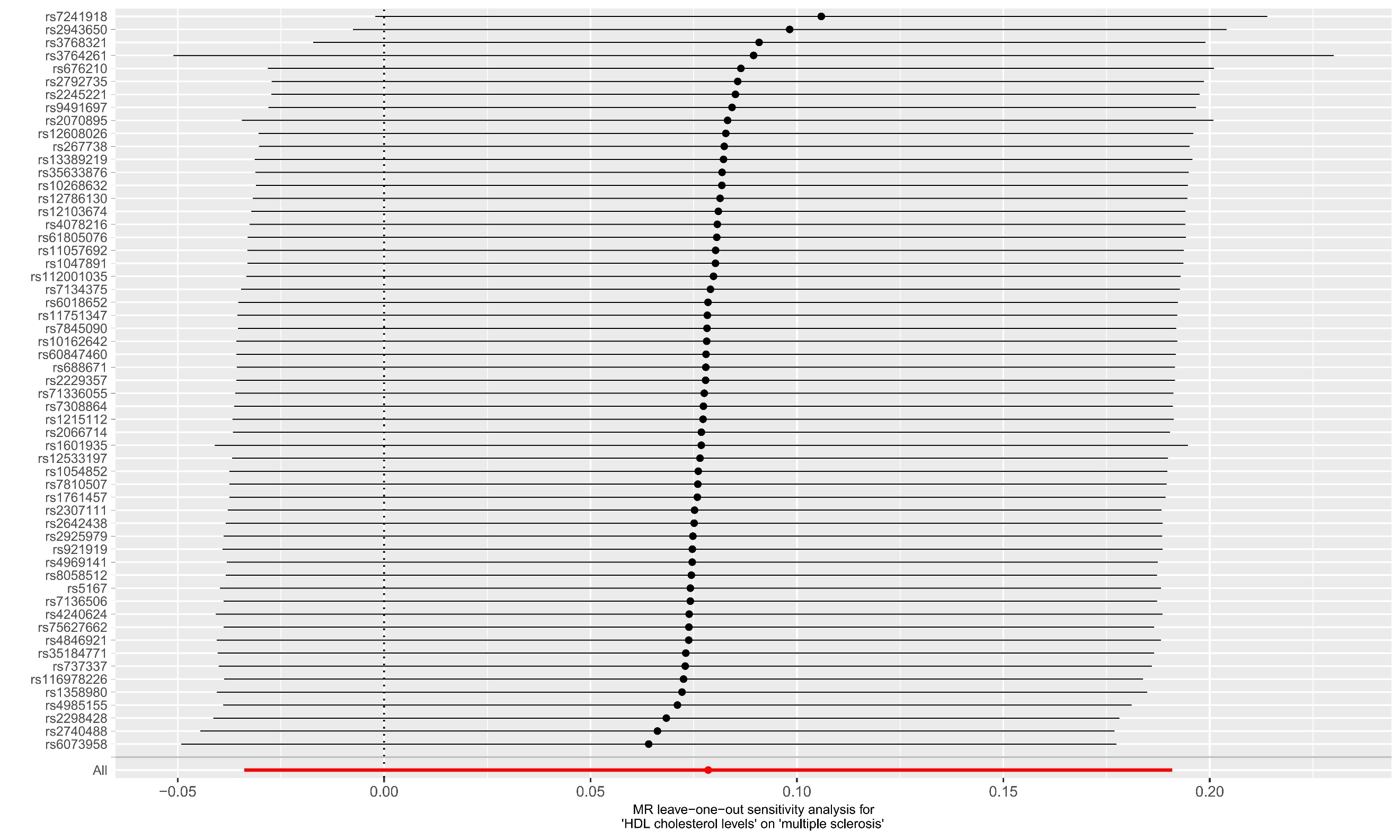


**Supplementary Figure 1X** Leave-one-out analysis illustrates causality analysis of HDL cholesterol levels on multiple sclerosis


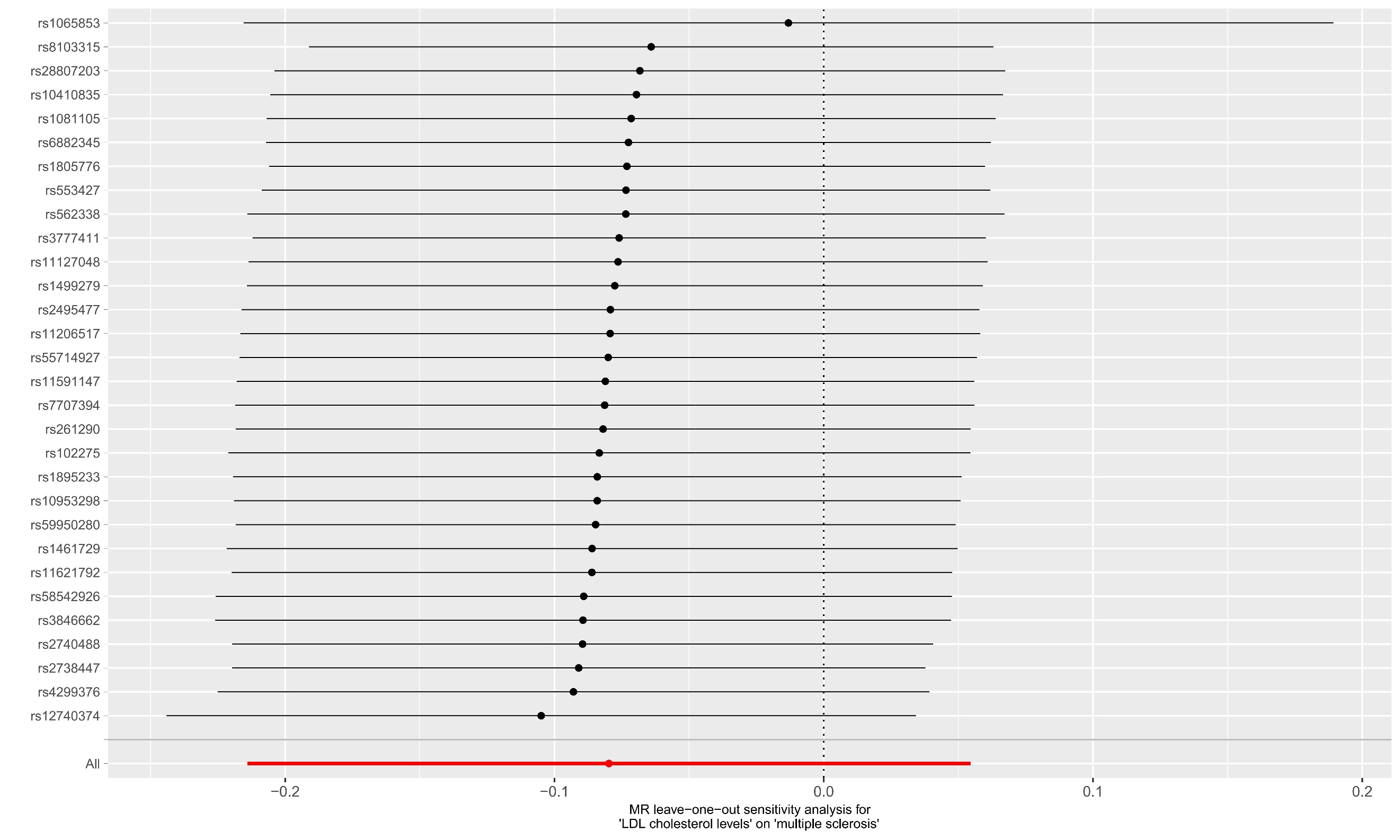


**Supplementary Figure 1Y** Leave-one-out analysis illustrates causality analysis of LDL cholesterol levels on multiple sclerosis


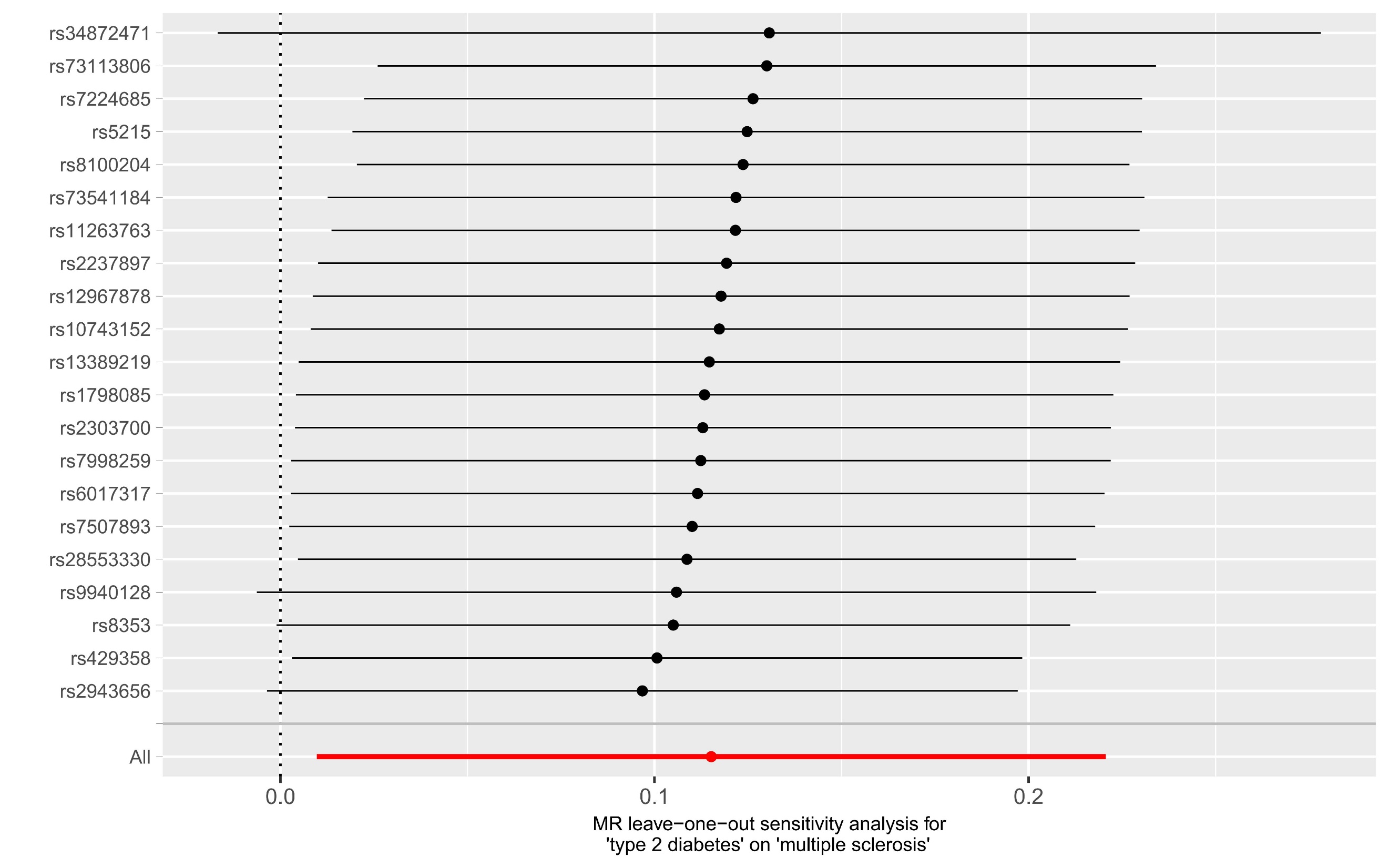


**Supplementary Figure 1Z** Leave-one-out analysis illustrates causality analysis of type 2 diabetes on multiple sclerosis


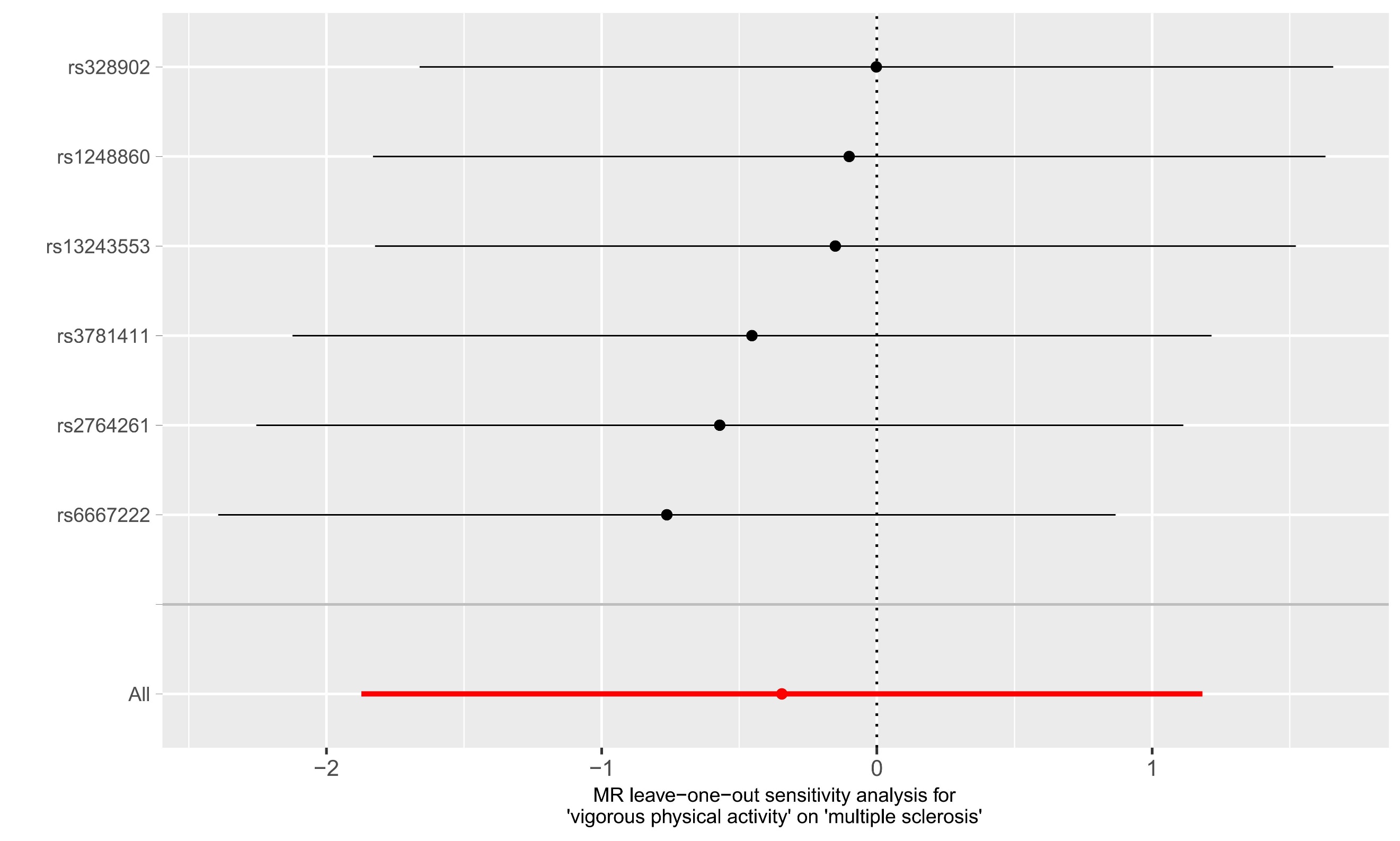


**Supplementary Figure 1AA** Leave-one-out analysis illustrates causality analysis of vigorous physical activity on multiple sclerosis


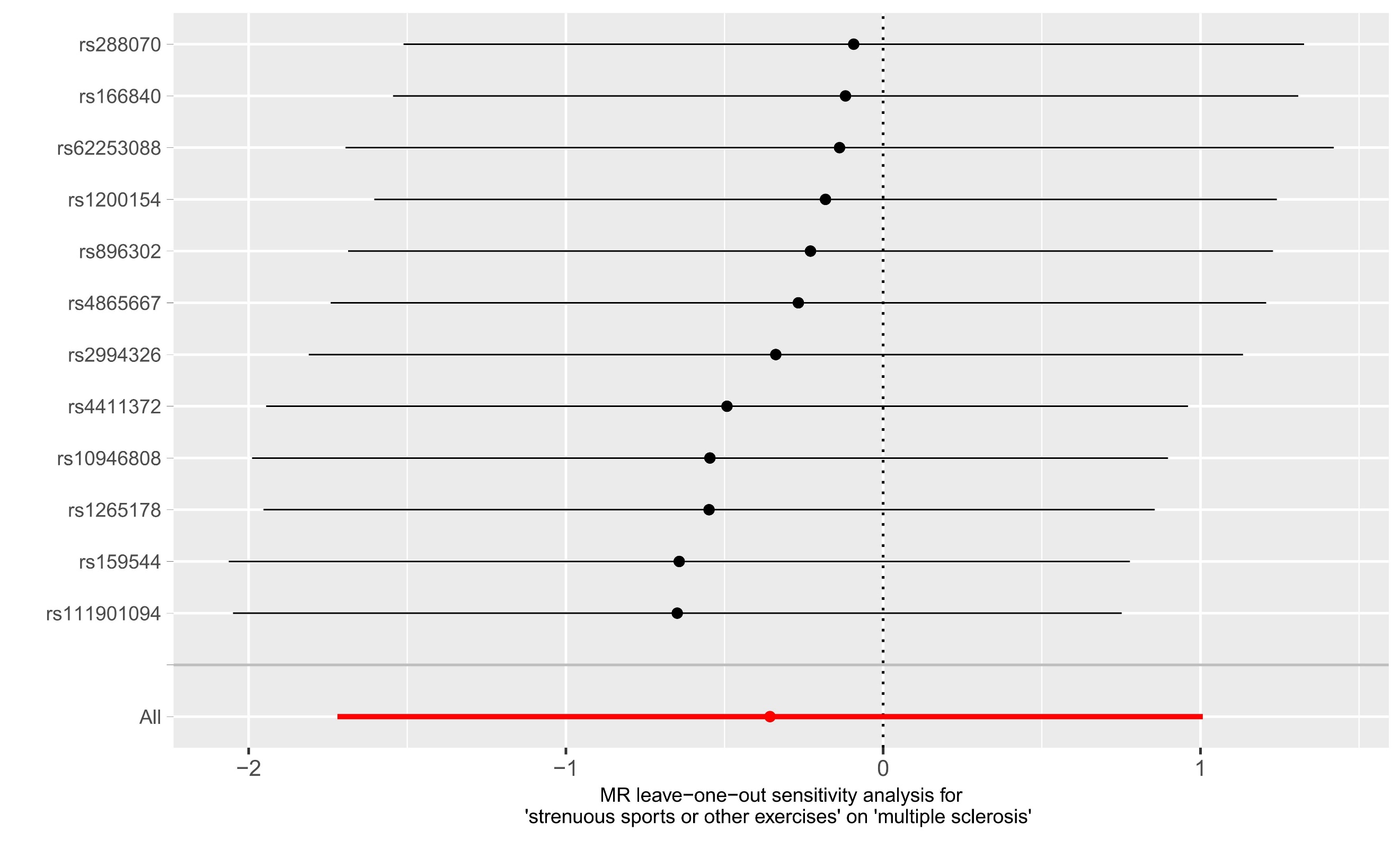


**Supplementary Figure 1BB** Leave-one-out analysis illustrates causality analysis of strenuous sports or other exercises on multiple sclerosis


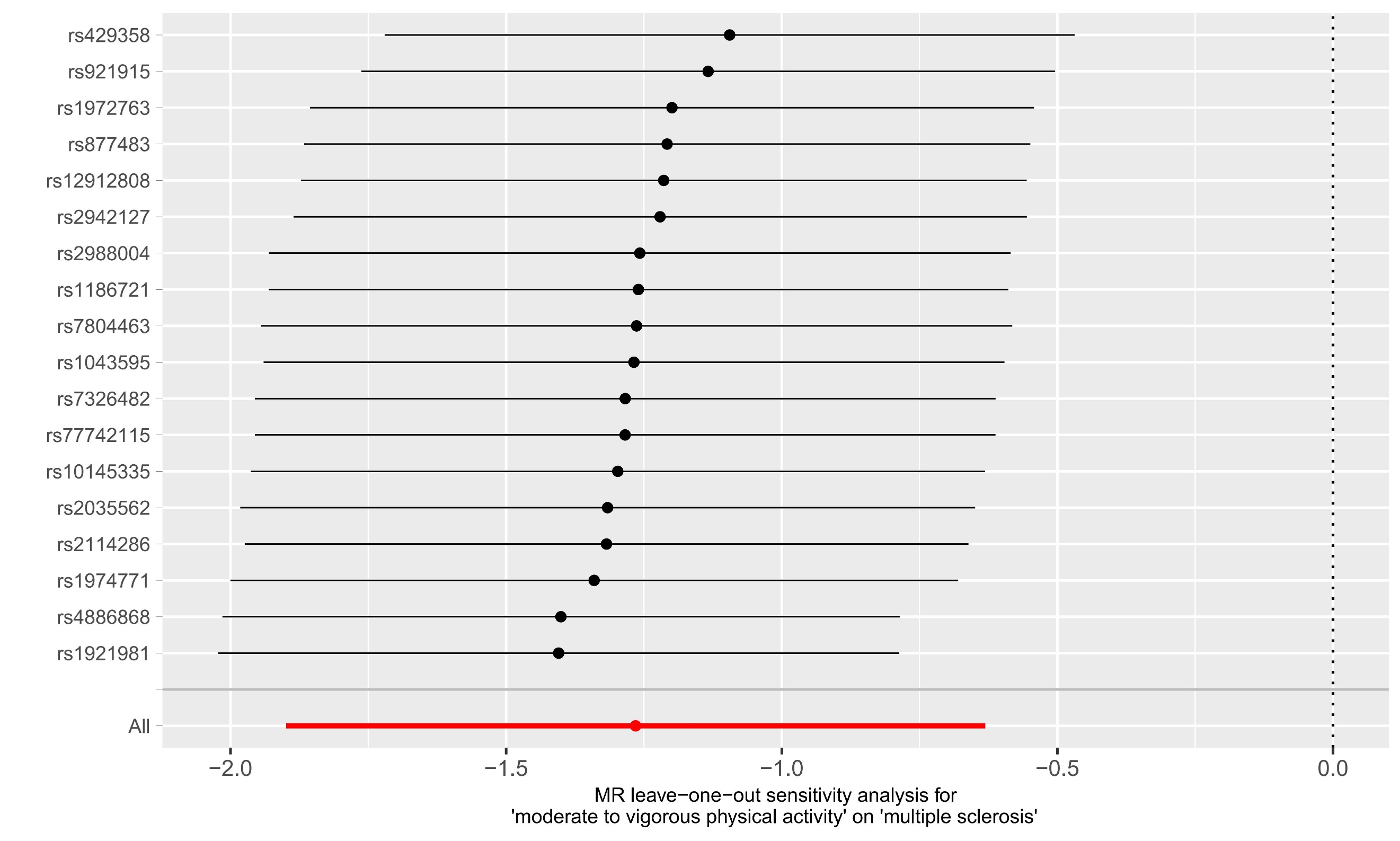


**Supplementary Figure 1CC** Leave-one-out analysis illustrates causality analysis of moderate to vigorous physical activity on multiple sclerosis
