# Supplementary material for: Associations Between Dietary Factors, Metabolic Factors, Sleep Disorders, Physical Activity, and the Risk of Multiple Sclerosis: A Univariable and Multivariable Mendelian Randomization Study
Source: Food Sci Nutr. 2025 Sep 11;13(9):e70905. doi: 10.1002/fsn3.70905 (PMC12424063; doi:10.1002/fsn3.70905)
Supplement: Supplementary file 2 — Tables S1–S4: fsn370905‐sup‐0002‐TablesS1‐S4.docx. [file FSN3-13-e70905-s002.docx]

**Associations between** **dietary factors, metabolic factors, sleep disorders, physical activity, and the risk of multiple sclerosis: A univariable and multivariable Mendelian Randomization study**

**Supplementary Table 1** STROBE-MR checklist [Page 2-5]

**Supplementary Table 2** Single nucleotide polymorphisms (SNPs) were used as instrumental variables (IVs) from sleep disorders, dietary factors, metabolic factors and physical activity [Page 6-47]

**Supplementary Table 3**. Specific information for univariable MR analysis of sleep disorders, dietary factors, metabolic factors and physical activity as exposure and multiple sclerosis as outcome [Page 48-53]

**Supplementary Table 4** Specific information for multivariable MR analysis of sleep disorders, dietary factors, metabolic factors and physical activity as exposure and multiple sclerosis as outcome [Page 54-55]

**Supplementary Table 1** STROBE-MR checklist

| **No.** | **Section** | **Checklist item** | **Position** |
| --- | --- | --- | --- |
| 1 | Title and abstract | Indicate MR as the study’s design in the title and the abstract as a main purpose of the study | Title, Abstract |
| 2 | Background | Explain the scientific background and rationale for the reported study. Explain the exposure and a plausible potential causal relationship between exposure and outcome. Justify why MR is a helpful method to address the study question. | Introduction: paragraphs 1-3 (page 2-3) |
| 3 | Objectives | State specific objectives clearly, including prespecified causal hypotheses. State that MR is a method that intends to estimate causal effects. | Introduction: paragraphs 3 (page 3) |
| 4a | Study design and data sourses | Setting: Describe the study design (two-sample MR) and the underlying population . Describe the setting, locations, and relevant dates, including periods of recruitment, exposure, follow-up, and data collection. | Materials and Methods: study design and data sources |
| 4b |  | Participants: Report the eligibility criteria and the sources and methods of selection of participants. Report the sample size and whether any power or sample size calculations were carried out prior to the main analysis. | Materials and Methods: data sources and selection of instrumental variables (IVs) |
| 4c |  | Describe measurement, quality control, and selection of genetic variants. | Materials and Methods: data sources and selection of instrumental variables (IVs)-selection of instrumental variables in 29 distinct traits |
| 4d |  | For each exposure, outcome, and other relevant variables, describe methods of assessment and diagnostic criteria for diseases. | Materials and Methods: data sources |
| 4e |  | Provide details of ethics committee approval and participant informed consent, if relevant. | Not applicable |
| 5 | Assumptions | Explicitly state the 3 core instrumental variable (IV) assumptions for the main analysis (relevance, independence, and exclusion restriction), as well assumptions for any additional or sensitivity analysis. | Materials and Methods: study design and statistical analysis |
| 6a | Statistical methods: | Describe how quantitative variables were handled in the analyses. | Materials and Methods: instrumental variables (IVs) and statistical analysis |
| 6b |  | Describe how genetic variants were handled in the analyses and, if applicable, how their weights were selected |  |
| 6c |  | Describe the MR estimator and related statistics. Detail the included covariates and, in case of 2-sample MR, whether the same covariate set was used for adjustment in the 2 samples. |  |
| 6d |  | Explain how missing data were addressed. | N/A |
| 6e |  | Indicate how multiple testing was addressed (false discovery rate method) | N/A |
| 7 | Assessment of assumptions | Describe any methods or prior knowledge used to assess the assumptions or justify their validity | N/A |
| 8 | Sensitivity analyses and additional analyses | Describe any sensitivity analyses or additional analyses performed (eg, comparison of effect estimates from different approaches, independent replication, bias analytic techniques, validation of instruments, simulations). | Materials and Methods: statistical analysis |
| 9a | Software and preregistration | Name statistical software and package(s), including version and settings used. | Materials and Methods: statistical analysis |
| 9b |  | State whether the study protocol and details were preregistered (as well as when and where). | N/A |
| 10a | Descriptive data | Report the numbers of individuals at each stage of included studies and reasons for exclusion. Use of a flow diagram. | Methods: Study design , Exposure GWAS and Outcome GWAS |
| 10b |  | Report summary statistics for phenotypic exposure , outcomes, and other relevant variables. | Table 1 |
| 10c |  | If the data sources include meta-analyses of previous studies, provide the assessments of heterogeneity and pleiotropy across these studies. | Table 2 |
| 10d |  | For 2-sample MR: i. Provide justification of the similarity of the genetic variant-exposure associations between the exposure and outcome samples. ii. Provide information on the number of individuals who overlap between the exposure and outcome studies. | Results, Supplementary Table 1 |
| 11a | Main results | Report the associations between genetic variant and exposure and between genetic variant and outcome, preferably on an interpretable scale. | Results, Supplementary Table 1 |
| 11b |  | Report MR estimates of the relationship between exposure and outcome and the measures of uncertainty from the MR analysis, on an interpretable scale, such as odds ratio or relative risk per SD difference. | Results, Supplementary Table 2, 3 and 4 |
| 11c |  | If relevant, consider translating estimates of relative risk into absolute risk for a meaningful time period. | N/A |
| 11d |  | Consider plots to visualize results (eg, forest plot, scatterplot of associations between genetic variants and outcome vs between genetic variants and exposure). | Figures 2, Supplementary Figure 1 and  Supplementary Figure 2 |
| 12a | Assessment of assumptions | Report the assessment of the validity of the assumptions by removing confounders-related SNPs. | Results, mendelian randomization analyses for vestibular disorders and inflammatory cytokines |
| 12b |  | Report any additional statistics (eg, assessments of heterogeneity across genetic variants, such as I2, Q statistic). |  |
| 13a | Sensitivity analyses and additional analyses | Report any sensitivity analyses to assess the robustness of the main results to violations of the assumptions. | Results, mendelian randomization analyses for MS risk and 29 distinct traits cytokines, Supplementary Table 3 |
| 13b |  | Report results from other sensitivity analyses or additional analyses |  |
| 13c |  | Report any assessment of the direction of the causal relationship. |  |
| 13d |  | When relevant, report and compare with estimates from other RCTs and meta-analyses. | N/A |
| 13e |  | Consider additional plots to visualize results. | Figure 3 and 4 |
| 14 | Key results | Summarize key results with reference to study objectives. | Discussion: paragraph 1 |
| 15 | Limitations | Discuss limitations of the study, taking into account the validity of the IV assumptions, other sources of potential bias, and imprecision. Discuss both direction and magnitude of any potential bias and any efforts to address them. | Discussion: paragraph 7 |
| 16a | Interpretation | Meaning: Give a cautious overall interpretation of results in the context of their limitations and in comparison with other studies. | Discussion: paragraphs 2-6 |
| 16b |  | Mechanism: Discuss underlying biological mechanisms that could drive a potential causal relationship between the investigated exposure and the outcome, and whether the gene-environment equivalence assumption is reasonable. Use causal language carefully, clarifying that IV estimates may provide causal effects only under certain assumptions. |  |
| 16c |  | Clinical relevance: Discuss whether the results have clinical or public policy relevance, and to what extent they inform effect sizes of possible interventions. |  |
| 17 |  | Discuss the generalizability of the study results (a) to other populations, (b) across other exposure periods/timings, and (c) across other levels of exposure. |  |
| 18 | Funding | Describe sources of funding and the role offunders in the present study. | Funding statement |
| 19 | Data and data sharing | Provide the data used to perform all analyses or report where and how the data can be accessed, and reference these sources in the article. | Materials and Methods: data sources |
| 20 | Conflicts of interest | All authors should declare all potential conflicts of interest. | Declaration of Competing Interest |

**Abbreviation:** STROBE-MR, Strengthening the reporting of observational studies in epidemiology-mendelian randomization; MR, Mendelian randomization; IVs, Instrumental variables; SNPs, Single nucleotide polymorphisms; SD, Standard deviation; RCTs, Randomized controlled trials

**Supplementary Table 2** Single nucleotide polymorphisms (SNPs) were used as instrumental variables (IVs) from sleep disorders, dietary factors, metabolic factors and physical activity

| **Exposure** | **SNPs** | **EA** | **OA** | **EAF** | **Beta** | **SE** | **P value** | **F** |
| --- | --- | --- | --- | --- | --- | --- | --- | --- |
| Snoring | rs11205713 | C | T | 0.435 | -0.006 | 0.001 | 1.70E-09 | 36.337 |
| Snoring | rs12119849 | A | G | 0.086 | -0.012 | 0.002 | 9.90E-11 | 41.833 |
| Snoring | rs72902175 | T | C | 0.134 | -0.012 | 0.001 | 3.70E-15 | 61.846 |
| Snoring | rs1374895 | T | C | 0.547 | 0.006 | 0.001 | 1.00E-09 | 37.329 |
| Snoring | rs75563552 | G | A | 0.250 | -0.007 | 0.001 | 3.40E-08 | 30.484 |
| Snoring | rs1609721 | C | T | 0.397 | 0.006 | 0.001 | 5.80E-09 | 33.900 |
| Snoring | rs4493577 | A | G | 0.242 | -0.007 | 0.001 | 3.20E-08 | 30.579 |
| Snoring | rs34811474 | A | G | 0.231 | 0.008 | 0.001 | 1.70E-10 | 40.826 |
| Snoring | rs4976269 | A | G | 0.328 | 0.007 | 0.001 | 2.20E-10 | 40.271 |
| Snoring | rs2307111 | C | T | 0.395 | 0.007 | 0.001 | 7.10E-13 | 51.508 |
| Snoring | rs13156484 | A | G | 0.472 | 0.006 | 0.001 | 3.00E-08 | 30.708 |
| Snoring | rs17060460 | G | A | 0.226 | -0.007 | 0.001 | 4.00E-09 | 34.638 |
| Snoring | rs17151229 | C | G | 0.340 | -0.007 | 0.001 | 9.20E-10 | 37.497 |
| Snoring | rs13251292 | G | A | 0.409 | -0.007 | 0.001 | 1.80E-12 | 49.697 |
| Snoring | rs4523230 | T | A | 0.716 | -0.007 | 0.001 | 7.90E-11 | 42.271 |
| Snoring | rs4744369 | A | T | 0.586 | -0.006 | 0.001 | 1.80E-09 | 36.228 |
| Snoring | rs2648315 | T | C | 0.340 | -0.006 | 0.001 | 1.30E-08 | 32.340 |
| Snoring | rs957349 | A | T | 0.812 | 0.009 | 0.001 | 1.10E-11 | 46.101 |
| Snoring | rs2049045 | C | G | 0.186 | 0.009 | 0.001 | 1.60E-11 | 45.438 |
| Snoring | rs11018488 | T | A | 0.374 | 0.007 | 0.001 | 7.30E-10 | 37.947 |
| Snoring | rs10765567 | T | A | 0.373 | -0.006 | 0.001 | 4.10E-09 | 34.564 |
| Snoring | rs10505911 | A | C | 0.221 | -0.007 | 0.001 | 1.90E-08 | 31.616 |
| Snoring | rs10878271 | C | T | 0.636 | 0.009 | 0.001 | 5.60E-18 | 74.653 |
| Snoring | rs10844665 | A | C | 0.413 | -0.006 | 0.001 | 2.10E-08 | 31.366 |
| Snoring | rs9521988 | A | G | 0.340 | 0.006 | 0.001 | 1.50E-08 | 31.991 |
| Snoring | rs592333 | G | A | 0.444 | -0.009 | 0.001 | 5.00E-18 | 74.864 |
| Snoring | rs12429765 | G | A | 0.491 | 0.007 | 0.001 | 7.80E-11 | 42.313 |
| Snoring | rs743122 | C | T | 0.301 | -0.006 | 0.001 | 1.60E-08 | 31.970 |
| Snoring | rs2664303 | T | C | 0.421 | 0.007 | 0.001 | 7.30E-13 | 51.464 |
| Snoring | rs11855999 | C | A | 0.279 | 0.006 | 0.001 | 4.70E-08 | 29.819 |
| Snoring | rs1558901 | T | A | 0.423 | -0.007 | 0.001 | 3.00E-11 | 44.202 |
| Snoring | rs34838 | G | A | 0.446 | -0.006 | 0.001 | 2.90E-08 | 30.769 |
| Snoring | rs1108431 | T | C | 0.372 | -0.007 | 0.001 | 2.50E-10 | 39.998 |
| Snoring | rs78608249 | T | C | 0.290 | -0.006 | 0.001 | 7.70E-09 | 33.344 |
| Snoring | rs227727 | T | A | 0.456 | -0.006 | 0.001 | 6.70E-10 | 38.098 |
| Snoring | rs180110 | A | G | 0.591 | -0.007 | 0.001 | 6.60E-12 | 47.132 |
| Snoring | rs12150290 | C | A | 0.191 | -0.007 | 0.001 | 3.10E-08 | 30.663 |
| Snoring | rs8069947 | T | C | 0.447 | 0.007 | 0.001 | 4.50E-11 | 43.384 |
| Snoring | rs12603115 | T | C | 0.576 | 0.006 | 0.001 | 3.50E-09 | 34.900 |
| Snoring | rs57222984 | G | A | 0.244 | -0.008 | 0.001 | 1.70E-11 | 45.242 |
| Snoring | rs4987719 | T | C | 0.034 | -0.016 | 0.003 | 9.60E-09 | 32.930 |
| Snoring | rs73030263 | C | T | 0.095 | 0.011 | 0.002 | 3.40E-10 | 39.425 |
| Snoring | rs6054427 | A | G | 0.621 | -0.007 | 0.001 | 3.40E-10 | 39.448 |
| Snoring | rs6099273 | T | C | 0.251 | -0.007 | 0.001 | 4.60E-09 | 34.364 |
| Chronotype | rs516134 | T | C | 0.969 | -0.081 | 0.011 | 8.90E-13 | 51.076 |
| Chronotype | rs2050122 | C | T | 0.805 | -0.028 | 0.005 | 2.40E-08 | 31.148 |
| Chronotype | rs12140153 | T | G | 0.098 | -0.039 | 0.007 | 7.10E-09 | 33.510 |
| Chronotype | rs113240734 | A | G | 0.161 | 0.037 | 0.005 | 2.20E-12 | 49.330 |
| Chronotype | rs10157197 | A | G | 0.398 | -0.025 | 0.004 | 1.00E-09 | 37.254 |
| Chronotype | rs1075265 | G | C | 0.522 | 0.025 | 0.004 | 1.80E-10 | 40.629 |
| Chronotype | rs12635074 | G | T | 0.317 | 0.023 | 0.004 | 2.80E-08 | 30.824 |
| Chronotype | rs5876436 | C | A | 0.784 | -0.026 | 0.005 | 4.30E-08 | 30.021 |
| Chronotype | rs372229746 | A | G | 0.448 | -0.028 | 0.005 | 4.10E-09 | 34.586 |
| Chronotype | rs77641763 | T | C | 0.121 | -0.039 | 0.006 | 5.30E-11 | 43.054 |
| Chronotype | rs9961653 | C | T | 0.578 | -0.023 | 0.004 | 9.60E-09 | 32.930 |
| Chronotype | rs4821940 | C | T | 0.553 | -0.022 | 0.004 | 3.40E-08 | 30.482 |
| Sleep duration | rs915416 | G | C | 0.709 | -0.013 | 0.002 | 4.80E-13 | 52.277 |
| Sleep duration | rs2186122 | T | A | 0.560 | -0.011 | 0.002 | 2.30E-11 | 44.685 |
| Sleep duration | rs2279681 | G | C | 0.342 | 0.009 | 0.002 | 3.40E-08 | 30.462 |
| Sleep duration | rs7517981 | C | T | 0.601 | -0.010 | 0.002 | 1.10E-09 | 37.198 |
| Sleep duration | rs12567114 | A | G | 0.276 | 0.012 | 0.002 | 6.10E-12 | 47.282 |
| Sleep duration | rs1463053 | A | G | 0.640 | 0.009 | 0.002 | 2.40E-08 | 31.132 |
| Sleep duration | rs6681755 | A | G | 0.200 | 0.012 | 0.002 | 9.00E-09 | 33.054 |
| Sleep duration | rs374153 | T | C | 0.843 | -0.013 | 0.002 | 2.50E-09 | 35.548 |
| Sleep duration | rs2863957 | A | C | 0.221 | 0.029 | 0.002 | 9.60E-51 | 224.459 |
| Sleep duration | rs1972712 | C | T | 0.249 | 0.012 | 0.002 | 1.70E-10 | 40.763 |
| Sleep duration | rs72831782 | A | T | 0.269 | -0.010 | 0.002 | 3.40E-08 | 30.490 |
| Sleep duration | rs2683630 | G | C | 0.629 | 0.015 | 0.002 | 1.70E-19 | 81.557 |
| Sleep duration | rs75539574 | C | A | 0.086 | 0.024 | 0.003 | 1.80E-16 | 67.790 |
| Sleep duration | rs35662245 | A | T | 0.339 | 0.010 | 0.002 | 1.90E-09 | 36.088 |
| Sleep duration | rs6783516 | T | G | 0.584 | -0.010 | 0.002 | 1.60E-09 | 36.375 |
| Sleep duration | rs76258078 | G | A | 0.050 | -0.022 | 0.004 | 3.80E-09 | 34.705 |
| Sleep duration | rs113021516 | C | G | 0.336 | 0.011 | 0.002 | 1.30E-11 | 45.756 |
| Sleep duration | rs17732997 | G | C | 0.430 | -0.009 | 0.002 | 4.60E-08 | 29.858 |
| Sleep duration | rs9810474 | T | C | 0.232 | -0.011 | 0.002 | 3.90E-09 | 34.686 |
| Sleep duration | rs7644809 | C | T | 0.576 | -0.010 | 0.002 | 4.10E-10 | 39.044 |
| Sleep duration | rs13107325 | T | C | 0.075 | -0.024 | 0.003 | 1.40E-15 | 63.767 |
| Sleep duration | rs2192528 | G | A | 0.522 | -0.010 | 0.002 | 9.10E-10 | 37.503 |
| Sleep duration | rs2839753 | C | T | 0.265 | -0.011 | 0.002 | 4.40E-09 | 34.442 |
| Sleep duration | rs7711696 | T | G | 0.305 | -0.010 | 0.002 | 1.30E-08 | 32.331 |
| Sleep duration | rs12518468 | C | T | 0.329 | -0.011 | 0.002 | 4.10E-10 | 39.066 |
| Sleep duration | rs365663 | G | A | 0.455 | -0.009 | 0.002 | 8.10E-09 | 33.246 |
| Sleep duration | rs6889592 | A | G | 0.333 | 0.012 | 0.002 | 4.10E-12 | 48.070 |
| Sleep duration | rs151014368 | A | G | 0.207 | 0.011 | 0.002 | 1.00E-08 | 32.757 |
| Sleep duration | rs9382445 | C | T | 0.375 | -0.009 | 0.002 | 8.90E-09 | 33.077 |
| Sleep duration | rs1611719 | A | G | 0.210 | -0.013 | 0.002 | 7.10E-11 | 42.500 |
| Sleep duration | rs113113059 | C | T | 0.220 | -0.011 | 0.002 | 8.60E-09 | 33.129 |
| Sleep duration | rs7740402 | G | T | 0.306 | -0.010 | 0.002 | 4.10E-08 | 30.090 |
| Sleep duration | rs9345234 | C | A | 0.578 | 0.009 | 0.002 | 1.50E-08 | 32.057 |
| Sleep duration | rs34556183 | G | A | 0.280 | -0.013 | 0.002 | 6.60E-14 | 56.181 |
| Sleep duration | rs11982852 | T | C | 0.244 | -0.012 | 0.002 | 3.10E-10 | 39.636 |
| Sleep duration | rs62444917 | C | A | 0.222 | 0.013 | 0.002 | 1.70E-11 | 45.290 |
| Sleep duration | rs2079070 | G | C | 0.735 | -0.013 | 0.002 | 1.20E-13 | 55.052 |
| Sleep duration | rs7831557 | A | G | 0.517 | -0.011 | 0.002 | 4.20E-11 | 43.533 |
| Sleep duration | rs7016314 | C | T | 0.656 | 0.010 | 0.002 | 3.10E-09 | 35.094 |
| Sleep duration | rs17391944 | G | T | 0.050 | 0.022 | 0.004 | 4.40E-09 | 34.431 |
| Sleep duration | rs112100783 | A | G | 0.033 | -0.025 | 0.005 | 2.70E-08 | 30.926 |
| Sleep duration | rs10510128 | A | G | 0.208 | 0.011 | 0.002 | 7.70E-09 | 33.359 |
| Sleep duration | rs2236295 | T | G | 0.403 | -0.009 | 0.002 | 2.90E-08 | 30.762 |
| Sleep duration | rs1517572 | C | A | 0.581 | 0.012 | 0.002 | 6.50E-13 | 51.678 |
| Sleep duration | rs7115856 | C | A | 0.461 | 0.011 | 0.002 | 1.50E-11 | 45.564 |
| Sleep duration | rs11039216 | T | C | 0.533 | -0.010 | 0.002 | 1.50E-10 | 41.062 |
| Sleep duration | rs2734831 | G | T | 0.607 | -0.010 | 0.002 | 2.20E-09 | 35.752 |
| Sleep duration | rs174564 | G | A | 0.349 | 0.010 | 0.002 | 6.30E-09 | 33.730 |
| Sleep duration | rs1939455 | T | G | 0.120 | -0.016 | 0.003 | 3.50E-10 | 39.393 |
| Sleep duration | rs1553132 | G | A | 0.259 | 0.011 | 0.002 | 8.10E-09 | 33.253 |
| Sleep duration | rs34354917 | A | C | 0.289 | -0.010 | 0.002 | 1.40E-08 | 32.121 |
| Sleep duration | rs4767550 | G | A | 0.413 | 0.011 | 0.002 | 2.70E-11 | 44.354 |
| Sleep duration | rs6561715 | A | T | 0.631 | 0.010 | 0.002 | 3.90E-09 | 34.685 |
| Sleep duration | rs55658675 | T | C | 0.353 | -0.010 | 0.002 | 7.10E-09 | 33.514 |
| Sleep duration | rs11621908 | T | C | 0.083 | -0.020 | 0.003 | 1.10E-11 | 46.105 |
| Sleep duration | rs2748809 | C | T | 0.429 | -0.009 | 0.002 | 1.90E-08 | 31.624 |
| Sleep duration | rs8038326 | G | A | 0.273 | -0.013 | 0.002 | 8.40E-14 | 55.712 |
| Sleep duration | rs56367859 | G | A | 0.398 | 0.012 | 0.002 | 1.20E-12 | 50.473 |
| Sleep duration | rs9302680 | A | G | 0.439 | 0.012 | 0.002 | 7.60E-14 | 55.912 |
| Sleep duration | rs11643715 | G | C | 0.293 | 0.011 | 0.002 | 5.00E-10 | 38.688 |
| Sleep duration | rs8047587 | T | G | 0.440 | -0.011 | 0.002 | 8.30E-12 | 46.684 |
| Sleep duration | rs72771082 | G | A | 0.218 | 0.011 | 0.002 | 1.40E-08 | 32.135 |
| Sleep duration | rs8074498 | A | T | 0.581 | -0.009 | 0.002 | 1.10E-08 | 32.595 |
| Sleep duration | rs11650677 | A | G | 0.339 | 0.011 | 0.002 | 3.80E-11 | 43.705 |
| Sleep duration | rs9903898 | T | C | 0.489 | -0.009 | 0.002 | 3.60E-09 | 34.826 |
| Sleep duration | rs8072993 | G | T | 0.636 | 0.011 | 0.002 | 4.20E-08 | 30.049 |
| Sleep duration | rs1348047 | T | G | 0.267 | -0.013 | 0.002 | 3.80E-12 | 48.223 |
| Sleep duration | rs35126035 | C | A | 0.558 | -0.009 | 0.002 | 2.20E-08 | 31.294 |
| Sleep duration | rs34786000 | T | G | 0.553 | 0.011 | 0.002 | 1.70E-11 | 45.310 |
| Sleep duration | rs2072727 | C | T | 0.565 | -0.009 | 0.002 | 9.30E-09 | 32.981 |
| Sleep duration | rs9611007 | T | C | 0.142 | -0.014 | 0.002 | 3.30E-09 | 35.017 |
| Short sleep duration | rs62158211 | T | G | 0.212 | -0.010 | 0.002 | 2.80E-06 | 21.936 |
| Short sleep duration | rs573615914 | T | C | 0.011 | 0.047 | 0.010 | 8.70E-07 | 24.194 |
| Short sleep duration | rs112287772 | C | T | 0.014 | 0.038 | 0.008 | 1.70E-06 | 22.902 |
| Short sleep duration | rs9474974 | G | C | 0.569 | -0.008 | 0.002 | 4.90E-06 | 20.873 |
| Short sleep duration | rs546786239 | C | T | 0.006 | 0.059 | 0.013 | 3.00E-06 | 21.806 |
| Short sleep duration | rs1456031 | C | T | 0.555 | 0.009 | 0.002 | 1.30E-06 | 23.376 |
| Short sleep duration | rs66501154 | G | A | 0.222 | -0.011 | 0.002 | 6.60E-07 | 24.728 |
| Short sleep duration | rs10510128 | A | G | 0.205 | -0.011 | 0.002 | 1.00E-06 | 23.893 |
| Short sleep duration | rs10995281 | C | T | 0.414 | 0.009 | 0.002 | 4.10E-06 | 21.214 |
| Short sleep duration | rs7483691 | T | C | 0.714 | 0.010 | 0.002 | 5.10E-07 | 25.213 |
| Short sleep duration | rs11607779 | T | C | 0.038 | 0.023 | 0.005 | 1.60E-06 | 23.059 |
| Short sleep duration | rs116988821 | A | G | 0.030 | 0.024 | 0.005 | 4.40E-06 | 21.080 |
| Short sleep duration | rs8008258 | T | C | 0.430 | 0.009 | 0.002 | 3.60E-06 | 21.492 |
| Short sleep duration | rs140450540 | A | G | 0.016 | 0.038 | 0.008 | 8.80E-07 | 24.175 |
| Short sleep duration | rs74500417 | C | A | 0.512 | 0.012 | 0.003 | 4.20E-06 | 21.169 |
| Long sleep duration | rs12751487 | T | A | 0.269 | -0.007 | 0.002 | 3.10E-06 | 21.775 |
| Long sleep duration | rs114263478 | T | C | 0.013 | 0.028 | 0.006 | 4.00E-06 | 21.275 |
| Long sleep duration | rs2387776 | C | T | 0.694 | 0.008 | 0.002 | 4.40E-06 | 21.068 |
| Long sleep duration | rs3845807 | T | C | 0.530 | -0.006 | 0.001 | 3.40E-06 | 21.558 |
| Long sleep duration | rs6781801 | A | G | 0.300 | -0.008 | 0.002 | 5.00E-07 | 25.258 |
| Long sleep duration | rs1471172 | A | G | 0.657 | -0.007 | 0.001 | 1.60E-06 | 23.004 |
| Long sleep duration | rs79245886 | A | G | 0.023 | -0.022 | 0.005 | 3.00E-06 | 21.842 |
| Long sleep duration | rs2973993 | T | A | 0.493 | 0.007 | 0.001 | 1.50E-06 | 23.087 |
| Long sleep duration | rs190517796 | T | C | 0.010 | 0.039 | 0.008 | 1.70E-06 | 22.870 |
| Long sleep duration | rs10039466 | A | C | 0.001 | 0.122 | 0.027 | 4.10E-06 | 21.197 |
| Long sleep duration | rs113012428 | A | G | 0.115 | 0.011 | 0.002 | 1.10E-06 | 23.743 |
| Long sleep duration | rs6456355 | T | A | 0.752 | -0.007 | 0.002 | 4.40E-06 | 21.081 |
| Long sleep duration | rs148948944 | A | T | 0.013 | 0.030 | 0.006 | 3.70E-06 | 21.430 |
| Long sleep duration | rs73196898 | A | C | 0.194 | -0.008 | 0.002 | 2.10E-06 | 22.497 |
| Long sleep duration | rs73538586 | C | T | 0.018 | 0.025 | 0.005 | 2.70E-06 | 21.998 |
| Long sleep duration | rs138854460 | C | T | 0.034 | 0.018 | 0.004 | 3.70E-06 | 21.403 |
| Long sleep duration | rs58517549 | C | T | 0.046 | 0.016 | 0.003 | 2.10E-06 | 22.502 |
| Long sleep duration | rs60780105 | C | T | 0.171 | -0.009 | 0.002 | 3.60E-06 | 21.453 |
| Long sleep duration | rs183208795 | C | T | 0.019 | 0.027 | 0.005 | 7.40E-07 | 24.499 |
| Long sleep duration | rs9915132 | G | T | 0.018 | -0.026 | 0.005 | 6.50E-07 | 24.746 |
| Long sleep duration | rs55700403 | A | C | 0.015 | 0.027 | 0.006 | 2.30E-06 | 22.356 |
| Long sleep duration | rs4006399 | C | T | 0.017 | 0.028 | 0.006 | 7.80E-07 | 24.396 |
| Long sleep duration | rs6035251 | C | A | 0.929 | -0.013 | 0.003 | 1.10E-06 | 23.778 |
| Long sleep duration | rs35718341 | G | A | 0.036 | 0.019 | 0.004 | 3.70E-07 | 25.856 |
| Sleep duration | rs915416 | G | C | 0.709 | -0.013 | 0.002 | 4.80E-13 | 52.277 |
| Sleep duration | rs2186122 | T | A | 0.560 | -0.011 | 0.002 | 2.30E-11 | 44.685 |
| Sleep duration | rs2279681 | G | C | 0.342 | 0.009 | 0.002 | 3.40E-08 | 30.462 |
| Sleep duration | rs7517981 | C | T | 0.601 | -0.010 | 0.002 | 1.10E-09 | 37.198 |
| Sleep duration | rs12567114 | A | G | 0.276 | 0.012 | 0.002 | 6.10E-12 | 47.282 |
| Sleep duration | rs1463053 | A | G | 0.640 | 0.009 | 0.002 | 2.40E-08 | 31.132 |
| Sleep duration | rs6681755 | A | G | 0.200 | 0.012 | 0.002 | 9.00E-09 | 33.054 |
| Sleep duration | rs374153 | T | C | 0.843 | -0.013 | 0.002 | 2.50E-09 | 35.548 |
| Sleep duration | rs2863957 | A | C | 0.221 | 0.029 | 0.002 | 9.60E-51 | 224.459 |
| Sleep duration | rs1972712 | C | T | 0.249 | 0.012 | 0.002 | 1.70E-10 | 40.763 |
| Sleep duration | rs72831782 | A | T | 0.269 | -0.010 | 0.002 | 3.40E-08 | 30.490 |
| Sleep duration | rs2683630 | G | C | 0.629 | 0.015 | 0.002 | 1.70E-19 | 81.557 |
| Sleep duration | rs75539574 | C | A | 0.086 | 0.024 | 0.003 | 1.80E-16 | 67.790 |
| Sleep duration | rs35662245 | A | T | 0.339 | 0.010 | 0.002 | 1.90E-09 | 36.088 |
| Sleep duration | rs6783516 | T | G | 0.584 | -0.010 | 0.002 | 1.60E-09 | 36.375 |
| Sleep duration | rs76258078 | G | A | 0.050 | -0.022 | 0.004 | 3.80E-09 | 34.705 |
| Sleep duration | rs113021516 | C | G | 0.336 | 0.011 | 0.002 | 1.30E-11 | 45.756 |
| Sleep duration | rs17732997 | G | C | 0.430 | -0.009 | 0.002 | 4.60E-08 | 29.858 |
| Sleep duration | rs9810474 | T | C | 0.232 | -0.011 | 0.002 | 3.90E-09 | 34.686 |
| Sleep duration | rs7644809 | C | T | 0.576 | -0.010 | 0.002 | 4.10E-10 | 39.044 |
| Sleep duration | rs13107325 | T | C | 0.075 | -0.024 | 0.003 | 1.40E-15 | 63.767 |
| Sleep duration | rs2192528 | G | A | 0.522 | -0.010 | 0.002 | 9.10E-10 | 37.503 |
| Sleep duration | rs2839753 | C | T | 0.265 | -0.011 | 0.002 | 4.40E-09 | 34.442 |
| Sleep duration | rs7711696 | T | G | 0.305 | -0.010 | 0.002 | 1.30E-08 | 32.331 |
| Sleep duration | rs12518468 | C | T | 0.329 | -0.011 | 0.002 | 4.10E-10 | 39.066 |
| Sleep duration | rs365663 | G | A | 0.455 | -0.009 | 0.002 | 8.10E-09 | 33.246 |
| Sleep duration | rs6889592 | A | G | 0.333 | 0.012 | 0.002 | 4.10E-12 | 48.070 |
| Sleep duration | rs151014368 | A | G | 0.207 | 0.011 | 0.002 | 1.00E-08 | 32.757 |
| Sleep duration | rs9382445 | C | T | 0.375 | -0.009 | 0.002 | 8.90E-09 | 33.077 |
| Sleep duration | rs1611719 | A | G | 0.210 | -0.013 | 0.002 | 7.10E-11 | 42.500 |
| Sleep duration | rs113113059 | C | T | 0.220 | -0.011 | 0.002 | 8.60E-09 | 33.129 |
| Sleep duration | rs7740402 | G | T | 0.306 | -0.010 | 0.002 | 4.10E-08 | 30.090 |
| Sleep duration | rs9345234 | C | A | 0.578 | 0.009 | 0.002 | 1.50E-08 | 32.057 |
| Sleep duration | rs34556183 | G | A | 0.280 | -0.013 | 0.002 | 6.60E-14 | 56.181 |
| Sleep duration | rs11982852 | T | C | 0.244 | -0.012 | 0.002 | 3.10E-10 | 39.636 |
| Sleep duration | rs62444917 | C | A | 0.222 | 0.013 | 0.002 | 1.70E-11 | 45.290 |
| Sleep duration | rs2079070 | G | C | 0.735 | -0.013 | 0.002 | 1.20E-13 | 55.052 |
| Sleep duration | rs7831557 | A | G | 0.517 | -0.011 | 0.002 | 4.20E-11 | 43.533 |
| Sleep duration | rs7016314 | C | T | 0.656 | 0.010 | 0.002 | 3.10E-09 | 35.094 |
| Sleep duration | rs17391944 | G | T | 0.050 | 0.022 | 0.004 | 4.40E-09 | 34.431 |
| Sleep duration | rs112100783 | A | G | 0.033 | -0.025 | 0.005 | 2.70E-08 | 30.926 |
| Sleep duration | rs10510128 | A | G | 0.208 | 0.011 | 0.002 | 7.70E-09 | 33.359 |
| Sleep duration | rs2236295 | T | G | 0.403 | -0.009 | 0.002 | 2.90E-08 | 30.762 |
| Sleep duration | rs1517572 | C | A | 0.581 | 0.012 | 0.002 | 6.50E-13 | 51.678 |
| Sleep duration | rs7115856 | C | A | 0.461 | 0.011 | 0.002 | 1.50E-11 | 45.564 |
| Sleep duration | rs11039216 | T | C | 0.533 | -0.010 | 0.002 | 1.50E-10 | 41.062 |
| Sleep duration | rs2734831 | G | T | 0.607 | -0.010 | 0.002 | 2.20E-09 | 35.752 |
| Sleep duration | rs174564 | G | A | 0.349 | 0.010 | 0.002 | 6.30E-09 | 33.730 |
| Sleep duration | rs1939455 | T | G | 0.120 | -0.016 | 0.003 | 3.50E-10 | 39.393 |
| Sleep duration | rs1553132 | G | A | 0.259 | 0.011 | 0.002 | 8.10E-09 | 33.253 |
| Sleep duration | rs34354917 | A | C | 0.289 | -0.010 | 0.002 | 1.40E-08 | 32.121 |
| Sleep duration | rs4767550 | G | A | 0.413 | 0.011 | 0.002 | 2.70E-11 | 44.354 |
| Sleep duration | rs6561715 | A | T | 0.631 | 0.010 | 0.002 | 3.90E-09 | 34.685 |
| Sleep duration | rs55658675 | T | C | 0.353 | -0.010 | 0.002 | 7.10E-09 | 33.514 |
| Sleep duration | rs11621908 | T | C | 0.083 | -0.020 | 0.003 | 1.10E-11 | 46.105 |
| Sleep duration | rs2748809 | C | T | 0.429 | -0.009 | 0.002 | 1.90E-08 | 31.624 |
| Sleep duration | rs8038326 | G | A | 0.273 | -0.013 | 0.002 | 8.40E-14 | 55.712 |
| Sleep duration | rs56367859 | G | A | 0.398 | 0.012 | 0.002 | 1.20E-12 | 50.473 |
| Sleep duration | rs9302680 | A | G | 0.439 | 0.012 | 0.002 | 7.60E-14 | 55.912 |
| Sleep duration | rs11643715 | G | C | 0.293 | 0.011 | 0.002 | 5.00E-10 | 38.688 |
| Sleep duration | rs8047587 | T | G | 0.440 | -0.011 | 0.002 | 8.30E-12 | 46.684 |
| Sleep duration | rs72771082 | G | A | 0.218 | 0.011 | 0.002 | 1.40E-08 | 32.135 |
| Sleep duration | rs8074498 | A | T | 0.581 | -0.009 | 0.002 | 1.10E-08 | 32.595 |
| Sleep duration | rs11650677 | A | G | 0.339 | 0.011 | 0.002 | 3.80E-11 | 43.705 |
| Sleep duration | rs9903898 | T | C | 0.489 | -0.009 | 0.002 | 3.60E-09 | 34.826 |
| Sleep duration | rs8072993 | G | T | 0.636 | 0.011 | 0.002 | 4.20E-08 | 30.049 |
| Sleep duration | rs1348047 | T | G | 0.267 | -0.013 | 0.002 | 3.80E-12 | 48.223 |
| Sleep duration | rs35126035 | C | A | 0.558 | -0.009 | 0.002 | 2.20E-08 | 31.294 |
| Sleep duration | rs34786000 | T | G | 0.553 | 0.011 | 0.002 | 1.70E-11 | 45.310 |
| Sleep duration | rs2072727 | C | T | 0.565 | -0.009 | 0.002 | 9.30E-09 | 32.981 |
| Sleep duration | rs9611007 | T | C | 0.142 | -0.014 | 0.002 | 3.30E-09 | 35.017 |
| Sleep apnea syndrome | rs58138946 | A | G | 0.044 | 0.170 | 0.037 | 4.05E-06 | 21.215 |
| Sleep apnea syndrome | rs17513135 | T | C | 0.206 | 0.077 | 0.016 | 7.37E-07 | 24.550 |
| Sleep apnea syndrome | rs573593537 | C | T | 0.078 | 0.203 | 0.044 | 3.95E-06 | 21.273 |
| Sleep apnea syndrome | rs116182122 | G | T | 0.013 | 0.309 | 0.060 | 2.27E-07 | 26.752 |
| Sleep apnea syndrome | rs116420235 | T | C | 0.016 | -0.209 | 0.044 | 2.51E-06 | 22.158 |
| Sleep apnea syndrome | rs16861404 | A | G | 0.169 | 0.079 | 0.017 | 2.10E-06 | 22.435 |
| Sleep apnea syndrome | rs114983649 | A | C | 0.042 | 0.154 | 0.030 | 3.66E-07 | 25.899 |
| Sleep apnea syndrome | rs79672111 | A | G | 0.018 | 0.239 | 0.052 | 4.46E-06 | 21.061 |
| Sleep apnea syndrome | rs10513713 | A | G | 0.274 | 0.075 | 0.016 | 1.52E-06 | 22.991 |
| Sleep apnea syndrome | rs145058358 | C | A | 0.056 | 0.158 | 0.032 | 5.58E-07 | 25.095 |
| Sleep apnea syndrome | rs4488813 | G | A | 0.132 | -0.106 | 0.023 | 4.50E-06 | 21.017 |
| Sleep apnea syndrome | rs6815442 | T | A | 0.315 | 0.069 | 0.015 | 2.53E-06 | 22.142 |
| Sleep apnea syndrome | rs4365709 | C | A | 0.396 | 0.061 | 0.013 | 3.94E-06 | 21.399 |
| Sleep apnea syndrome | rs2611742 | C | T | 0.422 | 0.063 | 0.013 | 1.30E-06 | 23.411 |
| Sleep apnea syndrome | rs4559013 | G | A | 0.514 | -0.059 | 0.013 | 4.67E-06 | 21.131 |
| Sleep apnea syndrome | rs142416325 | A | G | 0.012 | -0.347 | 0.067 | 2.09E-07 | 26.987 |
| Sleep apnea syndrome | rs115903639 | A | G | 0.014 | 0.193 | 0.042 | 4.87E-06 | 20.839 |
| Sleep apnea syndrome | rs1885070 | G | A | 0.283 | 0.066 | 0.014 | 4.32E-06 | 21.198 |
| Sleep apnea syndrome | rs146794654 | T | C | 0.019 | -0.202 | 0.043 | 1.90E-06 | 22.658 |
| Sleep apnea syndrome | rs17547430 | G | A | 0.060 | 0.127 | 0.026 | 6.36E-07 | 24.726 |
| Sleep apnea syndrome | rs12699649 | T | C | 0.366 | 0.063 | 0.013 | 2.90E-06 | 22.034 |
| Sleep apnea syndrome | rs72683533 | A | G | 0.045 | 0.166 | 0.036 | 4.75E-06 | 20.887 |
| Sleep apnea syndrome | rs11774552 | T | C | 0.414 | -0.066 | 0.013 | 5.05E-07 | 25.152 |
| Sleep apnea syndrome | rs17212403 | A | G | 0.129 | 0.092 | 0.019 | 8.95E-07 | 24.200 |
| Sleep apnea syndrome | rs181154734 | A | G | 0.006 | 0.406 | 0.079 | 2.83E-07 | 26.345 |
| Sleep apnea syndrome | rs76054872 | T | G | 0.025 | 0.208 | 0.045 | 3.10E-06 | 21.764 |
| Sleep apnea syndrome | rs7133573 | T | C | 0.568 | -0.061 | 0.013 | 2.44E-06 | 22.235 |
| Sleep apnea syndrome | rs10507084 | T | C | 0.165 | 0.103 | 0.019 | 5.51E-08 | 29.527 |
| Sleep apnea syndrome | rs11173733 | T | A | 0.312 | 0.119 | 0.024 | 1.12E-06 | 23.781 |
| Sleep apnea syndrome | rs2277339 | G | T | 0.135 | -0.104 | 0.020 | 1.49E-07 | 27.709 |
| Sleep apnea syndrome | rs9318788 | G | A | 0.001 | 1.473 | 0.300 | 9.21E-07 | 24.086 |
| Sleep apnea syndrome | rs73156514 | G | C | 0.119 | 0.092 | 0.019 | 1.17E-06 | 23.643 |
| Sleep apnea syndrome | rs1956376 | T | C | 0.435 | 0.069 | 0.013 | 1.59E-07 | 27.324 |
| Sleep apnea syndrome | rs10906985 | A | G | 0.378 | 0.066 | 0.013 | 4.05E-07 | 25.775 |
| Sleep apnea syndrome | rs11075985 | A | C | 0.372 | 0.104 | 0.013 | 1.99E-15 | 63.509 |
| Sleep apnea syndrome | rs144160082 | G | A | 0.053 | 0.125 | 0.027 | 4.00E-06 | 21.344 |
| Sleep apnea syndrome | rs11704027 | G | A | 0.281 | 0.069 | 0.015 | 1.72E-06 | 22.842 |
| Sleep apnea syndrome | rs132561 | T | C | 0.586 | -0.064 | 0.014 | 3.17E-06 | 21.575 |
| Sleeplessness/insomnia | rs2803296 | C | G | 0.544 | -0.009 | 0.001 | 7.30E-09 | 33.464 |
| Sleeplessness/insomnia | rs12049261 | C | G | 0.293 | 0.011 | 0.002 | 6.80E-12 | 47.074 |
| Sleeplessness/insomnia | rs6690017 | G | T | 0.409 | -0.010 | 0.002 | 1.10E-11 | 46.217 |
| Sleeplessness/insomnia | rs2644128 | G | C | 0.548 | 0.011 | 0.001 | 1.00E-12 | 50.812 |
| Sleeplessness/insomnia | rs4572538 | T | C | 0.364 | -0.010 | 0.002 | 7.70E-10 | 37.839 |
| Sleeplessness/insomnia | rs56365214 | A | C | 0.156 | -0.015 | 0.002 | 5.60E-13 | 51.971 |
| Sleeplessness/insomnia | rs4577309 | G | A | 0.534 | -0.009 | 0.001 | 1.00E-08 | 32.820 |
| Sleeplessness/insomnia | rs12470989 | G | A | 0.204 | -0.010 | 0.002 | 2.80E-08 | 30.826 |
| Sleeplessness/insomnia | rs113851554 | T | G | 0.057 | 0.047 | 0.003 | 2.90E-45 | 199.345 |
| Sleeplessness/insomnia | rs56093896 | A | C | 0.214 | -0.012 | 0.002 | 7.70E-12 | 46.836 |
| Sleeplessness/insomnia | rs2014830 | T | C | 0.304 | -0.012 | 0.002 | 8.90E-13 | 51.080 |
| Sleeplessness/insomnia | rs705219 | A | T | 0.887 | 0.013 | 0.002 | 1.20E-08 | 32.543 |
| Sleeplessness/insomnia | rs9845387 | A | C | 0.040 | -0.022 | 0.004 | 7.10E-09 | 33.495 |
| Sleeplessness/insomnia | rs1988337 | G | A | 0.552 | 0.008 | 0.001 | 2.10E-08 | 31.413 |
| Sleeplessness/insomnia | rs11097861 | G | A | 0.716 | 0.010 | 0.002 | 1.10E-09 | 37.077 |
| Sleeplessness/insomnia | rs2604551 | G | T | 0.640 | -0.008 | 0.002 | 4.70E-08 | 29.846 |
| Sleeplessness/insomnia | rs1592757 | C | G | 0.356 | 0.010 | 0.002 | 4.30E-11 | 43.491 |
| Sleeplessness/insomnia | rs7711696 | T | G | 0.305 | 0.011 | 0.002 | 4.10E-12 | 48.066 |
| Sleeplessness/insomnia | rs1430205 | T | C | 0.462 | 0.009 | 0.001 | 2.10E-10 | 40.379 |
| Sleeplessness/insomnia | rs314280 | G | A | 0.547 | 0.010 | 0.001 | 7.30E-11 | 42.424 |
| Sleeplessness/insomnia | rs6975972 | G | A | 0.579 | -0.009 | 0.002 | 2.00E-09 | 35.960 |
| Sleeplessness/insomnia | rs8180817 | C | G | 0.431 | -0.010 | 0.002 | 2.70E-11 | 44.359 |
| Sleeplessness/insomnia | rs17151854 | T | G | 0.152 | 0.013 | 0.002 | 3.80E-10 | 39.211 |
| Sleeplessness/insomnia | rs11790060 | C | T | 0.331 | -0.010 | 0.002 | 5.80E-11 | 42.900 |
| Sleeplessness/insomnia | rs224032 | A | G | 0.550 | 0.008 | 0.001 | 1.80E-08 | 31.666 |
| Sleeplessness/insomnia | rs17709610 | G | A | 0.298 | -0.010 | 0.002 | 9.50E-10 | 37.431 |
| Sleeplessness/insomnia | rs2297787 | A | T | 0.080 | -0.018 | 0.003 | 9.60E-11 | 41.898 |
| Sleeplessness/insomnia | rs72924721 | T | C | 0.073 | 0.016 | 0.003 | 1.10E-08 | 32.712 |
| Sleeplessness/insomnia | rs10838708 | A | G | 0.459 | -0.009 | 0.002 | 2.90E-10 | 39.770 |
| Sleeplessness/insomnia | rs68094047 | T | C | 0.251 | 0.010 | 0.002 | 1.70E-09 | 36.248 |
| Sleeplessness/insomnia | rs931221 | A | T | 0.237 | 0.011 | 0.002 | 1.30E-09 | 36.808 |
| Sleeplessness/insomnia | rs324017 | C | A | 0.705 | -0.010 | 0.002 | 1.40E-09 | 36.693 |
| Sleeplessness/insomnia | rs9570080 | C | T | 0.344 | -0.011 | 0.002 | 1.60E-11 | 45.413 |
| Sleeplessness/insomnia | rs6561715 | A | T | 0.631 | -0.012 | 0.002 | 4.80E-14 | 56.791 |
| Sleeplessness/insomnia | rs1547630 | A | G | 0.652 | 0.009 | 0.002 | 5.80E-09 | 33.894 |
| Sleeplessness/insomnia | rs4886860 | C | G | 0.767 | -0.012 | 0.002 | 1.80E-11 | 45.143 |
| Sleeplessness/insomnia | rs11635495 | C | T | 0.512 | 0.009 | 0.001 | 2.80E-10 | 39.837 |
| Sleeplessness/insomnia | rs2062113 | C | T | 0.568 | -0.010 | 0.002 | 1.60E-10 | 40.953 |
| Sleeplessness/insomnia | rs9894577 | A | G | 0.318 | 0.013 | 0.002 | 1.30E-16 | 68.382 |
| Sleeplessness/insomnia | rs9906181 | G | A | 0.688 | -0.009 | 0.002 | 2.40E-08 | 31.180 |
| Sleeplessness/insomnia | rs11152363 | A | G | 0.186 | 0.016 | 0.002 | 4.50E-16 | 65.992 |
| Sleeplessness/insomnia | rs56330606 | G | A | 0.379 | 0.009 | 0.002 | 1.20E-09 | 37.000 |
| Tea intake | rs34619 | A | G | 0.431 | 0.012 | 0.002 | 4.30E-08 | 30.021 |
| Tea intake | rs11164870 | G | C | 0.605 | -0.012 | 0.002 | 4.20E-08 | 30.037 |
| Tea intake | rs17576658 | A | G | 0.247 | -0.013 | 0.002 | 4.10E-08 | 30.117 |
| Tea intake | rs2279844 | A | G | 0.379 | -0.012 | 0.002 | 4.00E-08 | 30.151 |
| Tea intake | rs2783129 | G | C | 0.485 | -0.012 | 0.002 | 3.80E-08 | 30.254 |
| Tea intake | rs2645929 | G | A | 0.813 | -0.015 | 0.003 | 3.50E-08 | 30.424 |
| Tea intake | rs7757102 | G | A | 0.555 | -0.012 | 0.002 | 3.10E-08 | 30.624 |
| Tea intake | rs9302428 | G | C | 0.636 | 0.012 | 0.002 | 2.60E-08 | 30.949 |
| Tea intake | rs17245213 | A | G | 0.208 | -0.015 | 0.003 | 2.00E-08 | 31.521 |
| Tea intake | rs10764990 | A | G | 0.607 | -0.012 | 0.002 | 1.90E-08 | 31.589 |
| Tea intake | rs57462170 | A | G | 0.109 | 0.019 | 0.003 | 1.90E-08 | 31.620 |
| Tea intake | rs57631352 | G | A | 0.297 | -0.013 | 0.002 | 1.70E-08 | 31.868 |
| Tea intake | rs2351187 | A | G | 0.319 | 0.013 | 0.002 | 1.60E-08 | 31.959 |
| Tea intake | rs149805207 | G | A | 0.009 | -0.072 | 0.013 | 1.10E-08 | 32.685 |
| Tea intake | rs9648476 | A | G | 0.623 | 0.013 | 0.002 | 1.10E-08 | 32.722 |
| Tea intake | rs13282783 | T | C | 0.286 | -0.014 | 0.002 | 7.90E-09 | 33.289 |
| Tea intake | rs56348300 | G | C | 0.185 | 0.016 | 0.003 | 6.10E-09 | 33.799 |
| Tea intake | rs1453548 | A | T | 0.665 | -0.013 | 0.002 | 3.00E-09 | 35.168 |
| Tea intake | rs1156588 | G | A | 0.210 | -0.015 | 0.003 | 2.90E-09 | 35.241 |
| Tea intake | rs141071726 | A | G | 0.027 | 0.041 | 0.007 | 2.20E-09 | 35.754 |
| Tea intake | rs2117137 | G | A | 0.405 | 0.013 | 0.002 | 1.70E-09 | 36.338 |
| Tea intake | rs10752269 | A | G | 0.506 | -0.013 | 0.002 | 1.30E-09 | 36.878 |
| Tea intake | rs713598 | G | C | 0.402 | 0.013 | 0.002 | 5.20E-10 | 38.590 |
| Tea intake | rs12591786 | T | C | 0.159 | -0.018 | 0.003 | 3.70E-10 | 39.274 |
| Tea intake | rs11587444 | G | A | 0.393 | 0.014 | 0.002 | 1.00E-10 | 41.789 |
| Tea intake | rs132904 | C | G | 0.779 | 0.017 | 0.003 | 7.80E-11 | 42.296 |
| Tea intake | rs9937354 | A | G | 0.424 | -0.014 | 0.002 | 4.90E-11 | 43.231 |
| Tea intake | rs2273447 | T | A | 0.204 | 0.017 | 0.003 | 3.30E-11 | 43.991 |
| Tea intake | rs4808193 | C | T | 0.335 | 0.015 | 0.002 | 1.70E-11 | 45.240 |
| Tea intake | rs10741694 | C | T | 0.628 | 0.015 | 0.002 | 7.90E-12 | 46.784 |
| Tea intake | rs4817505 | C | T | 0.390 | 0.015 | 0.002 | 4.20E-12 | 48.012 |
| Tea intake | rs72797284 | G | A | 0.271 | -0.017 | 0.002 | 7.00E-13 | 51.558 |
| Tea intake | rs56188862 | C | T | 0.387 | -0.016 | 0.002 | 4.30E-13 | 52.497 |
| Tea intake | rs977474 | T | C | 0.834 | 0.022 | 0.003 | 2.40E-14 | 58.180 |
| Tea intake | rs1481012 | G | A | 0.112 | -0.026 | 0.003 | 5.30E-15 | 61.148 |
| Tea intake | rs2478875 | G | A | 0.209 | 0.022 | 0.003 | 5.10E-17 | 70.299 |
| Tea intake | rs17685 | A | G | 0.278 | 0.023 | 0.002 | 1.60E-22 | 95.364 |
| Tea intake | rs9624470 | A | G | 0.580 | 0.025 | 0.002 | 1.30E-31 | 136.840 |
| Tea intake | rs4410790 | C | T | 0.631 | 0.041 | 0.002 | 3.40E-76 | 341.270 |
| Tea intake | rs2472297 | T | C | 0.262 | 0.053 | 0.002 | 2.30E-109 | 493.646 |
| Beef intake | rs11878917 | A | G | 0.110 | 0.015 | 0.003 | 4.60E-08 | 29.867 |
| Beef intake | rs62169335 | T | C | 0.543 | -0.010 | 0.002 | 2.40E-08 | 31.160 |
| Beef intake | rs1470610 | C | G | 0.196 | -0.012 | 0.002 | 1.50E-08 | 32.050 |
| Beef intake | rs11165829 | G | C | 0.360 | -0.010 | 0.002 | 9.80E-09 | 32.887 |
| Beef intake | rs1105388 | T | C | 0.300 | -0.011 | 0.002 | 1.30E-09 | 36.782 |
| Beef intake | rs132901 | T | C | 0.788 | 0.014 | 0.002 | 2.90E-11 | 44.271 |
| Beef intake | rs10789340 | G | A | 0.627 | -0.014 | 0.002 | 6.80E-15 | 60.652 |
| Beef intake | rs4676964 | T | C | 0.511 | 0.013 | 0.002 | 9.60E-15 | 59.967 |
| Pork intake | rs7641973 | A | G | 0.353 | 0.008 | 0.002 | 4.20E-08 | 30.042 |
| Pork intake | rs2387807 | T | C | 0.078 | -0.015 | 0.003 | 4.10E-08 | 30.124 |
| Pork intake | rs36124222 | C | T | 0.433 | 0.008 | 0.002 | 2.10E-08 | 31.395 |
| Pork intake | rs11211124 | C | T | 0.231 | -0.010 | 0.002 | 1.40E-08 | 32.195 |
| Pork intake | rs9973426 | G | A | 0.177 | 0.011 | 0.002 | 1.00E-08 | 32.756 |
| Pork intake | rs34161520 | G | C | 0.160 | 0.012 | 0.002 | 9.60E-09 | 32.912 |
| Pork intake | rs4146837 | T | C | 0.456 | 0.009 | 0.001 | 4.00E-09 | 34.615 |
| Pork intake | rs254152 | G | C | 0.235 | -0.010 | 0.002 | 2.20E-09 | 35.818 |
| Pork intake | rs3964074 | C | T | 0.547 | -0.009 | 0.001 | 1.60E-09 | 36.406 |
| Pork intake | rs10972033 | T | G | 0.456 | 0.009 | 0.001 | 1.30E-09 | 36.853 |
| Pork intake | rs12721051 | G | C | 0.188 | -0.012 | 0.002 | 5.60E-11 | 42.945 |
| Pork intake | rs9379832 | G | A | 0.255 | -0.011 | 0.002 | 1.80E-11 | 45.200 |
| Pork intake | rs838133 | G | A | 0.549 | 0.011 | 0.002 | 9.00E-13 | 51.041 |
| Pork intake | rs1355171 | A | C | 0.489 | -0.011 | 0.001 | 1.00E-13 | 55.296 |
| Coffee intake | rs62064918 | T | C | 0.245 | -0.010 | 0.002 | 4.10E-08 | 30.101 |
| Coffee intake | rs7811609 | T | C | 0.375 | 0.009 | 0.002 | 4.00E-08 | 30.137 |
| Coffee intake | rs1942965 | C | T | 0.505 | -0.009 | 0.002 | 3.80E-08 | 30.236 |
| Coffee intake | rs12989746 | T | G | 0.250 | 0.010 | 0.002 | 2.80E-08 | 30.821 |
| Coffee intake | rs75347775 | A | G | 0.245 | 0.010 | 0.002 | 2.70E-08 | 30.933 |
| Coffee intake | rs2597805 | T | C | 0.682 | 0.010 | 0.002 | 2.00E-08 | 31.489 |
| Coffee intake | rs6469262 | C | T | 0.565 | -0.009 | 0.002 | 1.90E-08 | 31.576 |
| Coffee intake | rs117968677 | A | G | 0.024 | -0.031 | 0.006 | 1.90E-08 | 31.645 |
| Coffee intake | rs1527961 | C | T | 0.135 | -0.013 | 0.002 | 1.70E-08 | 31.808 |
| Coffee intake | rs10119174 | C | G | 0.571 | -0.009 | 0.002 | 1.00E-08 | 32.774 |
| Coffee intake | rs57918684 | A | G | 0.155 | 0.013 | 0.002 | 8.60E-09 | 33.141 |
| Coffee intake | rs117810762 | A | G | 0.018 | 0.036 | 0.006 | 6.20E-09 | 33.775 |
| Coffee intake | rs1338549 | G | T | 0.534 | -0.009 | 0.002 | 5.60E-09 | 33.961 |
| Coffee intake | rs13054099 | C | T | 0.261 | -0.011 | 0.002 | 4.30E-09 | 34.460 |
| Coffee intake | rs516636 | A | C | 0.209 | 0.012 | 0.002 | 4.00E-09 | 34.632 |
| Coffee intake | rs78267637 | G | C | 0.038 | -0.025 | 0.004 | 3.90E-09 | 34.695 |
| Coffee intake | rs442355 | C | G | 0.254 | -0.011 | 0.002 | 1.90E-09 | 36.096 |
| Coffee intake | rs2189234 | G | T | 0.618 | 0.010 | 0.002 | 1.80E-09 | 36.172 |
| Coffee intake | rs8056750 | T | C | 0.359 | 0.011 | 0.002 | 1.30E-09 | 36.776 |
| Coffee intake | rs9398171 | T | C | 0.711 | 0.011 | 0.002 | 1.10E-09 | 37.210 |
| Coffee intake | rs2465037 | A | C | 0.343 | -0.011 | 0.002 | 4.80E-10 | 38.772 |
| Coffee intake | rs6063085 | C | A | 0.373 | 0.010 | 0.002 | 4.50E-10 | 38.899 |
| Coffee intake | rs6062682 | T | C | 0.465 | 0.010 | 0.002 | 2.50E-10 | 40.020 |
| Coffee intake | rs73075167 | T | A | 0.129 | -0.016 | 0.002 | 5.00E-11 | 43.191 |
| Coffee intake | rs4615895 | A | G | 0.741 | 0.012 | 0.002 | 4.20E-11 | 43.520 |
| Coffee intake | rs7224815 | T | A | 0.408 | -0.011 | 0.002 | 3.70E-11 | 43.765 |
| Coffee intake | rs17842490 | G | A | 0.014 | -0.045 | 0.007 | 3.30E-11 | 44.012 |
| Coffee intake | rs12514566 | A | G | 0.337 | -0.011 | 0.002 | 2.40E-11 | 44.651 |
| Coffee intake | rs630194 | C | T | 0.343 | -0.011 | 0.002 | 2.30E-11 | 44.678 |
| Coffee intake | rs13163336 | A | C | 0.158 | 0.015 | 0.002 | 1.30E-11 | 45.742 |
| Coffee intake | rs61928609 | C | A | 0.835 | -0.015 | 0.002 | 1.30E-11 | 45.854 |
| Coffee intake | rs13387939 | A | C | 0.828 | 0.017 | 0.002 | 9.80E-15 | 59.930 |
| Coffee intake | rs56113850 | C | T | 0.578 | 0.013 | 0.002 | 8.90E-15 | 60.129 |
| Coffee intake | rs34060476 | G | A | 0.134 | 0.018 | 0.002 | 7.50E-15 | 60.450 |
| Coffee intake | rs780093 | C | T | 0.616 | 0.013 | 0.002 | 1.00E-15 | 64.366 |
| Coffee intake | rs476828 | C | T | 0.237 | 0.017 | 0.002 | 5.60E-20 | 83.751 |
| Coffee intake | rs1057868 | T | C | 0.285 | 0.020 | 0.002 | 5.40E-29 | 124.901 |
| Coffee intake | rs1421085 | C | T | 0.404 | 0.019 | 0.002 | 1.70E-29 | 127.159 |
| Coffee intake | rs4410790 | C | T | 0.632 | 0.039 | 0.002 | 1.20E-120 | 545.509 |
| Coffee intake | rs2472297 | T | C | 0.263 | 0.046 | 0.002 | 1.10E-142 | 646.735 |
| Poultry intake | rs2426440 | G | A | 0.733 | 0.011 | 0.002 | 4.70E-08 | 29.849 |
| Poultry intake | rs2965200 | A | G | 0.640 | -0.010 | 0.002 | 4.20E-08 | 30.047 |
| Poultry intake | rs9997448 | T | C | 0.369 | -0.010 | 0.002 | 2.70E-08 | 30.889 |
| Poultry intake | rs1051730 | A | G | 0.331 | -0.011 | 0.002 | 1.70E-08 | 31.776 |
| Poultry intake | rs9923768 | A | G | 0.599 | 0.011 | 0.002 | 1.60E-08 | 31.977 |
| Poultry intake | rs2565017 | A | G | 0.373 | 0.011 | 0.002 | 5.90E-09 | 33.858 |
| Poultry intake | rs7829800 | G | A | 0.671 | 0.011 | 0.002 | 3.70E-09 | 34.781 |
| Poultry intake | rs7046351 | A | T | 0.510 | 0.011 | 0.002 | 1.10E-09 | 37.131 |
| Cheese intake | rs6685323 | T | C | 0.309 | -0.013 | 0.002 | 4.80E-08 | 29.801 |
| Cheese intake | rs12475594 | G | A | 0.178 | 0.016 | 0.003 | 4.40E-08 | 29.945 |
| Cheese intake | rs2802530 | A | G | 0.877 | 0.019 | 0.003 | 4.20E-08 | 30.065 |
| Cheese intake | rs1806771 | G | T | 0.088 | -0.022 | 0.004 | 4.10E-08 | 30.125 |
| Cheese intake | rs6873324 | C | A | 0.426 | -0.012 | 0.002 | 3.90E-08 | 30.190 |
| Cheese intake | rs7386207 | T | C | 0.564 | -0.012 | 0.002 | 3.60E-08 | 30.330 |
| Cheese intake | rs11620149 | C | T | 0.143 | -0.018 | 0.003 | 3.60E-08 | 30.345 |
| Cheese intake | rs78876700 | A | G | 0.137 | 0.018 | 0.003 | 3.40E-08 | 30.467 |
| Cheese intake | rs6126641 | A | G | 0.336 | 0.013 | 0.002 | 3.30E-08 | 30.498 |
| Cheese intake | rs4681981 | A | C | 0.469 | -0.012 | 0.002 | 2.90E-08 | 30.790 |
| Cheese intake | rs6774906 | C | A | 0.041 | 0.032 | 0.006 | 2.50E-08 | 31.090 |
| Cheese intake | rs73335955 | C | T | 0.053 | 0.028 | 0.005 | 2.40E-08 | 31.123 |
| Cheese intake | rs26579 | C | G | 0.586 | -0.013 | 0.002 | 2.40E-08 | 31.130 |
| Cheese intake | rs524468 | G | A | 0.261 | -0.014 | 0.003 | 2.40E-08 | 31.154 |
| Cheese intake | rs4860341 | C | T | 0.929 | 0.024 | 0.004 | 2.20E-08 | 31.351 |
| Cheese intake | rs17115145 | T | C | 0.401 | -0.013 | 0.002 | 1.80E-08 | 31.650 |
| Cheese intake | rs10938397 | G | A | 0.434 | -0.013 | 0.002 | 1.80E-08 | 31.684 |
| Cheese intake | rs531358 | T | C | 0.650 | 0.013 | 0.002 | 1.80E-08 | 31.733 |
| Cheese intake | rs1931805 | C | T | 0.500 | 0.013 | 0.002 | 1.60E-08 | 31.940 |
| Cheese intake | rs4503172 | T | C | 0.608 | 0.013 | 0.002 | 1.60E-08 | 31.943 |
| Cheese intake | rs61953351 | T | G | 0.250 | 0.015 | 0.003 | 1.50E-08 | 31.994 |
| Cheese intake | rs9504123 | C | A | 0.275 | 0.014 | 0.003 | 1.50E-08 | 32.030 |
| Cheese intake | rs62245792 | A | T | 0.150 | -0.018 | 0.003 | 1.40E-08 | 32.145 |
| Cheese intake | rs1024853 | G | C | 0.438 | -0.013 | 0.002 | 1.30E-08 | 32.327 |
| Cheese intake | rs4692708 | C | A | 0.253 | 0.015 | 0.003 | 1.30E-08 | 32.368 |
| Cheese intake | rs4296548 | G | T | 0.610 | 0.013 | 0.002 | 1.20E-08 | 32.410 |
| Cheese intake | rs12786959 | T | A | 0.196 | -0.016 | 0.003 | 1.20E-08 | 32.481 |
| Cheese intake | rs7298331 | C | A | 0.605 | -0.013 | 0.002 | 1.10E-08 | 32.646 |
| Cheese intake | rs71386942 | A | C | 0.269 | 0.014 | 0.003 | 9.90E-09 | 32.851 |
| Cheese intake | rs77742462 | G | A | 0.021 | -0.047 | 0.008 | 9.80E-09 | 32.879 |
| Cheese intake | rs1434511 | T | C | 0.455 | 0.013 | 0.002 | 9.50E-09 | 32.946 |
| Cheese intake | rs12672200 | A | G | 0.326 | -0.014 | 0.002 | 9.00E-09 | 33.037 |
| Cheese intake | rs12447542 | A | G | 0.126 | 0.020 | 0.003 | 6.80E-09 | 33.596 |
| Cheese intake | rs79184944 | A | T | 0.134 | 0.020 | 0.003 | 2.40E-09 | 35.615 |
| Cheese intake | rs11649653 | G | C | 0.382 | 0.014 | 0.002 | 1.50E-09 | 36.517 |
| Cheese intake | rs35270670 | G | A | 0.218 | 0.016 | 0.003 | 1.50E-09 | 36.538 |
| Cheese intake | rs62034322 | A | G | 0.380 | -0.014 | 0.002 | 1.40E-09 | 36.715 |
| Cheese intake | rs9649582 | T | A | 0.317 | -0.015 | 0.002 | 1.40E-09 | 36.737 |
| Cheese intake | rs113367286 | T | C | 0.278 | 0.015 | 0.002 | 1.30E-09 | 36.872 |
| Cheese intake | rs2339928 | A | G | 0.704 | 0.015 | 0.002 | 1.20E-09 | 36.929 |
| Cheese intake | rs67238148 | T | G | 0.217 | 0.017 | 0.003 | 1.10E-09 | 37.146 |
| Cheese intake | rs919109 | C | G | 0.139 | 0.020 | 0.003 | 7.90E-10 | 37.792 |
| Cheese intake | rs3911016 | G | T | 0.121 | 0.021 | 0.003 | 5.30E-10 | 38.548 |
| Cheese intake | rs34198643 | T | C | 0.224 | -0.017 | 0.003 | 4.50E-10 | 38.874 |
| Cheese intake | rs1514755 | G | A | 0.240 | 0.016 | 0.003 | 3.90E-10 | 39.166 |
| Cheese intake | rs12296440 | A | G | 0.170 | 0.019 | 0.003 | 2.80E-10 | 39.779 |
| Cheese intake | rs13257887 | C | T | 0.359 | 0.016 | 0.003 | 2.70E-10 | 39.892 |
| Cheese intake | rs61734410 | T | C | 0.255 | 0.017 | 0.003 | 2.20E-10 | 40.319 |
| Cheese intake | rs2352974 | T | C | 0.490 | -0.014 | 0.002 | 1.00E-10 | 41.782 |
| Cheese intake | rs10896050 | T | G | 0.193 | -0.018 | 0.003 | 7.20E-11 | 42.461 |
| Cheese intake | rs72970243 | A | G | 0.120 | 0.022 | 0.003 | 6.70E-11 | 42.596 |
| Cheese intake | rs73024305 | C | G | 0.055 | 0.033 | 0.005 | 4.00E-11 | 43.608 |
| Cheese intake | rs2854175 | A | C | 0.257 | 0.017 | 0.003 | 3.70E-11 | 43.772 |
| Cheese intake | rs4776970 | T | A | 0.358 | 0.015 | 0.002 | 3.50E-11 | 43.854 |
| Cheese intake | rs73096946 | C | T | 0.157 | -0.021 | 0.003 | 1.90E-11 | 45.086 |
| Cheese intake | rs62236533 | A | G | 0.109 | 0.025 | 0.004 | 1.10E-11 | 46.105 |
| Cheese intake | rs13107325 | T | C | 0.075 | -0.029 | 0.004 | 7.00E-12 | 47.025 |
| Cheese intake | rs1073242 | A | G | 0.554 | 0.016 | 0.002 | 6.70E-12 | 47.107 |
| Cheese intake | rs12951057 | G | C | 0.166 | -0.021 | 0.003 | 3.60E-12 | 48.310 |
| Cheese intake | rs7936836 | A | C | 0.418 | 0.016 | 0.002 | 2.60E-12 | 48.962 |
| Cheese intake | rs975303 | G | A | 0.181 | 0.021 | 0.003 | 2.50E-13 | 53.564 |
| Cheese intake | rs2960578 | G | T | 0.496 | 0.017 | 0.002 | 2.60E-14 | 57.999 |
| Cheese intake | rs7012814 | A | G | 0.474 | -0.019 | 0.002 | 2.10E-16 | 67.490 |
| Cheese intake | rs1291145 | C | T | 0.686 | -0.020 | 0.002 | 4.40E-17 | 70.583 |
| Cheese intake | rs504675 | T | C | 0.353 | 0.027 | 0.002 | 1.00E-31 | 137.315 |
| Dried fruit intake | rs261809 | G | A | 0.541 | -0.010 | 0.002 | 9.80E-09 | 32.886 |
| Dried fruit intake | rs11586016 | C | G | 0.371 | 0.010 | 0.002 | 1.10E-08 | 32.590 |
| Dried fruit intake | rs12137234 | T | C | 0.304 | 0.010 | 0.002 | 2.80E-08 | 30.847 |
| Dried fruit intake | rs72720396 | G | A | 0.229 | 0.011 | 0.002 | 8.70E-09 | 33.123 |
| Dried fruit intake | rs11811826 | A | T | 0.224 | 0.013 | 0.002 | 4.40E-11 | 43.425 |
| Dried fruit intake | rs3101339 | C | A | 0.603 | 0.014 | 0.002 | 6.20E-17 | 69.908 |
| Dried fruit intake | rs75641275 | C | A | 0.143 | -0.014 | 0.002 | 2.90E-09 | 35.250 |
| Dried fruit intake | rs7582086 | T | G | 0.468 | -0.010 | 0.002 | 8.80E-09 | 33.089 |
| Dried fruit intake | rs7599488 | T | C | 0.426 | -0.010 | 0.002 | 6.70E-10 | 38.104 |
| Dried fruit intake | rs4149513 | A | G | 0.494 | 0.012 | 0.002 | 2.20E-12 | 49.254 |
| Dried fruit intake | rs17184707 | T | C | 0.213 | -0.011 | 0.002 | 2.10E-08 | 31.434 |
| Dried fruit intake | rs4269101 | G | T | 0.719 | -0.014 | 0.002 | 1.10E-13 | 55.173 |
| Dried fruit intake | rs11720884 | G | A | 0.250 | 0.011 | 0.002 | 7.60E-09 | 33.362 |
| Dried fruit intake | rs57499472 | C | T | 0.404 | 0.010 | 0.002 | 8.10E-09 | 33.262 |
| Dried fruit intake | rs10026792 | A | G | 0.290 | 0.011 | 0.002 | 3.90E-09 | 34.662 |
| Dried fruit intake | rs1648404 | T | C | 0.476 | 0.009 | 0.002 | 1.80E-08 | 31.655 |
| Dried fruit intake | rs746868 | G | C | 0.615 | -0.013 | 0.002 | 5.20E-14 | 56.658 |
| Dried fruit intake | rs9385269 | T | C | 0.525 | 0.012 | 0.002 | 7.20E-13 | 51.482 |
| Dried fruit intake | rs2328887 | C | T | 0.899 | 0.019 | 0.003 | 8.80E-12 | 46.588 |
| Dried fruit intake | rs2533273 | A | C | 0.485 | -0.010 | 0.002 | 3.90E-09 | 34.680 |
| Dried fruit intake | rs7808471 | C | T | 0.322 | -0.012 | 0.002 | 1.10E-10 | 41.720 |
| Dried fruit intake | rs11772627 | C | G | 0.182 | 0.018 | 0.002 | 3.00E-17 | 71.316 |
| Dried fruit intake | rs7829800 | G | A | 0.671 | -0.010 | 0.002 | 5.10E-09 | 34.169 |
| Dried fruit intake | rs10740991 | C | G | 0.718 | 0.017 | 0.002 | 2.00E-19 | 81.233 |
| Dried fruit intake | rs7916868 | T | A | 0.503 | 0.010 | 0.002 | 9.10E-09 | 33.030 |
| Dried fruit intake | rs893856 | A | G | 0.149 | -0.013 | 0.002 | 1.30E-08 | 32.346 |
| Dried fruit intake | rs10896126 | G | A | 0.304 | -0.015 | 0.002 | 1.60E-16 | 68.069 |
| Dried fruit intake | rs11037497 | C | G | 0.446 | 0.010 | 0.002 | 5.70E-10 | 38.412 |
| Dried fruit intake | rs1622515 | G | A | 0.485 | 0.010 | 0.002 | 2.90E-09 | 35.233 |
| Dried fruit intake | rs3764002 | T | C | 0.261 | 0.013 | 0.002 | 5.10E-12 | 47.653 |
| Dried fruit intake | rs4140799 | A | G | 0.532 | 0.009 | 0.002 | 1.80E-08 | 31.743 |
| Dried fruit intake | rs34162196 | T | C | 0.101 | -0.022 | 0.003 | 7.10E-16 | 65.098 |
| Dried fruit intake | rs10129747 | G | A | 0.530 | 0.009 | 0.002 | 2.60E-08 | 30.988 |
| Dried fruit intake | rs1797235 | C | G | 0.375 | -0.010 | 0.002 | 8.90E-09 | 33.076 |
| Dried fruit intake | rs11632215 | C | A | 0.120 | -0.014 | 0.003 | 4.40E-08 | 29.963 |
| Dried fruit intake | rs862227 | G | A | 0.458 | -0.009 | 0.002 | 4.30E-08 | 30.030 |
| Dried fruit intake | rs1582322 | G | A | 0.605 | 0.010 | 0.002 | 6.80E-09 | 33.588 |
| Dried fruit intake | rs62084586 | C | T | 0.166 | 0.013 | 0.002 | 3.20E-09 | 35.073 |
| Dried fruit intake | rs8081370 | T | C | 0.910 | -0.017 | 0.003 | 1.40E-08 | 32.181 |
| Dried fruit intake | rs4800488 | A | C | 0.490 | 0.012 | 0.002 | 7.70E-13 | 51.368 |
| Dried fruit intake | rs17175518 | A | C | 0.233 | 0.011 | 0.002 | 5.90E-09 | 33.878 |
| Dried fruit intake | rs11152349 | A | G | 0.303 | 0.010 | 0.002 | 4.90E-08 | 29.740 |
| Dried fruit intake | rs429358 | C | T | 0.154 | 0.020 | 0.002 | 6.70E-18 | 74.313 |
| Processed meat intake | rs4077924 | C | T | 0.702 | 0.012 | 0.002 | 4.50E-08 | 29.928 |
| Processed meat intake | rs8096167 | C | T | 0.193 | -0.015 | 0.003 | 4.70E-08 | 29.856 |
| Processed meat intake | rs3762621 | T | C | 0.183 | -0.015 | 0.003 | 3.60E-08 | 30.377 |
| Processed meat intake | rs11887120 | T | C | 0.398 | 0.012 | 0.002 | 3.10E-08 | 30.657 |
| Processed meat intake | rs6786550 | C | T | 0.635 | 0.012 | 0.002 | 2.10E-08 | 31.423 |
| Processed meat intake | rs6765179 | A | G | 0.310 | -0.013 | 0.002 | 1.80E-08 | 31.709 |
| Processed meat intake | rs4778053 | G | C | 0.844 | 0.016 | 0.003 | 1.30E-08 | 32.270 |
| Processed meat intake | rs11894162 | T | C | 0.547 | 0.012 | 0.002 | 1.10E-08 | 32.702 |
| Processed meat intake | rs6010651 | C | A | 0.379 | -0.012 | 0.002 | 1.10E-08 | 32.746 |
| Processed meat intake | rs6961970 | A | C | 0.245 | -0.014 | 0.002 | 9.50E-09 | 32.951 |
| Processed meat intake | rs10454812 | C | A | 0.103 | -0.020 | 0.003 | 6.70E-09 | 33.626 |
| Processed meat intake | rs1422192 | A | G | 0.158 | 0.017 | 0.003 | 3.40E-09 | 34.923 |
| Processed meat intake | rs34241936 | G | A | 0.037 | 0.033 | 0.006 | 1.10E-08 | 32.574 |
| Processed meat intake | rs77165542 | T | C | 0.035 | 0.034 | 0.006 | 3.30E-09 | 35.018 |
| Processed meat intake | rs11032380 | T | A | 0.333 | -0.013 | 0.002 | 2.10E-09 | 35.920 |
| Processed meat intake | rs203319 | T | C | 0.205 | -0.016 | 0.003 | 2.80E-10 | 39.842 |
| Processed meat intake | rs9809856 | G | A | 0.476 | 0.013 | 0.002 | 2.50E-10 | 40.056 |
| Processed meat intake | rs2873054 | C | A | 0.353 | 0.014 | 0.002 | 1.60E-10 | 40.847 |
| Processed meat intake | rs6484504 | C | T | 0.725 | 0.015 | 0.002 | 4.40E-11 | 43.429 |
| Processed meat intake | rs7531118 | C | T | 0.531 | -0.014 | 0.002 | 2.80E-11 | 44.332 |
| Processed meat intake | rs2029401 | G | A | 0.586 | 0.015 | 0.002 | 6.30E-12 | 47.220 |
| Processed meat intake | rs4240672 | A | G | 0.494 | 0.017 | 0.002 | 3.00E-16 | 66.836 |
| Processed meat intake | rs838133 | G | A | 0.549 | 0.019 | 0.002 | 1.60E-18 | 77.090 |
| Salad/raw vegetable intake | rs9427220 | T | A | 0.555 | -0.008 | 0.001 | 2.80E-08 | 30.835 |
| Salad/raw vegetable intake | rs4083969 | G | C | 0.057 | 0.017 | 0.003 | 3.80E-08 | 30.248 |
| Salad/raw vegetable intake | rs7619139 | A | T | 0.589 | 0.012 | 0.001 | 8.00E-18 | 73.949 |
| Salad/raw vegetable intake | rs13102393 | G | C | 0.499 | 0.008 | 0.001 | 2.40E-08 | 31.123 |
| Salad/raw vegetable intake | rs17460017 | T | A | 0.190 | 0.011 | 0.002 | 7.20E-10 | 37.973 |
| Salad/raw vegetable intake | rs2194027 | A | T | 0.485 | -0.009 | 0.001 | 2.00E-09 | 35.950 |
| Salad/raw vegetable intake | rs3129962 | A | G | 0.129 | -0.013 | 0.002 | 3.70E-10 | 39.281 |
| Salad/raw vegetable intake | rs12203592 | T | C | 0.219 | -0.010 | 0.002 | 1.30E-09 | 36.866 |
| Salad/raw vegetable intake | rs3095337 | C | G | 0.204 | -0.013 | 0.002 | 9.00E-13 | 51.060 |
| Salad/raw vegetable intake | rs75248709 | T | C | 0.046 | -0.020 | 0.004 | 2.20E-08 | 31.292 |
| Salad/raw vegetable intake | rs57221424 | G | C | 0.322 | 0.009 | 0.002 | 5.50E-09 | 34.004 |
| Salad/raw vegetable intake | rs62461186 | C | A | 0.180 | -0.011 | 0.002 | 1.00E-09 | 37.303 |
| Salad/raw vegetable intake | rs790561 | G | A | 0.704 | 0.012 | 0.002 | 1.40E-15 | 63.776 |
| Salad/raw vegetable intake | rs7821179 | C | G | 0.847 | -0.011 | 0.002 | 4.40E-08 | 29.961 |
| Salad/raw vegetable intake | rs10819082 | A | G | 0.667 | -0.009 | 0.002 | 1.40E-09 | 36.671 |
| Salad/raw vegetable intake | rs6482190 | G | A | 0.719 | 0.011 | 0.002 | 1.40E-12 | 50.242 |
| Salad/raw vegetable intake | rs1890012 | G | T | 0.195 | -0.010 | 0.002 | 8.10E-09 | 33.255 |
| Salad/raw vegetable intake | rs12908495 | A | C | 0.243 | -0.009 | 0.002 | 2.00E-08 | 31.508 |
| Salad/raw vegetable intake | rs1052352 | T | C | 0.524 | 0.008 | 0.001 | 1.00E-08 | 32.797 |
| Salad/raw vegetable intake | rs34186148 | C | G | 0.370 | -0.008 | 0.001 | 4.80E-08 | 29.800 |
| Salad/raw vegetable intake | rs4291983 | A | C | 0.518 | -0.008 | 0.001 | 3.70E-09 | 34.756 |
| Salad/raw vegetable intake | rs8130508 | A | G | 0.290 | 0.009 | 0.002 | 3.00E-08 | 30.681 |
| Average weekly red wine intake | rs35698271 | C | A | 0.180 | -0.015 | 0.003 | 1.30E-08 | 32.386 |
| Average weekly red wine intake | rs10925183 | A | G | 0.607 | -0.012 | 0.002 | 1.60E-08 | 31.874 |
| Average weekly red wine intake | rs1446577 | G | C | 0.256 | 0.015 | 0.002 | 1.50E-10 | 41.079 |
| Average weekly red wine intake | rs12692596 | T | C | 0.371 | -0.012 | 0.002 | 4.50E-08 | 29.934 |
| Average weekly red wine intake | rs11714337 | A | G | 0.433 | 0.012 | 0.002 | 2.90E-09 | 35.218 |
| Average weekly red wine intake | rs11715683 | A | T | 0.345 | 0.014 | 0.002 | 1.30E-10 | 41.358 |
| Average weekly red wine intake | rs4643716 | A | C | 0.584 | 0.012 | 0.002 | 4.30E-08 | 30.002 |
| Average weekly red wine intake | rs1229984 | C | T | 0.977 | 0.061 | 0.007 | 1.50E-19 | 81.856 |
| Average weekly red wine intake | rs6882046 | G | A | 0.269 | 0.016 | 0.002 | 4.10E-12 | 48.073 |
| Average weekly red wine intake | rs6908328 | A | C | 0.514 | 0.013 | 0.002 | 7.20E-10 | 37.974 |
| Average weekly red wine intake | rs9388171 | G | C | 0.522 | 0.015 | 0.002 | 1.80E-12 | 49.700 |
| Average weekly red wine intake | rs713598 | G | C | 0.400 | -0.017 | 0.002 | 1.50E-16 | 68.222 |
| Average weekly red wine intake | rs55968191 | A | G | 0.247 | 0.013 | 0.002 | 4.70E-08 | 29.855 |
| Average weekly red wine intake | rs62573521 | T | C | 0.040 | -0.032 | 0.005 | 7.50E-10 | 37.888 |
| Average weekly red wine intake | rs10822129 | T | C | 0.407 | 0.013 | 0.002 | 2.70E-10 | 39.874 |
| Average weekly red wine intake | rs17817497 | C | T | 0.392 | 0.014 | 0.002 | 2.60E-11 | 44.426 |
| Average weekly red wine intake | rs898751 | T | C | 0.492 | -0.012 | 0.002 | 2.10E-09 | 35.870 |
| Average weekly red wine intake | rs303753 | A | G | 0.345 | -0.018 | 0.002 | 6.00E-17 | 69.989 |
| Average weekly red wine intake | rs627685 | C | T | 0.303 | -0.013 | 0.002 | 3.00E-08 | 30.738 |
| Fish/liver oil dietary supplements | rs1344459 | T | C | 0.755 | -0.005 | 0.001 | 4.80E-06 | 20.910 |
| Fish/liver oil dietary supplements | rs6668468 | A | G | 0.205 | -0.005 | 0.001 | 4.80E-06 | 20.919 |
| Fish/liver oil dietary supplements | rs55983260 | C | T | 0.305 | 0.005 | 0.001 | 4.60E-06 | 21.000 |
| Fish/liver oil dietary supplements | rs4698039 | G | T | 0.934 | 0.009 | 0.002 | 4.50E-06 | 21.051 |
| Fish/liver oil dietary supplements | rs141833759 | T | C | 0.147 | 0.006 | 0.001 | 4.40E-06 | 21.089 |
| Fish/liver oil dietary supplements | rs17597661 | T | G | 0.047 | -0.011 | 0.002 | 4.40E-06 | 21.093 |
| Fish/liver oil dietary supplements | rs11898568 | A | G | 0.288 | -0.005 | 0.001 | 4.30E-06 | 21.113 |
| Fish/liver oil dietary supplements | rs4554183 | C | T | 0.825 | 0.006 | 0.001 | 3.60E-06 | 21.451 |
| Fish/liver oil dietary supplements | rs12880503 | T | A | 0.516 | -0.004 | 0.001 | 3.40E-06 | 21.573 |
| Fish/liver oil dietary supplements | rs2764688 | G | A | 0.821 | -0.006 | 0.001 | 3.40E-06 | 21.580 |
| Fish/liver oil dietary supplements | rs11110 | T | C | 0.296 | -0.005 | 0.001 | 3.00E-06 | 21.786 |
| Fish/liver oil dietary supplements | rs56305307 | C | A | 0.212 | -0.006 | 0.001 | 2.90E-06 | 21.864 |
| Fish/liver oil dietary supplements | rs75649946 | C | A | 0.342 | 0.005 | 0.001 | 2.80E-06 | 21.926 |
| Fish/liver oil dietary supplements | rs116453686 | A | G | 0.015 | -0.019 | 0.004 | 2.70E-06 | 22.043 |
| Fish/liver oil dietary supplements | rs17756220 | G | A | 0.068 | 0.009 | 0.002 | 2.40E-06 | 22.247 |
| Fish/liver oil dietary supplements | rs906343 | A | G | 0.809 | 0.006 | 0.001 | 2.30E-06 | 22.327 |
| Fish/liver oil dietary supplements | rs9579775 | C | A | 0.136 | -0.007 | 0.001 | 2.20E-06 | 22.384 |
| Fish/liver oil dietary supplements | rs2192530 | G | C | 0.523 | 0.005 | 0.001 | 2.00E-06 | 22.573 |
| Fish/liver oil dietary supplements | rs143029017 | C | G | 0.059 | -0.010 | 0.002 | 1.60E-06 | 23.010 |
| Fish/liver oil dietary supplements | rs2126266 | T | C | 0.314 | 0.005 | 0.001 | 1.60E-06 | 23.075 |
| Fish/liver oil dietary supplements | rs4146426 | T | A | 0.044 | -0.011 | 0.002 | 1.30E-06 | 23.372 |
| Fish/liver oil dietary supplements | rs61937595 | T | C | 0.094 | 0.008 | 0.002 | 1.30E-06 | 23.477 |
| Fish/liver oil dietary supplements | rs35295103 | T | C | 0.192 | -0.006 | 0.001 | 1.10E-06 | 23.729 |
| Fish/liver oil dietary supplements | rs9365169 | C | G | 0.501 | 0.005 | 0.001 | 1.10E-06 | 23.741 |
| Fish/liver oil dietary supplements | rs11737459 | A | T | 0.697 | -0.005 | 0.001 | 1.10E-06 | 23.747 |
| Fish/liver oil dietary supplements | rs12308147 | A | C | 0.156 | 0.007 | 0.001 | 9.90E-07 | 23.941 |
| Fish/liver oil dietary supplements | rs71311699 | T | G | 0.512 | -0.005 | 0.001 | 9.60E-07 | 24.003 |
| Fish/liver oil dietary supplements | rs116572724 | G | C | 0.032 | 0.014 | 0.003 | 8.50E-07 | 24.239 |
| Fish/liver oil dietary supplements | rs9366658 | A | G | 0.190 | -0.006 | 0.001 | 8.40E-07 | 24.267 |
| Fish/liver oil dietary supplements | rs10267346 | C | T | 0.124 | 0.007 | 0.001 | 8.20E-07 | 24.316 |
| Fish/liver oil dietary supplements | rs12585257 | C | G | 0.334 | -0.005 | 0.001 | 6.00E-07 | 24.903 |
| Fish/liver oil dietary supplements | rs7657731 | T | G | 0.298 | -0.005 | 0.001 | 4.20E-07 | 25.600 |
| Fish/liver oil dietary supplements | rs808903 | T | C | 0.763 | -0.006 | 0.001 | 4.10E-07 | 25.644 |
| Fish/liver oil dietary supplements | rs7598185 | A | C | 0.304 | -0.006 | 0.001 | 1.90E-07 | 27.154 |
| Fish/liver oil dietary supplements | rs10995566 | T | C | 0.327 | -0.005 | 0.001 | 1.30E-07 | 27.822 |
| Fish/liver oil dietary supplements | rs6027709 | G | A | 0.541 | -0.005 | 0.001 | 9.90E-08 | 28.384 |
| Fish/liver oil dietary supplements | rs9870832 | C | T | 0.426 | 0.006 | 0.001 | 4.20E-09 | 34.506 |
| Fish/liver oil dietary supplements | rs4861024 | G | A | 0.239 | -0.007 | 0.001 | 2.50E-09 | 35.502 |
| Fish/liver oil dietary supplements | rs2533273 | A | C | 0.484 | -0.006 | 0.001 | 5.00E-10 | 38.674 |
| Fish/liver oil dietary supplements | rs3789045 | T | C | 0.209 | 0.009 | 0.001 | 6.40E-13 | 51.717 |
| Body mass index | rs543874 | G | A | 0.267 | 0.048 | 0.004 | 2.62E-35 | 152.744 |
| Body mass index | rs11165643 | T | C | 0.575 | 0.022 | 0.003 | 2.07E-12 | 49.453 |
| Body mass index | rs17024393 | C | T | 0.042 | 0.066 | 0.009 | 7.03E-14 | 55.910 |
| Body mass index | rs3101336 | C | T | 0.649 | 0.033 | 0.003 | 2.66E-26 | 116.083 |
| Body mass index | rs2820292 | C | A | 0.508 | 0.020 | 0.003 | 1.83E-10 | 39.568 |
| Body mass index | rs657452 | G | A | 0.583 | -0.023 | 0.003 | 5.48E-13 | 53.620 |
| Body mass index | rs6656785 | G | A | 0.383 | 0.022 | 0.003 | 3.83E-12 | 49.000 |
| Body mass index | rs1528435 | T | C | 0.583 | 0.018 | 0.003 | 1.20E-08 | 32.970 |
| Body mass index | rs7599312 | A | G | 0.292 | -0.022 | 0.003 | 1.17E-10 | 41.869 |
| Body mass index | rs10182181 | G | A | 0.500 | 0.031 | 0.003 | 8.78E-24 | 98.074 |
| Body mass index | rs13021737 | G | A | 0.875 | 0.060 | 0.004 | 1.11E-50 | 225.751 |
| Body mass index | rs2121279 | T | C | 0.117 | 0.025 | 0.004 | 2.31E-08 | 31.005 |
| Body mass index | rs12986742 | C | T | 0.500 | 0.021 | 0.004 | 1.01E-08 | 32.830 |
| Body mass index | rs1016287 | C | T | 0.675 | -0.023 | 0.003 | 2.25E-11 | 45.364 |
| Body mass index | rs2365389 | T | C | 0.342 | -0.020 | 0.003 | 1.63E-10 | 41.623 |
| Body mass index | rs3849570 | A | C | 0.367 | 0.019 | 0.003 | 2.60E-08 | 30.574 |
| Body mass index | rs13078960 | G | T | 0.183 | 0.030 | 0.004 | 1.74E-14 | 57.994 |
| Body mass index | rs16851483 | T | G | 0.092 | 0.048 | 0.008 | 3.55E-10 | 39.347 |
| Body mass index | rs6804842 | G | A | 0.575 | 0.019 | 0.003 | 2.48E-09 | 35.614 |
| Body mass index | rs1516725 | C | T | 0.908 | 0.045 | 0.005 | 1.89E-22 | 96.125 |
| Body mass index | rs13107325 | T | C | 0.117 | 0.048 | 0.007 | 1.83E-12 | 49.206 |
| Body mass index | rs11727676 | C | T | 0.075 | -0.036 | 0.006 | 2.55E-08 | 31.290 |
| Body mass index | rs10938397 | G | A | 0.433 | 0.040 | 0.003 | 3.20E-38 | 168.162 |
| Body mass index | rs17001654 | G | C | 0.158 | 0.031 | 0.005 | 7.76E-09 | 33.334 |
| Body mass index | rs2112347 | G | T | 0.375 | -0.026 | 0.003 | 6.19E-17 | 70.886 |
| Body mass index | rs13191362 | G | A | 0.200 | -0.028 | 0.005 | 7.34E-09 | 33.303 |
| Body mass index | rs2033529 | G | A | 0.258 | 0.019 | 0.003 | 1.39E-08 | 33.150 |
| Body mass index | rs9400239 | C | T | 0.700 | 0.019 | 0.003 | 1.61E-08 | 32.455 |
| Body mass index | rs205262 | G | A | 0.267 | 0.022 | 0.004 | 1.75E-10 | 39.870 |
| Body mass index | rs2207139 | G | A | 0.100 | 0.045 | 0.004 | 4.13E-29 | 124.881 |
| Body mass index | rs1167827 | G | A | 0.542 | 0.020 | 0.003 | 6.33E-10 | 37.469 |
| Body mass index | rs2245368 | T | C | 0.758 | -0.032 | 0.006 | 3.19E-08 | 30.929 |
| Body mass index | rs17405819 | C | T | 0.367 | -0.022 | 0.003 | 2.07E-11 | 46.075 |
| Body mass index | rs2033732 | C | T | 0.758 | 0.019 | 0.004 | 4.89E-08 | 30.093 |
| Body mass index | rs10968576 | G | A | 0.292 | 0.025 | 0.003 | 6.61E-14 | 56.934 |
| Body mass index | rs6477694 | T | C | 0.642 | -0.017 | 0.003 | 2.67E-08 | 31.505 |
| Body mass index | rs1928295 | C | T | 0.425 | -0.019 | 0.003 | 7.91E-10 | 36.778 |
| Body mass index | rs4740619 | C | T | 0.467 | -0.018 | 0.003 | 4.56E-09 | 33.341 |
| Body mass index | rs10733682 | G | A | 0.575 | -0.017 | 0.003 | 1.83E-08 | 31.505 |
| Body mass index | rs7903146 | T | C | 0.250 | -0.023 | 0.003 | 1.11E-11 | 47.367 |
| Body mass index | rs7899106 | G | A | 0.050 | 0.040 | 0.007 | 2.96E-08 | 30.951 |
| Body mass index | rs17094222 | C | T | 0.208 | 0.025 | 0.004 | 5.94E-11 | 42.937 |
| Body mass index | rs2176598 | C | T | 0.800 | -0.020 | 0.004 | 2.97E-08 | 30.250 |
| Body mass index | rs4256980 | G | C | 0.725 | 0.021 | 0.003 | 2.90E-11 | 45.454 |
| Body mass index | rs3817334 | T | C | 0.450 | 0.026 | 0.003 | 5.15E-17 | 71.430 |
| Body mass index | rs12286929 | G | A | 0.433 | 0.022 | 0.003 | 1.31E-12 | 49.000 |
| Body mass index | rs11030104 | G | A | 0.200 | -0.041 | 0.004 | 5.56E-28 | 118.695 |
| Body mass index | rs11057405 | A | G | 0.092 | -0.031 | 0.006 | 2.02E-08 | 31.157 |
| Body mass index | rs7138803 | A | G | 0.442 | 0.032 | 0.003 | 8.15E-24 | 103.252 |
| Body mass index | rs12429545 | A | G | 0.100 | 0.033 | 0.005 | 1.09E-12 | 50.501 |
| Body mass index | rs9579083 | C | G | 0.233 | 0.030 | 0.005 | 3.46E-10 | 39.396 |
| Body mass index | rs10132280 | A | C | 0.333 | -0.023 | 0.003 | 1.14E-11 | 45.761 |
| Body mass index | rs7141420 | T | C | 0.617 | 0.024 | 0.003 | 1.23E-14 | 57.466 |
| Body mass index | rs16951275 | C | T | 0.225 | -0.031 | 0.004 | 1.91E-17 | 70.651 |
| Body mass index | rs3736485 | G | A | 0.575 | -0.018 | 0.003 | 7.41E-09 | 32.233 |
| Body mass index | rs879620 | T | C | 0.592 | 0.024 | 0.004 | 1.06E-09 | 37.210 |
| Body mass index | rs758747 | T | C | 0.267 | 0.023 | 0.004 | 7.47E-10 | 36.980 |
| Body mass index | rs9926784 | C | T | 0.208 | -0.027 | 0.004 | 1.85E-10 | 39.810 |
| Body mass index | rs3888190 | A | C | 0.358 | 0.031 | 0.003 | 3.14E-23 | 99.356 |
| Body mass index | rs4889606 | G | A | 0.358 | -0.018 | 0.003 | 4.86E-09 | 34.848 |
| Body mass index | rs1558902 | A | T | 0.450 | 0.082 | 0.003 | 7.52E-153 | 696.279 |
| Body mass index | rs12940622 | A | G | 0.458 | -0.018 | 0.003 | 2.49E-09 | 34.468 |
| Body mass index | rs1000940 | G | A | 0.225 | 0.019 | 0.003 | 1.28E-08 | 31.889 |
| Body mass index | rs1808579 | T | C | 0.475 | -0.017 | 0.003 | 4.17E-08 | 29.021 |
| Body mass index | rs6567160 | C | T | 0.283 | 0.056 | 0.004 | 3.93E-53 | 238.531 |
| Body mass index | rs17066856 | C | T | 0.133 | -0.040 | 0.006 | 6.22E-13 | 51.579 |
| Body mass index | rs29941 | G | A | 0.667 | 0.018 | 0.003 | 2.41E-08 | 30.417 |
| Body mass index | rs17724992 | G | A | 0.308 | -0.019 | 0.004 | 3.41E-08 | 30.723 |
| Body mass index | rs2287019 | T | C | 0.150 | -0.036 | 0.004 | 4.58E-18 | 73.469 |
| Waist-to-hip ratio | rs1011731 | A | G | 0.542 | -0.019 | 0.003 | 1.10E-08 | 33.150 |
| Waist-to-hip ratio | rs1563355 | C | T | 0.686 | 0.031 | 0.004 | 1.70E-12 | 49.638 |
| Waist-to-hip ratio | rs2765539 | T | C | 0.708 | 0.027 | 0.004 | 1.10E-12 | 50.485 |
| Waist-to-hip ratio | rs929641 | G | A | 0.383 | -0.020 | 0.003 | 4.20E-09 | 36.731 |
| Waist-to-hip ratio | rs1128249 | T | G | 0.442 | -0.021 | 0.003 | 1.60E-09 | 38.149 |
| Waist-to-hip ratio | rs1569135 | G | A | 0.467 | -0.024 | 0.003 | 1.00E-12 | 52.893 |
| Waist-to-hip ratio | rs2972164 | C | T | 0.500 | 0.019 | 0.003 | 2.40E-08 | 33.150 |
| Waist-to-hip ratio | rs17451107 | C | T | 0.375 | -0.023 | 0.004 | 3.50E-11 | 43.184 |
| Waist-to-hip ratio | rs9860730 | G | A | 0.233 | -0.023 | 0.004 | 2.80E-10 | 40.818 |
| Waist-to-hip ratio | rs459193 | G | A | 0.783 | -0.026 | 0.004 | 6.00E-12 | 46.814 |
| Waist-to-hip ratio | rs4715213 | T | C | 0.100 | 0.024 | 0.004 | 4.00E-08 | 29.752 |
| Waist-to-hip ratio | rs9491696 | G | C | 0.525 | 0.038 | 0.003 | 4.90E-30 | 132.599 |
| Waist-to-hip ratio | rs998584 | A | C | 0.475 | 0.029 | 0.004 | 5.00E-15 | 61.432 |
| Waist-to-hip ratio | rs1294421 | G | T | 0.600 | 0.025 | 0.003 | 6.90E-14 | 54.066 |
| Waist-to-hip ratio | rs10245353 | A | C | 0.183 | 0.027 | 0.004 | 1.60E-10 | 41.327 |
| Waist-to-hip ratio | rs12549058 | G | T | 0.058 | 0.040 | 0.006 | 3.20E-10 | 39.063 |
| Waist-to-hip ratio | rs4929927 | G | A | 0.725 | 0.020 | 0.003 | 7.60E-09 | 34.602 |
| Waist-to-hip ratio | rs10783615 | A | G | 0.867 | -0.035 | 0.005 | 7.00E-13 | 51.020 |
| Waist-to-hip ratio | rs11048470 | T | G | 0.233 | 0.025 | 0.004 | 6.30E-12 | 45.654 |
| Waist-to-hip ratio | rs1316952 | C | T | 0.117 | -0.028 | 0.005 | 7.30E-09 | 32.653 |
| Waist-to-hip ratio | rs17109256 | A | G | 0.275 | 0.023 | 0.004 | 3.00E-08 | 31.469 |
| Waist-to-hip ratio | rs1440372 | C | T | 0.742 | 0.021 | 0.004 | 7.60E-09 | 32.213 |
| Waist-to-hip ratio | rs1121980 | A | G | 0.475 | 0.043 | 0.003 | 1.30E-38 | 169.789 |
| Waist-to-hip ratio | rs4640244 | G | A | 0.375 | 0.021 | 0.004 | 3.10E-08 | 32.213 |
| Waist-to-hip ratio | rs11663816 | C | T | 0.317 | 0.025 | 0.004 | 2.70E-11 | 43.283 |
| Waist-to-hip ratio | rs2287019 | T | C | 0.150 | -0.026 | 0.005 | 4.30E-09 | 33.383 |
| Waist-to-hip ratio | rs3786897 | G | A | 0.408 | 0.022 | 0.003 | 4.00E-11 | 41.869 |
| Waist-to-hip ratio | rs16996700 | C | T | 0.300 | -0.021 | 0.004 | 1.60E-08 | 32.213 |
| Waist-to-hip ratio | rs2179129 | G | A | 0.450 | -0.021 | 0.003 | 1.20E-09 | 38.149 |
| Hip circumference | rs6678622 | T | C | 0.417 | 0.022 | 0.004 | 1.10E-09 | 35.354 |
| Hip circumference | rs11165623 | A | G | 0.483 | 0.022 | 0.004 | 3.00E-10 | 39.510 |
| Hip circumference | rs12086130 | T | C | 0.100 | 0.038 | 0.006 | 2.60E-09 | 36.382 |
| Hip circumference | rs7531118 | C | T | 0.608 | 0.024 | 0.004 | 4.00E-11 | 42.075 |
| Hip circumference | rs17024393 | C | T | 0.042 | 0.063 | 0.010 | 2.80E-10 | 39.690 |
| Hip circumference | rs543874 | G | A | 0.267 | 0.045 | 0.005 | 1.80E-23 | 100.000 |
| Hip circumference | rs2820443 | C | T | 0.300 | 0.034 | 0.004 | 4.90E-18 | 76.003 |
| Hip circumference | rs10929925 | A | C | 0.350 | -0.019 | 0.004 | 4.50E-08 | 27.855 |
| Hip circumference | rs355838 | G | T | 0.633 | 0.022 | 0.004 | 1.60E-09 | 37.346 |
| Hip circumference | rs6755502 | C | T | 0.875 | 0.054 | 0.005 | 2.40E-30 | 132.005 |
| Hip circumference | rs11676272 | G | A | 0.483 | 0.026 | 0.004 | 3.80E-11 | 44.444 |
| Hip circumference | rs887912 | C | T | 0.683 | -0.022 | 0.004 | 1.60E-08 | 31.821 |
| Hip circumference | rs1561277 | A | C | 0.225 | -0.024 | 0.004 | 6.00E-09 | 34.265 |
| Hip circumference | rs4132228 | T | C | 0.233 | 0.021 | 0.004 | 3.10E-08 | 28.994 |
| Hip circumference | rs2301573 | C | T | 0.075 | 0.035 | 0.006 | 2.10E-08 | 31.868 |
| Hip circumference | rs13098327 | A | G | 0.183 | 0.027 | 0.004 | 1.20E-09 | 37.655 |
| Hip circumference | rs1516725 | C | T | 0.908 | 0.031 | 0.005 | 1.00E-08 | 32.956 |
| Hip circumference | rs7632381 | C | T | 0.483 | 0.036 | 0.004 | 5.30E-24 | 105.796 |
| Hip circumference | rs10938397 | G | A | 0.433 | 0.030 | 0.004 | 9.30E-17 | 65.741 |
| Hip circumference | rs951252 | G | A | 0.525 | -0.028 | 0.004 | 4.50E-15 | 64.000 |
| Hip circumference | rs2112347 | G | T | 0.375 | -0.025 | 0.004 | 9.70E-12 | 48.225 |
| Hip circumference | rs16894959 | C | T | 0.100 | 0.037 | 0.005 | 9.60E-14 | 57.018 |
| Hip circumference | rs2206277 | T | C | 0.092 | 0.039 | 0.005 | 3.10E-17 | 71.881 |
| Hip circumference | rs1294409 | T | C | 0.617 | -0.020 | 0.004 | 3.00E-08 | 29.218 |
| Hip circumference | rs806794 | G | A | 0.275 | -0.032 | 0.004 | 2.80E-16 | 64.000 |
| Hip circumference | rs6569648 | T | C | 0.758 | -0.029 | 0.004 | 6.50E-12 | 47.676 |
| Hip circumference | rs3800229 | T | G | 0.692 | 0.021 | 0.004 | 3.20E-08 | 30.540 |
| Hip circumference | rs798528 | C | A | 0.300 | -0.021 | 0.004 | 1.70E-08 | 30.540 |
| Hip circumference | rs1548457 | C | T | 0.375 | 0.025 | 0.005 | 1.70E-08 | 30.864 |
| Hip circumference | rs16905212 | C | T | 0.325 | -0.021 | 0.004 | 1.90E-08 | 30.540 |
| Hip circumference | rs6163 | A | C | 0.392 | 0.021 | 0.004 | 1.60E-08 | 32.213 |
| Hip circumference | rs7903146 | T | C | 0.250 | -0.026 | 0.004 | 2.10E-11 | 44.444 |
| Hip circumference | rs2293576 | A | G | 0.367 | -0.023 | 0.004 | 2.10E-09 | 36.634 |
| Hip circumference | rs6265 | T | C | 0.175 | -0.034 | 0.005 | 1.50E-14 | 57.086 |
| Hip circumference | rs7138803 | A | G | 0.442 | 0.029 | 0.004 | 1.90E-15 | 61.432 |
| Hip circumference | rs11057405 | A | G | 0.092 | -0.040 | 0.006 | 2.10E-10 | 40.312 |
| Hip circumference | rs1351394 | C | T | 0.517 | -0.023 | 0.004 | 6.40E-11 | 43.184 |
| Hip circumference | rs4883723 | A | G | 0.125 | 0.032 | 0.005 | 5.20E-10 | 39.369 |
| Hip circumference | rs10132280 | A | C | 0.333 | -0.022 | 0.004 | 4.80E-09 | 33.518 |
| Hip circumference | rs7144011 | T | G | 0.275 | 0.030 | 0.004 | 8.60E-13 | 51.020 |
| Hip circumference | rs7183263 | G | T | 0.475 | 0.019 | 0.004 | 4.80E-08 | 27.855 |
| Hip circumference | rs879620 | T | C | 0.592 | 0.029 | 0.005 | 8.90E-10 | 38.072 |
| Hip circumference | rs4889606 | G | A | 0.358 | -0.021 | 0.004 | 8.80E-09 | 34.028 |
| Hip circumference | rs9939973 | A | G | 0.475 | 0.070 | 0.004 | 2.30E-86 | 378.086 |
| Hip circumference | rs3888190 | A | C | 0.358 | 0.035 | 0.004 | 9.40E-22 | 94.522 |
| Hip circumference | rs3087591 | G | A | 0.375 | 0.021 | 0.004 | 2.50E-08 | 30.540 |
| Hip circumference | rs13695 | T | C | 0.275 | 0.024 | 0.004 | 4.30E-08 | 29.752 |
| Hip circumference | rs1808579 | T | C | 0.475 | -0.023 | 0.004 | 2.10E-10 | 43.184 |
| Hip circumference | rs663129 | A | G | 0.283 | 0.050 | 0.004 | 1.40E-32 | 141.723 |
| Hip circumference | rs17066856 | C | T | 0.133 | -0.036 | 0.006 | 6.30E-09 | 32.653 |
| Hip circumference | rs11672660 | T | C | 0.175 | -0.028 | 0.005 | 7.10E-10 | 38.716 |
| Hip circumference | rs143384 | G | A | 0.400 | 0.026 | 0.004 | 3.90E-12 | 46.814 |
| Waist circumference | rs7550711 | T | C | 0.034 | 0.058 | 0.010 | 3.40E-09 | 35.027 |
| Waist circumference | rs7531118 | C | T | 0.608 | 0.027 | 0.004 | 1.50E-14 | 59.510 |
| Waist circumference | rs3127553 | A | G | 0.633 | -0.023 | 0.004 | 1.60E-10 | 43.184 |
| Waist circumference | rs2820292 | C | A | 0.508 | 0.019 | 0.003 | 2.40E-08 | 31.228 |
| Waist circumference | rs17381664 | C | T | 0.425 | 0.022 | 0.004 | 4.20E-10 | 39.510 |
| Waist circumference | rs11165623 | A | G | 0.483 | 0.020 | 0.003 | 5.20E-09 | 34.602 |
| Waist circumference | rs633715 | C | T | 0.267 | 0.043 | 0.004 | 3.30E-23 | 100.000 |
| Waist circumference | rs6545714 | A | G | 0.625 | -0.022 | 0.004 | 1.90E-10 | 39.510 |
| Waist circumference | rs6755502 | C | T | 0.875 | 0.051 | 0.005 | 2.00E-30 | 128.444 |
| Waist circumference | rs929641 | G | A | 0.383 | -0.021 | 0.003 | 1.20E-09 | 38.149 |
| Waist circumference | rs3849570 | A | C | 0.367 | 0.021 | 0.004 | 2.20E-08 | 30.540 |
| Waist circumference | rs10767658 | G | C | 0.642 | -0.031 | 0.004 | 3.30E-17 | 70.197 |
| Waist circumference | rs7138803 | A | G | 0.442 | 0.028 | 0.004 | 1.60E-15 | 64.000 |
| Waist circumference | rs12429545 | A | G | 0.100 | 0.031 | 0.005 | 2.50E-09 | 35.540 |
| Waist circumference | rs1558902 | A | T | 0.450 | 0.074 | 0.004 | 3.70E-101 | 447.020 |
| Waist circumference | rs2531992 | G | A | 0.833 | 0.028 | 0.005 | 3.00E-09 | 34.028 |
| Waist circumference | rs7498665 | G | A | 0.358 | 0.034 | 0.004 | 1.40E-22 | 94.367 |
| Waist circumference | rs17066856 | C | T | 0.133 | -0.037 | 0.006 | 9.00E-10 | 38.028 |
| Waist circumference | rs6567160 | C | T | 0.283 | 0.048 | 0.004 | 2.60E-33 | 144.000 |
| Waist circumference | rs7239883 | A | G | 0.683 | -0.021 | 0.004 | 2.30E-09 | 36.000 |
| Waist circumference | rs2287019 | T | C | 0.150 | -0.035 | 0.005 | 1.70E-14 | 57.892 |
| Waist circumference | rs16996700 | C | T | 0.300 | -0.023 | 0.004 | 1.50E-09 | 38.641 |
| Essential hypertension | rs880315 | C | T | 0.4127 | 0.096 | 0.010 | 1.16E-21 | 91.203 |
| Essential hypertension | rs6668768 | T | C | 0.2141 | 0.071 | 0.012 | 3.89E-09 | 34.614 |
| Essential hypertension | rs10776752 | T | G | 0.1691 | 0.128 | 0.013 | 2.03E-22 | 95.025 |
| Essential hypertension | rs1317181 | T | G | 0.2096 | 0.073 | 0.012 | 1.43E-09 | 36.498 |
| Essential hypertension | rs76778513 | T | A | 0.1132 | -0.131 | 0.016 | 3.78E-17 | 70.625 |
| Essential hypertension | rs143439093 | G | A | 0.07434 | -0.148 | 0.019 | 4.51E-15 | 61.486 |
| Essential hypertension | rs34924059 | T | G | 0.1554 | 0.075 | 0.014 | 4.12E-08 | 30.088 |
| Essential hypertension | rs1275984 | C | A | 0.5499 | -0.090 | 0.010 | 8.79E-20 | 82.828 |
| Essential hypertension | rs2704368 | G | A | 0.8376 | -0.086 | 0.013 | 1.29E-10 | 41.617 |
| Essential hypertension | rs3906954 | T | C | 0.092 | 0.093 | 0.017 | 4.69E-08 | 29.863 |
| Essential hypertension | rs16998073 | T | A | 0.3113 | 0.108 | 0.011 | 5.01E-24 | 103.042 |
| Essential hypertension | rs149150643 | C | T | 0.04522 | -0.137 | 0.024 | 8.39E-09 | 33.329 |
| Essential hypertension | rs3796582 | C | T | 0.3279 | -0.064 | 0.010 | 8.39E-10 | 37.988 |
| Essential hypertension | rs12656497 | C | T | 0.5852 | 0.065 | 0.010 | 8.61E-11 | 42.579 |
| Essential hypertension | rs6860901 | T | C | 0.2971 | 0.064 | 0.011 | 2.95E-09 | 35.330 |
| Essential hypertension | rs62434119 | T | C | 0.0761 | -0.138 | 0.019 | 8.34E-14 | 55.967 |
| Essential hypertension | rs2032451 | T | G | 0.1097 | 0.091 | 0.016 | 7.74E-09 | 33.448 |
| Essential hypertension | rs2077111 | G | A | 0.4978 | 0.055 | 0.010 | 1.69E-08 | 31.957 |
| Essential hypertension | rs3918226 | T | C | 0.06939 | 0.157 | 0.019 | 6.35E-16 | 65.160 |
| Essential hypertension | rs71554083 | G | A | 0.9039 | 0.128 | 0.017 | 2.97E-14 | 57.544 |
| Essential hypertension | rs17477177 | C | T | 0.2975 | 0.068 | 0.011 | 1.92E-10 | 40.864 |
| Essential hypertension | rs72779268 | A | C | 0.0646 | -0.120 | 0.020 | 2.40E-09 | 35.465 |
| Essential hypertension | rs10786736 | C | G | 0.09206 | -0.109 | 0.017 | 1.13E-10 | 41.828 |
| Essential hypertension | rs2484294 | A | G | 0.7473 | 0.085 | 0.011 | 3.92E-14 | 57.116 |
| Essential hypertension | rs2274224 | C | G | 0.3465 | -0.064 | 0.010 | 6.84E-10 | 38.368 |
| Essential hypertension | rs7483477 | G | T | 0.2345 | 0.074 | 0.012 | 3.09E-10 | 39.787 |
| Essential hypertension | rs557675 | G | T | 0.3977 | -0.055 | 0.010 | 4.14E-08 | 30.360 |
| Essential hypertension | rs12828438 | G | A | 0.5356 | -0.064 | 0.010 | 9.47E-11 | 42.053 |
| Essential hypertension | rs7310615 | G | C | 0.5868 | -0.079 | 0.010 | 2.45E-15 | 62.252 |
| Essential hypertension | rs7134677 | T | C | 0.3829 | -0.063 | 0.010 | 4.42E-10 | 39.155 |
| Essential hypertension | rs506409 | T | C | 0.5794 | 0.061 | 0.010 | 9.87E-10 | 37.210 |
| Essential hypertension | rs303949 | C | A | 0.09928 | 0.098 | 0.016 | 2.40E-09 | 35.781 |
| Essential hypertension | rs1894400 | T | C | 0.2708 | 0.081 | 0.011 | 2.31E-13 | 53.382 |
| Essential hypertension | rs9930501 | G | A | 0.4243 | 0.062 | 0.010 | 5.65E-10 | 38.716 |
| Essential hypertension | rs149441293 | C | G | 0.04467 | 0.143 | 0.024 | 2.37E-09 | 35.749 |
| Essential hypertension | rs8068318 | T | C | 0.7215 | 0.060 | 0.011 | 3.50E-08 | 30.604 |
| Essential hypertension | rs145153053 | G | A | 0.196 | 0.069 | 0.012 | 2.45E-08 | 30.874 |
| Essential hypertension | rs78008010 | C | A | 0.04681 | -0.149 | 0.023 | 1.68E-10 | 40.675 |
| Essential hypertension | rs167479 | T | G | 0.4256 | -0.082 | 0.010 | 1.83E-16 | 67.568 |
| Essential hypertension | rs78412528 | A | G | 0.1663 | 0.123 | 0.013 | 8.54E-21 | 86.970 |
| HDL cholesterol levels | rs193084249 | G | A | 0.023 | -0.107 | 0.012 | 7.20E-18 | 74.173 |
| HDL cholesterol levels | rs4846921 | A | G | 0.613 | 0.049 | 0.004 | 3.80E-38 | 166.749 |
| HDL cholesterol levels | rs140584594 | G | A | 0.730 | 0.025 | 0.004 | 1.00E-09 | 37.253 |
| HDL cholesterol levels | rs61805076 | C | T | 0.334 | -0.029 | 0.004 | 9.20E-14 | 55.534 |
| HDL cholesterol levels | rs2642438 | G | A | 0.704 | 0.036 | 0.004 | 9.50E-19 | 78.150 |
| HDL cholesterol levels | rs3768321 | T | G | 0.197 | -0.042 | 0.005 | 1.50E-19 | 81.850 |
| HDL cholesterol levels | rs267738 | G | T | 0.220 | 0.028 | 0.004 | 5.90E-10 | 38.356 |
| HDL cholesterol levels | rs676210 | A | G | 0.206 | 0.070 | 0.005 | 1.00E-52 | 233.517 |
| HDL cholesterol levels | rs78058190 | A | G | 0.050 | -0.061 | 0.010 | 2.10E-10 | 40.366 |
| HDL cholesterol levels | rs2943650 | T | C | 0.645 | -0.041 | 0.004 | 3.60E-26 | 111.982 |
| HDL cholesterol levels | rs35633876 | T | G | 0.482 | -0.023 | 0.004 | 2.50E-10 | 40.000 |
| HDL cholesterol levels | rs13389219 | T | C | 0.393 | 0.034 | 0.004 | 4.60E-19 | 79.587 |
| HDL cholesterol levels | rs1047891 | A | C | 0.315 | -0.023 | 0.004 | 5.90E-09 | 33.858 |
| HDL cholesterol levels | rs71336055 | T | C | 0.120 | 0.034 | 0.006 | 3.30E-09 | 34.986 |
| HDL cholesterol levels | rs13107325 | T | C | 0.074 | -0.075 | 0.007 | 2.80E-26 | 112.513 |
| HDL cholesterol levels | rs11429307 | T | G | 0.192 | -0.040 | 0.005 | 1.30E-17 | 73.058 |
| HDL cholesterol levels | rs2307111 | C | T | 0.397 | 0.025 | 0.004 | 4.80E-11 | 43.257 |
| HDL cholesterol levels | rs9491697 | G | A | 0.464 | -0.027 | 0.004 | 1.90E-13 | 54.125 |
| HDL cholesterol levels | rs34265539 | T | A | 0.644 | 0.027 | 0.004 | 3.70E-12 | 48.253 |
| HDL cholesterol levels | rs1358980 | T | C | 0.483 | -0.033 | 0.004 | 1.30E-18 | 77.488 |
| HDL cholesterol levels | rs11751347 | T | C | 0.102 | -0.055 | 0.006 | 2.10E-19 | 81.189 |
| HDL cholesterol levels | rs9265559 | G | C | 0.351 | -0.029 | 0.004 | 2.00E-13 | 54.009 |
| HDL cholesterol levels | rs3735687 | G | A | 0.423 | -0.025 | 0.004 | 5.20E-11 | 43.104 |
| HDL cholesterol levels | rs35493868 | G | C | 0.204 | 0.034 | 0.005 | 1.80E-13 | 54.224 |
| HDL cholesterol levels | rs7810507 | A | G | 0.281 | -0.030 | 0.004 | 2.40E-13 | 53.641 |
| HDL cholesterol levels | rs10268632 | C | A | 0.512 | 0.022 | 0.004 | 5.30E-09 | 34.090 |
| HDL cholesterol levels | rs12533197 | G | T | 0.439 | -0.021 | 0.004 | 1.40E-08 | 32.248 |
| HDL cholesterol levels | rs11434143 | GT | G | 0.189 | 0.026 | 0.005 | 3.50E-08 | 30.395 |
| HDL cholesterol levels | rs7845090 | A | G | 0.709 | 0.024 | 0.004 | 8.00E-09 | 33.281 |
| HDL cholesterol levels | rs2245221 | A | G | 0.559 | 0.031 | 0.004 | 8.60E-17 | 69.268 |
| HDL cholesterol levels | rs2954029 | T | A | 0.465 | 0.023 | 0.004 | 4.60E-10 | 38.827 |
| HDL cholesterol levels | rs4240624 | A | G | 0.909 | 0.093 | 0.006 | 1.80E-47 | 209.437 |
| HDL cholesterol levels | rs1561748 | C | G | 0.267 | 0.060 | 0.004 | 4.30E-41 | 180.252 |
| HDL cholesterol levels | rs328 | G | C | 0.100 | 0.157 | 0.006 | 6.90E-146 | 661.560 |
| HDL cholesterol levels | rs71205961 | T | C | 0.911 | 0.043 | 0.007 | 4.20E-09 | 34.519 |
| HDL cholesterol levels | rs2066714 | C | T | 0.129 | 0.034 | 0.005 | 6.10E-10 | 38.288 |
| HDL cholesterol levels | rs1215112 | A | G | 0.863 | 0.049 | 0.005 | 4.30E-20 | 84.297 |
| HDL cholesterol levels | rs11789603 | T | C | 0.109 | 0.053 | 0.006 | 6.30E-19 | 78.986 |
| HDL cholesterol levels | rs2740488 | C | A | 0.265 | -0.044 | 0.004 | 1.30E-25 | 109.372 |
| HDL cholesterol levels | rs148063610 | T | C | 0.237 | 0.034 | 0.004 | 1.40E-14 | 59.228 |
| HDL cholesterol levels | rs2792735 | A | G | 0.720 | -0.040 | 0.004 | 1.80E-22 | 95.106 |
| HDL cholesterol levels | rs60847460 | T | C | 0.143 | -0.036 | 0.005 | 6.30E-12 | 47.234 |
| HDL cholesterol levels | rs12786130 | T | C | 0.762 | 0.027 | 0.004 | 6.10E-10 | 38.292 |
| HDL cholesterol levels | rs1047964 | T | G | 0.010 | 0.203 | 0.019 | 3.20E-27 | 116.799 |
| HDL cholesterol levels | rs12295878 | T | C | 0.140 | 0.033 | 0.005 | 5.60E-10 | 38.450 |
| HDL cholesterol levels | rs35184771 | T | G | 0.353 | -0.039 | 0.004 | 1.00E-23 | 100.760 |
| HDL cholesterol levels | rs964184 | C | G | 0.867 | 0.087 | 0.005 | 6.40E-58 | 257.369 |
| HDL cholesterol levels | rs559355 | T | A | 0.158 | -0.052 | 0.005 | 3.00E-24 | 103.208 |
| HDL cholesterol levels | rs144018203 | C | G | 0.011 | -0.175 | 0.019 | 6.80E-21 | 87.931 |
| HDL cholesterol levels | rs116978226 | A | C | 0.033 | 0.065 | 0.010 | 3.80E-10 | 39.189 |
| HDL cholesterol levels | rs141949189 | T | A | 0.432 | -0.024 | 0.004 | 8.90E-10 | 37.550 |
| HDL cholesterol levels | rs174578 | A | T | 0.351 | -0.065 | 0.004 | 5.60E-64 | 285.172 |
| HDL cholesterol levels | rs2229357 | A | G | 0.241 | 0.028 | 0.004 | 4.00E-11 | 43.596 |
| HDL cholesterol levels | rs7308864 | G | A | 0.523 | 0.026 | 0.004 | 1.20E-12 | 50.468 |
| HDL cholesterol levels | rs17696736 | G | A | 0.430 | -0.022 | 0.004 | 6.50E-09 | 33.673 |
| HDL cholesterol levels | rs142265900 | T | A | 0.058 | 0.053 | 0.008 | 2.90E-11 | 44.222 |
| HDL cholesterol levels | rs921919 | A | G | 0.670 | -0.042 | 0.004 | 5.60E-26 | 111.096 |
| HDL cholesterol levels | rs7136506 | C | T | 0.216 | -0.041 | 0.005 | 6.40E-19 | 78.935 |
| HDL cholesterol levels | rs7134375 | A | C | 0.430 | 0.023 | 0.004 | 2.80E-10 | 39.832 |
| HDL cholesterol levels | rs1054852 | G | A | 0.377 | 0.032 | 0.004 | 1.80E-15 | 63.319 |
| HDL cholesterol levels | rs11057692 | G | A | 0.236 | -0.026 | 0.004 | 5.40E-09 | 34.038 |
| HDL cholesterol levels | rs4078216 | A | G | 0.241 | 0.026 | 0.004 | 2.30E-09 | 35.720 |
| HDL cholesterol levels | rs941507 | C | G | 0.461 | -0.020 | 0.004 | 4.50E-08 | 29.943 |
| HDL cholesterol levels | rs1601935 | T | G | 0.655 | -0.098 | 0.004 | 1.10E-138 | 628.407 |
| HDL cholesterol levels | rs147233090 | T | C | 0.025 | -0.076 | 0.012 | 2.40E-10 | 40.097 |
| HDL cholesterol levels | rs10162642 | A | G | 0.210 | -0.041 | 0.005 | 4.90E-19 | 79.449 |
| HDL cholesterol levels | rs2070895 | A | G | 0.219 | 0.108 | 0.004 | 1.70E-128 | 581.619 |
| HDL cholesterol levels | rs75714888 | A | C | 0.022 | -0.080 | 0.013 | 3.80E-10 | 39.198 |
| HDL cholesterol levels | rs3764261 | A | C | 0.324 | 0.208 | 0.004 | 1.00E-200 | 2806.377 |
| HDL cholesterol levels | rs8058512 | T | C | 0.724 | -0.027 | 0.004 | 5.90E-11 | 42.862 |
| HDL cholesterol levels | rs2925979 | C | T | 0.699 | 0.038 | 0.004 | 2.20E-21 | 90.116 |
| HDL cholesterol levels | rs4985155 | G | A | 0.335 | -0.024 | 0.004 | 1.10E-09 | 37.171 |
| HDL cholesterol levels | rs75911530 | A | G | 0.033 | -0.158 | 0.011 | 2.00E-48 | 213.816 |
| HDL cholesterol levels | rs4986970 | T | A | 0.034 | -0.093 | 0.010 | 1.00E-19 | 82.617 |
| HDL cholesterol levels | rs12103674 | C | T | 0.080 | -0.038 | 0.007 | 2.40E-08 | 31.145 |
| HDL cholesterol levels | rs72836561 | T | C | 0.031 | -0.156 | 0.011 | 1.80E-49 | 218.676 |
| HDL cholesterol levels | rs4969141 | T | C | 0.490 | 0.023 | 0.004 | 4.10E-10 | 39.066 |
| HDL cholesterol levels | rs112001035 | A | G | 0.060 | -0.058 | 0.008 | 3.70E-13 | 52.809 |
| HDL cholesterol levels | rs77960347 | G | A | 0.013 | 0.253 | 0.016 | 1.50E-55 | 246.536 |
| HDL cholesterol levels | rs688671 | G | A | 0.267 | -0.025 | 0.004 | 4.40E-09 | 34.440 |
| HDL cholesterol levels | rs12608026 | G | T | 0.042 | 0.072 | 0.009 | 5.30E-15 | 61.161 |
| HDL cholesterol levels | rs7241918 | T | G | 0.823 | 0.076 | 0.005 | 3.50E-55 | 244.818 |
| HDL cholesterol levels | rs116843064 | A | G | 0.020 | 0.215 | 0.013 | 2.70E-59 | 263.699 |
| HDL cholesterol levels | rs737337 | C | T | 0.077 | -0.060 | 0.007 | 3.00E-18 | 75.878 |
| HDL cholesterol levels | rs75627662 | T | C | 0.206 | -0.033 | 0.005 | 7.00E-13 | 51.553 |
| HDL cholesterol levels | rs5167 | G | T | 0.352 | 0.044 | 0.004 | 6.60E-30 | 129.040 |
| HDL cholesterol levels | rs1761457 | G | A | 0.327 | 0.028 | 0.004 | 1.10E-12 | 50.669 |
| HDL cholesterol levels | rs150224153 | T | C | 0.029 | -0.091 | 0.011 | 7.30E-16 | 65.051 |
| HDL cholesterol levels | rs6018652 | A | G | 0.793 | 0.036 | 0.005 | 5.40E-15 | 61.099 |
| HDL cholesterol levels | rs6073958 | C | T | 0.199 | -0.074 | 0.005 | 7.80E-57 | 252.386 |
| HDL cholesterol levels | rs2298428 | T | C | 0.183 | -0.037 | 0.005 | 1.60E-14 | 58.976 |
| LDL cholesterol levels | rs11591147 | T | G | 0.017 | -0.303 | 0.016 | 1.80E-83 | 374.682 |
| LDL cholesterol levels | rs11206517 | G | T | 0.033 | 0.096 | 0.012 | 9.10E-17 | 69.150 |
| LDL cholesterol levels | rs7534572 | G | C | 0.647 | 0.030 | 0.004 | 1.60E-12 | 49.928 |
| LDL cholesterol levels | rs12740374 | T | G | 0.221 | -0.117 | 0.005 | 4.20E-124 | 561.372 |
| LDL cholesterol levels | rs2495477 | G | A | 0.394 | -0.032 | 0.004 | 3.80E-14 | 57.250 |
| LDL cholesterol levels | rs553427 | T | C | 0.517 | 0.033 | 0.004 | 9.80E-16 | 64.473 |
| LDL cholesterol levels | rs140798831 | C | T | 0.657 | -0.029 | 0.004 | 1.50E-11 | 45.594 |
| LDL cholesterol levels | rs562338 | G | A | 0.820 | 0.106 | 0.005 | 4.10E-87 | 391.381 |
| LDL cholesterol levels | rs11127048 | A | G | 0.614 | -0.041 | 0.004 | 3.00E-21 | 89.545 |
| LDL cholesterol levels | rs4299376 | T | G | 0.676 | -0.047 | 0.004 | 3.20E-26 | 112.234 |
| LDL cholesterol levels | rs59950280 | A | G | 0.331 | 0.025 | 0.004 | 9.30E-09 | 32.984 |
| LDL cholesterol levels | rs7707394 | A | G | 0.357 | 0.037 | 0.004 | 4.60E-18 | 75.051 |
| LDL cholesterol levels | rs6882345 | A | G | 0.633 | 0.035 | 0.004 | 3.40E-16 | 66.580 |
| LDL cholesterol levels | rs1895233 | T | G | 0.545 | 0.025 | 0.004 | 2.20E-09 | 35.829 |
| LDL cholesterol levels | rs1499279 | G | T | 0.042 | -0.068 | 0.010 | 4.60E-11 | 43.331 |
| LDL cholesterol levels | rs3846662 | G | A | 0.424 | 0.054 | 0.004 | 5.80E-39 | 170.475 |
| LDL cholesterol levels | rs115740542 | C | T | 0.074 | -0.061 | 0.008 | 1.20E-14 | 59.529 |
| LDL cholesterol levels | rs140145058 | G | A | 0.135 | 0.049 | 0.006 | 6.90E-16 | 65.159 |
| LDL cholesterol levels | rs3777411 | T | C | 0.151 | -0.041 | 0.006 | 1.30E-12 | 50.288 |
| LDL cholesterol levels | rs10953298 | T | C | 0.236 | -0.027 | 0.005 | 2.60E-08 | 31.020 |
| LDL cholesterol levels | rs1461729 | G | A | 0.899 | 0.061 | 0.007 | 4.30E-19 | 79.722 |
| LDL cholesterol levels | rs112875651 | A | G | 0.392 | -0.055 | 0.004 | 4.50E-38 | 166.420 |
| LDL cholesterol levels | rs2740488 | C | A | 0.265 | -0.033 | 0.005 | 1.80E-12 | 49.639 |
| LDL cholesterol levels | rs115478735 | T | A | 0.183 | 0.061 | 0.005 | 2.70E-30 | 130.803 |
| LDL cholesterol levels | rs59379014 | T | C | 0.073 | 0.062 | 0.008 | 4.40E-15 | 61.505 |
| LDL cholesterol levels | rs525028 | A | G | 0.709 | -0.036 | 0.005 | 3.00E-15 | 62.243 |
| LDL cholesterol levels | rs102275 | C | T | 0.350 | -0.050 | 0.004 | 8.00E-32 | 137.822 |
| LDL cholesterol levels | rs1805776 | C | T | 0.226 | 0.029 | 0.005 | 3.30E-09 | 35.015 |
| LDL cholesterol levels | rs4766578 | A | T | 0.503 | 0.026 | 0.004 | 1.70E-10 | 40.815 |
| LDL cholesterol levels | rs35596121 | CT | C | 0.288 | -0.026 | 0.005 | 2.90E-08 | 30.780 |
| LDL cholesterol levels | rs11621792 | T | C | 0.454 | 0.028 | 0.004 | 1.40E-11 | 45.619 |
| LDL cholesterol levels | rs261290 | C | T | 0.655 | -0.024 | 0.004 | 1.60E-08 | 31.919 |
| LDL cholesterol levels | rs34042070 | G | C | 0.187 | 0.043 | 0.005 | 8.90E-16 | 64.659 |
| LDL cholesterol levels | rs55714927 | T | C | 0.191 | -0.037 | 0.005 | 1.20E-12 | 50.489 |
| LDL cholesterol levels | rs77542162 | G | A | 0.023 | 0.119 | 0.014 | 6.30E-18 | 74.437 |
| LDL cholesterol levels | rs72631343 | G | C | 0.129 | -0.038 | 0.006 | 5.40E-10 | 38.522 |
| LDL cholesterol levels | rs56325564 | A | G | 0.483 | 0.033 | 0.004 | 6.90E-16 | 65.150 |
| LDL cholesterol levels | rs12151108 | A | G | 0.120 | -0.145 | 0.006 | 2.00E-117 | 530.646 |
| LDL cholesterol levels | rs2738447 | C | A | 0.593 | 0.030 | 0.004 | 3.90E-13 | 52.716 |
| LDL cholesterol levels | rs28807203 | C | A | 0.049 | -0.133 | 0.010 | 4.60E-44 | 193.829 |
| LDL cholesterol levels | rs58542926 | T | C | 0.074 | -0.102 | 0.008 | 6.10E-39 | 170.377 |
| LDL cholesterol levels | rs111278137 | A | G | 0.022 | -0.136 | 0.015 | 1.20E-20 | 86.883 |
| LDL cholesterol levels | rs8103315 | A | C | 0.136 | 0.058 | 0.006 | 2.50E-21 | 89.929 |
| LDL cholesterol levels | rs10410835 | C | T | 0.523 | 0.055 | 0.004 | 4.00E-40 | 175.818 |
| LDL cholesterol levels | rs1081105 | C | A | 0.028 | 0.166 | 0.013 | 5.30E-40 | 175.254 |
| LDL cholesterol levels | rs1065853 | T | G | 0.081 | -0.509 | 0.008 | 1.00E-200 | 4531.458 |
| LDL cholesterol levels | rs1883711 | C | G | 0.031 | 0.091 | 0.012 | 4.00E-14 | 57.171 |
| LDL cholesterol levels | rs4810479 | T | C | 0.750 | -0.029 | 0.005 | 5.40E-10 | 38.511 |
| Type 2 diabetes mellitus | rs2943656 | G | A | 0.617 | 0.076 | 0.011 | 6.40E-12 | 47.374 |
| Type 2 diabetes mellitus | rs13389219 | T | C | 0.348 | -0.071 | 0.011 | 6.19E-10 | 38.353 |
| Type 2 diabetes mellitus | rs144155527 | T | C | 0.024 | -0.217 | 0.036 | 1.75E-09 | 36.200 |
| Type 2 diabetes mellitus | rs34872471 | C | T | 0.201 | 0.305 | 0.014 | 4.47E-109 | 494.657 |
| Type 2 diabetes mellitus | rs182788819 | T | C | 0.039 | 0.158 | 0.028 | 1.96E-08 | 31.456 |
| Type 2 diabetes mellitus | rs73541184 | A | G | 0.309 | -0.086 | 0.012 | 2.10E-13 | 54.406 |
| Type 2 diabetes mellitus | rs10743152 | C | T | 0.662 | -0.066 | 0.011 | 9.32E-09 | 33.214 |
| Type 2 diabetes mellitus | rs2237897 | T | C | 0.081 | -0.198 | 0.020 | 6.76E-23 | 97.233 |
| Type 2 diabetes mellitus | rs5215 | T | C | 0.529 | -0.062 | 0.011 | 1.12E-08 | 32.850 |
| Type 2 diabetes mellitus | rs10830963 | G | C | 0.357 | 0.132 | 0.011 | 2.35E-31 | 135.630 |
| Type 2 diabetes mellitus | rs112108223 | A | G | 0.022 | -0.371 | 0.038 | 1.75E-22 | 95.268 |
| Type 2 diabetes mellitus | rs78470967 | A | T | 0.039 | -0.243 | 0.029 | 1.69E-17 | 72.459 |
| Type 2 diabetes mellitus | rs74862545 | T | C | 0.020 | -0.258 | 0.041 | 1.85E-10 | 40.582 |
| Type 2 diabetes mellitus | rs76895963 | G | T | 0.031 | -0.507 | 0.034 | 1.51E-50 | 223.586 |
| Type 2 diabetes mellitus | rs73113806 | T | C | 0.023 | 0.255 | 0.036 | 1.22E-12 | 50.374 |
| Type 2 diabetes mellitus | rs1798085 | C | T | 0.558 | -0.060 | 0.011 | 3.58E-08 | 30.099 |
| Type 2 diabetes mellitus | rs56348580 | C | G | 0.283 | -0.079 | 0.012 | 8.39E-11 | 42.089 |
| Type 2 diabetes mellitus | rs7998259 | A | G | 0.390 | -0.077 | 0.011 | 5.56E-12 | 47.143 |
| Type 2 diabetes mellitus | rs28553330 | C | T | 0.060 | -0.128 | 0.023 | 2.18E-08 | 31.389 |
| Type 2 diabetes mellitus | rs12449219 | G | C | 0.058 | 0.131 | 0.023 | 1.84E-08 | 31.738 |
| Type 2 diabetes mellitus | rs9940128 | A | G | 0.429 | 0.118 | 0.011 | 4.75E-27 | 116.402 |
| Type 2 diabetes mellitus | rs55993634 | G | C | 0.087 | -0.165 | 0.019 | 2.10E-17 | 71.987 |
| Type 2 diabetes mellitus | rs7224685 | T | G | 0.302 | 0.064 | 0.012 | 4.19E-08 | 30.297 |
| Type 2 diabetes mellitus | rs11263763 | A | G | 0.646 | -0.067 | 0.011 | 4.31E-09 | 34.633 |
| Type 2 diabetes mellitus | rs12967878 | C | T | 0.181 | 0.077 | 0.014 | 3.91E-08 | 30.250 |
| Type 2 diabetes mellitus | rs2303700 | C | T | 0.674 | -0.068 | 0.012 | 5.75E-09 | 33.860 |
| Type 2 diabetes mellitus | rs8100204 | A | G | 0.160 | 0.098 | 0.015 | 5.73E-11 | 42.819 |
| Type 2 diabetes mellitus | rs429358 | C | T | 0.183 | -0.081 | 0.014 | 1.09E-08 | 32.780 |
| Type 2 diabetes mellitus | rs7507893 | A | G | 0.497 | -0.061 | 0.011 | 1.91E-08 | 31.422 |
| Type 2 diabetes mellitus | rs45551238 | T | C | 0.050 | -0.230 | 0.025 | 1.17E-19 | 82.286 |
| Type 2 diabetes mellitus | rs77735929 | A | T | 0.043 | 0.176 | 0.027 | 6.69E-11 | 42.710 |
| Type 2 diabetes mellitus | rs117657619 | A | G | 0.048 | -0.141 | 0.026 | 4.41E-08 | 30.037 |
| Type 2 diabetes mellitus | rs6017317 | G | T | 0.219 | 0.077 | 0.013 | 4.35E-09 | 34.370 |
| Type 2 diabetes mellitus | rs8353 | T | G | 0.301 | -0.074 | 0.012 | 2.94E-10 | 39.754 |
| Vigorous physical activity | rs6667222 | C | A | 0.252 | -0.009 | 0.002 | 8.70E-09 | 33.104 |
| Vigorous physical activity | rs1248860 | A | G | 0.516 | 0.010 | 0.001 | 1.10E-13 | 55.256 |
| Vigorous physical activity | rs9276758 | A | G | 0.312 | -0.008 | 0.001 | 1.40E-08 | 32.126 |
| Vigorous physical activity | rs2764261 | G | A | 0.626 | -0.009 | 0.001 | 2.00E-11 | 44.996 |
| Vigorous physical activity | rs13243553 | A | G | 0.392 | -0.009 | 0.001 | 9.00E-11 | 42.021 |
| Vigorous physical activity | rs328902 | T | C | 0.315 | 0.009 | 0.001 | 5.50E-10 | 38.479 |
| Vigorous physical activity | rs3781411 | T | C | 0.124 | -0.013 | 0.002 | 3.00E-10 | 39.670 |
| Strenuous sports or other exercises | rs1200154 | A | G | 0.593 | 0.006 | 0.001 | 3.90E-08 | 30.207 |
| Strenuous sports or other exercises | rs2994326 | C | T | 0.813 | 0.008 | 0.001 | 4.50E-08 | 29.928 |
| Strenuous sports or other exercises | rs288070 | A | G | 0.098 | 0.011 | 0.002 | 1.90E-08 | 31.575 |
| Strenuous sports or other exercises | rs62253088 | C | T | 0.673 | -0.011 | 0.001 | 1.00E-19 | 82.574 |
| Strenuous sports or other exercises | rs7627864 | G | C | 0.431 | -0.007 | 0.001 | 7.60E-09 | 33.372 |
| Strenuous sports or other exercises | rs4865667 | T | C | 0.387 | -0.007 | 0.001 | 1.00E-08 | 32.771 |
| Strenuous sports or other exercises | rs159544 | G | A | 0.395 | 0.007 | 0.001 | 1.30E-09 | 36.759 |
| Strenuous sports or other exercises | rs10946808 | G | A | 0.273 | 0.008 | 0.001 | 9.90E-10 | 37.342 |
| Strenuous sports or other exercises | rs1265178 | A | G | 0.238 | -0.007 | 0.001 | 3.20E-08 | 30.576 |
| Strenuous sports or other exercises | rs896302 | T | C | 0.713 | -0.007 | 0.001 | 1.70E-08 | 31.759 |
| Strenuous sports or other exercises | rs4411372 | C | T | 0.280 | 0.007 | 0.001 | 2.00E-08 | 31.459 |
| Strenuous sports or other exercises | rs75930676 | C | T | 0.051 | 0.016 | 0.003 | 2.00E-09 | 36.013 |
| Strenuous sports or other exercises | rs166840 | A | G | 0.412 | -0.008 | 0.001 | 3.10E-11 | 44.131 |
| Strenuous sports or other exercises | rs111901094 | T | G | 0.182 | -0.009 | 0.001 | 3.00E-09 | 35.160 |
| Moderate to vigorous physical activity | rs2942127 | A | G | 0.825 | -0.016 | 0.003 | 3.30E-08 | 30.522 |
| Moderate to vigorous physical activity | rs1974771 | A | G | 0.100 | 0.021 | 0.004 | 6.60E-09 | 33.654 |
| Moderate to vigorous physical activity | rs2114286 | G | A | 0.534 | 0.012 | 0.002 | 3.30E-08 | 30.501 |
| Moderate to vigorous physical activity | rs877483 | C | T | 0.567 | -0.012 | 0.002 | 4.00E-08 | 30.132 |
| Moderate to vigorous physical activity | rs2035562 | G | A | 0.672 | 0.014 | 0.002 | 3.90E-09 | 34.688 |
| Moderate to vigorous physical activity | rs1972763 | T | C | 0.658 | -0.013 | 0.002 | 3.30E-08 | 30.526 |
| Moderate to vigorous physical activity | rs77742115 | C | T | 0.138 | 0.018 | 0.003 | 9.60E-09 | 32.922 |
| Moderate to vigorous physical activity | rs2854277 | T | C | 0.083 | -0.032 | 0.005 | 2.60E-10 | 39.960 |
| Moderate to vigorous physical activity | rs7804463 | C | T | 0.470 | -0.015 | 0.002 | 1.20E-11 | 45.988 |
| Moderate to vigorous physical activity | rs921915 | C | T | 0.588 | 0.014 | 0.002 | 5.70E-10 | 38.437 |
| Moderate to vigorous physical activity | rs1186721 | A | G | 0.316 | 0.013 | 0.002 | 4.40E-08 | 29.984 |
| Moderate to vigorous physical activity | rs1043595 | A | G | 0.283 | -0.014 | 0.002 | 4.30E-09 | 34.481 |
| Moderate to vigorous physical activity | rs2988004 | G | T | 0.442 | 0.013 | 0.002 | 4.10E-09 | 34.579 |
| Moderate to vigorous physical activity | rs7326482 | T | G | 0.615 | 0.013 | 0.002 | 1.60E-08 | 31.915 |
| Moderate to vigorous physical activity | rs10145335 | A | G | 0.251 | 0.014 | 0.003 | 2.70E-08 | 30.878 |
| Moderate to vigorous physical activity | rs12912808 | T | C | 0.149 | -0.018 | 0.003 | 1.70E-08 | 31.853 |
| Moderate to vigorous physical activity | rs4886868 | G | T | 0.586 | 0.012 | 0.002 | 3.50E-08 | 30.405 |
| Moderate to vigorous physical activity | rs429358 | C | T | 0.154 | 0.022 | 0.003 | 6.10E-13 | 51.824 |
| Moderate to vigorous physical activity | rs1921981 | A | G | 0.326 | -0.013 | 0.002 | 3.80E-08 | 30.224 |

**Abbreviation:** SNPs: single nucleotide polymorphisms; EA: effect allele; OA: other allele; SE: standard error; HDL: High-density lipoprotein; LDL: Low-density lipoprotein.

**Supplementary Table 3**. Specific information for univariable MR analysis of sleep disorders, dietary factors, metabolic factors and physical activity as exposure and multiple sclerosis as outcome

| **Exposure** | **Method** | **SNPs** | **beta** | **SE** | **OR** | **95%CI** | **p-value** |
| --- | --- | --- | --- | --- | --- | --- | --- |
| Snoring | MR Egger | 31 | 5.226 | 3.778 | 186.127 | 0.113-(3.06E+05) | 0.177 |
|  | Weighted median | 31 | 0.197 | 0.703 | 1.218 | 0.307-4.833 | 0.779 |
|  | Inverse variance weighted | 31 | 0.461 | 0.567 | 1.585 | 0.522-4.816 | 0.416 |
|  | Simple mode | 31 | -1.062 | 1.362 | 0.346 | 0.024-4.993 | 0.442 |
|  | Weighted mode | 31 | -0.859 | 1.404 | 0.424 | 0.027-6.636 | 0.545 |
| Chronotype | MR Egger | 8 | -2.039 | 0.845 | 0.130 | 0.025-0.682 | 0.052 |
|  | Weighted median | 8 | -0.871 | 0.363 | 0.419 | 0.206-0.852 | 0.016 |
|  | Inverse variance weighted | 8 | -0.561 | 0.278 | 0.571 | 0.331-0.984 | 0.044 |
|  | Simple mode | 8 | -0.952 | 0.665 | 0.386 | 0.105-1.422 | 0.196 |
|  | Weighted mode | 8 | -1.057 | 0.580 | 0.347 | 0.111-1.083 | 0.111 |
| Sleep duration | MR Egger | 50 | -0.956 | 0.937 | 0.384 | 0.061-2.413 | 0.313 |
|  | Weighted median | 50 | 0.211 | 0.341 | 1.235 | 0.632-2.411 | 0.537 |
|  | Inverse variance weighted | 50 | -0.107 | 0.240 | 0.899 | 0.561-1.440 | 0.657 |
|  | Simple mode | 50 | 0.052 | 0.683 | 1.053 | 0.276-4.014 | 0.940 |
|  | Weighted mode | 50 | 0.186 | 0.536 | 1.205 | 0.421-3.447 | 0.730 |
| Short sleep duration | MR Egger | 9 | 3.040 | 3.284 | 20.907 | 0.033-(1.31E+04) | 0.385 |
|  | Weighted median | 9 | -1.383 | 0.916 | 0.251 | 0.042-1.512 | 0.131 |
|  | Inverse variance weighted | 9 | -1.772 | 0.710 | 0.170 | 0.042-0.684 | 0.013 |
|  | Simple mode | 9 | -1.024 | 1.253 | 0.359 | 0.031-4.183 | 0.437 |
|  | Weighted mode | 9 | -0.927 | 1.151 | 0.396 | 0.042-3.775 | 0.444 |
| Long sleep duration | MR Egger | 10 | -0.227 | 2.110 | 0.797 | 0.013-49.806 | 0.917 |
|  | Weighted median | 10 | 0.181 | 0.962 | 1.199 | 0.182-7.897 | 0.850 |
|  | Inverse variance weighted | 10 | 0.267 | 0.810 | 1.306 | 0.267-6.385 | 0.742 |
|  | Simple mode | 10 | 0.075 | 1.491 | 1.078 | 0.058-20.031 | 0.961 |
|  | Weighted mode | 10 | 0.123 | 1.452 | 1.131 | 0.066-19.464 | 0.934 |
| Sleep apnea syndrome | MR Egger | 22 | 0.208 | 0.262 | 1.231 | 0.736-2.058 | 0.438 |
|  | Weighted median | 22 | 0.111 | 0.083 | 1.117 | 0.948-1.316 | 0.185 |
|  | Inverse variance weighted | 22 | -0.016 | 0.065 | 0.985 | 0.867-1.118 | 0.811 |
|  | Simple mode | 22 | 0.189 | 0.212 | 1.208 | 0.797-1.832 | 0.384 |
|  | Weighted mode | 22 | 0.222 | 0.184 | 1.249 | 0.871-1.792 | 0.241 |
| Sleeplessness/insomnia | MR Egger | 30 | -0.796 | 1.148 | 0.451 | 0.048-4.276 | 0.493 |
|  | Weighted median | 30 | 0.216 | 0.449 | 1.241 | 0.515-2.989 | 0.631 |
|  | Inverse variance weighted | 30 | 0.087 | 0.341 | 1.091 | 0.559-2.131 | 0.798 |
|  | Simple mode | 30 | 0.473 | 0.854 | 1.605 | 0.301-8.562 | 0.584 |
|  | Weighted mode | 30 | 0.124 | 0.731 | 1.132 | 0.270-4.742 | 0.866 |
| Tea intake | MR Egger | 29 | 0.396 | 0.530 | 1.486 | 0.526-4.198 | 0.461 |
|  | Weighted median | 29 | -0.687 | 0.292 | 0.503 | 0.284-0.891 | 0.019 |
|  | Inverse variance weighted | 29 | -0.330 | 0.214 | 0.719 | 0.473-1.094 | 0.123 |
|  | Simple mode | 29 | -0.807 | 0.549 | 0.446 | 0.152-1.309 | 0.153 |
|  | Weighted mode | 29 | -0.790 | 0.660 | 0.454 | 0.124-1.656 | 0.242 |
| Beef intake | MR Egger | 6 | -6.918 | 4.787 | 0.001 | 8.34E-08-11.755 | 0.222 |
|  | Weighted median | 6 | -1.231 | 0.816 | 0.292 | 0.059-1.444 | 0.131 |
|  | Inverse variance weighted | 6 | -0.613 | 0.702 | 0.542 | 0.137-2.143 | 0.382 |
|  | Simple mode | 6 | -1.313 | 1.311 | 0.269 | 0.021-3.509 | 0.362 |
|  | Weighted mode | 6 | -1.384 | 0.934 | 0.251 | 0.040-1.564 | 0.199 |
| Pork intake | MR Egger | 10 | 0.026 | 4.826 | 1.026 | (8.01E-05)-(1.31E+04) | 0.996 |
|  | Weighted median | 10 | 2.135 | 0.822 | 8.459 | 1.689-42.366 | 0.009 |
|  | Inverse variance weighted | 10 | 1.912 | 0.683 | 6.764 | 1.772-25.815 | 0.005 |
|  | Simple mode | 10 | 2.749 | 1.397 | 15.633 | 1.012-241.585 | 0.081 |
|  | Weighted mode | 10 | 2.611 | 1.350 | 13.608 | 0.966-191.724 | 0.085 |
| Coffee intake | MR Egger | 33 | 0.551 | 0.508 | 1.735 | 0.641-4.698 | 0.286 |
|  | Weighted median | 33 | 0.512 | 0.347 | 1.669 | 0.846-3.291 | 0.140 |
|  | Inverse variance weighted | 33 | -0.071 | 0.238 | 0.931 | 0.584-1.486 | 0.765 |
|  | Simple mode | 33 | -1.480 | 0.788 | 0.228 | 0.049-1.067 | 0.070 |
|  | Weighted mode | 33 | 0.638 | 0.439 | 1.893 | 0.801-4.475 | 0.156 |
| Poultry intake | MR Egger | 7 | -19.004 | 26.103 | 5.58E-09 | (3.36E-31)-(9.25E+13) | 0.499 |
|  | Weighted median | 7 | -0.628 | 0.901 | 0.533 | 0.091-3.121 | 0.486 |
|  | Inverse variance weighted | 7 | -0.096 | 0.840 | 0.908 | 0.175-4.711 | 0.909 |
|  | Simple mode | 7 | -1.050 | 1.254 | 0.350 | 0.030-4.092 | 0.435 |
|  | Weighted mode | 7 | -1.072 | 1.221 | 0.342 | 0.031-3.749 | 0.414 |
| Cheese intake | MR Egger | 49 | -1.129 | 0.908 | 0.323 | 0.054-1.917 | 0.220 |
|  | Weighted median | 49 | -0.220 | 0.266 | 0.803 | 0.476-1.353 | 0.410 |
|  | Inverse variance weighted | 49 | 0.051 | 0.208 | 1.053 | 0.701-1.581 | 0.804 |
|  | Simple mode | 49 | -0.396 | 0.551 | 0.673 | 0.228-1.983 | 0.476 |
|  | Weighted mode | 49 | -0.312 | 0.439 | 0.732 | 0.309-1.732 | 0.481 |
| Dried fruit intake | MR Egger | 35 | -1.273 | 1.269 | 0.280 | 0.023-3.367 | 0.323 |
|  | Weighted median | 35 | -0.357 | 0.385 | 0.700 | 0.329-1.488 | 0.354 |
|  | Inverse variance weighted | 35 | -0.453 | 0.267 | 0.636 | 0.377-1.072 | 0.089 |
|  | Simple mode | 35 | -0.053 | 0.820 | 0.949 | 0.190-4.730 | 0.949 |
|  | Weighted mode | 35 | -0.196 | 0.755 | 0.822 | 0.187-3.607 | 0.796 |
| Processed meat intake | MR Egger | 17 | 1.936 | 2.687 | 6.934 | 0.036-(1.34E+03) | 0.482 |
|  | Weighted median | 17 | 0.150 | 0.468 | 1.162 | 0.464-2.911 | 0.748 |
|  | Inverse variance weighted | 17 | 0.342 | 0.415 | 1.408 | 0.624-3.178 | 0.410 |
|  | Simple mode | 17 | -0.314 | 0.909 | 0.730 | 0.123-4.336 | 0.734 |
|  | Weighted mode | 17 | -0.358 | 0.921 | 0.699 | 0.115-4.251 | 0.703 |
| Salad/raw vegetable intake | MR Egger | 9 | -7.079 | 5.346 | 0.001 | (2.37E-08)-29.947 | 0.227 |
|  | Weighted median | 9 | -0.854 | 0.915 | 0.426 | 0.071-2.556 | 0.350 |
|  | Inverse variance weighted | 9 | 0.155 | 0.824 | 1.168 | 0.232-5.873 | 0.851 |
|  | Simple mode | 9 | -0.853 | 1.156 | 0.426 | 0.044-4.109 | 0.482 |
|  | Weighted mode | 9 | -0.907 | 1.035 | 0.404 | 0.053-3.073 | 0.407 |
| Average weekly red wine intake | MR Egger | 11 | -1.228 | 1.200 | 0.293 | 0.028-3.079 | 0.333 |
|  | Weighted median | 11 | 0.096 | 0.504 | 1.101 | 0.410-2.954 | 0.848 |
|  | Inverse variance weighted | 11 | 0.106 | 0.401 | 1.112 | 0.506-2.443 | 0.791 |
|  | Simple mode | 11 | 0.204 | 0.857 | 1.226 | 0.228-6.578 | 0.817 |
|  | Weighted mode | 11 | -0.101 | 0.820 | 0.904 | 0.181-4.509 | 0.904 |
| Fish/liver oil dietary supplements | MR Egger | 30 | -5.148 | 3.687 | 0.006 | (4.22E-06)-7.992 | 0.174 |
|  | Weighted median | 30 | -1.827 | 0.857 | 0.161 | 0.030-0.862 | 0.033 |
|  | Inverse variance weighted | 30 | -2.124 | 0.669 | 0.119 | 0.032-0.444 | 0.002 |
|  | Simple mode | 30 | -0.925 | 1.824 | 0.397 | 0.011-14.169 | 0.616 |
|  | Weighted mode | 30 | -1.514 | 1.608 | 0.220 | 0.009-5.141 | 0.354 |
| Body mass index | MR Egger | 62 | 0.338 | 0.294 | 1.402 | 0.788-2.494 | 0.255 |
|  | Weighted median | 62 | 0.204 | 0.135 | 1.226 | 0.941-1.598 | 0.131 |
|  | Inverse variance weighted | 62 | 0.298 | 0.102 | 1.347 | 1.103-1.644 | 0.004 |
|  | Simple mode | 62 | 0.396 | 0.282 | 1.486 | 0.856-2.580 | 0.165 |
|  | Weighted mode | 62 | 0.225 | 0.183 | 1.252 | 0.875-1.794 | 0.224 |
| Waist-to-hip ratio | MR Egger | 27 | 1.097 | 0.623 | 2.995 | 0.883-10.154 | 0.091 |
|  | Weighted median | 27 | -0.058 | 0.195 | 0.944 | 0.643-1.384 | 0.767 |
|  | Inverse variance weighted | 27 | -0.061 | 0.146 | 0.940 | 0.707-1.251 | 0.673 |
|  | Simple mode | 27 | -0.029 | 0.399 | 0.971 | 0.444-2.124 | 0.942 |
|  | Weighted mode | 27 | 0.204 | 0.339 | 1.226 | 0.631-2.383 | 0.553 |
| Hip circumference | MR Egger | 46 | 0.162 | 0.303 | 1.176 | 0.650-2.129 | 0.595 |
|  | Weighted median | 46 | 0.316 | 0.146 | 1.371 | 1.030-1.826 | 0.030 |
|  | Inverse variance weighted | 46 | 0.237 | 0.104 | 1.267 | 1.033-1.555 | 0.023 |
|  | Simple mode | 46 | 0.174 | 0.251 | 1.190 | 0.728-1.944 | 0.492 |
|  | Weighted mode | 46 | 0.262 | 0.176 | 1.299 | 0.920-1.834 | 0.144 |
| Waist circumference | MR Egger | 17 | -0.173 | 0.492 | 0.841 | 0.321-2.205 | 0.729 |
|  | Weighted median | 17 | 0.280 | 0.214 | 1.323 | 0.869-2.014 | 0.192 |
|  | Inverse variance weighted | 17 | 0.326 | 0.167 | 1.385 | 0.999-1.921 | 0.051 |
|  | Simple mode | 17 | 0.268 | 0.373 | 1.307 | 0.629-2.716 | 0.484 |
|  | Weighted mode | 17 | 0.226 | 0.266 | 1.254 | 0.744-2.113 | 0.408 |
| Essential hypertension | MR Egger | 28 | -0.261 | 0.202 | 0.771 | 0.519-1.144 | 0.207 |
|  | Weighted median | 28 | 0.014 | 0.071 | 1.015 | 0.884-1.165 | 0.837 |
|  | Inverse variance weighted | 28 | 0.017 | 0.051 | 1.018 | 0.922-1.123 | 0.731 |
|  | Simple mode | 28 | -0.009 | 0.146 | 0.991 | 0.745-1.318 | 0.952 |
|  | Weighted mode | 28 | 0.018 | 0.133 | 1.019 | 0.785-1.322 | 0.890 |
| HDL cholesterol levels | MR Egger | 57 | 0.036 | 0.097 | 1.037 | 0.858-1.253 | 0.711 |
|  | Weighted median | 57 | 0.069 | 0.080 | 1.072 | 0.916-1.254 | 0.388 |
|  | Inverse variance weighted | 57 | 0.079 | 0.057 | 1.082 | 0.967-1.210 | 0.171 |
|  | Simple mode | 57 | 0.296 | 0.174 | 1.344 | 0.957-1.889 | 0.094 |
|  | Weighted mode | 57 | 0.096 | 0.070 | 1.100 | 0.959-1.263 | 0.180 |
| LDL cholesterol levels | MR Egger | 30 | -0.165 | 0.089 | 0.848 | 0.712-1.011 | 0.076 |
|  | Weighted median | 30 | -0.132 | 0.061 | 0.876 | 0.778-0.987 | 0.030 |
|  | Inverse variance weighted | 30 | -0.080 | 0.069 | 0.923 | 0.807-1.056 | 0.244 |
|  | Simple mode | 30 | -0.042 | 0.197 | 0.959 | 0.652-1.411 | 0.834 |
|  | Weighted mode | 30 | -0.106 | 0.061 | 0.900 | 0.798-1.014 | 0.094 |
| Type 2 diabetes mellitus | MR Egger | 21 | 0.068 | 0.101 | 1.071 | 0.878-1.306 | 0.509 |
|  | Weighted median | 21 | 0.102 | 0.056 | 1.108 | 0.992-1.237 | 0.069 |
|  | Inverse variance weighted | 21 | 0.115 | 0.054 | 1.122 | 1.010-1.247 | 0.032 |
|  | Simple mode | 21 | 0.136 | 0.117 | 1.145 | 0.911-1.440 | 0.258 |
|  | Weighted mode | 21 | 0.104 | 0.058 | 1.110 | 0.990-1.244 | 0.090 |
| Vigorous physical activity | MR Egger | 6 | 0.740 | 6.775 | 2.096 | (3.58E-06)-(1.23E+06) | 0.918 |
|  | Weighted median | 6 | -0.672 | 0.984 | 0.511 | 0.074-3.510 | 0.494 |
|  | Inverse variance weighted | 6 | -0.345 | 0.779 | 0.708 | 0.154-3.261 | 0.658 |
|  | Simple mode | 6 | -1.190 | 1.390 | 0.304 | 0.020-4.640 | 0.431 |
|  | Weighted mode | 6 | -1.128 | 1.358 | 0.324 | 0.023-4.635 | 0.444 |
| Strenuous sports or other exercises | MR Egger | 12 | -2.621 | 4.066 | 0.073 | (2.51E-05)-(2.10E+02) | 0.534 |
|  | Weighted median | 12 | -1.215 | 0.977 | 0.297 | 0.044-2.014 | 0.214 |
|  | Inverse variance weighted | 12 | -0.357 | 0.696 | 0.700 | 0.179-2.736 | 0.608 |
|  | Simple mode | 12 | -1.917 | 1.670 | 0.147 | 0.006-3.883 | 0.275 |
|  | Weighted mode | 12 | -1.757 | 1.453 | 0.173 | 0.010-2.978 | 0.252 |
| Moderate to vigorous physical activity | MR Egger | 18 | -3.609 | 1.924 | 0.027 | 0.001-1.175 | 0.079 |
|  | Weighted median | 18 | -1.253 | 0.430 | 0.286 | 0.123-0.663 | 0.004 |
|  | Inverse variance weighted | 18 | -1.265 | 0.323 | 0.282 | 0.150-0.532 | 9.16E-05 |
|  | Simple mode | 18 | -1.121 | 0.779 | 0.326 | 0.071-1.500 | 0.168 |
|  | Weighted mode | 18 | -1.102 | 0.774 | 0.332 | 0.073-1.514 | 0.172 |

**Abbreviation:** SNPs: single nucleotide polymorphisms; EA: effect allele; OA: other allele; SE: standard error; HDL: High-density lipoprotein; LDL: Low-density lipoprotein

**Supplementary Table 4** Specific information for multivariable MR analysis of sleep disorders, dietary factors, metabolic factors and physical activity as exposure and multiple sclerosis as outcome

| Exposure/Confounder | Method | SNPs | beta | SE | OR | 95%CI | p-value |
| --- | --- | --- | --- | --- | --- | --- | --- |
| Past tobacco smoking | Inverse variance weighted | 34 | -0.005 | 0.226 | 0.995 | 0.639-1.548 | 0.981 |
| Particulate matter air pollution | Inverse variance weighted | 5 | -0.330 | 0.764 | 0.719 | 0.161-3.217 | 0.666 |
| Chronotype | Inverse variance weighted | 8 | -0.310 | 0.352 | 0.734 | 0.368-1.463 | 0.379 |
| Past tobacco smoking | Inverse variance weighted | 35 | -0.038 | 0.233 | 0.963 | 0.610-1.520 | 0.871 |
| Particulate matter air pollution | Inverse variance weighted | 5 | -0.647 | 0.827 | 0.524 | 0.104-2.647 | 0.434 |
| Short sleep duration | Inverse variance weighted | 4 | 1.569 | 2.285 | 4.801 | 0.545-12.291 | 0.492 |
| Past tobacco smoking | Inverse variance weighted | 34 | -0.221 | 0.184 | 0.802 | 0.559-1.149 | 0.229 |
| Particulate matter air pollution | Inverse variance weighted | 4 | -0.381 | 0.686 | 0.683 | 0.178-2.619 | 0.578 |
| Pork intake | Inverse variance weighted | 10 | 1.321 | 0.641 | 3.749 | 1.067-13.166 | 0.039 |
| Past tobacco smoking | Inverse variance weighted | 34 | -0.061 | 0.227 | 0.940 | 0.603-1.467 | 0.787 |
| Particulate matter air pollution | Inverse variance weighted | 5 | -0.497 | 0.779 | 0.608 | 0.132-2.798 | 0.523 |
| Fish/liver oil dietary supplements | Inverse variance weighted | 4 | -3.003 | 1.836 | 0.050 | 0.001-1.816 | 0.102 |
| Past tobacco smoking | Inverse variance weighted | 22 | 0.064 | 0.227 | 1.066 | 0.684-1.663 | 0.776 |
| Particulate matter air pollution | Inverse variance weighted | 3 | -0.502 | 0.728 | 0.606 | 0.145-2.521 | 0.491 |
| Body mass index | Inverse variance weighted | 56 | 0.280 | 0.131 | 1.323 | 1.024-1.711 | 0.032 |
| Past tobacco smoking | Inverse variance weighted | 24 | -0.126 | 0.229 | 0.322 | 0.563-1.380 | 0.581 |
| Particulate matter air pollution | Inverse variance weighted | 3 | 0.463 | 0.764 | 1.961 | 0.356-7.105 | 0.544 |
| Hip circumference | Inverse variance weighted | 43 | 0.135 | 0.131 | 0.392 | 0.885 -1.480 | 0.304 |
| Past tobacco smoking | Inverse variance weighted | 31 | -0.106 | 0.232 | 0.899 | 0.571-1.417 | 0.647 |
| Particulate matter air pollution | Inverse variance weighted | 3 | -0.956 | 0.830 | 0.384 | 0.076-1.955 | 0.249 |
| Type 2 diabetes mellitus | Inverse variance weighted | 33 | 0.141 | 0.051 | 1.152 | 1.042-1.272 | 0.005 |
| Past tobacco smoking | Inverse variance weighted | 33 | -0.244 | 0.166 | 0.783 | 0.566-1.085 | 0.141 |
| Particulate matter air pollution | Inverse variance weighted | 5 | -0.324 | 0.561 | 0.723 | 0.241-2.173 | 0.564 |
| Moderate to vigorous physical activity | Inverse variance weighted | 17 | -1.439 | 0.324 | 0.237 | 0.126-0.448 | 9.18E-06 |

SNPs: single nucleotide polymorphisms; EA: effect allele; OA: other allele; SE: standard error.
